# Intertwining Olefin Thianthrenation with Kornblum/Ganem Oxidations: Ene‐type Oxidation to Furnish α,β‐Unsaturated Carbonyls

**DOI:** 10.1002/anie.202214096

**Published:** 2022-12-02

**Authors:** Péter Angyal, András M. Kotschy, Ádám Dudás, Szilárd Varga, Tibor Soós

**Affiliations:** ^1^ Institute of Organic Chemistry Research Centre for Natural Sciences Magyar tudósok körútja 2 1117 Budapest Hungary; ^2^ Hevesy György PhD School of Chemistry Eötvös Loránd University Pázmány Péter sétány 1/A 1117 Budapest Hungary

**Keywords:** Alkenes, Alkenyl Electrophiles, C−H Functionalization, Oxidation, Thianthrenation

## Abstract

A widely applicable, practical, and scalable synthetic method for efficient ene‐type double oxidation of alkenes is reported via a two‐step alkenyl thianthrenium umpolung/Kornblum‐Ganem oxidation strategy. This chemo‐ and stereoselective procedure allows easy access to various α,β‐unsaturated carbonyls that may be otherwise difficult or cumbersome to synthesize by conventional methods. For α‐olefins, this metal‐free transformation can be tuned according to synthetic needs to produce either the elusive (*Z*)‐unsaturated aldehydes or their (*E*) counterparts. Moreover, this strategy has enabled streamlined synthesis of distinct butadienyl pheromones and kairomones.

## Graphical abstract

A metal‐free double oxidation of unactivated alkenes has been developed to deliver unsaturated conjugated carbonyls. This practical procedure allows valorization of the olefinic feedstock with high chemo‐ and stereoselectivity as demonstrated by the syntheses of difficult‐to‐access building blocks and industrially relevant pheromones and kairomones.

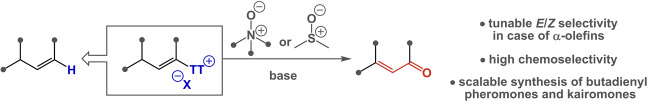

Despite their ubiquity and wide‐ranging synthetic applicability, the synthesis of α,β‐unsaturated carbonyl compounds is still often a tedious and challenging transformation.^[1]^ Among established and emerging approaches for obtaining these molecules,[^2^, ^3^, ^4^] one particularly appealing strategy is the allylic C−H oxidation of olefins.^[5]^ Nevertheless, these straightforward processes typically use toxic reagents^[6]^ (based around elements such as selenium or chromium) or invoke transition metal catalysts^[7]^ (such as palladium, rhodium, and ruthenium). Furthermore, the regio‐ and stereoselective oxidation of olefins, especially that of α‐olefins, is still a limiting factor.^[8]^ Given the prevalence of α,β‐unsaturated carbonyl compounds in synthetic organic chemistry and some of the limitations of their current synthesis, we pursued a distinct, transition metal‐free synthetic approach. Herein we describe an operationally straightforward, intertwined thianthrenation (TT)‐Kornblum/Ganem method which facilitates the synthesis of unsaturated carbonyls from non‐activated olefins.

Owing to the abundance and diversity of the olefin feedstock, the efficient and practical functionalization of olefinic bond has been a constant preoccupation in organic chemistry. In this context, the recently discovered olefin umpolung via direct alkenyl sulfonium salt formation creates a new opportunity for unactivated olefin functionalization. In a series of seminal works, Mukaiyama described the direct synthesis of alkenyl diphenyl sulfonium salts and illuminated their distinct reactivities, such as 1,2‐dielectrophilic addition and allylic electrophilic substitution.^[9]^ The narrow synthetic scope of diphenyl sulfonium salts, however, hampered the spread of this synthetic tactic.^[10]^ These challenges could be mitigated by switching to alkenyl thianthrenium salts,^[11]^ pioneered by Shine.^[12]^ Based on this recognition, Ritter's group has brought a further breakthrough in this field as their advances allowed the robust synthesis of alkenyl thianthrenium salts (**1 a**) from a broad range of olefins in a stereo‐ and regioselective manner.^[13]^ Additionally, these sulfonium salts served as an alkenyl electrophile in various cross‐coupling reactions (Figure 1A). The enabling element of Ritter's modification was the efficient and easy assembly of the dicationic thianthrenium salt precursor (**1 b**) from a broad range of olefins via an inverse electron‐demand Diels–Alder reaction.

**Figure 1:**
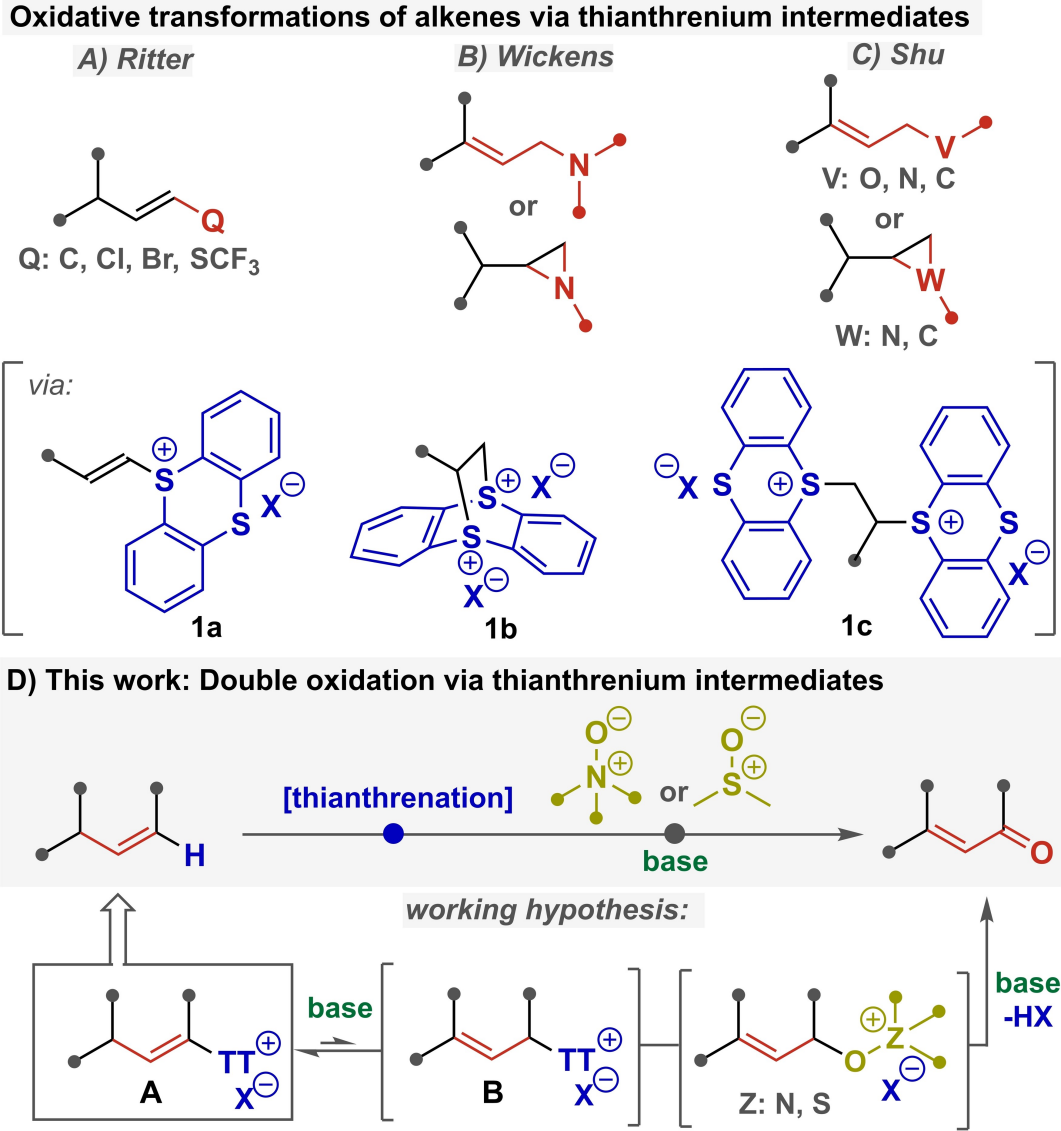
Introduction to single and double oxidative transformations of alkenyl thianthrenium salts.

Subsequently, Wickens demonstrated that dicationic (**1 b**) and related metastable bis‐adducts (**1 c**) are readily generated by means of electrochemistry and their reactivity can be directed to furnish either aziridines or allylic amines (Figure 1B).^[14]^ Independently, Shu reported a similar allylic functionalization to effect ene‐type olefin amination, oxygenation, carbonation and more recently aziridination and cyclopropanation (Figure 1C).^[15]^ Importantly, the allylic transformations of **1 a**–**c** thianthrenium salts provided preferably (*Z*)‐allylic products. Thus, this method complements more conventional single oxidation methodologies (e.g., White's allylic oxidation^[16]^), which usually favor (*E*)‐selectivity.

Having dissected the distinct reactivity of alkenyl thianthrenium salts, we addressed the question of whether the olefin umpolung through thianthrenation can be intertwined with a Kornblum/Ganem‐like oxidation step^[17]^ to afford α,β‐unsaturated carbonyl compounds. More specifically, we hypothesized (Figure 1D) that alkenyl thianthrenium salts **A** are in a base‐catalyzed tautomeric equilibrium with allyl‐type thianthrenium salts **B**[^18^, ^19^] and the more reactive allyl tautomers can be selectively intercepted with relatively weak nucleophiles such as sulfoxides or *N*‐oxides. The resulting allyloxy‐sulphonium/ammonium intermediate then affords an unsaturated carbonyl compound after elimination.

First, we explored this idea in the context of α‐olefin oxidation by exposing alkenyl thianthrenium salt **2 a** to DMSO and K_2_CO_3_. Gratifyingly, we could observe the formation of the desired unsaturated aldehyde **3 a** at room temperature (Table 1, **entry 2**). This result supports not only the intermediacy but also the high reactivity of allyl‐thianthrenium salts, as the classical Kornblum reactions are performed at significantly higher temperatures. Optimized reaction conditions were then readily established (Table 1).^[20]^ First, we examined the influence of alternative oxidants on the reaction's efficiency and found that Ganem's modification (**entries 1**, **3**–**5**), especially the utilization of NMO, is advantageous. Nevertheless, using only an equimolar reagent caused diminished yield (**entry 3**). Additional evaluation of less nucleophilic or sterically less hindered *N*‐oxides (PICNO, TMANO) revealed that slight modification of the oxidant significantly lowers the yields (**entries 4**, **5**). These experiments also indicate that the byproduct base competes as a nucleophile in this reaction and forms *N*‐allylated side products (**4**).^[21]^ For similar reasons, even slightly nucleophilic bases proved to be unsuitable to affect the elimination (DBU, **entry 6**). While more hindered amines could suppress the amine‐allylation side‐reaction (2,6‐lutidine, **entry 7**), similarly effective inorganic bases were used to facilitate and simplify the isolation of enal **3 a** and thereby increase the practicality of this method (**entry 8** vs **9**). Importantly, there is a clear kinetic preference for the (*Z*)‐enal formation which might be the result of a Curtin–Hammett scenario (**entries 10**, **11**).^[22]^ Nevertheless, the *Z : E* selectivity can be fully shifted towards the thermodynamically preferred (*E*)‐isomer (**entries 12**, **13**). With the optimized conditions in hand (**entry 1**), we evaluated the generality of the developed reaction's scope (Figure 2, method **A**).

**Table 1:**
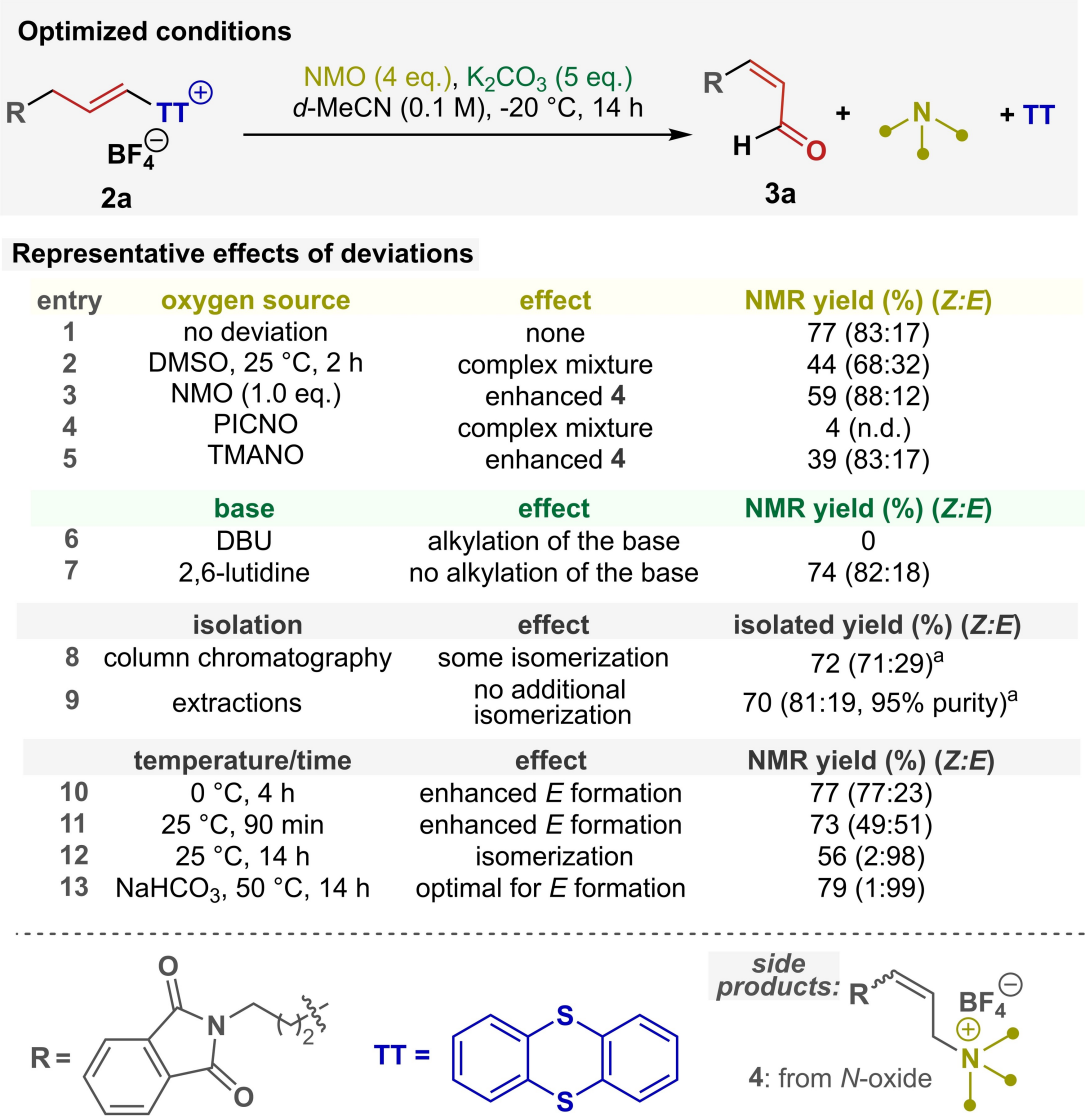
Summary of the optimization study of the TT‐Kornblum reaction.

All reactions were performed on a 0.1 mmol scale under the optimal conditions with the indicated deviations. NMR yields were determined by crude ^1^H qNMR using benzotrifluoride as the internal standard. NMO=*N*‐methylmorpholine *N*‐oxide; DMSO=dimethyl sulfoxide; PICNO=2‐methylpyridine *N*‐oxide; TMANO=trimethylamine *N*‐oxide; DBU=1,8‐diazabicyclo(5.4.0)undec‐7‐ene; n.d.=not determined. [a] Conducted on a 0.4 mmol scale using non‐deuterated solvents.

**Figure 2:**
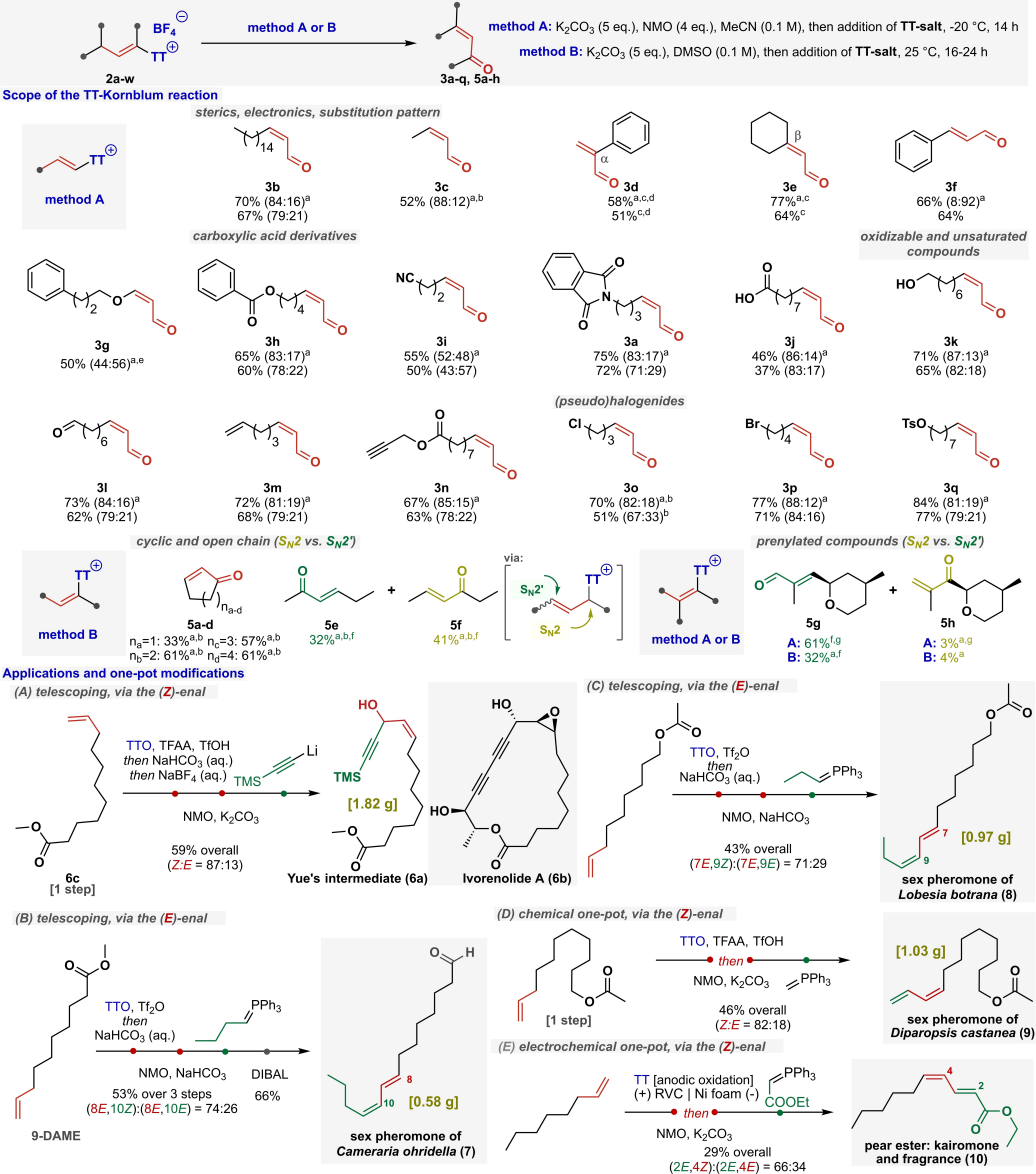
Scope, applications, and one‐pot modifications of the TT‐Kornblum reaction. The values in parenthesis represent the (*Z : E*) ratio of the product as determined by ^1^H qNMR. TTO=thianthrene *S*‐oxide; TFAA=trifluoroacetic anhydride; DIBAL=diisobutylaluminium hydride. 9‐DAME=methyl 9‐decenoate [a] qNMR yield. [b] Volatile compound. [c] Conducted at 0 °C. [d] Starting from the 1 : 1 isomeric mixture of allyl‐ and alkenyl thianthrenium salts. [e] Unstable to be isolated. [f] Only the formation of the (*E*)‐stereoisomer was observed. [g] Conducted at 25 °C.

We initially explored variations of the substituent pattern of the terminal double bond and found that alkyl groups with different lengths (**3 b**–**c**), α‐ or β‐disubstitution (**3 d**–**e**), and aromatic functionalities (**3 f**) were all tolerated. Interestingly, heteroatom connection at the allylic position is allowed, forming the difficult‐to‐access push‐pull olefin **3 g**. Importantly, the synthesis of **3 d** allowed us to gain further insight into the reaction mechanism. In this particular case, the preceding thianthrenation step provided both **2 d** alkenyl and **2 d’** allylic thianthrenium salts as a mixture. The kinetic study of their Kornblum/Ganem oxidation reaction indicated that the productive intermediate was the presumed allylic thianthrenium salt.^[20]^

Furthermore, the method accommodates substrates bearing reactive handles such as ester (**3 h**), nitrile (**3 i**), imide (**3 a**), or unprotected carboxylic acid group (**3 j**).^[23]^ We similarly found that substrates with oxidatively labile functional groups can be used in our oxidative protocol. Thus, alcohol (**3 k**) and various unsaturated functionalities (**3 l**–**n**) such as aldehyde, alkene, and alkyne are all well‐tolerated. Finally, the selective oxidations of alkenes with (pseudo)halogenide functionalities, such as chloride (**3 o**), bromide (**3 p**), and even tosylate (**3 q**), were investigated. While these functional groups are reactive in classical Kornblum/Ganem oxidations, we expected that the high reactivity of the allyl‐thianthrenium moieties allows the chemoselective transformation. Gratifyingly, not only good yields but also exclusive selectivity for olefin oxidation was observed in all cases. Importantly, for nearly all substrates, the elusive (*Z*)‐enals could be obtained with good selectivity (up to 88 : 12). The only exception was the oxidation of the aromatic conjugated system **3 f** that afforded exclusively the (*E*)‐isomer upon isolation. It is also noteworthy that the chemo‐ and stereoselective oxidation of the above substrates to α,β*‐*unsaturated carbonyls is difficult or cumbersome using previous methods.[^8^, ^15a^, ^16^]

Next, we became interested in extending our methodology to internal olefins. We expected that thianthrenated internal olefins can form the desired unsaturated ketones, however, we conceived that these sterically more hindered allyl‐sulfonium intermediates could react not only via S_N_2 but also in an S_N_2′ manner. Indeed, our first presumption was correct, cyclic olefins (**5 a**–**d**) were converted to unsaturated ketones, however, a sterically less demanding oxidant (i.e., DMSO, Figure 2, method B) proved to be the reagent of choice. As these substrates did not allow us to deduce the regioselectivity of the oxidation, we embarked on studying various linear substrates. As shown in Figure 2, we were gratified to find that hex‐3‐ene afforded **5 e** and **5 f** isomers in close to 1 : 1 ratio which confirmed the existence of the competing allylic substitution and reinforced the mechanistic basis of this synthetic manifold. Then, triggered by this novel thianthrenium reactivity, we aimed to shift selectivity toward the S_N_2′ product. Pleasingly, owing to steric effects, the double oxidation of rose oxide via the thianthrenation‐Kornblum sequence favored the S_N_2′ type product with high selectivity (**5 g:5 h**>20 : 1).

Finally, we sought to demonstrate the potential of our method to streamline the synthesis of various natural products (Figure 2A–E). First, to probe whether our methodology could be scaled up and telescoped, the synthesis of Yue's intermediate (**6 a**) towards the immunosuppressant ivorenolide A (**6 b**) was attempted.^[24]^ We were delighted to find that the synthesis of the desired product was achieved via the elusive (*Z*)‐enal intermediate on a gram scale, with enhanced *Z : E* selectivity, starting from the easily accessible **6 c** alkene (Figure 2A). Then, the syntheses of particularly important diene‐type pheromones and kairomones were investigated.^[25]^ The adjustable *Z : E* selectivity of our method is noteworthy in the context of the stereochemical synthesis of these diene natural products. First, the pheromones **7** and **8** of *Cameraria ohridella* (horse chestnut leaf miner moth)^[26]^ and *Lobesia botrana* (European grape moth)^[27]^ were synthesized in a telescopic manner via procedures ensuring selective formation of the intermediate (*E*)‐enals (Figure 2B and C). Furthermore, pheromone **9** of the *Diparopsis castanea* (red bollworm moth)^[28]^ was synthesized through the (*Z*)‐enal via a one‐pot procedure (Figure 2D). Lastly, the compatibility of the downstream Kornblum/Ganem‐Wittig chemistry with activation via Wickens’ cation pool approach was demonstrated by forming the kairomone and fragrance pear ester **10** (Figure 2E).^[29]^

In summary, we have developed a two‐step thianthrenation/Kornblum‐Ganem oxidation protocol for the transition‐metal‐free conversion of olefins into α,β‐unsaturated carbonyls. This synthetic manifold exploits the enhanced reactivity of allyl‐thianthrenium intermediates toward sulfoxides and *N*‐oxides. We demonstrated that this oxidation process could be telescoped, showcasing the utility of this method in streamlined natural product synthesis. We anticipate that this straightforward and highly selective protocol will expand the synthetic toolbox for olefin functionalization and enable complementary strategies for late‐stage functionalization.

## Conflict of interest

The authors declare no conflict of interest.

## Supporting information

As a service to our authors and readers, this journal provides supporting information supplied by the authors. Such materials are peer reviewed and may be re‐organized for online delivery, but are not copy‐edited or typeset. Technical support issues arising from supporting information (other than missing files) should be addressed to the authors.

Supporting Information

Supporting Information

Supporting Information

Supporting Information

## Data Availability

The data that support the findings of this study are available in the supplementary material of this article.
