# Supplementary material for: Intertwining Olefin Thianthrenation with Kornblum/Ganem Oxidations: Ene‐type Oxidation to Furnish α,β‐Unsaturated Carbonyls
Source: Angew Chem Int Ed Engl. 2022 Dec 2;62(2):e202214096. doi: 10.1002/anie.202214096 (PMC10108043; doi:10.1002/anie.202214096)

Angewandte Chemie
Eine Zeitschrift der Gesellschaft Deutscher Chemiker

Supporting Information

Intertwining Olefin Thianthrenation with Kornblum/Ganem Oxidations: Ene-type Oxidation to Furnish α,β-Unsaturated Carbonyls

P. Angyal, A. M. Kotschy, Á. Dudás, S. Varga, T. Soós**

SUPPORTING INFORMATION

Intertwining Olefin Thianthrenation with Kornblum/Ganem Oxidations: Ene-type Oxidation to Furnish α,β-Unsaturated Carbonyls

Péter Angyal[a,b], András M. Kotschy[a,b], Ádám Dudás[a,b], Szilárd Varga*[a], Tibor Soós*[a]

[a] P. Angyal, A. M. Kotschy, Á. Dudás, Dr. Sz. Varga, Dr. T. Soós
Institute of Organic Chemistry,
Research Centre for Natural Sciences
2. Magyar tudósok krt., H-1117 Budapest, Hungary
E-mail: varga.szilard@ttk.hu, soos.tibor@ttk.hu

[b] P. Angyal, A. M. Kotschy, Á. Dudás
Hevesy György PhD School of Chemistry,
Eötvös Loránd University
1/a Pázmány Péter sétány, H-1117 Budapest, Hungary

1. General information S3
2. Preparation of alkenyl thianthren-5-ium tetrafluoroborate salts S4
2.1. General procedure S5
Compound 2a S7
Compound 2b S7
Compound 2g S8
Compound 2h S9
Compound 2i S9
Compound 2m S10
Compound 2n S11
Compound 2o S11
Compound 2q S12
Compound 2v S12
2.2. Specific procedures S14
Compound 2c S14
Compound 2d S15
Compound 2j S17
Compound 2k S18
Compound 2l S19
3. General procedures for the preparation of unsaturated carbonyls S20
Compound 3a S21
Compound 3b S22
Compound 3c S23
Compound 3d S23
Compound 3e S24
Compound 3f S24
Compound 3g S24
Compound 3h S25
Compound 3i S26
Compound 3j S27
Compound 3k S27
Compound 3l S28
Compound 3m S29
Compound 3n S30
Compound 3o S30

Compound 3p S31
Compound 3q S32
Compound 5a S32
Compound 5b S33
Compound 5c S33
Compound 5d S33
Compounds 5e and 5f S33
Compounds 5g and 5h S34
4. Detailed optimization and practical guidance S36
5. Investigation of the reactive intermediates S40
6. Applications and one-pot modifications S41
Application (A) S41
Application (B) S43
Application (C) S46
Application (D) S48
Application (E) S50
NMR spectra S53

1. General information

All reactions were carried out using oven-dried glassware and anhydrous solvents, under an atmosphere of argon unless noted otherwise. All electrochemical oxidations were carried out using an IKA ElectraSyn 2.0 potentiostat (Ident. No. 0020008980, IKA), equipped with an IKA Pro-Divide divided cell (Ident. No. 0040006482, IKA) with a 10 µm frit, and standard IKA electrodes (RVC: Ident. No. 0040002860, IKA; Nickel foam: Ident. No. 0040002861, IKA). Flash chromatography was performed on silica gel (RediSep Gold Normal Phase Silica columns, 20-40 micron) with the indicated eluents. Thin-layer chromatography was performed on silica plates (Kieselgel 60 F_{254}, Merck). Compounds were visualized by UV (254 nm) or Ceric Ammonium Molybdate (CAM) staining. HRMS spectra were obtained using Agilent 6545 LC/ Q-TOF, using electrospray ionization (ESI). ^{1}H- and ^{13}C-NMR spectra were recorded using a Varian 500 MHz INOVA spectrometer. Chemical shifts are referenced to the residual solvent signals ($CDCl_3$: δ = 7.26 ppm for ^{1}H, δ = 77.0 ppm for ^{13}C, DMSO-d^6: δ = 2.50 ppm for ^{1}H, δ = 39.5 ppm for ^{13}C). ^{19}F-NMR spectra were recorded using a Varian 300 MHz INOVA spectrometer. ^{19}F-NMR spectra were referenced externally to benzotrifluoride at -63.72 ppm. Data are reported as follows: chemical shifts (ppm), multiplicity (s = singlet, d = doublet, t = triplet, q = quartet, br = broad, m = multiplet), and coupling constants (Hz). All spectra were recorded with the standard spectrometer pulse sequences and settings, ^{1}H decoupling was used for ^{13}C-NMR measurements. The stereochemistry of the unsaturated carbonyls was determined based on the *J* coupling constants of the representative multiplets in the ^{1}H-NMR spectra. The minor stereoisomers were marked with * on the spectra if their amount was higher than 10 mol% (based on ^{1}H-NMR integrals). If indicated, low-temperature thermostat cooled reactions were carried out using a JULABO FT902 FT immersion cooler. All starting materials were purchased from Aldrich, TCI, or Fluorochem and used without further purification unless stated otherwise. Anhydrous tetrahydrofuran was distilled from sodium/benzophenone, while dichloromethane (stabilized with amylene) and acetonitrile from calcium hydride.

2. Preparation of alkenyl thianthren-5-ium tetrafluoroborate salts

Alkenyl thianthren-5-ium salts shown below were reported by Ritter and Shu and were synthesized according to their general procedures with slight modifications.[1] Please see these references for their characterization as well as for graphical supporting information.

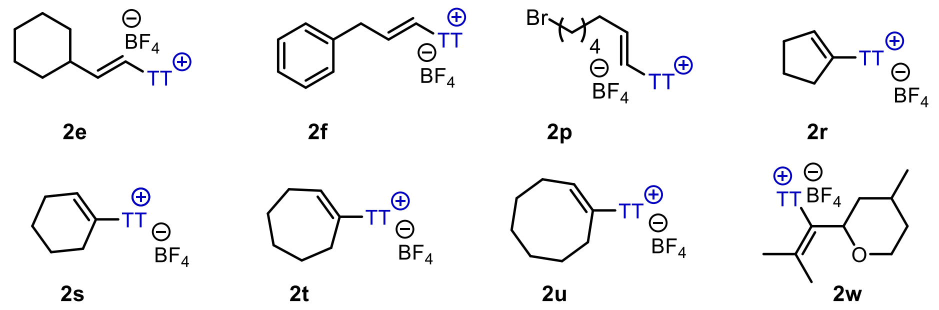

Figure 1. Known alkenyl thianthren-5-ium salts

Additional alkenyl thianthren-5-ium salts were synthesized according to the above-mentioned general procedures with slight modifications or by specific methods as listed below.

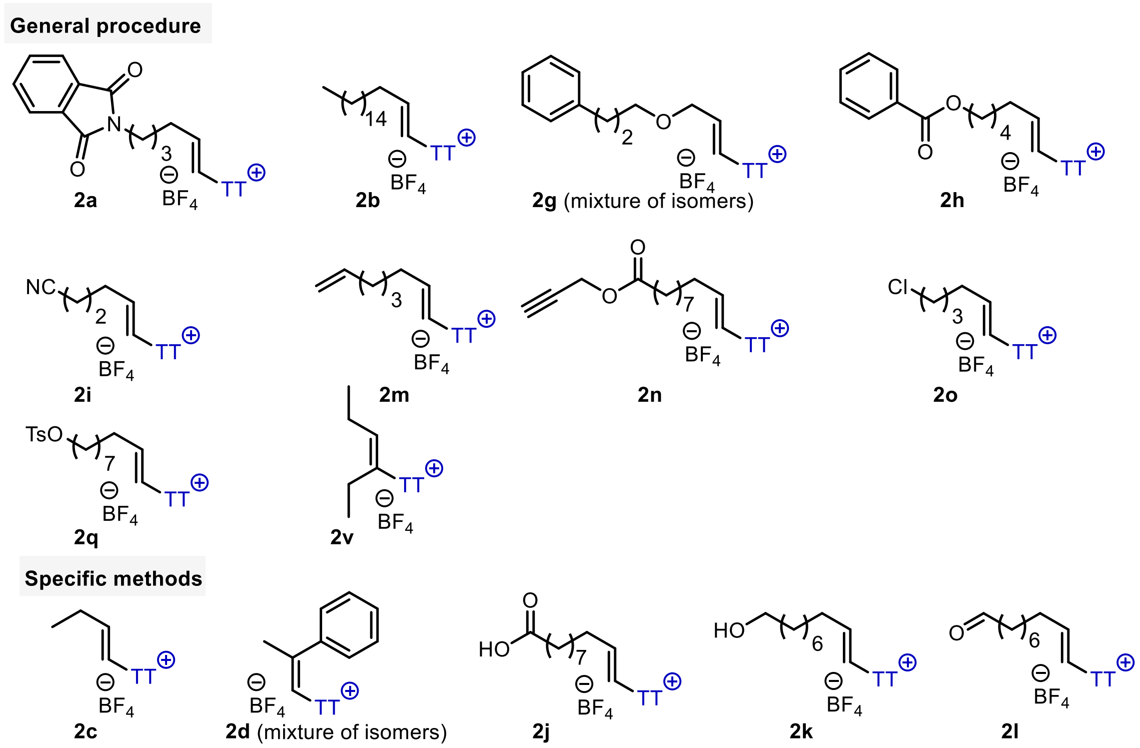

Figure 2. Previously unknown alkenyl thianthren-5-ium salts and the ones prepared by modified procedures

[1] (a) J. Chen, J. Li, M. B. Plutschack, F. Berger, T. Ritter, *Angew. Chem.* **2020**, *132*, 5665-5669.; *Angew. Chem. Int. Ed.* **2020**, *59*, 5616-5620 (b) M.-S. Liu, H.-W. Du, W. Shu, *Chem. Sci.* **2022**, *13*, 1003-1008.

All commercially available olefins were purchased from Aldrich, TCI, or Fluorochem and used without further purification. N-(5-hexenyl)phthalimide[2], (3-(allyloxy)propyl)benzene[3], hept-6-en-1-yl benzoate[4], prop-2-yn-1-yl undec-10-enoate[5] and dec-9-en-1-yl 4-methyl-benzene-sulfonate[6] were prepared according to previously reported procedures.

2.1. General procedure

Preparation of alkenyl thianthren-5-ium salts

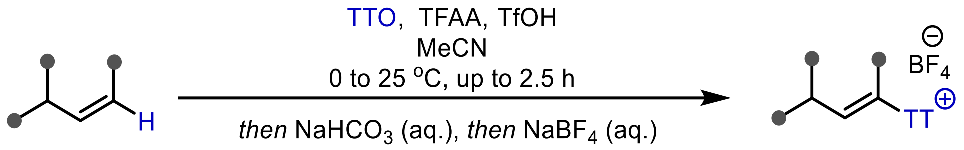

A suspension of alkene (3.15 mmol, 1 equiv) and thianthrene S-oxide (TTO, 754 mg, 3.24 mmol, 1.03 equiv) in anhydrous acetonitrile (10 mL, 0.3 M) was cooled to 0 °C under argon atmosphere. [Note 1] At this temperature, trifluoroacetic anhydride (TFAA, 1.33 mL, 9.45 mmol, 3.00 equiv) and trifluoromethanesulfonic acid (560 µL, 6.30 mmol, 2.00 equiv) were added dropwise consecutively. An immediate color change of the reaction mixture was observed at the beginning of the first addition step and the lilac/blue solution was stirred at 0 °C for a further 1 h. [Note 2] Then, the reaction mixture was warmed to 25 °C and stirred at this temperature for an additional 30 min-1.5 h until analysis by TLC indicated full conversion. [Note 3] At this point, the reaction mixture was concentrated under reduced pressure [Note 4] and subsequently dissolved in dichloromethane (30 mL). Saturated aqueous sodium bicarbonate solution (30 mL) was added, and the two-phase mixture was vigorously stirred at 25 °C for 15 min, whereupon a color change to yellow/brown was observed and the phases were separated. [Notes 5,6] This washing step was repeated one more time, after which aqueous sodium tetrafluoroborate solution (5 wt.%, 50 mL) was added to the organic phase and the mixture was stirred vigorously for 15 min and the phases were separated. This washing step was repeated as well one more time. [Note 7] The layers were separated, and the organic phase was dried over sodium sulfate. The dried solution was filtered, and the filtrate was concentrated under reduced pressure. The residue was purified by flash column chromatography on silica gel (gradient elution using isopropanol and dichloromethane) to yield alkenyl thiantren-5-ium tetrafluoroborate salts as a mixture of *E*/*Z* isomers. [Notes 8,9]

[Note 1]: An ice bath was used.
[Note 2]: In the presence of functional groups which are likely to be acetylated by trifluoroacetic anhydride (e.g., alcohols, carboxylic acids), this color change is not immediate but occurs during addition.

[2] L. Qin, X. Ren, Y. Lu, Y. Li, J. Zhou, *Angew. Chem.* **2012**, *124*, 6017-6021; *Angew. Chem. Int. Ed.* **2012**, *51*, 5915-5919
[3] A. Takahiko, H. Hajimu, O. Shorichiro, *Bull. Chem. Soc. Jpn.* **1992**, *65*, 1932-1938
[4] D. A. Cruz, V. Sinka, P. de Armas, H. S. Steingruber, I. Fernández, V. S. Martin, P. O. Miranda, J. I. Padrón, *Org. Lett.* **2021**, *23* (15), 6105-6109
[5] B. Gandhi, K. Greeshma, D. P. Ruvulapalli, S. S. Kaki, *Med. Chem. Res.* **2022**, *31*, 1558-1570
[6] J. Lee, M. M. Joullié, *Tetrahedron Lett.* **2015**, *56*, 3378-3381

[Note 3]: Alkenyl thianthren-5-ium products have a characteristic appearance on TLC, as demonstrated below by a typical example.
[Note 4]: Water jet vacuum pumps were used with gentle heating by a 30 °C water bath.
[Note 5]: This step promotes the formation of alkenyl thianthren-5-ium salts from the precursor dicationic species.
[Note 6]: In some specific cases, this color change was observed before washing with saturated aqueous sodium bicarbonate solution, however, this did not affect the outcome of the reaction.
[Note 7]: This step completes the counterion exchange. Completeness can be verified by ^{19}F-NMR.
[Note 8]: Isopropanol was preferred as the use of less hindered alcohols (i.e., methanol) as eluents may promote decomposition of the product.
[Note 9]: Drying under high vacuum is often required to completely get rid of solvent residues. We consider this to be particularly important as these residues might be reactive under basic conditions (i.e., isopropanol) and promote undesired reaction pathways in the upcoming step.

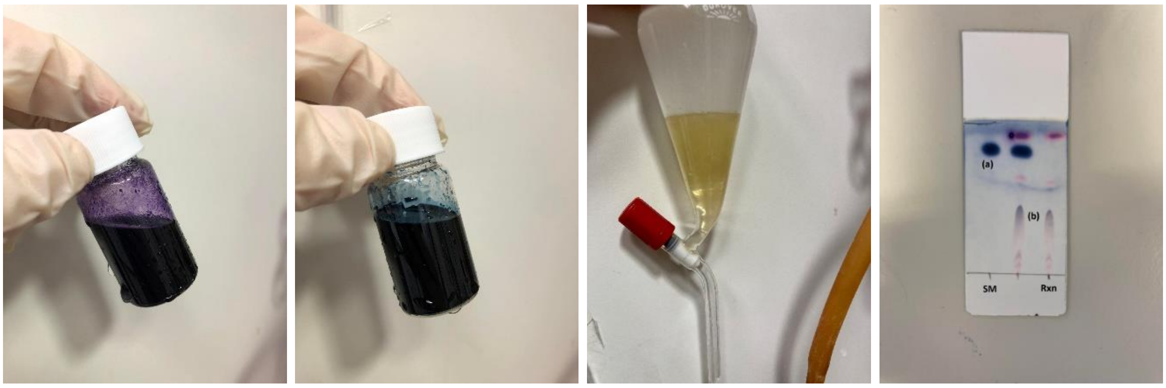

(A-B) Representative lilac/blue color of reaction mixtures; **(C)** yellow color after vigorous stirring with sat. aq. sodium bicarbonate solution; **(D)** a representative TLC of the crude mixture, stained with CAM: (a) alkene; (b) thianthren-5-ium salt

Compounds prepared by the General procedure as above:

Compound 2a (5-(6-(1,3-dioxoisoindolin-2-yl)hex-1-en-1-yl)-5*H*-thianthren-5-ium tetrafluoroborate)

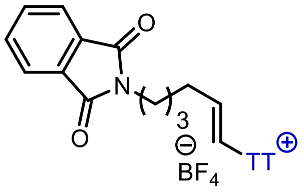

Following **General procedure** with the following modifications: carried out on a 10.0 mmol scale; 1 h reaction time at 25 °C. Purification by flash column chromatography on flash silica gel (0% isopropanol in dichloromethane grading to 10% isopropanol in dichloromethane) afforded 3.10 g (58%, *E*:*Z* = 97:3) of the title compound **2a**.

Physical State: white solid.

^{1}**H-NMR** (499.64 MHz, DMSO-d^6): δ = 8.24 (d, J = 7.9 Hz, 2H), 8.04 (d, J = 7.9 Hz, 2H), 7.92 – 7.80 (m, 6H), 7.75 (t, J = 7.7 Hz, 2H), 6.82 – 6.74 (m, 2H), 3.52 (t, J = 6.9 Hz, 2H), 2.29 (q, J = 6.7 Hz, 2H), 1.51 (p, J = 7.3 Hz, 2H), 1.38 ppm (p, J = 7.5 Hz, 2H). (see Spectrum)

^{13}**C-NMR** (125.65 MHz, DMSO-d^6): δ = 167.9, 153.8, 134.43, 134.35, 134.1, 133.4, 131.6, 130.0, 129.6, 123.0, 121.0, 111.4, 36.9, 31.6, 27.3, 24.4 ppm. (see Spectrum)

^{19}**F-NMR** (282.21 MHz, DMSO-d^6): δ = -90.55 (s), -90.60 ppm (s).

HRMS (ESI): M^+ calcd. for $[C_{26}H_{22}NO_2S_2]^+$ 444.1068, found 444.1068.

TLC: Rf = 0.50 (10% isopropanol in dichloromethane, CAM).

Compound 2b (5-(octadec-1-en-1-yl)-5*H*-thianthren-5-ium tetrafluoroborate)

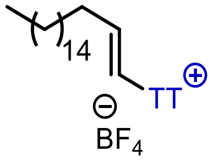

Following **General procedure** with the following modifications: carried out on a 4.5 mmol scale; 1 h reaction time at 25 °C. Purification by flash column chromatography on flash silica gel (0% isopropanol in dichloromethane grading to 10% isopropanol in dichloromethane) afforded 1.51 g (61%, *E*:*Z* = 93:7) of the title compound **2b**.

Physical State: brown amorphous solid.

^{1}**H-NMR** (499.64 MHz, DMSO-d^6): δ = 8.26 (dd, J = 7.9, 1.4 Hz, 2H), 8.06 (dd, J = 7.9, 1.3 Hz, 2H), 7.85 (td, J = 7.7, 1.4 Hz, 2H), 7.77 (td, J = 7.7, 1.3 Hz, 2H), 6.90 – 6.80 (m, 1H), 6.77 (d, J = 14.8 Hz, 1H), 2.25 (q, J = 7.0 Hz, 2H), 1.34 (q, J = 7.1 Hz, 2H), 1.28-1.17 (m, 26H), 0.85 ppm (t, J = 6.8 Hz, 3H). (see Spectrum)

^{13}**C-NMR** (125.65 MHz, DMSO-*d*^{6}): δ = 154.3, 134.3, 134.1, 133.3, 129.9, 129.6, 121.0, 111.1, 32.0, 31.2, 28.97, 28.96 (2C), 28.95, 28.93 (2C), 28.9, 28.8, 28.6, 28.5, 28.2, 26.9, 22.0, 13.9 ppm. (see Spectrum)

^{19}**F-NMR** (282.21 MHz, DMSO-*d*^{6}): δ = -90.55 (s), -90.60 ppm (s)

HRMS (ESI): M^{+} calcd. for $[C_{30}H_{43}S_2]^+$ 467.2801, found 467.2805.

TLC: Rf = 0.60 (10% isopropanol in dichloromethane, CAM).

Compound 2g (5-(3-(3-phenylpropoxy)prop-1-en-1-yl)-5*H*-thianthren-5-ium tetrafluoroborate)

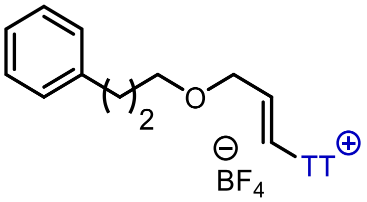

Following **General procedure** with the following modifications: 1 h reaction time at 25 °C. Purification by flash column chromatography on flash silica gel (0% isopropanol in dichloromethane grading to 10% isopropanol in dichloromethane) afforded 1.01 g (67%, *E*:*Z* = 34:66) of the title compound **2g**.

Physical State: brown oil.

***E*-2g**

^{1}**H-NMR** (499.64 MHz, DMSO-*d*^{6}): δ = 8.30 (dd, *J* = 7.9, 1.4 Hz, 2H), 8.07 (dd, *J* = 8.0, 1.3 Hz, 2H), 7.87 (td, *J* = 7.7, 1.4 Hz, 2H), 7.78 (td, *J* = 7.7, 1.3 Hz, 2H), 7.32-7.29 (m, 2H), 7.25-7.19 (m, 3H), 6.74 (dt, *J* = 14.7, 3.3 Hz, 1H), 6.69 (dt, *J* = 14.6, 1.4 Hz, 1H), 4.11 (dd, *J* = 3.4, 1.5 Hz, 2H), 3.36 (t, *J* = 6.4 Hz, 2H), 2.54 (dd, *J* = 8.6, 6.7 Hz, 2H), 1.80 – 1.70 ppm (m, 2H). (see Spectrum)

^{13}**C-NMR** (125.65 MHz, DMSO-*d*^{6}): δ = 148.9, 141.5, 134.7, 134.3, 134.2, 130.1, 129.6, 128.3, 128.2, 125.8, 120.0, 110.8, 69.7, 68.5, 31.6, 30.6 ppm (see Spectrum)

***Z*-2g**

^{1}**H-NMR** (499.64 MHz, DMSO-*d*^{6}): δ = 8.18 (dd, *J* = 8.0, 1.3 Hz, 2H), 8.07 (d, *J* = 1.3 Hz, 2H), 7.83 (td, *J* = 7.7, 1.4 Hz, 2H), 7.74 (td, *J* = 7.7, 1.3 Hz, 2H), 7.25-7.19 (m, 3H), 7.16-7.11 (m, 2H), 6.97 (dt, *J* = 9.3, 3.9 Hz, 1H), 6.90 (dt, *J* = 9.3, 2.0 Hz, 1H), 4.37 (dd, *J* = 3.9, 2.0 Hz, 2H), 3.64 (t, *J* = 6.6 Hz, 2H), 2.69 (dd, *J* = 8.9, 6.7 Hz, 2H), 2.02 – 1.94 ppm (m, 2H). (see Spectrum)

^{13}**C-NMR** (125.65 MHz, DMSO-*d*^{6}): δ = 149.0, 141.5, 134.5, 133.8, 133.1, 130.4, 129.4, 128.4 (2C), 125.9, 123.2, 113.8, 70.4, 68.0, 31.6, 30.5 ppm (see Spectrum)

^{19}**F-NMR** (282.21 MHz, DMSO-*d*^{6}): δ = -90.55 (s), -90.60 ppm (s).

HRMS (ESI): M^+ calcd. for $[C_{24}H_{23}OS_2]^+$ 391.1185, found 391.1187.

TLC: Rf = 0.50 (10% isopropanol in dichloromethane, CAM).

Compound 2h (5-(7-(benzoyloxy)hept-1-en-1-yl)-5*H*-thianthren-5-ium tetrafluoroborate)

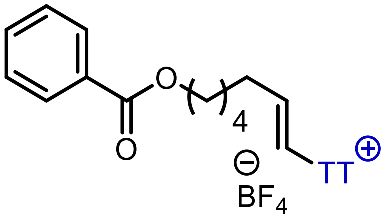

Following **General procedure** with the following modifications: carried out on a 1.25 mmol scale; 1 h reaction time at 25 °C. Purification by flash column chromatography on flash silica gel (0% isopropanol in dichloromethane grading to 10% isopropanol in dichloromethane) afforded 554 mg (84%, *E*:*Z* = 97:3) of the title compound **2h**.

Physical State: colorless oil.

^{1}H-NMR (499.64 MHz, DMSO-d^6): δ = 8.25 (dd, J =7.9, 1.3 Hz, 2H), 8.04 (dd, J = 7.9, 1.3 Hz, 2H), 7.94 (dd, J = 8.4, 1.3 Hz, 2H), 7.84 (td, J = 7.6, 1.4 Hz, 2H), 7.75 (td, J = 7.7, 1.3 Hz, 2H), 7.66 (t, J = 7.5 Hz, 1H), 7.53 (ddd, J = 8.8, 7.4, 1.5 Hz, 2H), 6.91-6.76 (m, 2H), 4.21 (t, J = 6.5 Hz, 2H), 2.30 (q, J = 7.0 Hz, 2H), 1.66 (p, J = 6.8 Hz, 2H), 1.44 (p, J = 7.3 Hz, 2H), 1.34 ppm (p, J = 7.0 Hz, 2H). (see Spectrum)

^{13}C-NMR (125.65 MHz, DMSO-d^6): δ = 165.7, 154.2, 134.3, 134.1, 133.3, 133.2, 130.0, 129.8, 129.6, 129.0, 128.7, 121.0, 111.2, 64.4, 32.0, 27.7, 26.6, 24.8 ppm. (see Spectrum)

^{19}F-NMR (282.21 MHz, DMSO-d^6): δ = -90.55 (s), -90.60 ppm (s).

HRMS (ESI): M^+ calcd. for $[C_{26}H_{25}O_2S_2]^+$ 433.1290, found 433.1293.

TLC: Rf = 0.45 (10% isopropanol in dichloromethane, CAM).

Compound 2i (5-(5-cyanopent-1-en-1-yl)-5*H*-thianthren-5-ium tetrafluoroborate)

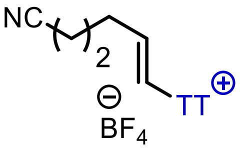

Following **General procedure** with the following modifications: 1.5 h reaction time at 25 °C. Purification by flash column chromatography on flash silica gel (0% isopropanol in dichloromethane grading to 10% isopropanol in dichloromethane) afforded 525 mg (42%, *E*:*Z* = 91:9) of the title compound **2i**.

Physical State: brown wax.

^{1}H-NMR (499.64 MHz, DMSO-d^{6}): δ = 8.26 (dd, J = 8.0, 1.4 Hz, 2H), 8.07 (dd, J = 8.0, 1.3 Hz, 2H), 7.86 (td, J = 7.7, 1.4 Hz, 2H), 7.77 (td, J = 7.7, 1.3 Hz, 2H), 6.86 – 6.77 (m, 2H), 2.46 (t, J = 7.2 Hz, 2H), 2.35 (td, J = 7.4, 5.3 Hz, 2H), 1.69 ppm (p, J = 7.3 Hz, 2H). (see Spectrum)

^{13}C-NMR (125.65 MHz, DMSO-d^{6}): δ = 152.3, 134.4, 134.1, 133.4, 130.0, 129.6, 120.8, 120.1, 112.2, 31.0, 22.8, 15.5 ppm. (see Spectrum)

^{19}F-NMR (282.21 MHz, DMSO-d^{6}): δ = -90.55 (s), -90.60 ppm (s).

HRMS (ESI): M^{+} calcd. for $[C_{18}H_{16}NS_{2}]^{+}$ 310.0719, found 310.0721.

TLC: Rf = 0.50 (10% isopropanol in dichloromethane, CAM).

Compound 2m (5-(octa-1,7-dien-1-yl)-5*H*-thianthren-5-ium tetrafluoroborate)

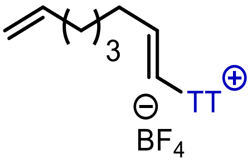

Following **General procedure** with the following modifications: 1 h reaction time at 25 °C. Purification by flash column chromatography on flash silica gel (0% isopropanol in dichloromethane grading to 10% isopropanol in dichloromethane) afforded 760 mg (59%, *E*:*Z* = 92:8) of the title compound **2m**.

Physical State: brown oil.

^{1}H-NMR (499.64 MHz, DMSO-d^{6}): δ = 8.26 (dd, J = 7.9, 1.4 Hz, 2H), 8.06 (dd, J = 7.9, 1.4 Hz, 2H), 7.85 (td, J = 7.7, 1.5 Hz, 2H), 7.77 (td, J = 7.7, 1.3 Hz, 2H), 6.90 – 6.77 (m, 2H), 5.76 – 5.68 (m, 1H), 5.02 – 4.90 (m, 2H), 2.27 (q, J = 6.9 Hz, 2H), 1.95 (q, J = 7.0 Hz, 2H), 1.36 (p, J = 7.3 Hz, 2H), 1.26 ppm (p, J = 7.5 Hz, 2H). (see Spectrum)

^{13}C-NMR (125.65 MHz, DMSO-d^{6}): δ = 154.2, 138.3, 134.3, 134.1, 133.3, 130.0, 129.6, 120.9, 114.8, 111.3, 32.5, 31.8, 27.3, 26.4 ppm. (see Spectrum)

^{19}F-NMR (282.21 MHz, DMSO-d^{6}): δ = -90.55 (s), -90.60 ppm (s).

HRMS (ESI): M^{+} calcd. for $[C_{20}H_{21}S_{2}]^{+}$ 325.1079, found 325.1083.

TLC: Rf = 0.60 (10% isopropanol in dichloromethane, CAM).

Compound 2n (5-(11-oxo-11-(prop-2-yn-1-yloxy)undec-1-en-1-yl)-5*H*-thianthren-5-ium tetrafluoroborate)

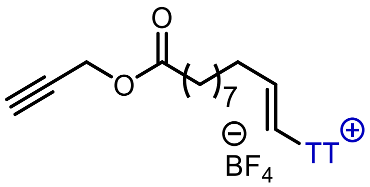

Following **General procedure** with the following modifications: 1.5 h reaction time at 25 °C. Purification by flash column chromatography on flash silica gel (0% isopropanol in dichloromethane grading to 10% isopropanol in dichloromethane) afforded 1.28 g (78%, *E*:*Z* = 96:4) of the title compound **2n**.

Physical State: brown oil.

^{1}H-NMR (499.64 MHz, DMSO-d^6): δ = 8.26 (dd, *J* = 8.0, 1.4 Hz, 2H), 8.06 (dd, *J* = 7.9, 1.3 Hz, 2H), 7.85 (td, *J* = 7.7, 1.4 Hz, 2H), 7.77 (td, *J* = 7.7, 1.3 Hz, 2H), 6.87 – 6.76 (m, 2H), 4.67 (d, *J* = 2.5 Hz, 2H), 3.52 (t, *J* = 2.4 Hz, 1H), 2.31 (t, *J* = 7.3 Hz, 2H), 2.25 (q, *J* = 7.0 Hz, 2H), 1.49 (p, *J* = 7.3 Hz, 2H), 1.35 (p, *J* = 7.2 Hz, 2H), 1.23 – 1.14 ppm (m, 8H). (see Spectrum)

^{13}C-NMR (125.65 MHz, DMSO-d^6): δ = 172.1, 154.3, 134.3, 134.1, 133.3, 129.9, 129.6, 120.9, 111.1, 78.5, 77.4, 51.4, 33.1, 32.0, 28.4, 28.3, 28.1 (2C), 26.9, 24.2 ppm. (see Spectrum)

^{19}F-NMR (282.21 MHz, DMSO-d^6): δ = -90.55 (s), -90.60 ppm (s).

HRMS (ESI): M^+ calcd. for $[C_{26}H_{29}O_2S_2]^+$ 437.1603, found 437.1611.

TLC: Rf = 0.55 (10% isopropanol in dichloromethane, CAM).

Compound 2o (5-(6-chlorohex-1-en-1-yl)-5*H*-thianthren-5-ium tetrafluoroborate)

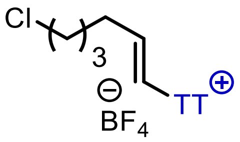

Following **General procedure** with the following modifications: 1 h reaction time at 25 °C. Purification by flash column chromatography on flash silica gel (0% isopropanol in dichloromethane grading to 10% isopropanol in dichloromethane) afforded 1.02 g (77%, *E*:*Z* = 94:6) of the title compound **2o**.

Physical State: brown oil.

^{1}H-NMR (499.64 MHz, DMSO-d^6): δ = 8.27 (dd, *J* = 8.0, 1.4 Hz, 2H), 8.07 (dd, *J* = 8.0, 1.3 Hz, 2H), 7.86 (td, *J* = 7.7, 1.4 Hz, 2H), 7.77 (td, *J* = 7.7, 1.3 Hz, 2H), 6.84 – 6.77 (m, 2H), 3.58 (t, *J* = 6.6 Hz, 2H), 2.29 (td, *J* = 7.3, 5.4 Hz, 2H), 1.64 (p, *J* = 6.9 Hz, 2H), 1.48 ppm (p, *J* = 7.1 Hz, 2H). (see Spectrum)

^{13}C-NMR (125.65 MHz, DMSO-d^6): 153.6, 134.4, 134.1, 133.4, 130.0, 129.6, 120.8, 111.6, 44.8, 31.2, 31.1, 24.3 ppm. (see Spectrum)

^{19}F-NMR (282.21 MHz, DMSO-*d*^{6}): δ = -90.55 (s), -90.60 ppm (s).

HRMS (ESI): M^+ calcd. for $[C_{18}H_{18}ClS_2]^+$ 333.0533, found 333.0535.

TLC: Rf = 0.50 (10% isopropanol in dichloromethane, CAM).

Compound 2q (5-(10-(tosyloxy)dec-1-en-1-yl)-5*H*-thianthren-5-ium tetrafluoroborate)

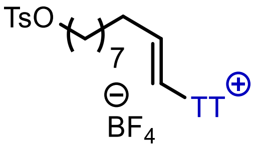

Following **General procedure** with the following modifications: carried out on a 4.5 mmol scale; 1 h reaction time at 25 °C. Purification by flash column chromatography on flash silica gel (0% isopropanol in dichloromethane grading to 10% isopropanol in dichloromethane) afforded 1.40 g (51%, *E*:*Z* = 98:2) of the title compound **2q**.

Physical State: colorless oil.

^{1}H-NMR (499.64 MHz, DMSO-*d*^{6}): δ = 8.27 (dd, *J* = 7.9, 1.3 Hz, 2H), 8.04 (dd, *J* = 7.9, 1.2 Hz, 2H), 7.84 (dt, *J* = 7.4, 1.0 Hz, 2H), 7.78-7.75 (m, 4H), 7.46 (d, *J* = 8.0 Hz, 2H), 6.85 (dt, *J* = 13.8, 6.8 Hz, 1H), 6.80 – 6.74 (m, 1H), 3.99 (t, *J* = 6.3 Hz, 2H), 2.40 (s, 3H), 2.24 (q, *J* = 7.2 Hz, 2H), 1.52 (p, *J* = 6.6 Hz, 2H), 1.32 (t, *J* = 7.1 Hz, 2H), 1.16 (t, *J* = 7.2 Hz, 2H), 1.13 – 1.07 ppm (m, 6H). (see Spectrum)

^{13}C-NMR (125.65 MHz, DMSO-*d*^{6}): δ = 154.3, 144.8, 134.3, 134.1, 133.3, 132.5, 130.1, 129.9, 129.6, 127.5, 120.9, 111.2, 70.8, 32.0, 28.2, 28.1, 28.0 (2C), 26.9, 24.6, 21.0 ppm. (see Spectrum)

^{19}F-NMR (282.21 MHz, DMSO-*d*^{6}): δ = -90.55 (s), -90.60 ppm (s).

HRMS (ESI): M^+ calcd. for $[C_{29}H_{33}O_3S_3]^+$ 525.1586, found 525.1588.

TLC: Rf = 0.60 (10% isopropanol in dichloromethane, CAM).

Compound 2v (5-(hex-3-en-3-yl)-5*H*-thianthren-5-ium tetrafluoroborate)

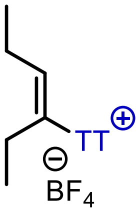

Following **General procedure** with the following modifications: starting from *cis*-hex-3-ene; 1 h reaction time at 25 °C. Purification by flash column chromatography on flash silica gel (0% isopropanol in dichloromethane grading to 10% isopropanol in dichloromethane) afforded 820 mg (67%, *E*:*Z* = 92:8) of the title compound **2v**.

Physical State: brown solid.

^{1}H-NMR (499.64 MHz, DMSO-*d*^{6}): δ = 8.42 (dd, *J* = 8.0, 1.3 Hz, 2H), 8.07 (dd, *J* = 8.0, 1.3 Hz, 2H), 7.91 (td, *J* = 7.7, 1.4 Hz, 2H), 7.81 (td, *J* = 7.7, 1.3 Hz, 2H), 5.51 (t, *J* = 7.7 Hz, 1H), 2.28 (q, *J* = 7.5 Hz, 2H), 2.16 (p, *J* = 7.5 Hz, 2H), 0.87 (t, *J* = 7.4 Hz, 3H), 0.72 ppm (t, *J* = 7.5 Hz, 3H). (see Spectrum)

^{13}C-NMR (125.65 MHz, DMSO-*d*^{6}): δ = 142.9, 135.5, 135.2, 134.7, 129.9, 129.7, 124.2, 116.7, 22.1, 21.0, 12.8, 12.7 ppm. (see Spectrum)

^{19}F-NMR (282.21 MHz, DMSO-*d*^{6}): δ = -90.55 (s), -90.60 ppm (s).

HRMS (ESI): M^+ calcd. for $[C_{18}H_{19}S_2]^+$ 299.0923, found 299.0924.

TLC: Rf = 0.55 (10% isopropanol in dichloromethane, CAM).

2.2. Specific procedures

Compound 2c (5-(but-1-en-1-yl)-5*H*-thianthren-5-ium tetrafluoroborate)

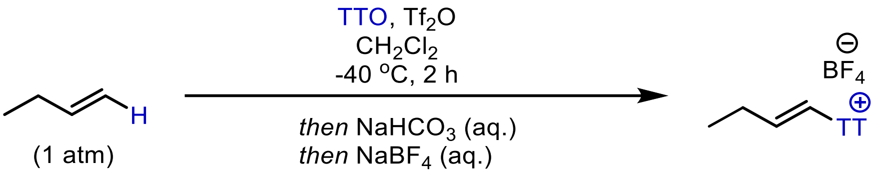

A round-bottom flask (100 mL) capped with a rubber septum and equipped with a stirring bar was charged with the solution of thianthrene S-oxide (TTO, 1.00 g, 4.30 mmol, 1 equiv) in anhydrous dichloromethane (35.0 mL, 0.125 M) and the mixture was cooled to -40 °C (internal temperature). Then, 1-butene gas was bubbled through the solution for 15 minutes, after which balloons filled with 1-butene were connected to the flask to maintain the 1-butene atmosphere throughout the reaction. Trifluoromethane sulfonic anhydride (873 µL, 5.17 mmol, 1.20 equiv) was added dropwise to the reaction, whereupon a dark purple suspension was progressively formed. After stirring at this temperature for further 30 min, the cooling bath was removed, and the mixture was stirred at 25 °C for 1.5 h until analysis by LC-MS indicated full conversion. At this point, the balloons and the rubber septum were removed, and saturated aqueous sodium bicarbonate solution (35 mL) was added carefully. The two-phase system was vigorously shaken in a separation funnel, phases were separated, and the aqueous layer was extracted with dichloromethane (2 × 20 mL). All organic phases were then combined and partially concentrated (to around 25 mL). Then, this organic phase was thoroughly washed with aqueous sodium tetrafluoroborate solution (5 wt.%, 5 × 30 mL) and dried over sodium sulfate. [Note 1] The dried solution was filtered, and the filtrate was concentrated under reduced pressure. The residue was purified by flash column chromatography on silica gel (5% isopropanol in dichloromethane grading to 10% isopropanol in dichloromethane) to yield alkenyl **2c** thiantren-5-ium tetrafluoroborate salt (1.04 g, 2.90 mmol, 68%, *E*:*Z* = 92:8) as a brown oil. [Notes 2,3]

[Note 1]: This step promotes counterion exchange; completeness may be practically verified by ^{19}F-NMR.
[Note 2]: Isopropanol was preferred as the use of less hindered alcohols (i.e., methanol) as eluents may promote decomposition of the product.
[Note 3]: Drying under high vacuum is often required to completely get rid of solvent residues. This is also important as these residues might be reactive under basic conditions (i.e., isopropanol) and promote undesired reaction pathways in the upcoming step.

Physical State: brown oil.

^{1}H-NMR (499.64 MHz, DMSO-d^6): δ = 8.26 (dd, J = 8.0, 1.4 Hz, 2H), 8.06 (dd, J = 7.9, 1.3 Hz, 2H), 7.85 (td, J = 7.7, 1.4 Hz, 2H), 7.77 (td, J = 7.7, 1.3 Hz, 2H), 6.97 – 6.86 (m, 1H), 6.76 (dt, J = 14.6, 1.6 Hz, 1H), 2.29 (tt, J = 7.4, 5.9 Hz, 2H), 0.97 ppm (t, J = 7.4 Hz, 3H). (see Spectrum)

^{13}C-NMR (125.65 MHz, DMSO-d^6): δ = 156.0, 134.3, 134.1, 133.3, 130.0, 129.6, 121.0, 110.3, 25.7, 11.6 ppm. (see Spectrum)

^{19}F-NMR (282.21 MHz, DMSO-d^6): δ = -90.55 (s), -90.60 ppm (s)

HRMS (ESI): M^+ calcd. for $[C_{16}H_{15}S_2]^+$ 271.0610, found 271.0611.

TLC: Rf = 0. 45 (10% isopropanol in dichloromethane, CAM).

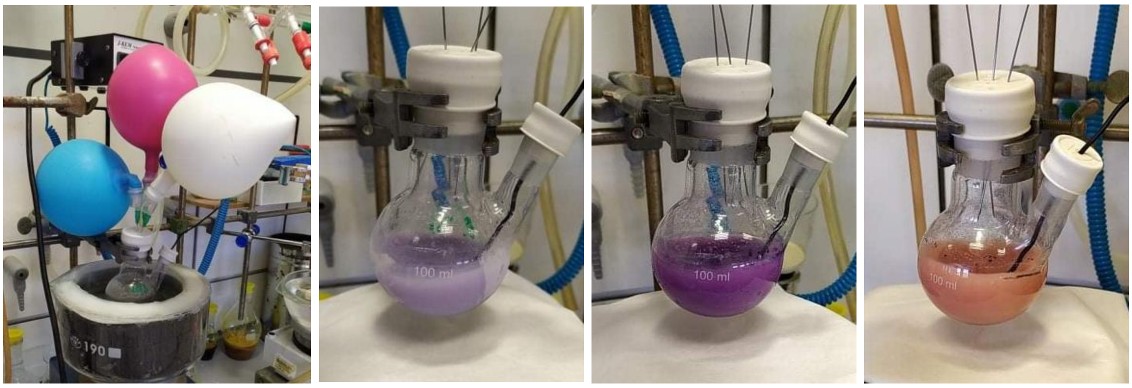

(A) Reaction setup; **(B-D)** color changes throughout the reaction

Compound 2d (5-(2-phenylprop-1-en-1-yl)-5*H*-thianthren-5-ium tetrafluoroborate) and 2d' (5-(2-phenylallyl)-5*H*-thianthren-5-ium tetrafluoroborate)

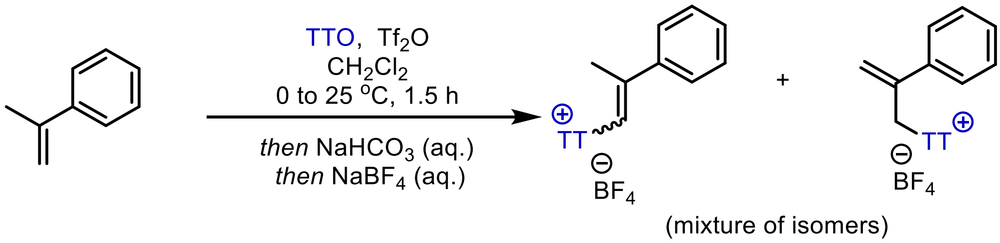

A solution of alkene (372 mg, 3.15 mmol, 1 equiv) and thianthrene S-oxide (TTO, 754 mg, 3.24 mmol, 1.03 equiv) in anhydrous dichloromethane (10 mL, 0.3 M) was cooled to 0 °C under argon atmosphere. [Note 1] At this temperature, trifluoromethane sulfonic anhydride (665 µL, 3.94 mmol, 1.25 equiv) was added dropwise. An immediate color change of the reaction mixture was observed upon addition and the blue solution was stirred at 0 °C for further 30 min. Then, the reaction mixture was warmed to 25 °C and stirred at this temperature for an additional 1 h until analysis by TLC indicated full conversion. At this point, saturated aqueous sodium bicarbonate solution (50 mL) was added, and the two-phase mixture was vigorously stirred at 25 °C for 15 min, whereupon a color change to brown was observed and the phases were separated. Then, aqueous sodium tetrafluoroborate solution (5 wt.%, 50 mL) was added to the organic phase and the mixture was stirred vigorously for 15 min and the phases were separated. This washing step using aqueous sodium tetrafluoroborate was repeated one more time. The layers were separated, and the organic phase was dried over sodium sulfate. The dried solution was filtered, and the filtrate was concentrated under reduced pressure. The residue was triturated with diethyl ether and dried under reduced pressure to yield the mixture of alkenyl and allylic thiantren-5-ium tetrafluoroborate salts **2d** and **2d'** (1.06 g, 80%, ratio 54:46). [Notes 2, 3]

[Note 1]: Dichloromethane was stabilized with amylene (not ethanol).

[Note 2]: Isopropanol readily reacts with the allylic isomer during column chromatography. [Note 3]: As both isomers are productive starting materials of the TT-Kornblum reaction, they were used as a mixture in the upcoming step.

Physical State: brown solid

2d

^{1}H-NMR (499.64 MHz, $CDCl_3$): δ = 8.45 (dd, J = 7.9, 1.3 Hz, 2H), 7.85 (dd, J = 7.9, 1.3 Hz, 2H), 7.71 (td, J = 7.7, 1.4 Hz, 2H), 7.65 (td, J = 7.7, 1.4 Hz, 2H), 7.41 – 7.36 (m, 1H), 7.34 (d, J = 4.1 Hz, 4H), 6.76 (q, J = 1.0 Hz, 1H), 2.78 ppm (d, J = 1.1 Hz, 3H). (see Spectrum)

^{13}C-NMR (125.65 MHz, $CDCl_3$): δ = 161.4, 137.7, 135.6, 134.2, 133.7, 131.1, 130.3 (2C), 129.0, 126.5, 121.6, 105.8, 19.4 ppm (see Spectrum)

2d'

^{1}H-NMR (499.64 MHz, $CDCl_3$): δ = 7.95 (dd, J = 7.9, 1.3 Hz, 2H), 7.71 (ddd, J = 7.8, 4.4, 1.7 Hz, 2H), 7.68 – 7.63 (m, 2H), 7.53 (td, J = 7.7, 1.4 Hz, 2H), 7.31 (td, J = 6.8, 3.1 Hz, 3H), 7.27 (dd, J = 6.9, 2.5 Hz, 2H), 5.58 (d, J = 11.6 Hz, 2H), 5.00 ppm (s, 2H). (see Spectrum)

^{13}C-NMR (125.65 MHz, $CDCl_3$): δ = 135.9, 135.0, 134.4, 129.7, 129.6, 129.2, 129.0, 128.7, 127.7, 126.2, 124.7, 116.6, 45.4 ppm (see Spectrum)

^{19}F-NMR (282.21 MHz, DMSO-d^6): δ = -90.55 (s), -90.60 ppm (s).

HRMS (ESI): M^+ calcd. for $[C_{21}H_{17}S_2]^+$ 333.0766, found 333.0770.

Compound 2j (5-(10-carboxydec-1-en-1-yl)-5*H*-thianthren-5-ium tetrafluoroborate)

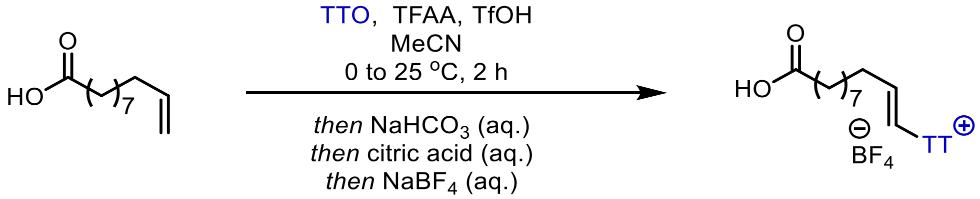

A suspension of alkene (580 mg, 3.15 mmol, 1 equiv) and thianthrene S-oxide (TTO, 754 mg, 3.24 mmol, 1.03 equiv) in anhydrous acetonitrile (10 mL, 0.3 M) was cooled to 0 °C under argon atmosphere. At this temperature, trifluoroacetic anhydride (TFAA, 1.33 mL, 9.45 mmol, 3.00 equiv) and trifluoromethanesulfonic acid (560 µL, 6.30 mmol, 2.00 equiv) were added dropwise consecutively. A delayed color change of the reaction mixture was observed upon addition and the blue solution was stirred at 0 °C for a further 1 h. [Note 1] Then, the reaction mixture was warmed to 25 °C and stirred at this temperature for an additional 1 h until analysis by TLC indicated full conversion. At this point, the reaction mixture was concentrated under reduced pressure [Note 2] and subsequently dissolved in dichloromethane (30 mL). Saturated aqueous sodium bicarbonate solution (50 mL) was added, and the two-phase mixture was vigorously stirred at 25 °C for 15 min, whereupon a color change to brown was observed. Aqueous citric acid solution (10 wt.%, approx. 50 mL) was added until pH = 6 and the phases were separated. [Note 3] Then, aqueous sodium tetrafluoroborate solution (5 wt.%, 50 mL) was added to the organic phase and the mixture was stirred vigorously for 15 min and the phases were separated. This washing step was repeated one more time. The layers were separated, and the organic phase was dried over sodium sulfate. The dried solution was filtered, and the filtrate was concentrated under reduced pressure. The residue was triturated with diethyl ether and dried under reduced pressure to yield **2j** alkenyl thiantren-5-ium tetrafluoroborate salt (1.24 g, 81%) [Note 4]

[Note 1]: In the presence of functional groups which are likely to be acetylated by trifluoroacetic anhydride (i.e., carboxylic acids), the color change is not immediate but occurs during addition.
[Note 2] Water jet vacuum pumps were used with gentle heating by a 30 °C water bath.
[Note 3] This step is required to ensure the protonation of the carboxylic acid.
[Note 4] No column chromatography was performed due to the particularly high polarity and acidity of the product.

Physical State: brown oil.

^{1}H-NMR (499.64 MHz, DMSO-*d*^{6}): δ = 11.99 (br s, 1H), 8.26 (dd, *J* = 8.0, 1.4 Hz, 2H), 8.06 (dd, *J* = 7.9, 1.4 Hz, 2H), 7.85 (td, *J* = 7.7, 1.4 Hz, 2H), 7.77 (td, *J* = 7.7, 1.3 Hz, 2H), 6.89 – 6.76 (m, 2H), 2.51-2.46 (m, 1H), 2.25 (q, *J* = 7.0 Hz, 2H), 2.17 (t, *J* = 7.4 Hz, 1H), 1.48 (dt, *J* = 24.7, 7.2 Hz, 2H), 1.35 (t, *J* = 7.1 Hz, 2H), 1.26 – 1.13 ppm (m, 8H). (see Spectrum)

^{13}C-NMR (125.65 MHz, DMSO-*d*^{6}): δ = 174.5, 154.4, 134.4, 134.1, 133.4, 130.0, 129.6, 121.0, 111.2, 33.6, 32.1, 28.6, 28.5, 28.4, 28.2, 27.0, 24.5 ppm. (see Spectrum)

^{19}F-NMR (282.21 MHz, DMSO-*d*^{6}): δ = -90.55 (s), -90.60 ppm (s).

HRMS (ESI): M^+ calcd. for $[C_{23}H_{27}O_2S_2]^+$ 399.1447, found 399.1448

TLC: Rf = 0.35 (10% isopropanol in dichloromethane, CAM).

Compound 2k (5-(10-hydroxydec-1-en-1-yl)-5*H*-thianthren-5-ium tetrafluoroborate)

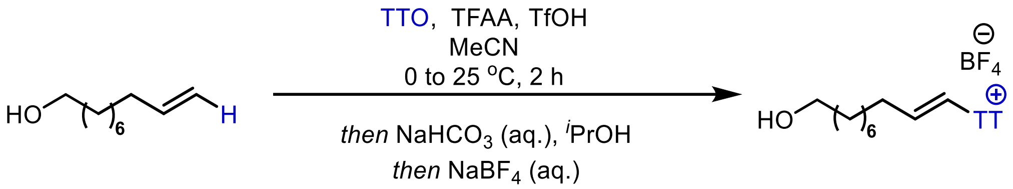

A suspension of alkene (492 mg, 3.15 mmol, 1 equiv) and thianthrene S-oxide (TTO, 754 mg, 3.24 mmol, 1.03 equiv) in anhydrous acetonitrile (10 mL, 0.3 M) was cooled to 0 °C under argon atmosphere. [Note 1] At this temperature, trifluoroacetic anhydride (1.78 mL, 12.6 mmol, 4.00 equiv) and trifluoromethanesulfonic acid (560 µL, 6.30 mmol, 2.00 equiv) were added dropwise consecutively. A delayed color change of the reaction mixture was observed upon addition and the lilac/blue solution was stirred at 0 °C for a further 1 h. [Note 2] Then, the reaction mixture was warmed to 25 °C and stirred at this temperature for an additional 1 h until analysis by TLC indicated full conversion. [Note 3] At this point, the reaction mixture was concentrated under reduced pressure [Note 4] and subsequently dissolved in isopropanol (20 mL). Saturated aqueous sodium bicarbonate solution (40 mL) was added, and the two-phase mixture was vigorously stirred at 25 °C for 150 min until analysis by TLC indicated full conversion. [Notes 5,6] Then, dichloromethane (40 mL) was added and the phases were separated. Aqueous sodium tetrafluoroborate solution (5 wt.%, 50 mL) was added to the organic phase and the mixture was stirred vigorously for 15 min and the phases were separated. This washing step was repeated one more time. [Note 7] The layers were separated, and the organic phase was dried over sodium sulfate. The dried solution was filtered, and the filtrate was concentrated under reduced pressure. The residue was purified by flash column chromatography on silica gel (0–10% isopropanol in dichloromethane) to yield **2k** alkenyl thiantren-5-ium tetrafluoroborate salt (1.05 g, 2.29 mmol, 73%) as a brown oil. [Notes 8,9]

[Note 1]: An ice bath was used.
[Note 2]: In the presence of functional groups which are likely to be acetylated by trifluoroacetic anhydride (i.e., alcohols), the color change is not immediate but occurs during addition.
[Note 3]: In this specific case, two thianthren-5-ium salts appeared on TLC: one with a trifluoroacetylated hydroxyl group and one with a free hydroxyl group.
[Note 4]: Water jet vacuum pumps were used with gentle heating by a 30 °C water bath.
[Note 5]: This step promotes the formation of alkenyl thianthren-5-ium salts from the precursor dicationic species and the hydrolyzation of the trifluoroacetyl esters.
[Note 6]: Full conversion leaves only one spot on the TLC out of the two characteristic spots of alkenyl thianthren-5-ium salts.
[Note 7]: This step completes the counterion exchange.
[Note 8]: Isopropanol was preferred as the use of less hindered alcohols (i.e., methanol) as eluents may promote decomposition of the product.
[Note 9]: Drying under high vacuum is often required to completely get rid of solvent residues. This is also important as these residues might be reactive under basic conditions (i.e., isopropanol) and promote undesired reaction pathways in the upcoming step.

Physical State: brown oil.

TLC: Rf = 0.55 (10% isopropanol in dichloromethane, CAM).

Spectral properties were in accordance with those reported in the literature.[7]

Compound 2l (5-(10-oxodec-1-en-1-yl)-5*H*-thianthren-5-ium tetrafluoroborate)

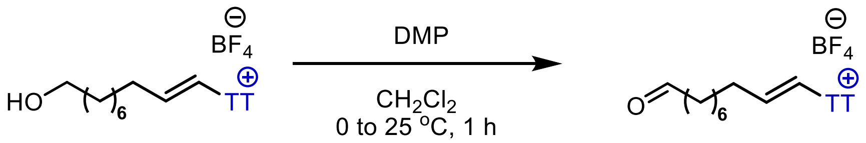

A solution of alcohol (756 mg, 1.65 mmol, 1 equiv) in dichloromethane (8.0 mL, 0.2 M) was cooled to 0 °C. [Note 1] To this solution was added Dess–Martin periodinane (DMP, 805 mg, 1.90 mmol, 1.15 equiv) in one portion. The mixture was allowed to warm to 25 °C and stirred at this temperature for 1 h when analysis by TLC indicated full conversion. At this point, saturated aqueous sodium bicarbonate solution (25 mL) and dichloromethane (20 mL) were added, and the phases were separated. Then, the organic layer was washed with aqueous sodium tetrafluoroborate solution (5 wt.%, 50 mL) and dried over sodium sulfate. [Note 2] The dried inhomogeneous solution was filtered through a plug of Celite and the clear filtrate was concentrated under reduced pressure. [Note 3] The residue was purified by flash column chromatography on silica gel (0–10% isopropanol in dichloromethane) to yield **2l** aldehyde (545 mg, 1.19 mmol, 72%, *E*:*Z* = 96:4) as a yellow amorphous solid.

[Note 1] Dichloromethane was stabilized with amylene (not ethanol).
[Note 2] This step ensures persistent counterion composition.
[Note 3] A white-colored byproduct precipitate derived from the DMP reagent was removed by the filtration.

Physical State: yellow amorphous solid.

^{1}H-NMR (499.64 MHz, DMSO-d^6): δ = 9.65 (t, J = 1.7 Hz, 1H), 8.26 (dd, J = 7.9, 1.4 Hz, 2H), 8.06 (dd, J = 7.9, 1.2 Hz, 2H), 7.85 (td, J = 7.7, 1.4 Hz, 2H), 7.77 (td, J = 7.7, 1.3 Hz, 2H), 6.89 – 6.72 (m, 2H), 2.39 (td, J = 7.2, 1.7 Hz, 2H), 2.26 (q, J = 7.0 Hz, 2H), 1.46 (p, J = 7.2 Hz, 2H), 1.35 (p, J = 7.2 Hz, 2H), 1.20-1.15 ppm (m, 6H). (see Spectrum)

^{13}C-NMR (125.65 MHz, DMSO-d^6): δ = 203.4, 154.3, 134.3, 134.1, 133.3, 129.9, 129.6, 120.9, 111.2, 42.9, 32.0, 28.3, 28.2, 27.9, 26.8, 21.3 ppm. (see Spectrum)

^{19}F-NMR (282.21 MHz, DMSO-d^6): δ = -90.55 (s), -90.60 ppm (s)

HRMS (ESI): M$^+$ calcd. for $[C_{22}H_{25}OS_2]^+$ 369.1341, found 369.1344.

TLC: Rf = 0.40 (10% isopropanol in dichloromethane, CAM).

[7] J. Chen, J. Li, M. B. Plutschack, F. Berger, T. Ritter, *Angew. Chem.* **2020**, *132*, 5665-5669.; *Angew. Chem. Int. Ed.* **2020**, *59*, 5616-5620.

3. General procedures for the preparation of unsaturated carbonyls

Method A (Ganem-like)

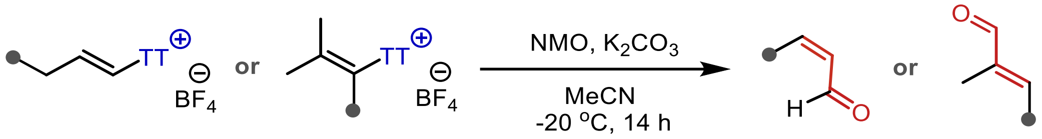

A suspension of *N*-methylmorpholine *N*-oxide (NMO, 187 mg, 1.60 mmol, 4.00 equiv) and well powdered, oven-dried potassium carbonate (276 mg, 2.00 mmol, 5.00 equiv) in anhydrous acetonitrile (3.0 mL) was cooled to -20 °C (±2 °C, internal temperature) and stirred at this temperature for 30 min. [Notes 1,2] Then, a solution of alkenyl thianthren-5-ium tetrafluoroborate (0.400 mmol, 1 equiv) in anhydrous acetonitrile (1.0 mL) was added dropwise while maintaining the internal temperature below -18 °C. [Note 3] The reaction mixture was stirred at this temperature for 14 h when analysis by TLC indicated full conversion. [Notes 4,5] Then, saturated aqueous sodium bicarbonate solution (30 mL) and dichloromethane (30 mL) were added subsequently. The layers were separated, and the organic phase was dried over sodium sulfate. [Note 6] The dried solution was filtered, and the filtrate was concentrated under reduced pressure. [Note 7] The residue was purified by flash column chromatography on silica gel (gradient elution using ethyl acetate and hexanes) to yield the unsaturated aldehydes. [Note 8]

Method B (Kornblum-like)

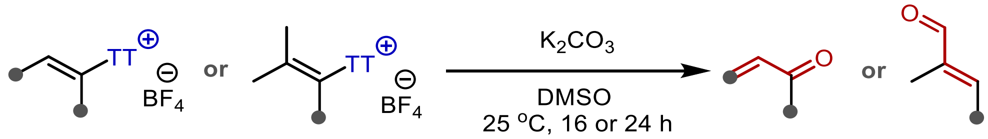

A suspension of well powdered, oven-dried potassium carbonate (276 mg, 2.00 mmol, 5.00 equiv) in anhydrous dimethyl sulfoxide (3.0 mL) was stirred at 25 °C for 5 min. Then, a solution of alkenyl thianthren-5-ium tetrafluoroborate (0.400 mmol, 1 equiv) in anhydrous dimethyl sulfoxide (1.0 mL) was added dropwise and the reaction mixture was stirred at this temperature for 14–24 h when analysis by TLC indicated full conversion. [Notes 3–5] Then, saturated aqueous sodium bicarbonate solution (30 mL) and diethyl ether (30 mL) were added subsequently. The layers were separated, and the aqueous phase was extracted with diethyl ether (2 × 30 mL). The combined organic layers were washed with brine (3 × 30 mL) and dried over sodium sulfate. The dried solution was filtered, and the filtrate was concentrated under reduced pressure. The residue was purified by flash column chromatography on silica gel (gradient elution using ethyl acetate and hexanes) to yield the unsaturated carbonyls. [Note 8]

[Note 1]: A low-temperature thermostat was used to control cooling bath temperature.
[Note 2]: This preliminary stirring ensures that the internal temperature stabilizes as initial overcooling to ca. -24 °C may occur due to the endothermic dissolution of K_2CO_3.
[Note 3]: Dissolution and addition was performed in two steps (0.75 mL + 0.25 mL wash) to secure complete transfer.

[Note 4]: The reaction mixture has a characteristic appearance on TLC, as demonstrated below by a typical example.
[Note 5]: At this point, NMR yield was measured as follows: trifluorotoluene or ethylene carbonate was added as an internal standard to the reaction mixture and a sample of it (0.1 mL) was diluted with deuterated chloroform (0.8 mL) and filtered. The yield and *Z*:*E* ratio (if relevant) were determined by ^{1}H NMR spectroscopy using solvent suppression.
[Note 6]: This step eliminates most of the excess NMO and *N*-alkylated by-products. In case of purification by column chromatography, no more washings are required, however, if using the crude mixture, additional washing with saturated aqueous sodium bicarbonate solution (2 × 30 mL) is recommended.
[Note 7]: To avoid isomerization of (*Z*)-aldehydes, the water bath temperature was set to 30 °C.
[Note 8]: Washing with pure hexanes until the by-product thianthrene elutes, then switching to gradient elution is a practical and effective procedure in most cases. Please see further details at specific substrates.

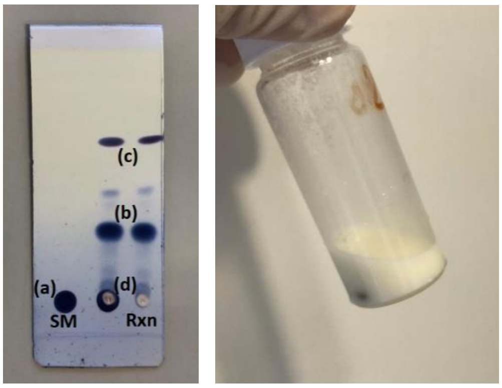

(A) A typical TLC during the reaction, directly spotted, stained with CAM; a) alkenyl thianthren-5-ium salt; b) unsaturated carbonyl product; c) thianthrene byproduct d) excess NMO **(B)** A typical reaction mixture upon completion

Compound 3a ((Z)-6-(1,3-dioxoisoindolin-2-yl)hex-2-enal)

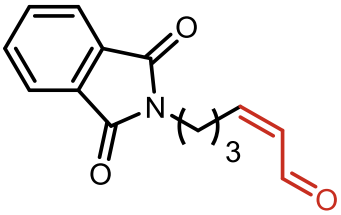

Starting from **2a** following **method A** without any modifications. NMR yield: 75% (*Z*:*E* = 83:17). Purification by flash column chromatography on flash silica gel (0% ethyl acetate in hexanes grading to 50% ethyl acetate in hexanes) afforded 70 mg (72%, *Z*:*E* = 71:29) of the title compound **3a**.

Physical State: colorless oil.

***Z*-3a**

^{1}H-NMR (499.64 MHz, $CDCl_3$): δ = 10.04 (d, *J* = 7.8 Hz, 1H), 7.84 (dd, *J* = 5.4, 3.1 Hz, 2H), 7.72 (dd, *J* = 5.5, 3.0 Hz, 2H), 6.59 (dt, *J* = 11.2, 8.0 Hz, 1H), 5.96 (ddt, *J* = 11.2, 7.9, 1.6 Hz, 1H), 3.75 (q, *J* = 7.0 Hz, 2H), 2.68 (qd, *J* = 7.8, 1.6 Hz, 2H), 1.92 ppm (p, *J* = 7.3 Hz, 2H). (see Spectrum)

^{13}C-NMR (125.65 MHz, $CDCl_3$): δ = 190.4, 168.2, 150.9, 134.0, 131.9, 130.6, 123.2, 37.1, 28.1, 25.4 ppm. (see Spectrum)

***E*-3a**

^{1}H-NMR (499.64 MHz, $CDCl_3$): δ = 9.47 (d, *J* = 7.8 Hz, 1H), 7.84 (dd, *J* = 5.4, 3.1 Hz, 2H), 7.72 (dd, *J* = 5.5, 3.0 Hz, 2H), 6.82 (dt, *J* = 15.6, 6.7 Hz, 1H), 5.96 (ddd, *J* = 15.7, 7.7, 1.5 Hz, 1H), 3.75 (q, *J* = 7.0 Hz, 2H), 2.40 (q, *J* = 7.7 Hz, 2H), 1.92 ppm (p, *J* = 7.3 Hz, 2H). (see Spectrum)

^{13}C-NMR (125.65 MHz, $CDCl_3$): δ = 193.5, 168.2, 156.4, 133.4, 131.9, 130.6, 123.2, 37.1, 29.9, 26.7 ppm. (see Spectrum)

HRMS (ESI): $[M+H]^+$ calcd. for $[C_{14}H_{14}NO_3]^+$ 244.0968, found 244.0967.

TLC: Rf = 0.50 (50% ethyl acetate in hexanes, CAM).

Compound 3b ((Z)-octadec-2-enal)

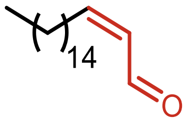

Starting from **2b** following **method A** without any modifications. NMR yield: 70% (*Z*:*E* = 84:16). Purification by flash column chromatography on flash silica gel (0% ethyl acetate in hexanes grading to 20% ethyl acetate in hexanes) afforded 71 mg (67%, *Z*:*E* = 79:21) of the title compound **3b**.

Physical State: colorless oil.

***Z*-3b**

^{1}H-NMR (499.64 MHz, $CDCl_3$): δ = 10.07 (d, *J* = 8.1 Hz, 1H), 6.62 (dt, *J* = 11.1, 8.2 Hz, 1H), 5.95 (ddt, *J* = 11.1, 8.1, 1.5 Hz, 1H), 2.60 (qd, *J* = 7.6, 1.5 Hz, 2H), 1.50 (p, *J* = 7.2 Hz, 2H), 1.39 – 1.17 (m, 24H), 0.87 ppm (t, *J* = 6.9 Hz, 3H). (see Spectrum)

^{13}C-NMR (125.65 MHz, $CDCl_3$): δ = 190.8, 153.3, 130.2, 31.9, 29.67, 29.66, 29.64, 29.63, 29.62, 29.58, 29.5, 29.33, 29.32, 29.2, 29.1, 28.0, 22.7, 14.1 ppm. (see Spectrum)

***E*-3b**

^{1}H-NMR (499.64 MHz, $CDCl_3$): δ = 9.50 (d, *J* = 7.9 Hz, 1H), 6.84 (dt, *J* = 15.5, 6.8 Hz, 1H), 6.11 (ddt, *J* = 15.6, 7.9, 1.5 Hz, 1H), 2.37 – 2.27 (m, 2H), 1.50 (p, *J* = 7.2 Hz, 2H), 1.41 – 1.18 (m, 24H), 0.87 ppm (t, *J* = 6.9 Hz, 3H). (see Spectrum)

^{13}**C-NMR** (125.65 MHz, $CDCl_3$): δ = 194.0, 158.9, 133.0, 32.7, 29(10 C overlapped with major component) 29.1, 27.8, 22.7, 14.1 ppm. (see Spectrum)

HRMS (ESI): $[M+H]^+$ calcd. for $[C_{18}H_{35}O]^+$ 267.2682, found 267.2685.

TLC: Rf = 0.50 (10% ethyl acetate in hexanes, CAM).

Compound 3c ((Z)-pent-2-enal)

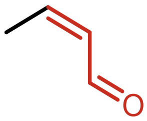

Starting from **2c** following **method A** without any modifications. NMR yield: 52% (*Z*:*E* = 88:12). No isolated yield was determined due to the volatility of the product. Spectral properties were in accordance with those reported in the literature.[8]

Compound 3d (2-phenylacrylaldehyde)

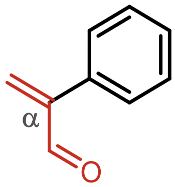

Starting from the 1:1 mixture of allyl and vinyl isomers (**2d** and **2d'**) following **method A** with the following modifications: reaction temperature at 0 °C; reaction time: 7 h. NMR yield: 58%. Purification by flash column chromatography on flash silica gel (0% ethyl acetate in hexanes grading to 20% ethyl acetate in hexanes) afforded 27 mg (51%) of the title compound **3d**.

Physical State: yellow oil.

TLC: Rf = 0.40 (10% ethyl acetate in hexanes, CAM).

Spectral properties were in accordance with those reported in the literature.[9]

[8] K. E. O'Shea, C. S. Foote, *J. Am. Chem. Soc.* **1988**, *110*, 7167–7170.
[9] Q. Zhu, Y. Luo, Y. Guo, Y. Zhang, Y. Tao, *J. Org. Chem.* **2021**, *86*, 5463–5476.

Compound 3e (2-cyclohexylideneacetaldehyde)

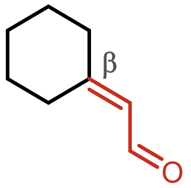

Starting from **2e** following **method A** with the following modifications: reaction temperature at 0 °C. NMR yield: 77%. Purification by flash column chromatography on flash silica gel (0% ethyl acetate in hexanes grading to 20% ethyl acetate in hexanes) afforded 32 mg (64%) of the title compound **3e**.

Physical State: colorless oil.

TLC: Rf = 0.30 (10% ethyl acetate in hexanes, CAM).

Spectral properties were in accordance with those reported in the literature.[10]

Compound 3f (cinnamaldehyde)

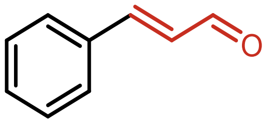

Starting from **2f** following **method A** without any modifications. NMR yield: 66% (*Z*:*E* = 8:92). Purification by flash column chromatography on flash silica gel (0% ethyl acetate in hexanes grading to 25% ethyl acetate in hexanes) afforded 34 mg (64%, *E* isomer) of the title compound **3f**.

Physical State: yellow oil.

TLC: Rf = 0.50 (25% ethyl acetate in hexanes, CAM).

Spectral properties were in accordance with those reported in the literature.[11]

Compound 3g ((Z)-3-(3-phenylpropoxy)acrylaldehyde)

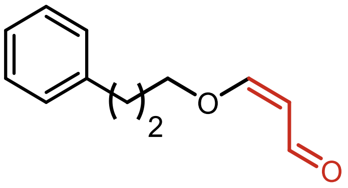

---

[10] B. Zhou, Q. Wu, Z. Dong, J. Xu, Z. Yang, *Org. Lett.* **2019**, *21*, 3594–3599.
[11] M. Stephan, J. Panther, F. Wilbert, P. Ozog, T. J. J. Müller, *European J. Org. Chem.* **2020**, *2020*, 2086–2092.

Starting from the mixture of regioisomeric thianthren-5-ium salts (**2g**) following **method A** without any modifications. NMR yield: 50% (*Z*:*E* = 44:56). Unfortunately, it was not possible to isolate the title compound in its pure form due to enhanced instability. Purification by flash column chromatography on flash silica gel (0% ethyl acetate in hexanes grading to 20% ethyl acetate in hexanes) afforded the title compound alongside with 3-phenylpropan-1-ol. Spectral properties were determined from this mixture.

Physical State: colorless oil.

***Z*-3g**

^{1}H-NMR (499.64 MHz, $CDCl_3$): δ = 10.06 (d, *J* = 8.4 Hz, 1H), 7.33 – 7.27 (m, 2H), 7.24 – 7.16 (m, 3H), 6.93 (d, *J* = 6.3 Hz, 1H), 5.11 (dd, *J* = 8.3, 6.3 Hz, 1H), 4.02 (t, *J* = 6.4 Hz, 2H), 2.78 – 2.74 (m, 2H), 2.07 ppm (qt, *J* = 7.3, 6.4 Hz, 2H). (see Spectrum)

^{13}C-NMR (125.65 MHz, $CDCl_3$): δ = 189.2, 163.4, 140.5, 128.6, 128.4, 126.3, 108.7, 74.6, 31.6, 31.0 ppm. (see Spectrum)

***E*-3g**

^{1}H-NMR (499.64 MHz, $CDCl_3$): δ = 9.37 (d, *J* = 7.9 Hz, 1H), 7.37 (d, *J* = 12.8 Hz, 1H), 7.33 – 7.27 (m, 2H), 7.24 – 7.16 (m, 3H), 5.59 (dd, *J* = 12.7, 7.9 Hz, 1H), 3.93 (t, *J* = 6.3 Hz, 2H), 2.78 – 2.74 (m, 2H), 2.07 ppm (qt, *J* = 7.3, 6.4 Hz, 2H). (see Spectrum)

^{13}C-NMR (125.65 MHz, $CDCl_3$): δ = 191.2, 170.4, 140.5, 128.6, 128.4, 126.3, 110.2, 70.7, 31.7, 30.2 ppm. (see Spectrum)

HRMS (ESI): $[M+H]^+$ calcd. for $[C_{12}H_{15}O]^+$ 191.1067, found 191.1067.

TLC: Rf = 0.35 (33% ethyl acetate in hexanes, CAM).

Compound 3h ((Z)-7-oxohept-5-en-1-yl benzoate)

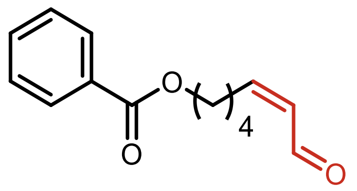

Starting from **2h** following **method A** without any modifications. NMR yield: 65% (*Z*:*E* = 83:17). Purification by flash column chromatography on flash silica gel (0% ethyl acetate in hexanes grading to 25% ethyl acetate in hexanes) afforded 56 mg (60%, *Z*:*E* = 78:22) of the title compound **3h**.

Physical State: colorless oil.

***Z*-3h**

^{1}H-NMR (499.64 MHz, $CDCl_3$): δ = 10.08 (d, *J* = 7.9 Hz, 1H), 8.03 (d, *J* = 7.1 Hz, 2H), 7.55 (d, *J* = 7.3 Hz, 1H), 7.44 (t, *J* = 7.7 Hz, 2H), 6.61 (dt, *J* = 11.2, 8.1 Hz, 1H), 5.99 (ddt, *J* = 11.2, 7.9, 1.6 Hz, 1H), 4.36 (t, *J* = 6.5 Hz, 2H), 2.70 (qd, *J* = 7.6, 1.6 Hz, 2H), 1.84 (dq, *J* = 15.2, 6.6 Hz, 2H), 1.69 ppm (tt, *J* = 9.9, 6.5 Hz, 2H). (see Spectrum)

^{13}C-NMR (125.65 MHz, $CDCl_3$): δ = 190.6, 166.5, 152.1, 133.3, 132.9, 130.5, 129.5, 128.4, 64.3, 28.3, 27.6, 25.8 ppm. (see Spectrum)

***E*-3h**

^{1}H-NMR (499.64 MHz, $CDCl_3$): δ = 9.51 (d, *J* = 7.8 Hz, 1H), 8.03 (dd, *J* = 8.3, 1.4 Hz, 2H), 7.55 (d, *J* = 7.3 Hz, 1H), 7.44 (t, *J* = 7.7 Hz, 2H), 6.85 (dt, *J* = 15.6, 6.7 Hz, 1H), 6.15 (ddt, *J* = 15.7, 7.8, 1.5 Hz, 1H), 4.35 (t, *J* = 6.5 Hz, 2H), 2.42 (qd, *J* = 7.4, 1.4 Hz, 2H), 1.84 (dq, *J* = 15.2, 6.6 Hz, 2H), 1.69 ppm (tt, *J* = 9.9, 6.5 Hz, 2H). (see Spectrum)

^{13}C-NMR (125.65 MHz, $CDCl_3$): δ = 193.8, 166.5, 157.5, 133.3, 132.9, 130.2, 129.5, 128.4, 64.3, 32.2, 28.3, 24.4 ppm. (see Spectrum)

HRMS (ESI): $[M+H]^+$ calcd. for $[C_{14}H_{17}O_3]^+$ 233.1172, found 233.1173.

TLC: Rf = 0.50 (25% ethyl acetate in hexanes, CAM).

Compound 3i ((Z)-6-oxohex-4-enenitrile)

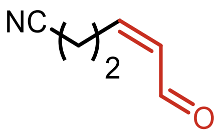

Starting from **2i** following **method A** without any modifications. NMR yield: 55% (*Z*:*E* = 52:48). Purification by flash column chromatography on flash silica gel (0% ethyl acetate in hexanes grading to 50% ethyl acetate in hexanes) afforded 22 mg (50%, *Z*:*E* = 43:57) of the title compound **3i**.

Physical State: colorless oil.

***Z*-3i**

^{1}H-NMR (499.64 MHz, $CDCl_3$): δ = 10.01 (d, *J* = 6.7 Hz, 1H), 6.51 (dt, *J* = 11.1, 7.9 Hz, 1H), 6.15 (ddt, *J* = 11.2, 6.7, 1.6 Hz, 1H), 2.96 (qd, *J* = 7.2, 1,5 Hz, 2H), 2.61 – 2.53 ppm (m, 2H). (see Spectrum)

^{13}C-NMR (125.65 MHz, $CDCl_3$): δ = 189.8, 145.6, 131.7, 118.0, 24.0, 17.0 ppm. (see Spectrum)

***E*-3i**

^{1}H-NMR (499.64 MHz, $CDCl_3$): δ = 9.55 (d, *J* = 7.6 Hz, 1H), 6.80 (dt, *J* = 15.8, 6.5 Hz, 1H), 6.21 (ddt, *J* = 15.8, 7.5, 1.6 Hz, 1H), 2.70 (qd, *J* = 7.1, 1.4 Hz, 2H), 2.60 – 2.54 ppm (m, 2H). (see Spectrum)

^{13}C-NMR (125.65 MHz, $CDCl_3$): δ = 192.8, 151.3, 134.5, 118.0, 28.0, 16.0 ppm. (see Spectrum)

HRMS (ESI): $[M+H]^+$ calcd. for $[C_6H_8NO]^+$ 110.0600, found 110.0601.

TLC: Rf = 0.35 (50% ethyl acetate in hexanes, CAM).

Compound 3j ((Z)-11-oxoundec-9-enoic acid)

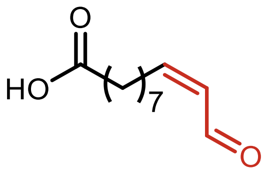

Starting from **2j** following **method A** with the following modifications: aqueous citric acid solution (2 wt.%, 50 mL) was used instead of saturated aqueous sodium bicarbonate solution during the work-up. NMR yield was determined after this modified aqueous work-up from the crude mixture. NMR yield: 46% (*Z*:*E* = 86:14). Purification by flash column chromatography on flash silica gel (isocratic elution using 10% ethyl acetate and 5% acetic acid in hexanes) afforded 29 mg (37%, *Z*:*E* = 83:17) of the title compound **3j**.

Physical State: colorless oil.

***Z*-3j**

^{1}H-NMR (499.64 MHz, $CDCl_3$): δ = 10.54 (br s, 1H), 10.06 (d, *J* = 8.1 Hz, 1H), 6.63 (dt, *J* = 11.2, 8.2 Hz, 1H), 5.96 (ddt, *J* = 11.2, 8.1, 1.5 Hz, 1H), 2.75 – 2.51 (m, 2H), 2.39 – 2.26 (m, 2H), 1.63 (p, *J* = 7.2 Hz, 2H), 1.50 (p, *J* = 7.3 Hz, 2H), 1.38-1.30 ppm (m, 6H). (see Spectrum)

^{13}C-NMR (125.65 MHz, $CDCl_3$): δ = 191.0, 179.8, 153.5, 130.1, 33.9, 29.0, 28.9, 28.8 (2C), 27.9, 24.5 ppm. (see Spectrum)

***E*-3j**

^{1}H-NMR (499.64 MHz, $CDCl_3$): δ = 10.54 (br s, 1H), 9.49 (d, *J* = 7.9 Hz, 1H), 6.85 (dt, *J* = 15.6, 6.8 Hz, 1H), 6.11 (ddt, *J* = 15.6, 7.9, 1.5 Hz, 1H), 2.75 – 2.51 (m, 2H), 2.39 – 2.26 (m, 2H), 1.63 (p, *J* = 7.2 Hz, 2H), 1.50 (p, *J* = 7.3 Hz, 2H), 1.38-1.30 ppm (m, 6H). (see Spectrum)

^{13}C-NMR (125.65 MHz, $CDCl_3$): δ = 194.3, 179.8, 159.0, 132.9, 32.6, 29.0, 28.9, 28.8 (2C), 27.7, 24.5 ppm. (see Spectrum)

HRMS (ESI): $[M+H]^+$ calcd. for $[C_{11}H_{19}O_3]^+$ 199.1329, found 199.1330.

TLC: Rf = 0.30 (10% ethyl acetate and 5% acetic acid in hexanes, CAM).

Compound 3k ((Z)-10-hydroxydec-2-enal)

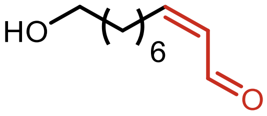

Starting from **2k** following **method A** without any modifications. NMR yield: 71% (*Z*:*E* = 87:13). Purification by flash column chromatography on flash silica gel (0% ethyl acetate in hexanes grading to 50% ethyl acetate in hexanes) afforded 65 mg (65%, *Z*:*E* = 82:18) of the title compound **3k**.

Physical State: colorless oil.

***Z*-3k**

^{1}H-NMR (499.64 MHz, $CDCl_3$): δ = 10.06 (d, J = 8.1 Hz, 1H), 6.61 (dt, J = 11.2, 8.2 Hz, 1H), 6.01 – 5.86 (m, 1H), 3.63 (t, J = 6.6 Hz, 2H), 2.59 (qd, J = 7.7, 1.5 Hz, 2H), 1.96 (br s, 1H), 1.55 (t, J = 6.8 Hz, 2H), 1.51 (t, J = 7.1 Hz, 2H), 1.40 – 1.32 ppm (m, 6H). (see Spectrum)

^{13}C-NMR (125.65 MHz, $CDCl_3$): δ = 190.9, 153.3, 130.1, 62.8, 32.6, 29.1 (2C), 29.0, 27.9, 25.6 ppm. (see Spectrum)

***E*-3k**

^{1}H-NMR (499.64 MHz, $CDCl_3$): δ = 9.49 (d, J = 7.9 Hz, 1H), 6.83 (dt, J = 15.6, 6.8 Hz, 1H), 6.10 (ddt, J = 15.6, 7.8, 1.5 Hz, 1H), 3.63 (t, J = 6.6 Hz, 2H), 2.39 – 2.25 (m, 2H), 1.96 (br s, 1H), 1.55 (t, J = 7.0 Hz, 2H), 1.51 (dd, J = 11.6, 4.9 Hz, 2H), 1.38 – 1.31 ppm (m, 6H). (see Spectrum)

^{13}C-NMR (125.65 MHz, $CDCl_3$): δ = 194.1, 158.8, 133.0, 62.8, 32.6, 29.1 (2C), 29.0, 27.7, 25.6 ppm. (see Spectrum)

HRMS (ESI): $[M+H]^+$ calcd. for $[C_{10}H_{19}O_2]^+$ 171.1380, found 171.1378.

TLC: Rf = 0.35 (50% ethyl acetate in hexanes, CAM)

Compound 3l ((Z)-dec-2-enedial)

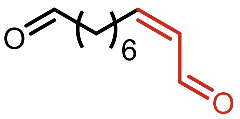

Starting from **2l** following **method A** without any modifications. NMR yield: 73% (*Z*:*E* = 84:16). Purification by flash column chromatography on flash silica gel (0% ethyl acetate in hexanes grading to 50% ethyl acetate in hexanes) afforded 42 mg (62%, *Z*:*E* = 79:21) of the title compound **3l**.

Physical State: colorless oil.

***Z*-3l**

^{1}H-NMR (499.64 MHz, $CDCl_3$): δ = 10.05 (d, J = 8.0 Hz, 1H), 9.74 (t, J = 1.7 Hz, 1H), 6.59 (dt, J = 11.2, 8.2 Hz, 1H), 5.94 (ddt, J = 11.2, 8.0, 1.5 Hz, 1H), 2.59 (qd, J = 7.6, 1.6 Hz, 2H), 2.41 (td, J = 7.3, 1.7 Hz, 2H), 1.62 (p, J = 7.3 Hz, 2H), 1.50 (p, J = 6.9 Hz, 2H), 1.39 – 1.32 ppm (m, 4H). (see Spectrum)

^{13}C-NMR (125.65 MHz, $CDCl_3$): δ = 202.4, 190.7, 152.9, 130.2, 43.7, 28.9, 28.8 (2C), 27.8, 21.8 ppm. (see Spectrum)

***E*-3l**

^{1}H-NMR (499.64 MHz, $CDCl_3$): δ = 9.74 (t, *J* = 1.7 Hz, 1H), 9.48 (d, *J* = 7.9 Hz, 1H), 6.82 (dt, *J* = 15.6, 6.8 Hz, 1H), 6.09 (ddt, *J* = 15.6, 7.8, 1.5 Hz, 1H), 2.41 (td, *J* = 7.3, 1.7 Hz, 2H), 2.32 (td, *J* = 7.4, 1.4 Hz, 2H), 1.62 (p, *J* = 7.3 Hz, 2H), 1.50 (q, *J* = 7.1 Hz, 2H), 1.39 – 1.32 ppm (m, 4H). (see Spectrum)

^{13}C-NMR (125.65 MHz, $CDCl_3$): δ = 202.4, 193.9, 158.4, 133.0, 43.7, 32.5, 28.8 (2C), 27.5, 21.8 ppm. (see Spectrum)

HRMS (ESI): $[M+H]^+$ calcd. for $[C_{10}H_{17}O_2]^+$ 169.1223, found 169.1221.

TLC: Rf = 0.40 (25% ethyl acetate in hexanes, CAM).

Compound 3m ((Z)-octa-2,7-dienal)

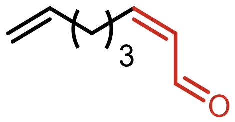

Starting from **2m** following **method A** without any modifications. NMR yield: 72% (*Z*:*E* = 81:19). Purification by flash column chromatography on flash silica gel (0% ethyl acetate in hexanes grading to 50% ethyl acetate in hexanes) afforded 34 mg (68%, *Z*:*E* = 79:21) of the title compound **3m**.

Physical State: colorless oil.

***Z*-3m**

^{1}H-NMR (499.64 MHz, $CDCl_3$): δ = 10.07 (d, *J* = 8.0 Hz, 1H), 6.61 (dt, *J* = 11.2, 8.2 Hz, 1H), 5.97 (ddt, *J* = 11.2, 8.1, 1.6 Hz, 1H), 5.79 (ddt, *J* = 17.0, 10.2, 6.7 Hz, 1H), 5.07 – 5.01 (m, 2H), 2.62 (qd, *J* = 7.8, 1.4 Hz, 2H), 2.13 (p, *J* = 7.4 Hz, 2H), 1.62 ppm (p, *J* = 7.5 Hz, 2H). (see Spectrum)

^{13}C-NMR (125.65 MHz, $CDCl_3$): δ = 190.8, 152.7, 137.6, 130.4, 115.5, 32.9, 28.2, 27.2 ppm. (see Spectrum)

***E*-3m**

^{1}H-NMR (499.64 MHz, $CDCl_3$): δ = 9.51 (d, *J* = 7.9 Hz, 1H), 6.84 (dt, *J* = 15.6, 6.8 Hz, 1H), 6.13 (ddt, *J* = 15.7, 7.9, 1.5 Hz, 1H), 5.79 (ddt, *J* = 17.0, 10.2, 6.7 Hz, 1H), 5.12 – 4.96 (m, 2H), 2.36 (dt, *J* = 7.4, 1.4 Hz, 2H), 2.13 (p, *J* = 7.4 Hz, 2H), 1.62 ppm (p, *J* = 7.5 Hz, 2H). (see Spectrum)

^{13}C-NMR (125.65 MHz, $CDCl_3$): δ = 193.9, 158.3, 133.2, 130.4, 115.5, 33.0, 32.0, 27.0 ppm. (see Spectrum)

HRMS (ESI): $[M+H]^+$ calcd. for $[C_8H_{13}O]^+$ 125.0961, found 125.0962.

TLC: Rf = 0.70 (25% ethyl acetate in hexanes, CAM).

Compound 3n (prop-2-yn-1-yl (Z)-11-oxoundec-9-enoate)

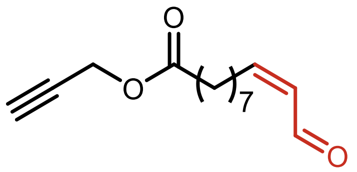

Starting from **2n** following **method A** without any modifications. NMR yield: 67% (*Z*:*E* = 85:15). Purification by flash column chromatography on flash silica gel (0% ethyl acetate in hexanes grading to 25% ethyl acetate in hexanes) afforded 60 mg (63%, *Z*:*E* = 78:22) of the title compound **3n**.

Physical State: colorless oil.

***Z*-3n**

^{1}H-NMR (499.64 MHz, $CDCl_3$): δ = 10.06 (d, *J* = 8.0 Hz, 1H), 6.60 (dt, *J* = 11.2, 8.2 Hz, 1H), 5.94 (ddt, *J* = 11.1, 8.0, 1.6 Hz, 1H), 4.66 (d, *J* = 2.5 Hz, 2H), 2.59 (qd, *J* = 7.6, 1.5 Hz, 2H), 2.45 (t, *J* = 2.5 Hz, 1H), 2.34 (t, *J* = 7.5 Hz, 2H), 1.63 (dq, *J* = 9.7, 7.2 Hz, 2H), 1.50 (p, *J* = 7.3 Hz, 2H), 1.36 – 1.30 ppm (m, 6H). (see Spectrum)

^{13}C-NMR (125.65 MHz, $CDCl_3$): δ = 190.8, 172.8, 153.1, 130.2, 77.8, 74.7, 51.7, 33.9, 29.1, 28.9, 28.8 (2C), 27.9, 24.7 ppm. (see Spectrum)

***E*-3n**

^{1}H-NMR (499.64 MHz, $CDCl_3$): δ = 9.49 (d, *J* = 7.9 Hz, 1H), 6.83 (dt, *J* = 15.5, 6.8 Hz, 1H), 6.10 (ddt, *J* = 15.6, 7.9, 1.5 Hz, 1H), 4.66 (d, *J* = 2.5 Hz, 2H), 2.45 (t, *J* = 2.5 Hz, 1H), 2.35 – 2.30 (m, *J* = 7.5 Hz, 4H), 1.63 (dq, *J* = 9.7, 7.2 Hz, 2H), 1.50 (p, *J* = 7.3 Hz, 2H), 1.36 – 1.30 ppm (m, 6H). (see Spectrum)

^{13}C-NMR (125.65 MHz, $CDCl_3$): δ = 194.0, 172.8, 158.6, 133.0, 77.8, 74.7, 51.7, 32.6, 29.1, 28.86, 28.85 (2C), 27.7, 24.7 ppm. (see Spectrum)

HRMS (ESI): $[M+H]^+$ calcd. for $[C_{14}H_{21}O_3]^+$ 237.1485, found 237.1486.

TLC: Rf = 0.45 (25% ethyl acetate in hexanes, CAM).

Compound 3o ((Z)-6-chlorohex-2-enal)

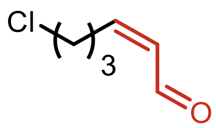

Starting from **2o** following **method A** without any modifications. NMR yield: 70% (*Z*:*E* = 82:18). Purification by flash column chromatography on flash silica gel (0% ethyl acetate in hexanes grading to 25% ethyl acetate in hexanes) afforded 27 mg (51%, *Z*:*E* = 67:33) of the title compound **3o**. Significant yield loss and isomer ratio change were experienced probably due to the enhanced and different volatility of the two isomers.

Physical State: colorless oil.

***Z*-3o**

^{1}H-NMR (499.64 MHz, $CDCl_3$): δ = 10.09 (d, J = 7.8 Hz, 1H), 6.56 (dt, J = 11.2, 8.1 Hz, 1H), 6.01 (ddt, J = 11.1, 7.9, 1.6 Hz, 1H), 3.57 (td, J = 6.4, 4.5 Hz, 2H), 2.79 (tdd, J = 8.1, 6.8, 1.6 Hz, 2H), 1.99 ppm (dddd, J = 12.8, 8.1, 6.4, 2.2 Hz, 2H). (see Spectrum)

^{13}C-NMR (125.65 MHz, $CDCl_3$): δ = 190.5, 150.3, 131.2, 43.7, 31.5, 25.1 ppm. (see Spectrum)

***E*-3o**

^{1}H-NMR (499.64 MHz, $CDCl_3$): δ = 9.52 (d, J = 7.8 Hz, 1H), 6.82 (dt, J = 15.6, 6.7 Hz, 1H), 6.15 (ddt, J = 15.6, 7.7, 1.6 Hz, 1H), 3.57 (td, J = 6.3, 4.5 Hz, 2H), 2.52 (dt, J = 7.2, 1.4 Hz, 2H), 1.99 (dddd, J = 12.8, 8.1, 6.4, 2.2 Hz, 2H). (see Spectrum)

^{13}C-NMR (125.65 MHz, $CDCl_3$): δ = 193.6, 156.0, 133.7, 43.8, 30.5, 29.7 ppm. (see Spectrum)

HRMS (ESI): $[M+H]^+$ calcd. for $[C_6H_{10}ClO]^+$ 133.0415, found 133.0414.

TLC: Rf = 0.40 (25% ethyl acetate in hexanes, CAM).

Compound 3p ((Z)-7-bromohept-2-enal)

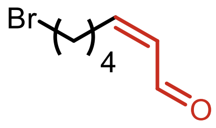

Starting from **2p** following **method A** without any modifications. NMR yield: 77% (*Z*:*E* = 88:12). Purification by flash column chromatography on flash silica gel (0% ethyl acetate in hexanes grading to 25% ethyl acetate in hexanes) afforded 54 mg (71%, *Z*:*E* = 84:16) of the title compound **3p**.

Physical State: colorless oil.

***Z*-3p**

^{1}H-NMR (499.64 MHz, $CDCl_3$): δ = 10.06 (d, J = 7.9 Hz, 1H), 6.59 (dt, J = 11.2, 8.1 Hz, 1H), 5.99 (ddd, J = 11.2, 7.8, 1.6 Hz, 1H), 3.42 (t, J = 6.6 Hz, 2H), 2.65 (qd, J = 7.7, 1.6 Hz, 2H), 1.92 (dt, J = 15.1, 6.8 Hz, 2H), 1.68 ppm (p, J = 7.5 Hz, 2H). (see Spectrum)

^{13}C-NMR (125.65 MHz, $CDCl_3$): δ = 190.5, 151.8, 130.5, 32.9, 31.9, 27.6, 27.1 ppm. (see Spectrum)

***E*-3p**

^{1}H-NMR (499.64 MHz, $CDCl_3$): δ = 9.51 (d, J = 7.8 Hz, 1H), 6.82 (dt, J = 15.6, 6.7 Hz, 1H), 6.13 (ddt, J = 15.6, 7.8, 1.5 Hz, 1H), 3.42 (t, J = 6.6 Hz, 2H), 2.38 (qd, J = 7.1, 1.5 Hz, 2H), 1.92 (dt, J = 15.1, 6.8 Hz, 2H), 1.68 ppm (p, J = 7.5 Hz, 2H). (see Spectrum)

^{13}C-NMR (125.65 MHz, $CDCl_3$): δ = 193.7, 157.3, 133.4, 32.0, 31.7, 27.6, 26.3 ppm. (see Spectrum)

HRMS (ESI): $[M+H]^+$ calcd. for $[C_7H_{12}BrO]^+$ 191.0066, found 191.0065.

TLC: Rf = 0.50 (25% ethyl acetate in hexanes, CAM).

Compound 3q ((Z)-10-oxodec-8-en-1-yl 4-methylbenzenesulfonate)

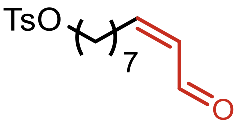

Starting from **2q** following **method A** without any modifications. NMR yield: 84% (*Z*:*E* = 81:19). Purification by flash column chromatography on flash silica gel (0% ethyl acetate in hexanes grading to 30% ethyl acetate in hexanes) afforded 100 mg (77%, *Z*:*E* = 79:21) of the title compound **3q**.

Physical State: colorless oil.

***Z*-3q**

^{1}H-NMR (499.64 MHz, $CDCl_3$): δ = 10.05 (d, *J* = 8.0 Hz, 1H), 7.77 (d, *J* = 8.3 Hz, 2H), 7.33 (d, *J* = 8.0 Hz, 2H), 6.59 (dt, *J* = 11.2, 8.2 Hz, 1H), 5.94 (ddt, *J* = 11.2, 8.0, 1.5 Hz, 1H), 4.01 (t, *J* = 6.5 Hz, 2H), 2.57 (qd, *J* = 7.6, 1.5 Hz, 2H), 2.44 (s, 3H), 1.63 (p, *J* = 7.3 Hz, 2H), 1.47 (p, *J* = 7.2 Hz, 2H), 1.33 – 1.22 ppm (m, 6H). (see Spectrum)

^{13}C-NMR (125.65 MHz, $CDCl_3$): δ = 190.8, 153.0, 144.6, 133.3, 130.2, 129.8, 127.8, 70.5, 29.0, 28.8, 28.7, 28.6, 27.9, 25.2, 21.6 ppm. (see Spectrum)

***E*-3q**

^{1}H-NMR (499.64 MHz, $CDCl_3$): δ = 9.49 (d, *J* = 7.9 Hz, 1H), 7.77 (d, *J* = 8.3 Hz, 2H), 7.33 (d, *J* = 8.0 Hz, 2H), 6.82 (dt, *J* = 15.6, 6.8 Hz, 1H), 6.09 (ddt, *J* = 15.6, 7.9, 1.5 Hz, 1H), 4.01 (t, *J* = 6.5 Hz, 2H), 2.44 (s, 3H), 2.36 – 2.26 (m, 2H), 1.63 (p, *J* = 7.3 Hz, 2H), 1.47 (p, *J* = 7.2 Hz, 2H), 1.33 – 1.22 ppm (m, 6H). (see Spectrum)

^{13}C-NMR (125.65 MHz, $CDCl_3$): δ = 193.9, 158.5, 144.6, 133.0, 130.2, 129.8, 127.8, 70.5, 32.5, 28.8, 28.7, 28.6, 27.6, 25.2, 21.6 ppm. (see Spectrum)

HRMS (ESI): $[M+H]^+$ calcd. for $[C_{17}H_{25}O_4S_3]^+$ 325.1468, found 325.1464.

TLC: Rf = 0.35 (25% ethyl acetate in hexanes, CAM).

Compound 5a (cyclopent-2-en-1-one)

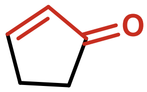

Starting from **2r** following **method B** without any modifications. NMR yield: 33%. No isolated yield was determined due to the volatility of the product. Spectral properties were in accordance with those reported in the literature.[12]

[12] J. Zhang, L. Wang, Q. Liu, Z. Yang, Y. Huang, *Chem. Commun.* **2013**, *49*, 11662–11664.

Compound 5b (cyclohex-2-en-1-one)

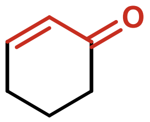

Starting from **2s** following **method B** without any modifications. NMR yield: 61%. No isolated yield was determined due to the volatility of the product. Spectral properties were in accordance with those reported in the literature.[12]

Compound 5c (cyclohept-2-en-1-one)

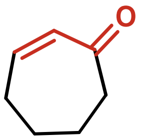

Starting from **2t** following **method B** without any modifications. NMR yield: 57%. No isolated yield was determined due to the volatility of the product. Spectral properties were in accordance with those reported in the literature.[13]

Compound 5d (cyclooct-2-en-1-one)

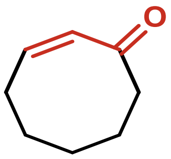

Starting from **2u** following **method B** without any modifications. NMR yield: 61%. No isolated yield was determined due to the volatility of the product. Spectral properties were in accordance with those reported in the literature.[13]

Compounds 5e ((E)-hex-3-en-2-one)and 5f ((E)-hex-4-en-3-one)

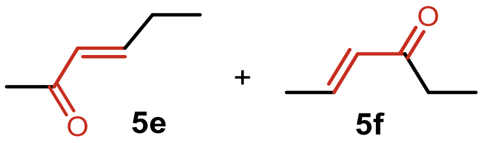

Starting from **2v** following **method B** without any modifications. NMR yield: 41% **5e** and 32% **5f**. No isolated yield was determined due to the volatility of the products. Spectral properties were in accordance with those reported in the literature.[14]

---

[13] L.-Q. Cui, K. Liu, C. Zhang, *Org. Biomol. Chem.* **2011**, *9*, 2258–2265.
[14] a) D. A. Oare, M. A. Henderson, M. A. Sanner, C. H. Heathcock, *J. Org. Chem.* **1990**, *55*, 132–157.
b) S. Nakatsu, A. T. Gubaidullin, V. A. Mamedov, S. Tsuboi, *Tetrahedron* **2004**, *60*, 2337–2349.

Compounds 5g ((E)-2-methyl-3-((2R,4S)-4-methyltetrahydro-2H-pyran-2-yl) acrylaldehyde) and 5h (2-methyl-1-((2R,4S)-4-methyltetrahydro-2*H*-pyran-2-yl)prop-2-en-1-one)

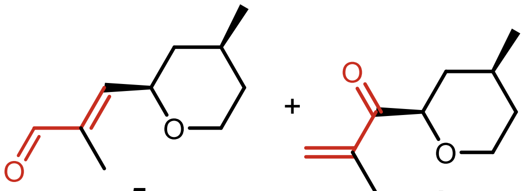

5g **5h**

Starting from **2w** following **method A** with the following modifications: reaction temperature at 25 °C. NMR yield: 3% **5h** and 69% **5g**. Purification by flash column chromatography on flash silica gel (0% ethyl acetate in hexanes grading to 15% ethyl acetate in hexanes) afforded 41 mg (61%) of the title compound **5g**.

Following **method B** without any modifications. NMR yield: 4% **5h** and 32% **5g**.

Unfortunately, it was not possible to isolate adequate amounts of the low yielding ketone **5h** on this scale. To afford a small amount of clean analytical sample, the reaction was also carried out and purified on a 1.2 mmol scale using **method B**.

5g aldehyde

Physical State: colorless oil.

^{1}H-NMR (499.64 MHz, $CDCl_3$): δ = 9.38 (s, 1H), 6.37 (dq, *J* = 7.4, 1.3 Hz, 1H), 4.26 (ddd, *J* = 11.4, 7.3, 2.3 Hz, 1H), 4.01 (ddd, *J* = 11.6, 4.6, 1.6 Hz, 1H), 3.47 (ddd, *J* = 12.4, 11.5, 2.2 Hz, 1H), 1.74 (d, *J* = 1.5 Hz, 3H), 1.68 (tdd, *J* = 10.2, 7.2, 5.1 Hz, 1H), 1.63 – 1.52 (m, 2H), 1.25 (dd, *J* = 12.6, 4.6 Hz, 1H), 1.08 (dt, *J* = 13.1, 11.6 Hz, 1H), 0.95 ppm (d, *J* = 6.6 Hz, 3H). (see Spectrum)

^{13}C-NMR (125.65 MHz, $CDCl_3$): δ = 195.0, 152.9, 138.1, 74.7, 67.8, 38.7, 34.0, 29.9, 22.1, 9.6 ppm. (see Spectrum)

HRMS (ESI): $[M+H]^+$ calcd. for $[C_{10}H_{17}O_2]^+$ 169.1223, found 169.1223.

TLC: Rf = 0.25 (10% ethyl acetate in hexanes, CAM).

5h ketone

Physical State: colorless oil.

^{1}H-NMR (499.64 MHz, $CDCl_3$): δ = 5.99 (q, *J* = 0.9 Hz, 1H), 5.83 (dt, *J* = 1.5, 0.7 Hz, 1H), 4.47 (dd, *J* = 11.7, 2.2 Hz, 1H), 4.14 (ddd, *J* = 11.4, 4.7, 1.5 Hz, 1H), 3.50 (ddd, *J* = 12.3, 11.5, 2.2 Hz, 1H), 1.90 (t, *J* = 1.2 Hz, 3H), 1.83 (ddd, *J* = 13.3, 4.0, 2.0 Hz, 1H), 1.71 (tdd, *J* = 9.3, 7.2, 4.5 Hz, 1H), 1.58 (dqd, *J* = 13.4, 3.9, 2.7 Hz, 1H), 1.31 (qd, *J* = 12.3, 4.7 Hz, 1H), 1.18 (dt, *J* = 13.0, 11.7 Hz, 1H), 0.97 (d, *J* = 6.5 Hz, 3H). (see Spectrum)

^{13}C-NMR (125.65 MHz, $CDCl_3$): δ = 199.6, 142.3, 125.4, 79.0, 68.3, 37.6, 34.0, 30.3, 22.2, 18.1 ppm. (see Spectrum)

HRMS (ESI): $[M+H]^+$ calcd. for $[C_{10}H_{17}O_2]^+$ 169.1223, found 169.1223.

TLC: Rf = 0.35 (10% ethyl acetate in hexanes, CAM).

4. Detailed optimization and practical guidance

See below for extended optimization data and a summary of useful knowledge that may be helpful in case of application to a new substrate:

a) Oxygen source

NMO turned out to be superior among *N*-oxides **[method A]**, however, DMSO is a possible alternative **[method B]**.

method A: general conditions

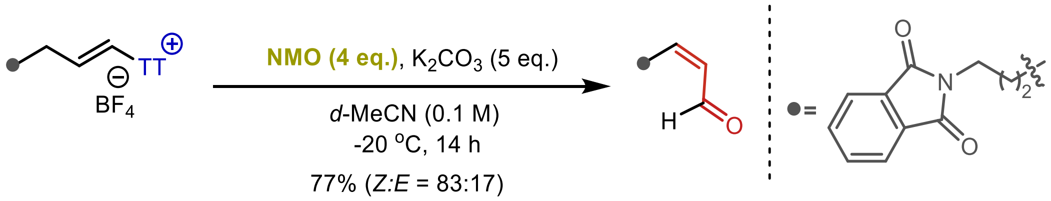

	deviation: oxygen source	effect	NMR yield (%) (*Z/E*)
1	DIPEANO	complex mixture	0 (n.d.)
2	PICNO	complex mixture	4 (n.d.)
3	TMANO	enhanced sideproduct formation	39 (83:17)
4	NMO (1.0 eq.)	enhanced sideproduct formation	59 (88:12)
5	DMSO, 25 °C **[method B]**	complex mixture	44 (68:32)
6	Thianthrene S-oxide	no reaction	0 (n.d.)

(DIPEANO = N,N-Diisopropylethylamine N-oxide; PICNO = 2-methylpyridine N-oxide; TMANO = trimethylamine N-oxide; NMO = N-methylmorpholine N-oxide, DMSO = dimethyl sulfoxide, n.d. = not determined)

b) Base

In the case of **method A** (Ganem-like, using NMO): A range of bases is suitable, as *N*-methylmorpholine (which is formed) autocatalyzes the reaction. Generally, K_2CO_3 is recommended to be used, however, $NaHCO_3$ might also be suitable if an even less basic catalyst is preferred due to isomerization issues or increased sensitivity. If a homogeneous base is needed, DIPEA would be a practical first choice (as it is volatile).

method A: general conditions

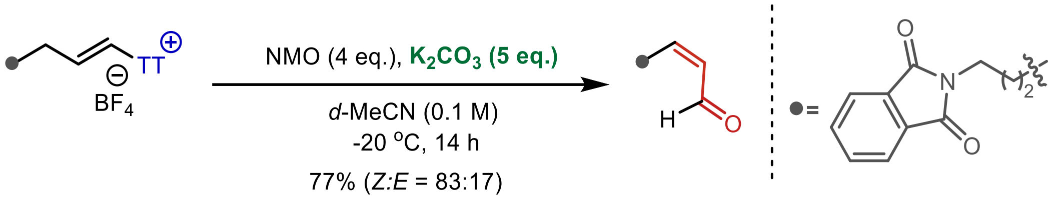

	deviation: base	effect	NMR yield (%) (*Z/E*)
1	DBU	alkylation of the base	0
2	2,6-Lutidine	moderatly enhanced sideproduct formation	74 (82:18)
3	TMP	moderatly enhanced sideproduct formation	70 (80:20)
4	DIPEA	moderatly enhanced sideproduct formation	69 (87:13)
5	$NaHCO_3$	not significant	76 (86:14)
6	K_3PO_4	not significant	63 (80:20)

(DBU = 1,8-diazabicyclo(5.4.0)undec-7-ene; TMP = 2,2,6,6-tetramethylpiperidine; DIPEA = N,N-diisopropylethylamine)

In the case of **method B** (Kornblum-like, using DMSO): The range of bases that can be used is much narrower, however, $NaHCO_3$ and DIPEA also promote the reaction (significantly slower), and these are recommended to be tested alongside K_2CO_3 at first.

method B: general conditions

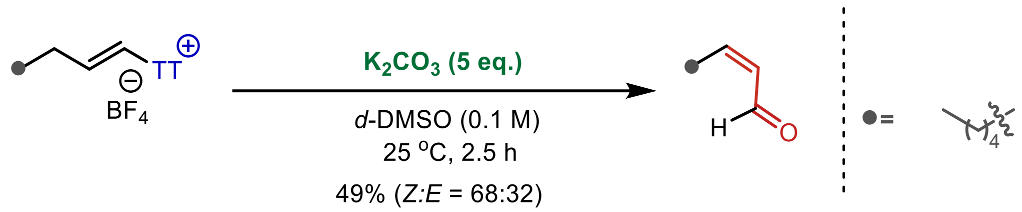

	Deviation: base	effect	NMR yield (%) (*Z*/*E*)
1	Cs_2CO_3	significant decomposition	0 (n.d.)
2	DBU	only sideproduct formation	0 (n.d.)
3	TMP	significant sideproduct formation	26 (70:30)
4	DIPEA, 16 h	slower conversion	46 (78:22)
5	$NaHCO_3$, 16 h	slower conversion	52 (70:30)
6	2,6-Lutidine	mostly intact	0 (n.d.)
7	*N*,*N*-dimethylaniline	mostly intact	traces (n.d.)

(DBU = 1,8-diazabicyclo(5.4.0)undec-7-ene; TMP = 2,2,6,6-tetramethylpiperidine; DIPEA = N,N-diisopropylethylamine)

c) Solvent (method A)

If only reactivity is considered, tetrahydrofuran and acetone might be good alternatives. However, acetonitrile has the advantage of not being miscible with hexanes. This allows washing the reaction mixture to remove the byproduct thianthrene if necessary (see details at “e) Work-up, isolation”).

method A: general conditions

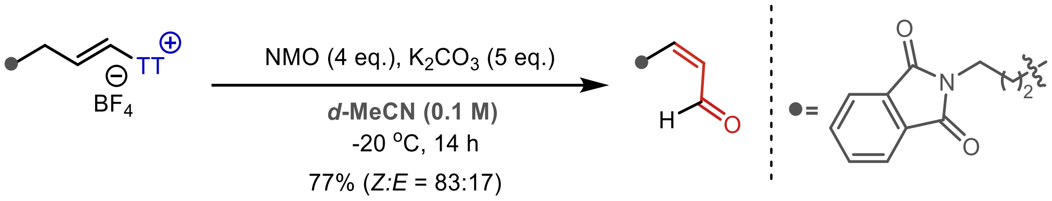

	deviation: solvent	effect	NMR yield (%) (*Z*/*E*)
1	*d*-DCM	enhanced sideproduct formation	53 (72:28)
2	*d*-THF	not significant	71 (81:21)
3	*d*-Acetone	not significant	75 (82:18)
4	*d*-Toluene	some dissolution issues	72 (76:24)

d) Reaction time, temperature, and monitoring of the reaction

Importantly, temperature and reaction time affect the *Z*:*E* ratio mostly. As is, optimization of reaction times in the case of a specific substrate might be important to avoid partial isomerization to (*E*)-enals due to unnecessarily long reaction times. In our procedures, we used general reaction times, which were adequate in most cases, as indicated by TLC analysis. However, LC-MS is also a suitable option to check conversion, as alkenyl thianthrenium salts are usually well detectable. In case of elevated temperatures, when selectivity swap toward (*E*)-enals was targeted, using the milder base sodium bicarbonate is recommended as potassium carbonate promotes significant decomposition of the product over time.

method A: general conditions

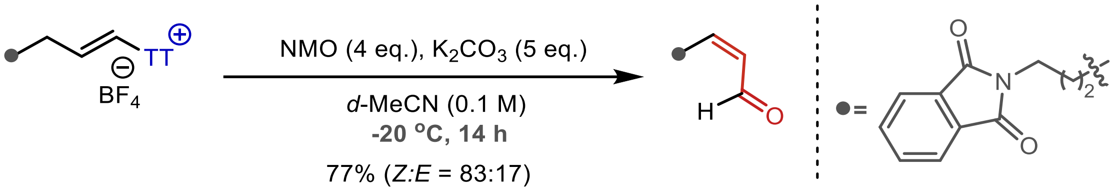

	deviation: temperature/time	effect	NMR yield (%) (*Z*/*E*)
1	0 °C, 4 h	enhanced *E* formation	77 (77:24)
2	25 °C, 90 min	enhanced *E* formation	73 (49:51)
3	25 °C, 14 h	isomerization	56 (2:98)
4	$NaHCO_3$, 50 °C, 14 h	optimal for *E* formation	79 (1:99)

e) Work-up, isolation

Polar substrates, which are not soluble in hexanes, can be isolated with good purity utilizing only extractions (see below for the modified work-up procedure). This modification results in minimal isomerization. Interestingly, it turned out that the substrate used for optimization was quite keen to isomerize, compared to other screened substrates. Therefore, this is an illustrative example.

method A: general conditions

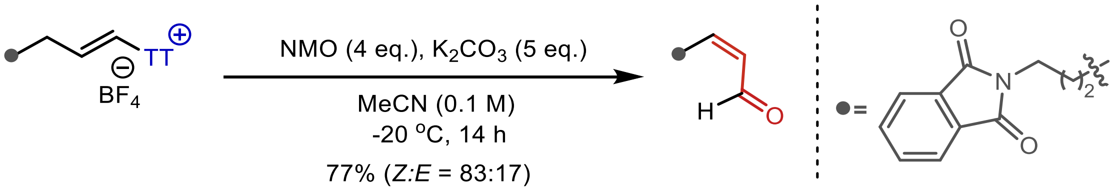

	deviation: isolation	effect	isolated yield (%) (*Z*/*E*)
1	column chromatography	some isomerization	72% (71:29)
2	extractions	no additional isomerization	70% (81:19) [95% NMR pur.]

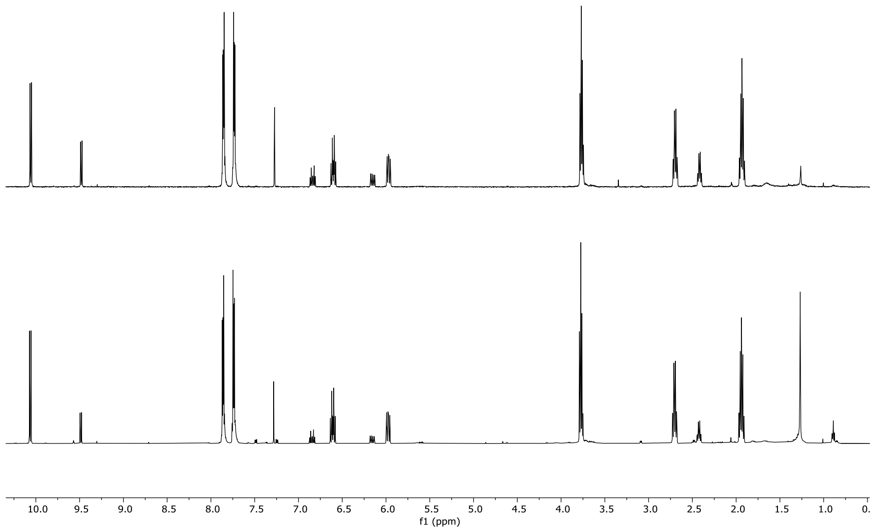

Representative ^{1}H-NMR spectra to compare purity in case of column chromatography **(up)** and extractions **(down)**

Modified work up of method A - extractions (0.4 mmol)

[…] When analysis by TLC indicated full conversion of the reaction, the mixture was filtered, and the filter cake was washed with acetonitrile (25 mL). The washing solution was recombined with the filtrate. The filtrate was washed with hexanes (5 × 60 mL) and then diluted with dichloromethane (120 mL). This combined solution was washed with saturated aqueous sodium bicarbonate (2 × 60 mL) and brine (60 mL) and dried over sodium sulfate. The dried solution was filtered, and the filtrate was concentrated under reduced pressure to yield the unsaturated aldehydes.

f) Robustness

We consider this reaction to be quite robust and not sensitive to water and air. We didn't have significant scale-up problems when scaling up to 10 mmol scale (see Applications down below). However, the heterogenicity of the reaction mixture should be kept in mind (i.e., the mixture should not adhere to the uncooled parts of the flask, as enhanced isomerization (*Z* to *E*) might occur; setting up the mixing and cooling should be precise).

method A: general conditions

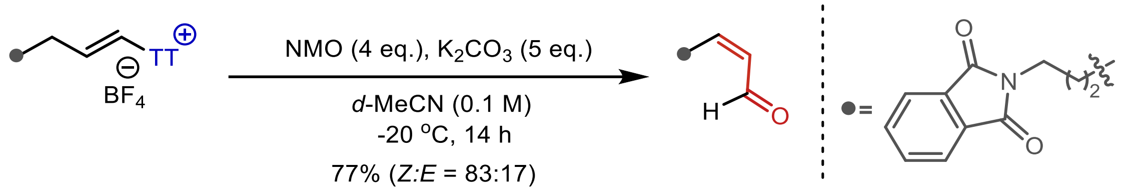

	deviation	effect	change in NMR yield (%)
1	water added (10 equiv)	not significant	<5%
2	open to air	not significant	<5%
3	triflate counterion	not significant	<5%
4	K_2CO_3 neither well powdered, nor oven dried	not significant	<5%

5. Investigation of the reactive intermediates

In a 5 mm NMR tube a 58:42 mixture (0.10 mmol, 1 equiv) of **2d** (Vinyl-TT) and **2d’** (Allyl-TT) was dissolved in CD_3CN (1.0 mL, 0.1 M), then NMO (4 equiv) and DIPEA (5 equiv) were added subsequently. We followed the conversion of our starting materials by carrying out an ^{1}H-NMR measurement every 90 seconds for 45 minutes at 30 °C. We also followed the formation of the product **3d** which is the result of the oxidation of **2d** and **2d’** as well. To quantify our results, we used the residual peak of CD_3CN as an internal standard.

Our results were in accordance with the proposed mechanism as we observed that the allyl species **2d’** reached full conversion within 4 minutes, whereas the vinyl-thianthrenium salt reached only 70% conversion under 45 minutes. This indicates that the allyl-thianthrenium species **2d’** is a highly reactive and productive intermediate of the TT-Kornblum/Ganem reaction.

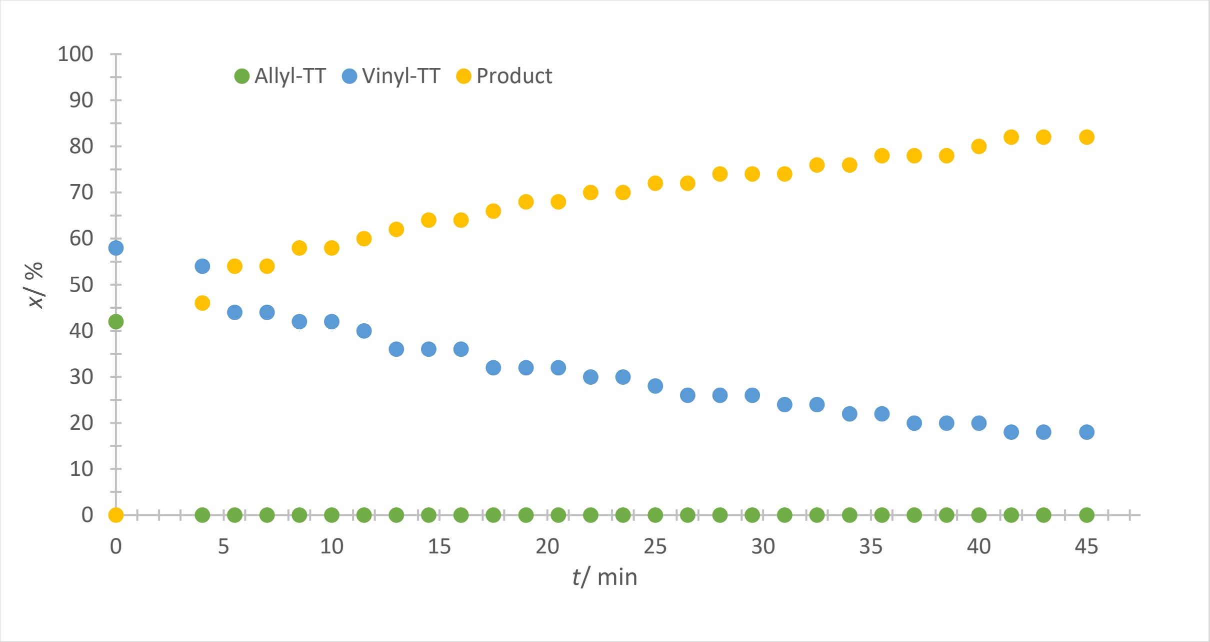

(x = The ratio of each species in the mixture, where *x*=100% is the sum of the *x*(TT-allyl) and *x*(TT-vinyl) in the t_0 state)

6. Applications and one-pot modifications

Application (A): Synthesis of Yue's intermediate towards Ivorenolide A

Upscaled, slightly modified versions of our general procedures for the preparation of alkenyl thianthren-5-ium salts and unsaturated carbonyls, and Yue's protocol[15] were used as follows:

Preparation of alcohol 6a

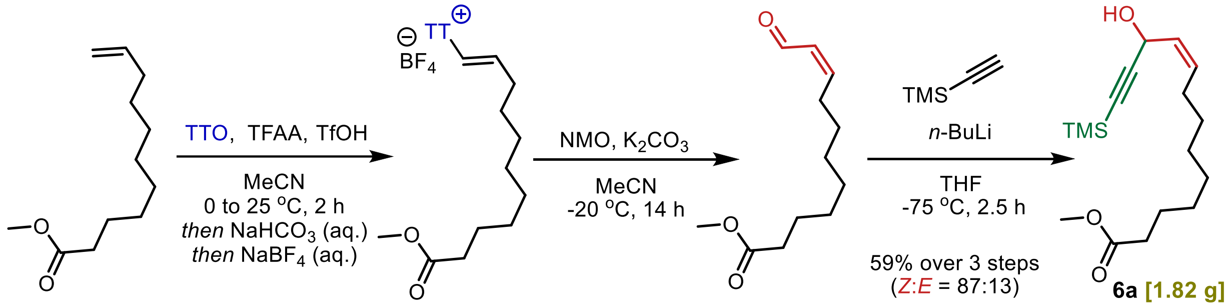

A suspension of methyl undec-10-enoate (1.98 g, 10.0 mmol, 1 equiv) and thianthrene S-oxide (TTO, 2.39 g, 10.3 mmol, 1.03 equiv) in anhydrous acetonitrile (30 mL, 0.3 M) was cooled to 0 °C (internal temperature) under argon atmosphere. Then, trifluoroacetic anhydride (TFAA, 4.24 mL, 40.0 mmol, 3.00 equiv) and trifluoromethanesulfonic acid (1.78 mL, 30.0 mmol, 2.00 equiv) were added consecutively dropwise, while maintaining the internal temperature below 10 °C. [Note 1] An immediate color change of the reaction mixture was observed upon the first addition step and the lilac solution was stirred at 0 °C for a further 1 h. Then, the reaction mixture was warmed to 25 °C and stirred at this temperature for an additional 1 h, when analysis by TLC indicated full conversion. At this point, the reaction mixture was concentrated under reduced pressure and subsequently dissolved in dichloromethane (100 mL). [Note 2] Saturated aqueous sodium bicarbonate solution (100 mL) was added, and the two-phase mixture was vigorously stirred at 25 °C for 15 min, whereupon a color change to brown was observed. [Note 3] The phases were separated and aqueous sodium tetrafluoroborate solution (5 wt.%, 150 mL) was added to the organic phase and the mixture was stirred vigorously for 10 min and the phases were separated. This washing step was repeated two more times. [Note 4] The layers were separated, and the organic phase was dried over sodium sulfate. The dried solution was filtered, and the filtrate was concentrated under reduced pressure. The crude alkenyl thianthren-5-ium tetrafluoroborate was used in the next step without further purification.

[Note 1]: Caution! Special attention should be taken when adding the corrosive trifluoromethanesulfonic acid dropwise at this scale as disposable syringes/needles may disintegrate.
[Note 2]: Water jet vacuum pumps were used with gentle heating by a 30 °C water bath.
[Note 3]: This step promotes the formation of alkenyl thianthrenium salts from the precursor dicationic species.
[Note 4]: This step completes the counterion exchange. Completeness may be practically verified by ^{19}F-NMR.

---

[15] B. Zhang, Y. Wang, S.-P. Yang, Y. Zhou, W.-B. Wu, W. Tang, J.-P. Zuo, Y. Li, J.-M. Yue, *J. Am. Chem. Soc.* **2012**, *134*, 20605-20608.

A suspension of *N*-methylmorpholine *N*-oxide (NMO, 4.69 g, 40 mmol, 4.00 equiv) and well powdered, oven-dried potassium carbonate (6.91 g, 50 mmol, 5.00 equiv) in anhydrous acetonitrile (75 mL) was cooled to -20 °C (±2 °C, internal temperature) and stirred at this temperature for 30 min. [Notes 5,6] Then, a solution of the crude alkenyl thianthren-5-ium tetrafluoroborate in anhydrous acetonitrile (25 mL) was added dropwise while maintaining the internal temperature below -18 °C. The reaction mixture was stirred at this temperature for 14 h when analysis by TLC indicated full conversion. Then, saturated aqueous sodium bicarbonate solution (400 mL) and dichloromethane (400 mL) were added subsequently. The layers were separated, and the organic phase was washed with saturated aqueous sodium bicarbonate solution (2 × 200 mL) and dried over sodium sulfate. [Note 7] The dried solution was filtered, and the filtrate was concentrated under reduced pressure. [Note 8] The crude enal was used in the next step without further purification.

[Note 5]: A low-temperature thermostat was used to control cooling bath temperature.
[Note 6]: This preliminary stirring ensures that the internal temperature stabilizes as initial overcooling to ca. -24 °C may occur due to the endothermic dissolution of K_2CO_3.
[Note 7]: These steps eliminate excess NMO and *N*-alkylated by-products.
[Note 8]: To avoid isomerization of the (*Z*)-enal, the water bath temperature was set to 30 °C.

A solution of ethynyltrimethylsilane (1.1 mL, 7.5 mmol, 0.75 equiv) in anhydrous tetrahydrofuran (46.7 mL) was cooled to -75 °C (±2 °C, internal temperature). Then, *n*-BuLi in hexanes (3.0 mL, 2.5 M, 7.5 mmol, 0.75 equiv) was added dropwise, while maintaining the internal temperature below -70 °C and the mixture was stirred for an additional 20 min. Then, a solution of the crude enal in anhydrous tetrahydrofuran (20 mL) was added dropwise while maintaining the internal temperature below -70 °C and the mixture was stirred for an additional 2.5 h. At this point, saturated aqueous ammonium chloride solution (60 mL) was added, and under stirring the two-phase system was allowed to warm to 25 °C. Then, ethyl acetate (150 mL) was added, and the phases were separated. The aqueous phase was extracted with ethyl acetate (2 × 100 mL). The combined organic phases were washed with brine (150 mL) and dried over sodium sulfate. The dried solution was filtered, and the filtrate was concentrated under reduced pressure. The residue was purified by flash column chromatography on silica gel (0–20% ethyl acetate in hexanes) to afford alcohol **6a** (1.82 g, 5.86 mmol, 59%, *Z*:*E* = 87:13) as a colorless oil.

^{1}H-NMR (499.64 MHz, $CDCl_3$): δ = 5.55-5.50 (m, 2H), 5.10 (dd, *J* = 6.8, 4.4 Hz, 1H), 3.64 (s, 3H), 2.28 (t, *J* = 7.6 Hz, 2H), 2.17 – 2.03 (m, 3H), 1.59 (t, *J* = 7.4 Hz, 2H), 1.36 (t, *J* = 6.7 Hz, 2H), 1.28 (d, *J* = 4.1 Hz, 5H), 0.21 – 0.07 ppm (m, 9H). (see Spectrum)

^{13}C-NMR (125.65 MHz, $CDCl_3$): δ = 174.3, 133.5, 129.0, 105.6, 89.3, 58.5, 51.4, 34.0, 29.1, 29.0 (2C), 28.9, 27.5, 24.8, -0.2 ppm. (see Spectrum)

TLC: Rf = 0.30 (10% ethyl acetate in hexanes, CAM).

Application (B): Synthesis of the sex pheromone of the Horse-Chestnut Leaf Miner

Upscaled, modified versions of our general procedures for the preparation of alkenyl thianthren-5-ium salts and unsaturated carbonyls were used as follows: (1) Tf_2O instead of TFAA and TfOH was used to demonstrate the feasibility of this alternative process; (2) No counterion exchange was performed as triflates are also appropriate starting materials of the TT-Kornblum reaction; (3) $NaHCO_3$ instead of K_2CO_3 and elevated temperature were used as more suitable conditions to promote the formation of the desired (*E*)-enal.

Preparation of ester SI7

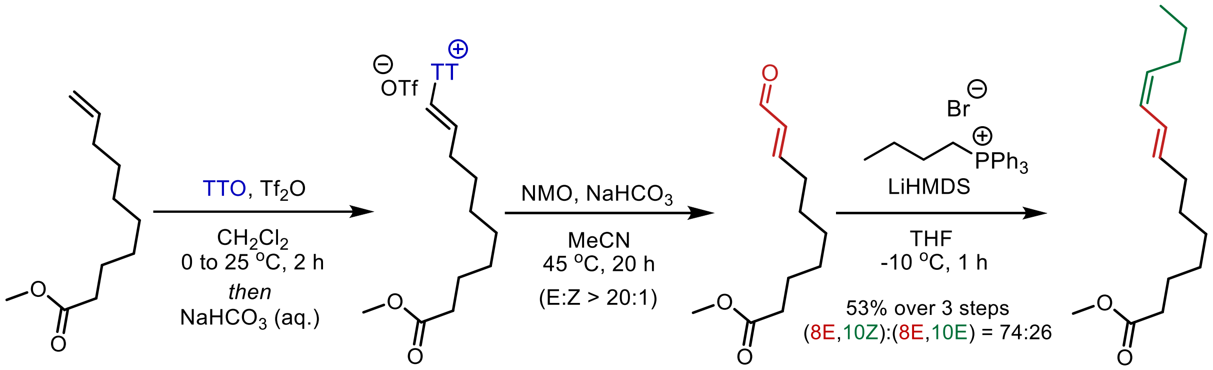

SI-7 [1.26 g]

A solution of methyl 9-decenoate (1.84 g, 10.0 mmol, 1 equiv) and thianthrene S-oxide (TTO, 2.56 g, 11.0 mmol, 1.10 equiv) in anhydrous dichloromethane (100 mL, 0.1 M) was cooled to 0 °C (internal temperature) under argon atmosphere. Then, trifluoromethanesulfonic anhydride (2.11 mL, 12.5 mmol, 1.25 equiv) was added dropwise, while maintaining the internal temperature below 10 °C. An immediate color change of the reaction mixture was observed upon addition and the lilac solution was stirred at 0 °C for a further 1 h. Then, the reaction mixture was warmed to 25 °C and stirred at this temperature for an additional 1 h, when a color change to brown was observed and analysis by TLC indicated full conversion. At this point, saturated aqueous sodium bicarbonate solution (100 mL) was added, and the two-phase mixture was vigorously stirred at 25 °C for 15 min. [Note 1] The phases were separated, and the organic phase was dried over sodium sulfate. The dried solution was filtered, and the filtrate was concentrated under reduced pressure to yield the crude thianthren-5-ium salt.

[Note 1]: This step promotes the formation of alkenyl thianthrenium salts from the precursor dicationic species.

A solution of the crude alkenyl thianthren-5-ium salt in acetonitrile (100 mL, 0.1 M) was stirred under argon atmosphere at 45 °C. To this solution was added *N*-methylmorpholine *N*-oxide monohydrate (NMO, 5.41 g, 40 mmol, 4.00 equiv) and well-powdered sodium bicarbonate (4.20 g, 50 mmol, 5.00 equiv) in one portion, and the suspension was stirred at this temperature for 20 h. [Note 2] Then, saturated aqueous sodium bicarbonate solution (200 mL) and diethyl ether (400 mL) were added subsequently. The layers were separated, and the organic phase was washed with saturated aqueous sodium bicarbonate solution (2 × 200 mL) and brine (200 mL), and was dried over sodium sulfate. The dried solution was filtered, and the filtrate was concentrated under reduced pressure. [Note 3] The crude enal was used in the next step without further purification.

[Note 2]: Technical grade acetonitrile and NMO monohydrate were used as the reaction is not sensitive to water.
[Note 3]: Water bath temperature was set to 30 °C due to the possible volatility of the product.

A suspension of butyltriphenylphosphonium bromide (3.99 g, 10.0 mmol, 1.00 equiv) in anhydrous tetrahydrofuran (75 mL) was cooled to -15 °C (±2 °C, internal temperature). Then, lithium bis(trimethylsilyl)amide in tetrahydrofuran (LiHMDS, 10.0 mL, 1.0 M, 10.0 mmol, 1.00 equiv) was added dropwise, while maintaining the internal temperature below -10 °C. The bright orange mixture was warmed to 0 °C and stirred at this temperature for an additional 40 min. After cooling back to -15 °C, a solution of the crude enal in anhydrous tetrahydrofuran (25 mL) was added dropwise while maintaining the internal temperature below -10 °C and the mixture was stirred at this temperature for an additional 60 min. At this point, analysis by TLC indicated full conversion, and saturated aqueous sodium bicarbonate solution (200 mL) was added. The well-stirred two-phase system was allowed to warm to 25 °C. Then, diethyl ether (400 mL) was added, and the phases were separated. The organic phase was washed with saturated aqueous sodium bicarbonate solution (2 × 200 mL) and brine (200 mL), and was dried over sodium sulfate. The dried solution was filtered, and the filtrate was concentrated under reduced pressure. The residue was redissolved in hexanes (100 mL), filtered and the precipitate was washed with hexanes. [Note 4] The filtrate was concentrated under reduced pressure and the residue was purified by flash column chromatography on silica gel (0–20% ethyl acetate in hexanes) to afford diene **SI7**(1.26 g, 5.29 mmol, 53%, *Z*:*E* = 74:26) as a colorless oil.

[Note 4]: The washing was continued until the washing solution did not contain the desired product as indicated by TLC analysis.

***E*,*Z*-SI7**

^{1}H-NMR (499.64 MHz, $CDCl_3$): δ = 6.31 – 6.23 (m, 1H), 6.03 – 5.90 (m, 1H), 5.63 (dt, *J* = 14.6, 7.0 Hz, 1H), 5.30 (dt, *J* = 10.9, 7.6 Hz, 1H), 3.66 (s, 3H), 2.29 (td, *J* = 7.5, 2.2 Hz, 2H), 2.16 – 2.11 (m, 1H), 2.08 (q, *J* = 7.5 Hz, 1H), 2.03 (t, *J* = 7.1 Hz, 1H), 1.63 – 1.59 (m, 2H), 1.42 – 1.39 (m, 4H), 1.34 – 1.29 (m, 5H), 0.90 ppm (t, *J* = 7.4 Hz, 3H). (see Spectrum)

^{13}C-NMR (125.65 MHz, $CDCl_3$): δ = 174.3, 134.4, 129.9, 128.7, 125.7, 51.4, 34.0, 32.7, 29.7, 29.1, 29.0, 28.8, 24.9, 22.9, 13.8 ppm. (see Spectrum)

***E*,*E*-SI7**

^{1}H-NMR (499.64 MHz, $CDCl_3$): δ = 6.03 – 5.90 (m, 2H), 5.58 – 5.51 (m, 2H), 3.66 (s, 3H), 2.29 (td, *J* = 7.5, 2.2 Hz, 2H), 2.16 – 2.11 (m, 1H), 2.08 (q, *J* = 7.5 Hz, 1H), 2.03 (t, *J* = 7.1 Hz, 1H), 1.63 – 1.59 (m, 2H), 1.42 – 1.39 (m, 4H), 1.34 – 1.29 (m, 5H), 0.89 ppm (t, *J* = 7.2 Hz, 3H). (see Spectrum)

^{13}C-NMR (125.65 MHz, $CDCl_3$): δ = 174.3, 132.3, 132.1, 130.42, 130.39, 51.4, 34.7, 32.5, 29.7, 29.1, 29.0, 28.8, 24.9, 22.5, 13.7 ppm. (see Spectrum)

HRMS (EI): M^+ calcd. for $[C_{15}H_{26}O_2]^+$ 238.1927, found 238.1924

TLC: Rf = 0.45 (10% ethyl acetate in hexanes, CAM).

Preparation of aldehyde 7

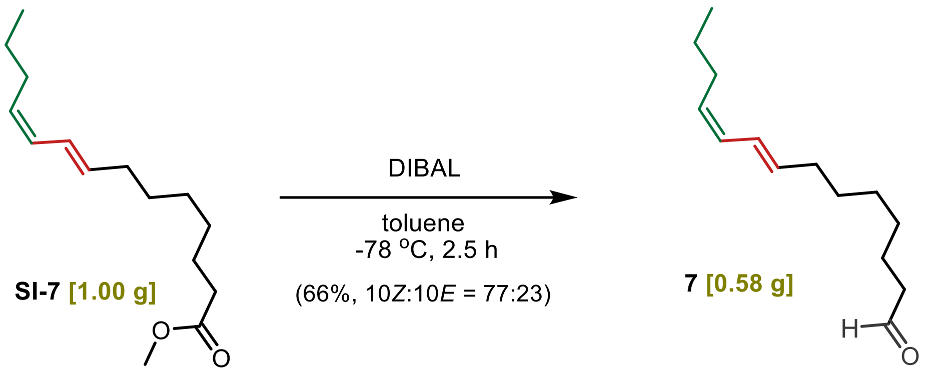

A solution of ester **SI7** (1.00 g, 4.20 mmol, 1 equiv) in anhydrous toluene (21 mL, 0.2 M) was cooled to -78 °C (±2 °C, internal temperature). Then, diisobutylaluminium hydride in hexanes (DIBAL, 4.82 mL, 1.0 M, 4.82 mmol, 1.15 equiv) was added dropwise, while maintaining the internal temperature below -73 °C. The mixture was stirred at this temperature for an additional 2 h, when analysis by TLC indicated incomplete conversion. Thus, a further portion of DIBAL in hexanes (0.42 mL, 1.0 M, 0.42 mmol, 0.10 equiv) was added dropwise while maintaining the internal temperature below -73 °C. [Note 1] After stirring at this temperature for an additional 20 min, saturated aqueous Rochelle salt solution (50 mL) was added. The well-stirred two-phase system was allowed to warm to 25 °C and stirred at this temperature for 14 h. Then, diethyl ether (30 mL) was added, and the phases were separated. The organic phase was dried over sodium sulfate. The dried solution was filtered, and the filtrate was concentrated under reduced pressure. The residue was purified by flash column chromatography on silica gel (0–20% ethyl acetate in hexanes) to afford diene **7** (0.58 g, 2.76 mmol, 66%, 10*Z*:10*E* = 77:23) as a colorless oil.

[Note 1]: Full conversion is targeted as the starting material has a similar polarity to the desired product. Overreduction to the alcohol does not cause isolation difficulties due to significantly different polarities.

***Z,E*-7**[16]

^{1}H-NMR (499.64 MHz, $CDCl_3$): δ = 9.76 (t, *J* = 1.8 Hz, 1H), 6.34 – 6.26 (m, 1H), 5.99 – 5.90 (m, 1H), 5.63 (dt, *J* = 14.6, 7.0 Hz, 1H), 5.31 (dt, *J* = 10.8, 7.6 Hz, 1H), 2.42 (td, *J* = 7.4, 1.9 Hz, 2H), 2.17 – 2.00 (m, 5H), 1.62 (ddd, *J* = 11.6, 5.9, 2.7 Hz, 2H), 1.40 (ddt, *J* = 10.6, 7.3, 3.4 Hz, 3H), 1.35 – 1.29 (m, 4H), 0.90 ppm (dt, *J* = 10.0, 7.4 Hz, 3H). (see Spectrum)

^{13}C-NMR (125.65 MHz, $CDCl_3$): δ = 202.9, 134.3, 130.0, 128.7, 125.8, 43.9, 32.7, 29.7, 29.1, 29.0, 28.9, 22.9, 22.0, 13.8 ppm. (see Spectrum)

TLC: Rf = 0.40 (10% ethyl acetate in hexanes, CAM).

[16] P. Chourreu, O. Guerret, L. Guillonneau, E. Gayon, G. Lefèvre, *Org. Process Res. Dev.* **2020**, *24*, 1335-1340.

Application (C): Synthesis of the sex pheromone of Lobesia Botrana

Upscaled, modified versions of our general procedures for the preparation of alkenyl thianthren-5-ium salts and unsaturated carbonyls were used as follows: (1) Tf_2O instead of TFAA and TfOH was used to demonstrate the feasibility of this alternative process; (2) No counterion exchange was performed as triflates are also appropriate starting materials of the TT-Kornblum reaction; (3) $NaHCO_3$ instead of K_2CO_3 and elevated temperature were used as more suitable conditions to promote the formation of the desired (*E*)-enal.

Preparation of diene 8

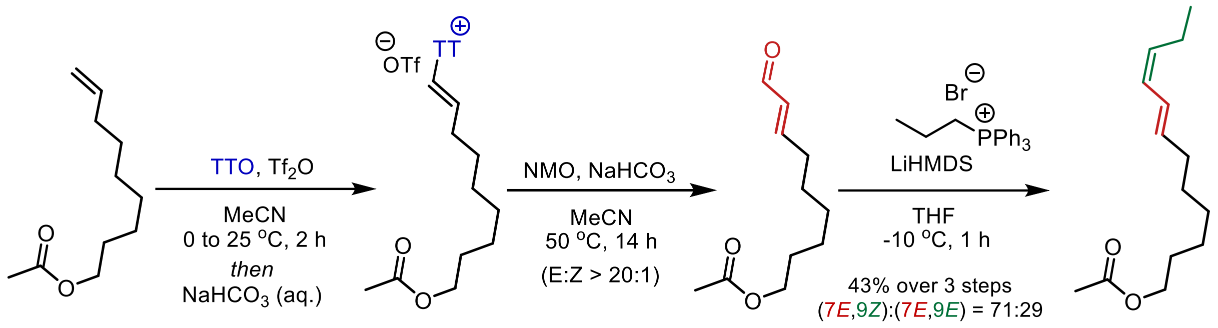

8 [0.97 g]

A suspension of 8-nonenyl acetate (1.84 g, 10.0 mmol, 1 equiv) and thianthrene S-oxide (TTO, 2.56 g, 11.0 mmol, 1.10 equiv) in anhydrous acetonitrile (50 mL, 0.2 M) was cooled to 0 °C (internal temperature) under argon atmosphere. Then, trifluoromethanesulfonic anhydride (2.11 mL, 12.5 mmol, 1.25 equiv) was added dropwise, while maintaining the internal temperature below 10 °C. An immediate color change of the reaction mixture was observed upon addition and the lilac solution was stirred at 0 °C for a further 1 h. Then, the reaction mixture was warmed to 25 °C and stirred at this temperature for an additional 1 h, when analysis by TLC indicated full conversion. At this point, the reaction mixture was concentrated under reduced pressure and subsequently dissolved in dichloromethane (100 mL). Saturated aqueous sodium bicarbonate solution (100 mL) was added, and the two-phase mixture was vigorously stirred at 25 °C for 15 min. [Note 1] The phases were separated, and the organic phase was dried over sodium sulfate. The dried solution was filtered, and the filtrate was concentrated under reduced pressure to yield the crude thianthren-5-ium salt.

[Note 1]: This step promotes the formation of alkenyl thianthrenium salts from the precursor dicationic species.

A solution of the crude alkenyl thianthren-5-ium salt in acetonitrile (100 mL, 0.1 M) was stirred under argon atmosphere at 50 °C. To this solution was added *N*-methylmorpholine *N*-oxide monohydrate (NMO, 5.41 g, 40 mmol, 4.00 equiv) and well-powdered sodium bicarbonate (4.20 g, 50 mmol, 5.00 equiv) in one portion, and the suspension was stirred at this temperature for 14 h. [Note 2] Then, saturated aqueous sodium bicarbonate solution (200 mL) and diethyl ether (400 mL) were added subsequently. The layers were separated, and the organic phase was washed with saturated aqueous sodium bicarbonate solution (2 × 200 mL) and brine (200 mL), and was dried over sodium sulfate. The dried solution was filtered, and the filtrate was concentrated under reduced pressure. [Note 3] The crude enal was used in the next step without further purification.

[Note 2]: Technical grade acetonitrile and NMO monohydrate were used as the reaction is not sensitive to water.
[Note 3]: Water bath temperature was set to 30 °C due to possible volatility of the product.

A suspension of propyltriphenylphosphonium bromide (3.85 g, 10.0 mmol, 1.00 equiv) in anhydrous tetrahydrofuran (75 mL) was cooled to -15 °C (±2 °C, internal temperature). Then, lithium bis(trimethylsilyl)amide in tetrahydrofuran (LiHMDS, 10.0 mL, 1.0 M, 10.0 mmol, 1.00 equiv) was added dropwise, while maintaining the internal temperature below -10 °C. The bright orange mixture was warmed to 0 °C and stirred at this temperature for an additional 40 min. After cooling back to -15 °C, a solution of the crude enal in anhydrous tetrahydrofuran (25 mL) was added dropwise while maintaining the internal temperature below -10 °C and the mixture was stirred at this temperature for an additional 60 min. At this point, analysis by TLC indicated full conversion, and saturated aqueous sodium bicarbonate solution (200 mL) was added. The well-stirred two-phase system was allowed to warm to 25 °C. Then, diethyl ether (400 mL) was added, and the phases were separated. The organic phase was washed with saturated aqueous sodium bicarbonate solution (2 × 200 mL) and brine (200 mL), and was dried over sodium sulfate. The dried solution was filtered, and the filtrate was concentrated under reduced pressure. The residue was redissolved in hexanes (100 mL), filtered and the precipitate was washed with hexanes. [Note 4] The filtrate was concentrated under reduced pressure and the residue was purified by flash column chromatography on silica gel (0–20% ethyl acetate in hexanes) to afford diene **8** (0.97 g, 4.32 mmol, 43%, *Z*:*E* = 71:29) as a colorless oil.

[Note 4]: The washing was continued until the washing solution did not contain the desired product as indicated by TLC analysis.

^{1}H-NMR (499.64 MHz, $CDCl_3$): δ = 6.34 – 6.25 (m, 1H), 5.90 (tq, *J* = 10.9, 1.3 Hz, 1H), 5.64 (dt, *J* = 14.7, 7.0 Hz, 1H), 5.29 (dt, *J* = 10.8, 7.5 Hz, 1H), 4.04 (td, *J* = 6.7, 2.2 Hz, 2H), 2.17 (pd, *J* = 7.6, 1.6 Hz, 1H), 2.12 – 2.05 (m, 3H), 2.04 (s, 3H), 1.65 – 1.56 (m, 2H), 1.43 – 1.29 (m, 6H), 0.99 ppm (td, *J* = 7.5, 1.6 Hz, 3H). (see Spectrum)

^{13}C-NMR (125.65 MHz, $CDCl_3$): δ = 171.2, 134.3, 131.7, 127.9, 125.6, 64.5, 32.7, 29.2, 28.8, 28.5, 25.7, 21.0 (2C), 14.3 ppm. (see Spectrum)

TLC: Rf = 0.40 (10% ethyl acetate in hexanes, CAM).

The acquired spectral data was in accordance with the previously reported results.[17]

[17] G. Cahiez, O. Guerret, A. Moyeux, S. Dufour, N. Lefevre, *Org. Process Res. Dev.* **2017**, *21*, 1542-1546.

Application (D): Synthesis of the sex pheromone of the Red Bollworm Moth

Upscaled, one-pot versions of our general procedures for the preparation of alkenyl thianthren-5-ium salts and unsaturated carbonyls were used as follows: (1) No aqueous work-up was performed in the first step, thianthren-5-ium species were directly transferred into conditions of the TT-Kornblum reaction. (2) The reaction was conducted at 0 °C instead of -20 °C to promote the *in-situ* transformation of the different thianthrenium species into the alkenyl thianthrenium salt.

Preparation of diene 9

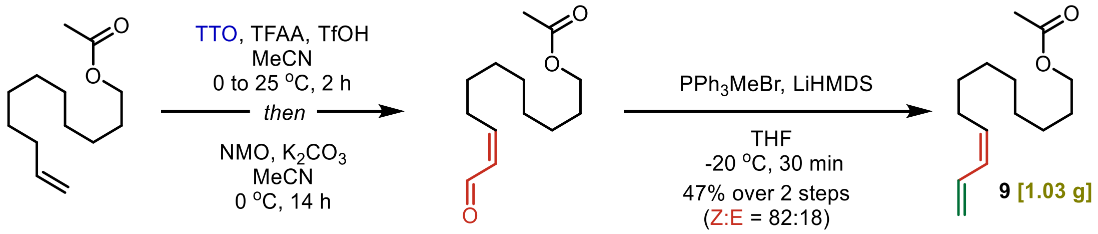

A suspension of undec-10-en-1-yl acetate (2.13 g, 10.0 mmol, 1 equiv) and thianthrene S-oxide (TTO, 2.39 g, 10.3 mmol, 1.03 equiv) in anhydrous acetonitrile (30 mL, 0.3 M) was cooled to 0 °C (internal temperature) under argon atmosphere. Then, trifluoroacetic anhydride (TFAA, 4.24 mL, 40.0 mmol, 3.00 equiv) and trifluoromethanesulfonic acid (1.78 mL, 30.0 mmol, 2.00 equiv) were added consecutively dropwise, while maintaining the internal temperature below 10 °C. [Note 1] An immediate color change of the reaction mixture was observed upon the first addition step and the lilac solution was stirred at 0 °C for a further 1 h. Then, the reaction mixture was warmed to 25 °C and stirred at this temperature for an additional 1 h, when analysis by TLC indicated full conversion. At this point, the reaction mixture was concentrated under reduced pressure to yield the crude thianthrenium salt. [Note 2]

[Note 1]: Caution! Special attention should be taken when adding the corrosive trifluoromethanesulfonic acid dropwise at this scale as disposable syringes/needles may disintegrate.
[Note 2]: Water jet vacuum pumps were used with gentle heating by a 30 °C water bath.

A suspension of *N*-methylmorpholine *N*-oxide (NMO, 4.69 g, 40 mmol, 4.00 equiv) and well powdered, oven-dried potassium carbonate (6.91 g, 50 mmol, 5.00 equiv) in anhydrous acetonitrile (75 mL) was cooled to 0 °C (±2 °C, internal temperature) and stirred at this temperature for 30 min. [Notes 3,4] Then, a solution of the crude alkenyl thianthren-5-ium salt in anhydrous acetonitrile (25 mL) was added dropwise while maintaining the internal temperature below 5 °C. The reaction mixture was stirred at this temperature for 14 h when analysis by TLC indicated full conversion. Then, saturated aqueous sodium bicarbonate solution (400 mL) and dichloromethane (400 mL) were added subsequently. The layers were separated, and the organic phase was washed with saturated aqueous sodium bicarbonate solution (2 × 200 mL) and dried over sodium sulfate. [Note 5] The dried solution was filtered, and the filtrate was concentrated under reduced pressure. [Note 6] The crude enal was used in the next step without further purification.

[Note 3]: A low-temperature thermostat was used to control cooling bath temperature.
[Note 4]: This preliminary stirring ensures that the internal temperature stabilizes as initial overcooling to ca. -24 °C may occur due to the endothermic dissolution of K_2CO_3.
[Note 5]: These steps eliminate excess NMO and *N*-alkylated by-products.
[Note 6]: To avoid isomerization of the (*Z*)-enal, the water bath temperature was set to 30 °C.

A suspension of methyltriphenylphosphonium bromide (2.68 g, 7.5 mmol, 0.75 equiv) in anhydrous tetrahydrofuran (46.7 mL) was cooled to -20 °C (±2 °C, internal temperature). Then, lithium bis(trimethylsilyl)amide in tetrahydrofuran (LiHMDS, 7.5 mL, 1.0 M, 7.5 mmol, 0.75 equiv) was added dropwise, while maintaining the internal temperature below -15 °C and the bright yellow mixture was stirred for an additional 30 min. Then, the mixture was warmed to 0 °C and stirred at this temperature for an additional 30 min. After cooling back to -20 °C, a solution of the crude enal in anhydrous tetrahydrofuran (20 mL) was added dropwise while maintaining the internal temperature below -15 °C and the mixture was stirred for an additional 30 min. At this point, saturated aqueous sodium bicarbonate solution (200 mL) was added, and the two-phase system was allowed to warm to 25 °C. Then, dichloromethane (400 mL) was added, and the phases were separated. The aqueous phase was extracted with dichloromethane (2 × 200 mL). The combined organic phases were washed with brine (300 mL) and dried over sodium sulfate. The dried solution was filtered, and the filtrate was concentrated under reduced pressure. The residue was purified by flash column chromatography on silica gel (0–20% ethyl acetate in hexanes) to afford diene **9** (1.03 g, 4.57 mmol, 46%, *Z*:*E* = 82:18) as a colorless oil.

^{1}H-NMR (499.64 MHz, $CDCl_3$): δ = 6.64 – 6.54 (m, 1H), 5.98 – 5.92 (m, 1H), 5.44 – 5.37 (m, 1H), 5.17 – 5.09 (m, 1H), 5.03 (dd, *J* = 10.2, 2.0 Hz, 1H), 4.11 – 3.97 (m, 2H), 2.18 – 2.10 (m, 2H), 2.00 (s, 3H), 1.57 (p, *J* = 6.8 Hz, 2H), 1.41 – 1.22 ppm (m, 10H). (see Spectrum)

^{13}C-NMR (125.65 MHz, $CDCl_3$): δ = 170.9, 132.7, 132.1, 129.1, 116.5, 64.4, 29.4, 29.2, 29.0 (2C), 28.4, 27.5, 25.7, 20.8 ppm. (see Spectrum)

TLC: Rf = 0.40 (10% ethyl acetate in hexanes, CAM).

The acquired spectral data was in accordance with the previously reported results.[18]

[18] (*Z*): D. Michelot, *Synthesis* **1983**, *1983*, 130-134. (*E*): G. Cahiez, V. Habiak, O. Gager, *Org. Lett.* **2008**, *10*, 2389–2392.

Application (E): Synthesis of the pear ester kairomone and fragrance

We implemented a slightly modified version of Wickens' cation pool approach[19] for the electrochemical oxidation of 1-octene using thianthrene. The formed thianthren-5-ium adducts were directly transferred into the conditions of the TT-Kornblum reaction, where a modified version of our general procedure for the preparation of unsaturated carbonyls was used as follows: (1) 2-MeTHF was used as a solvent instead of MeCN for easier removal of the electrolyte salt and because of its slightly higher volatility; (2) 10 equiv. of K_2CO_3 was used instead of 5 equiv, and the reaction was conducted at -10 °C instead of -20 °C to promote the *in situ* transformation of the different thianthrenium species into the alkenyl thianthren-5-ium salt. Finally, without isolation, the obtained enal compound was subjected to a Wittig reaction according to a modified version of Bestmann's procedure[20] to obtain the pear ester.

Preparation of ester 10

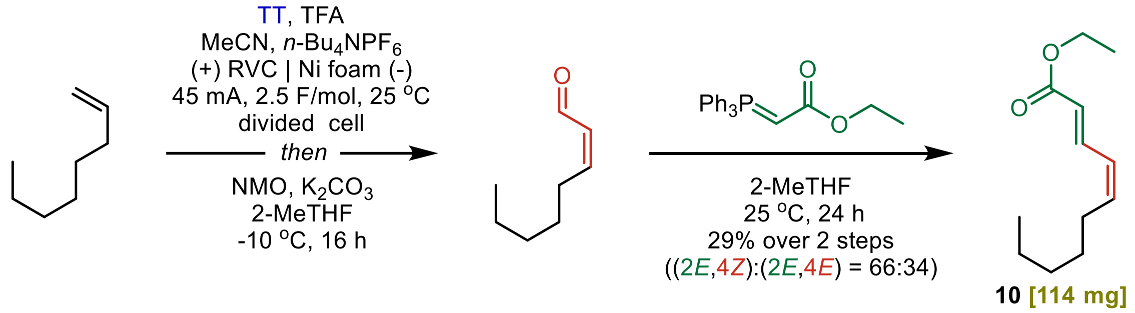

Trifluoroacetic acid (770 µL, 5 equiv, 10.0 mmol) was added to a solution of tetra-n-butylammonium hexafluorophosphate (620 mg, 0.8 equiv, 1.60 mmol) in anhydrous acetonitrile (7 mL, 0.2 M). This solution was transferred to the cathodic compartment of an IKA Pro-Divide divided electrochemical cell equipped with magnetic stirring bars. Next, 1-octene (224 mg, 1 equiv, 2.00 mmol) was added to a separate solution of tetra-n-butylammonium hexafluorophosphate (620 mg, 0.8 equiv, 1.60 mmol) in anhydrous acetonitrile (7 mL, 0.2 M). This solution in turn was transferred to the anodic compartment of the electrochemical cell. Finally, under stirring, thianthrene (TT, 649 mg, 1.5 equiv, 3.00 mmol) was added to the anodic compartment [Note 1]. The closing cap of the cell was equipped with a standard IKA RVC anode and a standard IKA nickel foam cathode, and the cell was sealed. Both compartments were flushed with argon gas through the septa on the top of the cell, after which argon-filled balloons were inserted into them. The cell was inserted into the IKA ElectraSyn 2.0 potentiostat and the reaction mixture was electrolyzed at a constant current of 45 mA for 2.5 F/mol of alkene, with stirring set to 750 RPM [Note 2]. Complete conversion of the alkene was achieved based on TLC and ^1H NMR measurement. Next, the cell was opened, and the lilac anodic solution was transferred into a 50 mL flask. The RVC anode and the anodic compartment were further washed with 7 mL anhydrous acetonitrile into the flask. Then, under reduced pressure, this anodic reaction mixture was evaporated to dryness to yield a crude mixture of different thianthrenium adducts.

[19] D. J. Wang, K. Targos, Z. K. Wickens, *J. Am. Chem. Soc.* **2021**, *143*, 21503-21510.
[20] H. J. Bestmann, J. Süß, *Liebigs Ann. Chem.* **1982**, *1982*, 363-365

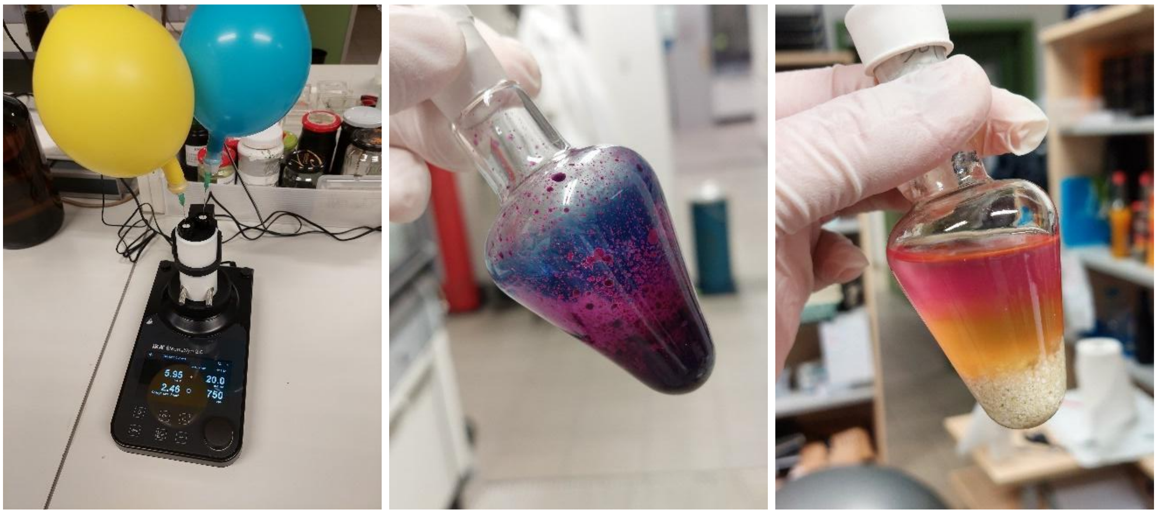

(A) Representative reaction setup with the IKA ElectraSyn 2.0 potentiostat; **(B)** flask containing the thianthrenium adducts after evaporation; **(C)** flask containing the thianthrenium adducts after addition of K_2CO_3 and NMO

To this flask, a magnetic stirring bar and 20 mL anhydrous 2-methyltetrahydrofuran (0.1 M) were added, and the contents of the flask were suspended using sonication. The flask was flushed with argon gas, closed with a rubber septum, and equipped with an argon-filled balloon. Under stirring, the reaction mixture was cooled to -10 °C, and potassium carbonate (2.76 g, 10 equiv, 20.0 mmol) and *N*-methylmorpholine *N*-oxide (NMO, 937 mg, 4 equiv, 8.00 mmol) were added to it in one portion. Almost immediately, the intensive lilac color of the suspension started to fade and transform into a pale-yellow color. The resulting suspension was stirred for further 16 hours at -10 °C, after which complete conversion of the alkenyl thianthrenium salt could be observed using TLC and ^{1}H NMR measurement. Next, while still cold, an aqueous 10% citric acid solution was added to the reaction mixture until neutral or slightly acidic pH is achieved (approx. 40-50 mL). The phases were separated [Note 3], and the organic layer was further washed with 2x30 mL brine. Then, the organic phase was dried over sodium sulfate and filtered, to yield a solution of the crude enal, which was used in the next synthetic step without further purification.

In the final synthetic step, ethyl 2-(triphenyl-λ^5-phosphaneylidene)acetate (766 mg, 1.1 equiv, 2.20 mmol) was added into an argon-flushed 50 mL flask equipped with a magnetic stirring bar, rubber septum, and an argon-filled balloon. The solution of the crude enal in 2-methyltetrahydrofuran (20 mL, 0.1 M) was added to the flask, and the reaction mixture was stirred at 25 °C for 24 hours until complete conversion of the enal could be observed using TLC and ^{1}H NMR measurement. Next, the reaction mixture was concentrated under reduced pressure [Note 4] and 5 mL hexane was added. The resulting suspension was sonicated for 10 minutes and filtered. The filtrate containing the crude product was subjected to flash column chromatography on silica gel (gradient elution, 0–10% ethyl acetate in hexanes) [Note 5] to afford the pear ester (**10**) (114 mg, 581 µmol, 29%, (2*E*,4*Z*):(2*E*,4*E*) = 66:34) as a colorless oil.

[Note 1]: As the solubility of thianthrene is low in acetonitrile, complete dissolution of the solids will take place only as the electrochemical reaction consumes the thianthrene. Intensive stirring is needed to suspend the thianthrene so that the stirring bars of the Pro-Divide cell don't get stuck in the solid material.
[Note 2]: The IKA ElectraSyn was set up as follows: New experiments → Constant current → 45 mA → No reference electrode → Total charge → 2.0 mmol, 2.5 F/mol → No alternating polarity → Start → 750 RPM stirring
[Note 3]: In some cases, a part of the supporting electrolyte precipitated during the extraction steps producing a layer of precipitate between the organic and aqueous phase which made their separation cumbersome. Filtering of the biphasic mixture through pleated filter paper helped in these cases.
[Note 4]: Due to the volatility of the pear ester, extra care should be taken during the evaporations under reduced pressure. Using a warmer water bath (50-60 °C) and higher pressures (>300 mbar) usually gave better results.
[Note 5]: The remaining supporting electrolyte and residual polar solvents may cause the product to elute with the solvent front. In these cases, the fractions containing the product should be concentrated under reduced pressure and the flash chromatography should be repeated.

Pear ester (10):

^{1}H-NMR (499.64 MHz, $CDCl_3$): δ = 7.60 (ddd, J = 15.2, 11.7, 1.1 Hz, 1H), 6.18 – 6.08 (m, 2H), 5.85 (d, J = 15.0 Hz, 1H), 4.19 (dq, J = 8.9, 7.1 Hz, 2H), 2.29 (qd, J = 7.6, 1.6 Hz, 2H), 1.44 – 1.38 (m, 2H), 1.33 – 1.24 (m, 7H), 0.89 – 0.86 ppm (m, 3H). (see Spectrum)

^{13}C-NMR (125.65 MHz, $CDCl_3$): δ = 167.3, 141.7, 139.5, 126.4, 121.1, 60.2, 31.3, 29.0, 28.2, 22.5, 14.3, 14.0 ppm. (see Spectrum)

TLC: Rf = 0.43 (10% ethyl acetate in hexanes, 254 nm UV light or CAM).

The acquired spectral data was in accordance with the previously reported results.[15]

^{1}**H-NMR** (499.64 MHz, DMSO-d^6) of **2a** (see Procedure)

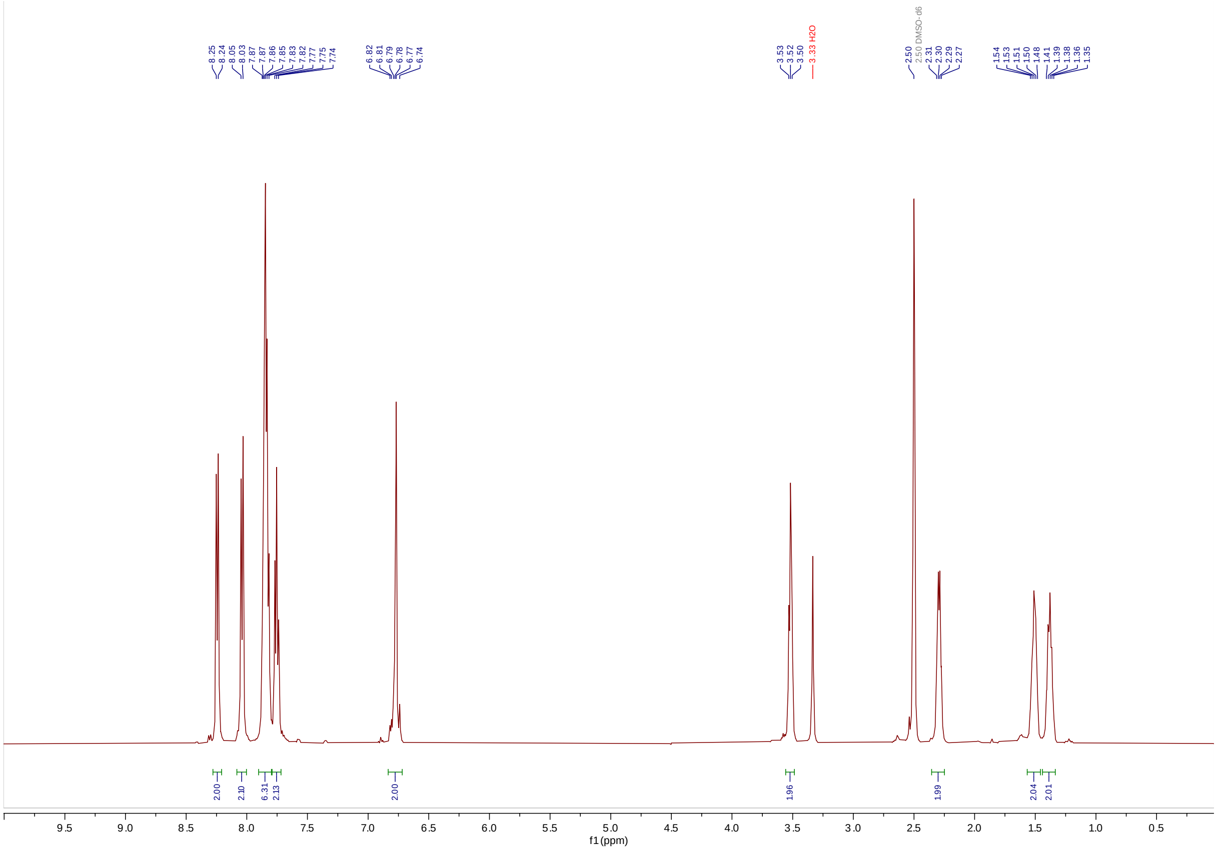

^{13}**C-NMR** (125.65 MHz, DMSO-d^6) of **2a** (see Procedure)

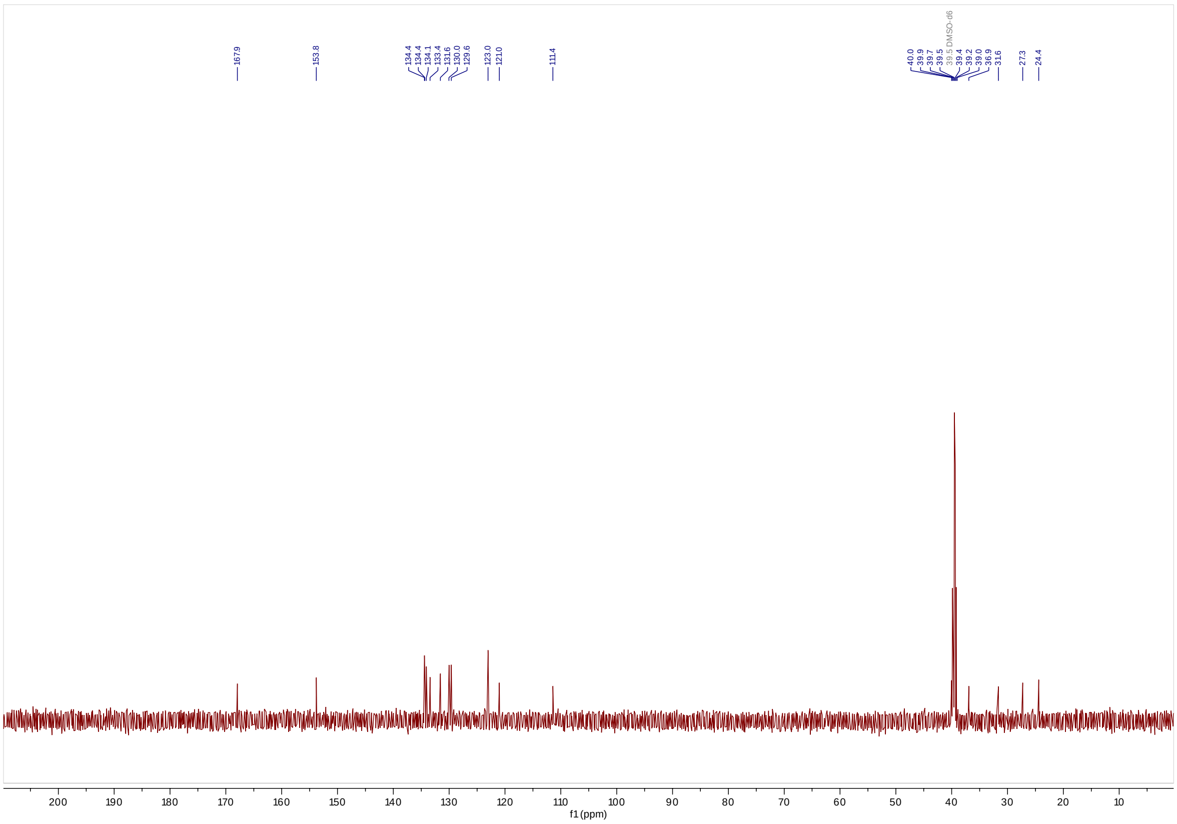

^{19}F-NMR (282.21 MHz, DMSO-*d*^{6}) of **2a**

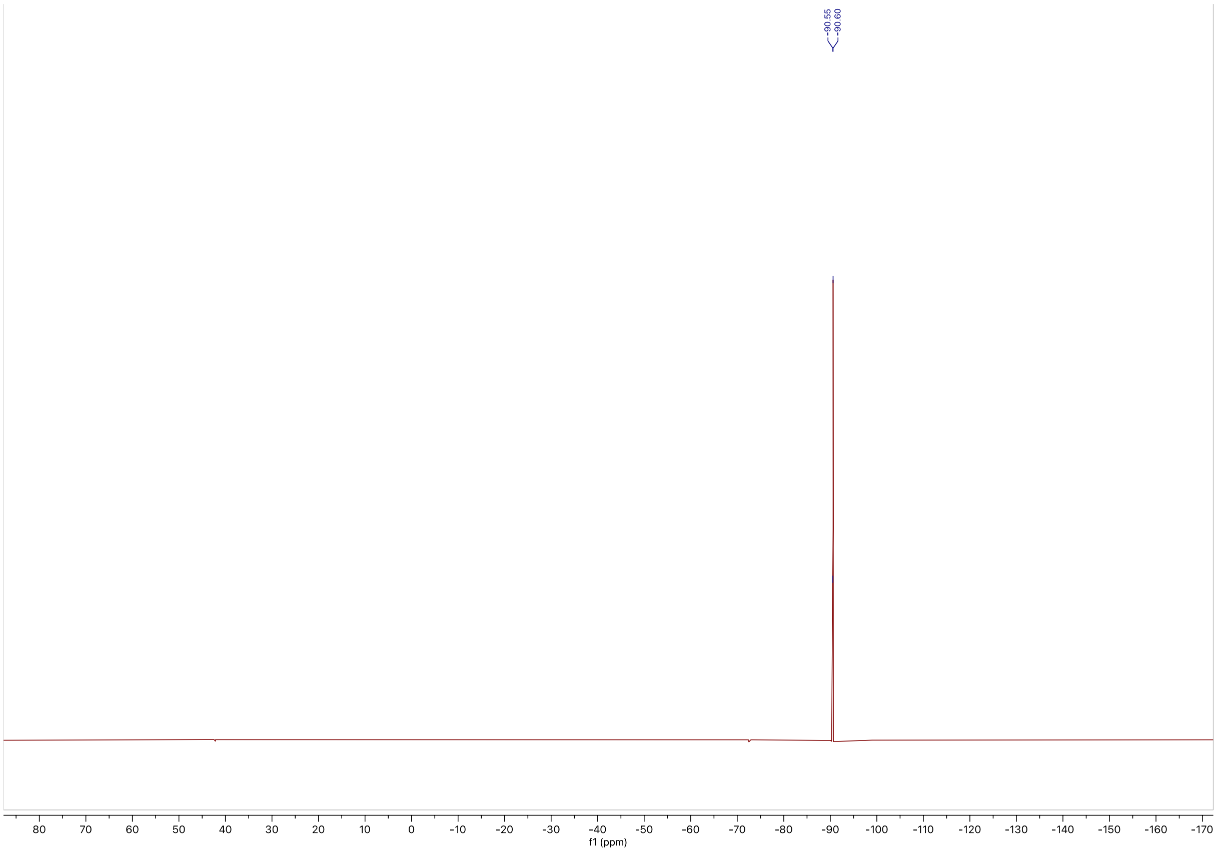

^{1}H-NMR (499.64 MHz, DMSO-d^{6}) of **2b** (see Procedure)

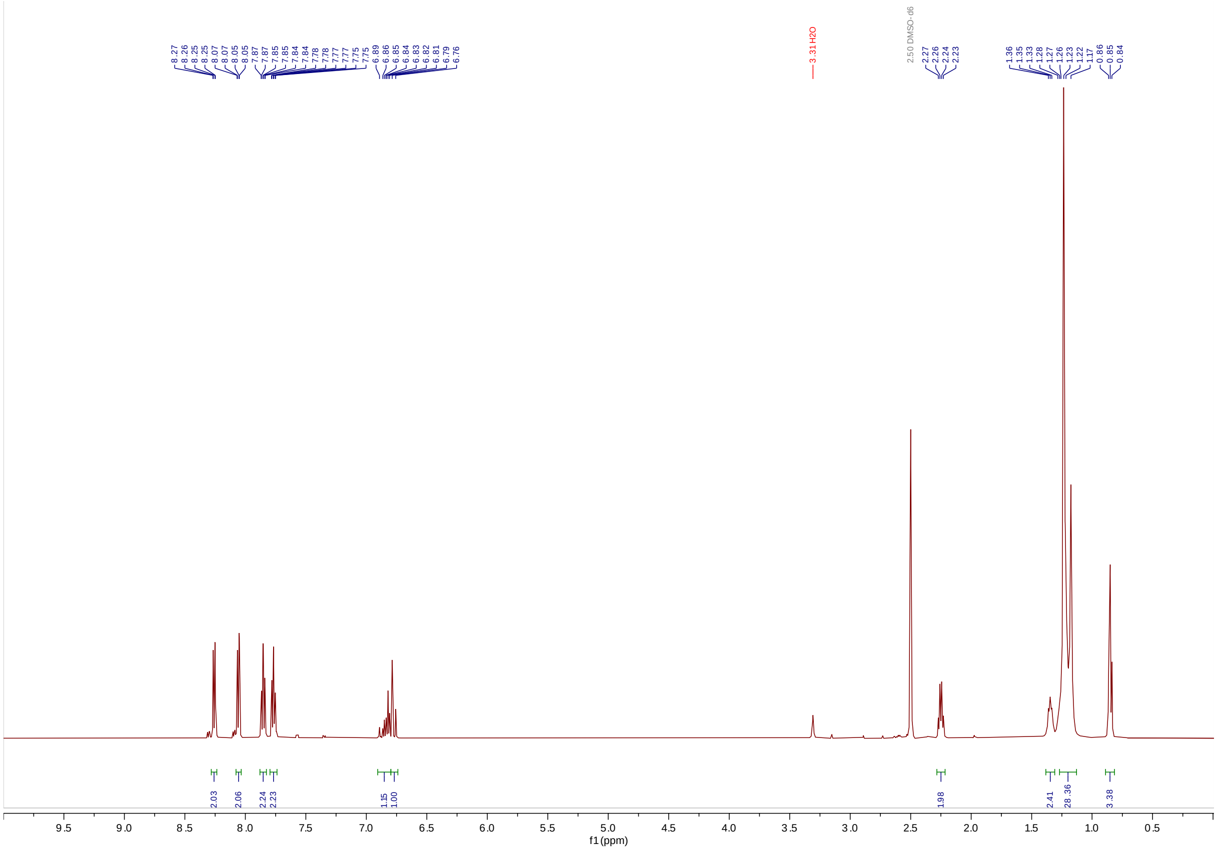

^{13}C-NMR (125.65 MHz, DMSO-d^{6}) of **2b** (see Procedure)

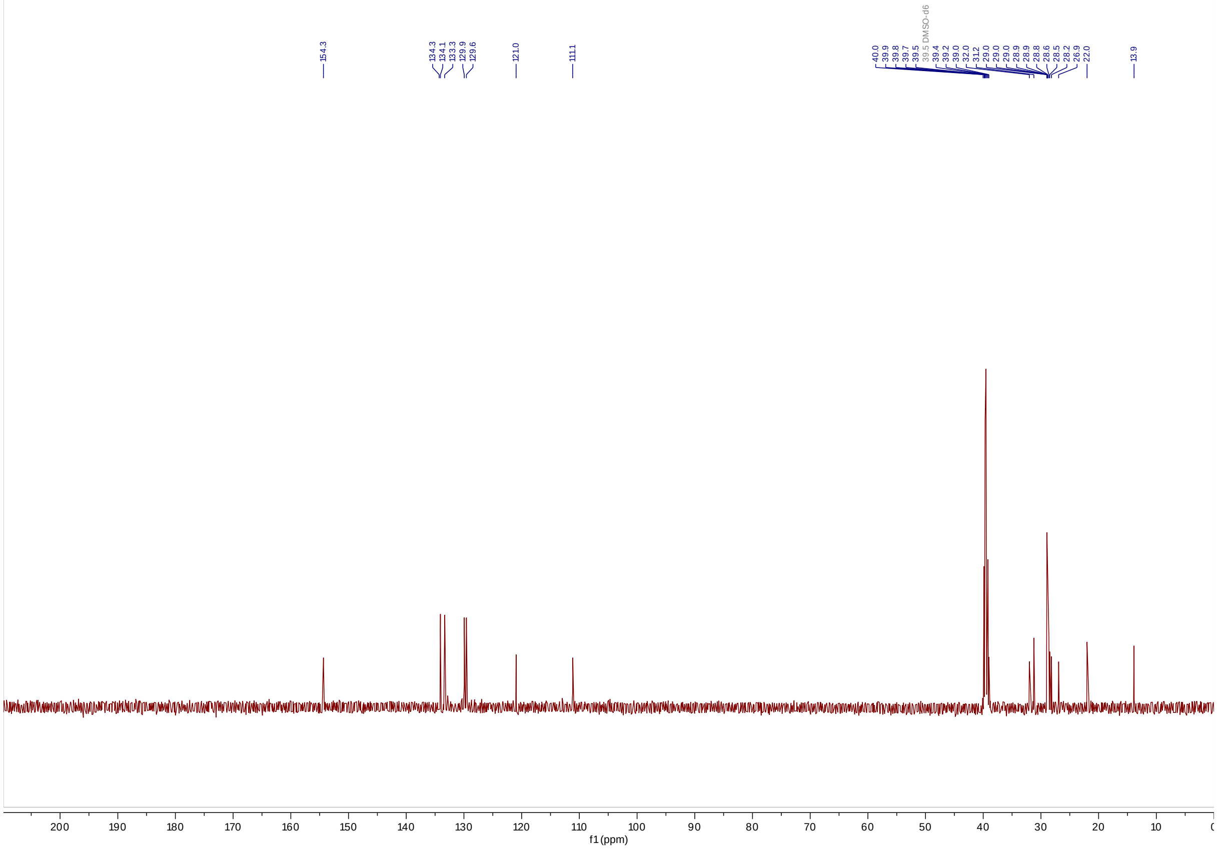

^{19}F-NMR (282.21 MHz, DMSO-*d*^{6}) of **2b**

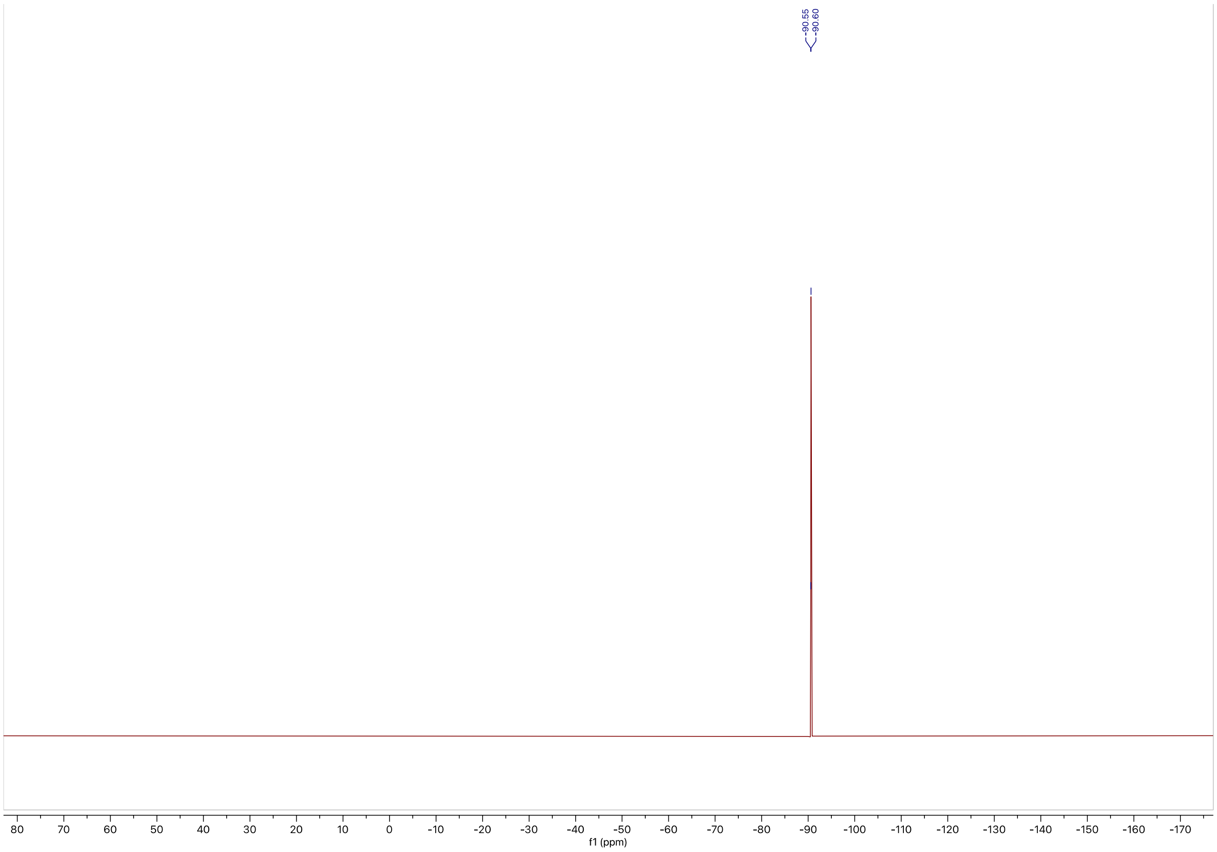

^{1}H-NMR (499.64 MHz, DMSO-*d*[6]) of **2c** (see Procedure)

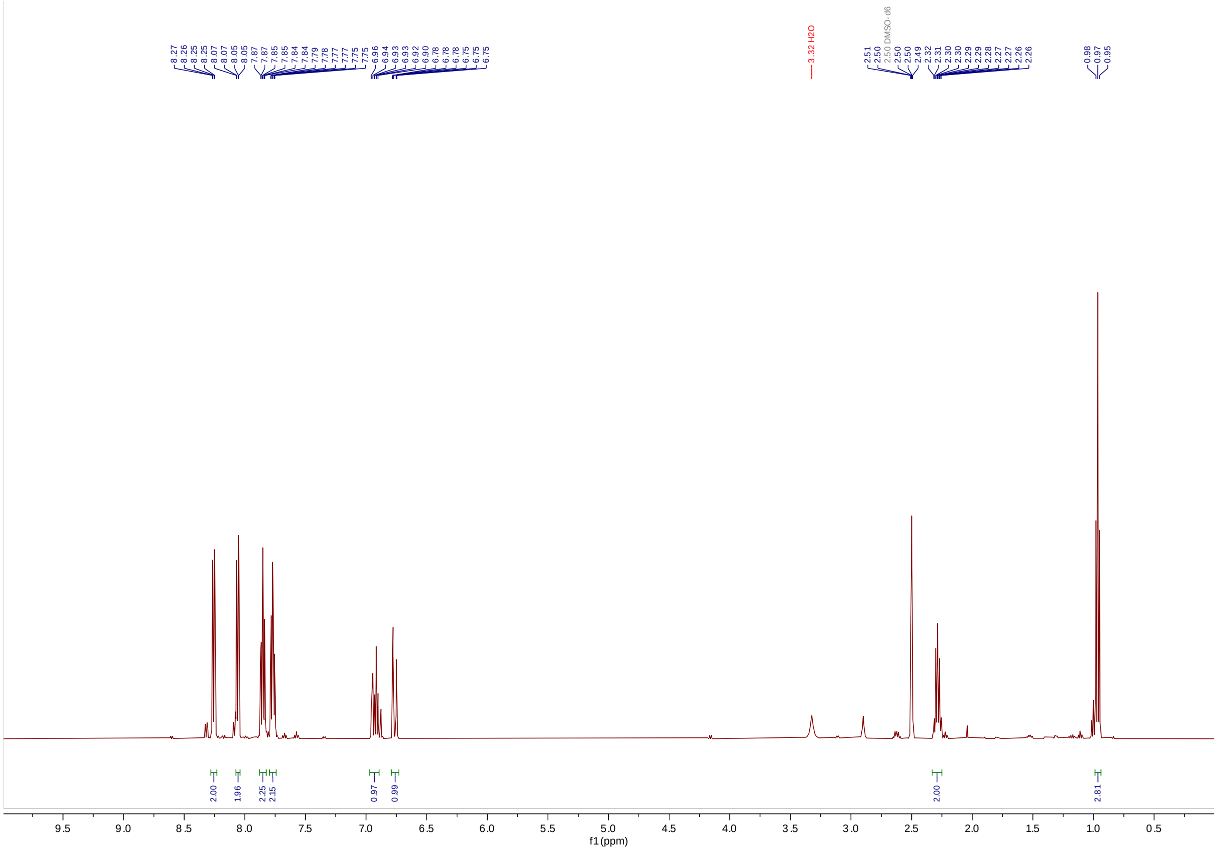

^{13}C-NMR (125.65 MHz, DMSO-*d*[6]) of **2c** (see Procedure)

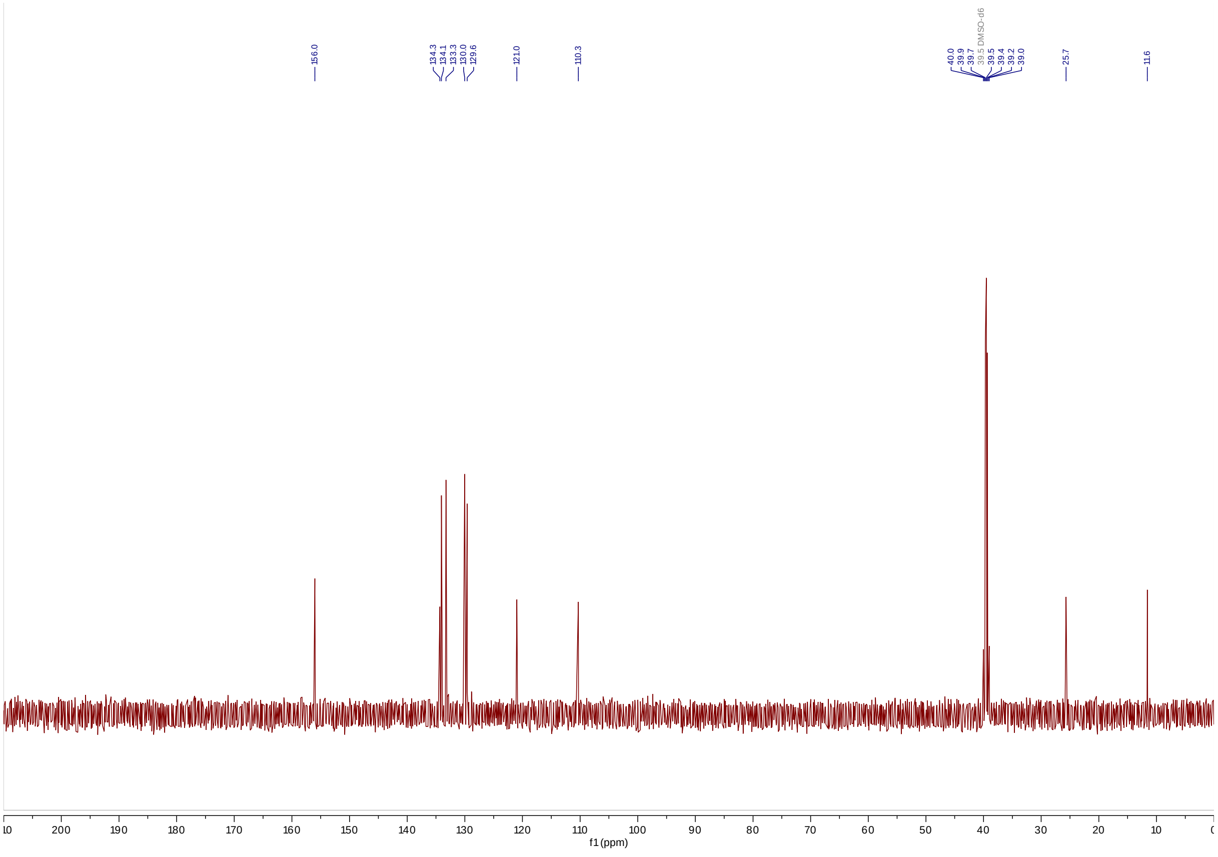

^{19}F-NMR (282.21 MHz, DMSO-*d*^{6}) of **2c**

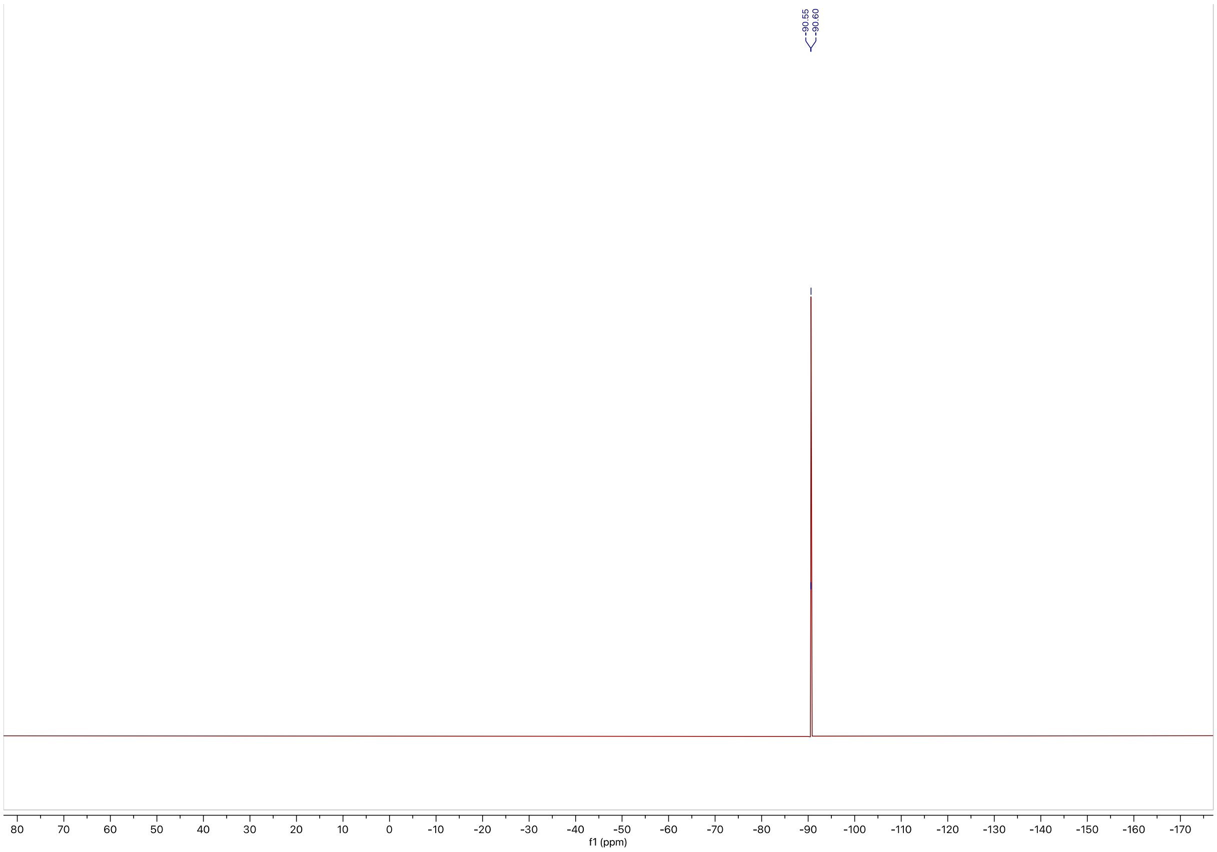

[1]**H-NMR** (499.64 MHz, $CDCl_3$) of **2d** and **2d**’ (see Procedure)

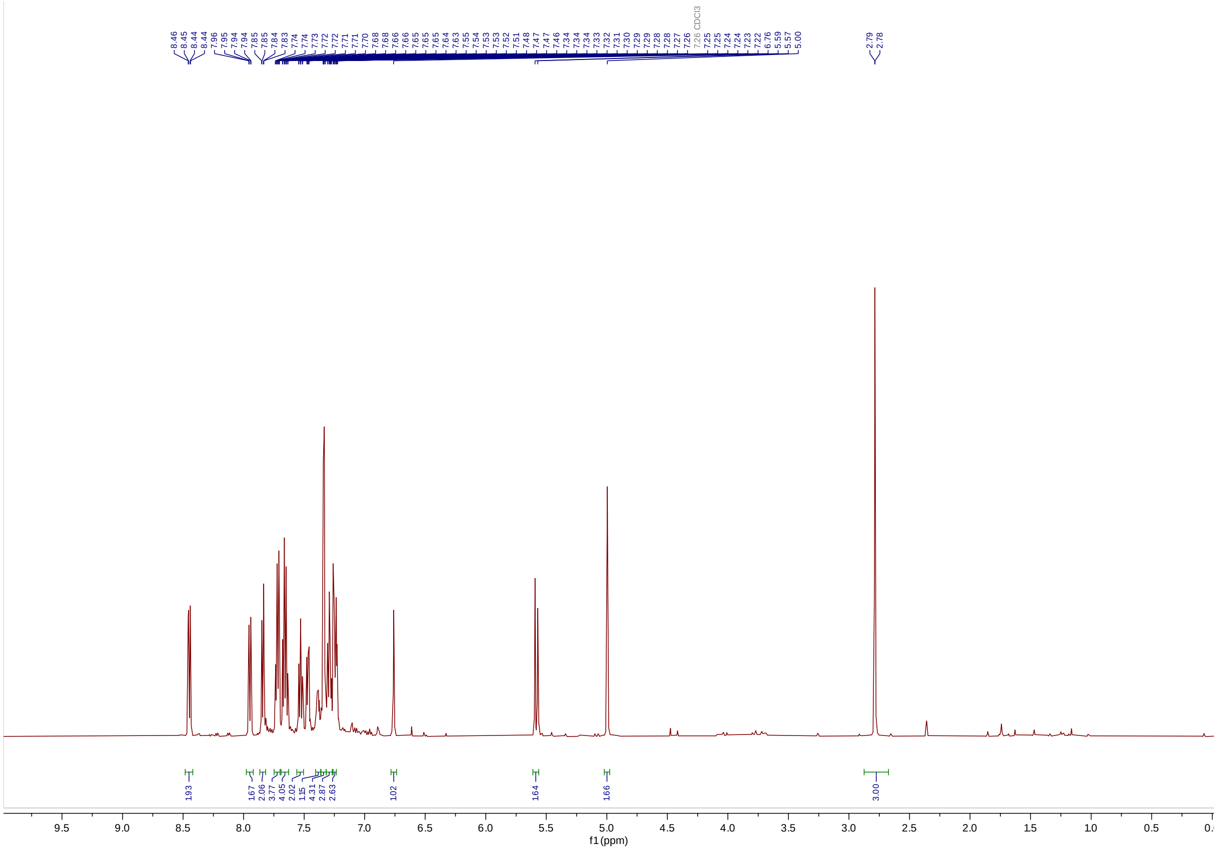

[13]**C-NMR** (125.65 MHz, $CDCl_3$) of **2d** and **2d**’ (see Procedure)

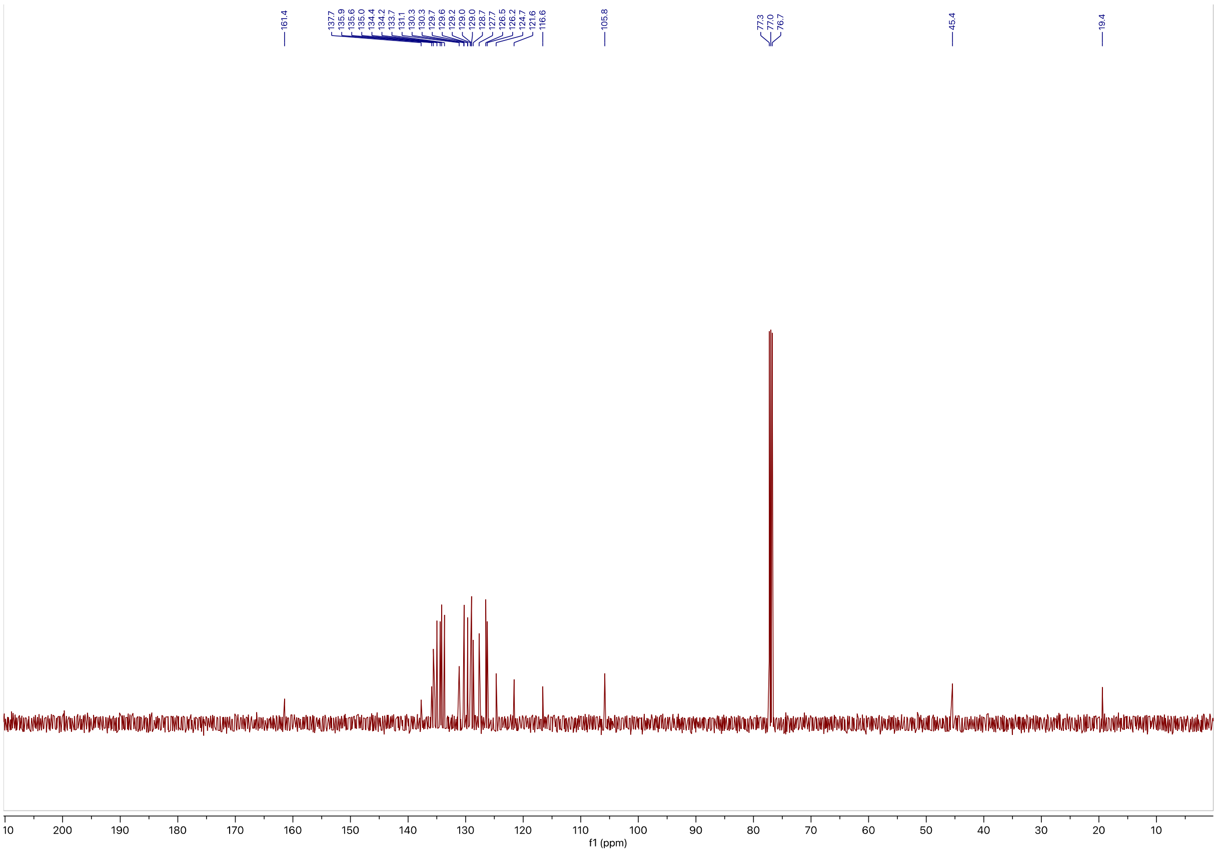

^{19}F-NMR (282.21 MHz, DMSO-d^6) of **2d** and **2d'**

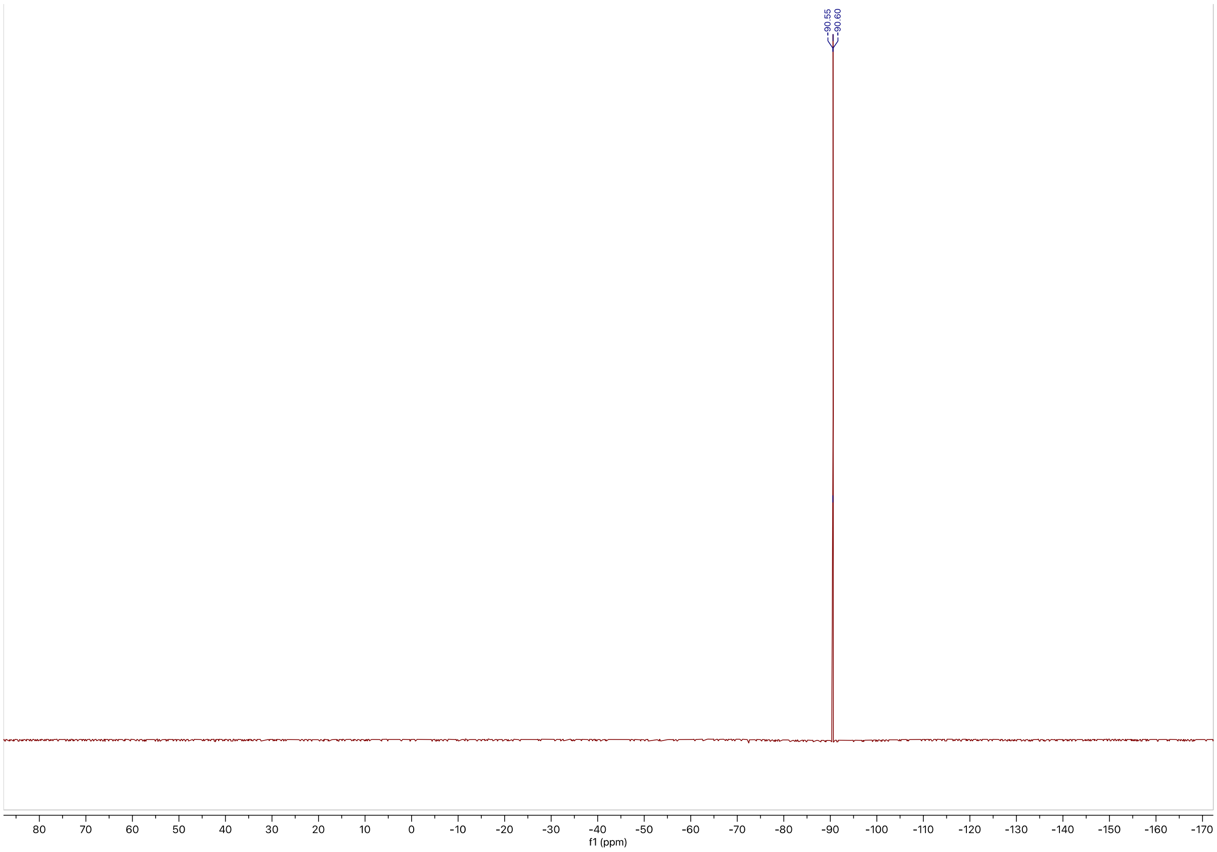

^{1}H-NMR (499.64 MHz, DMSO-d^6) of **2g** (see Procedure)

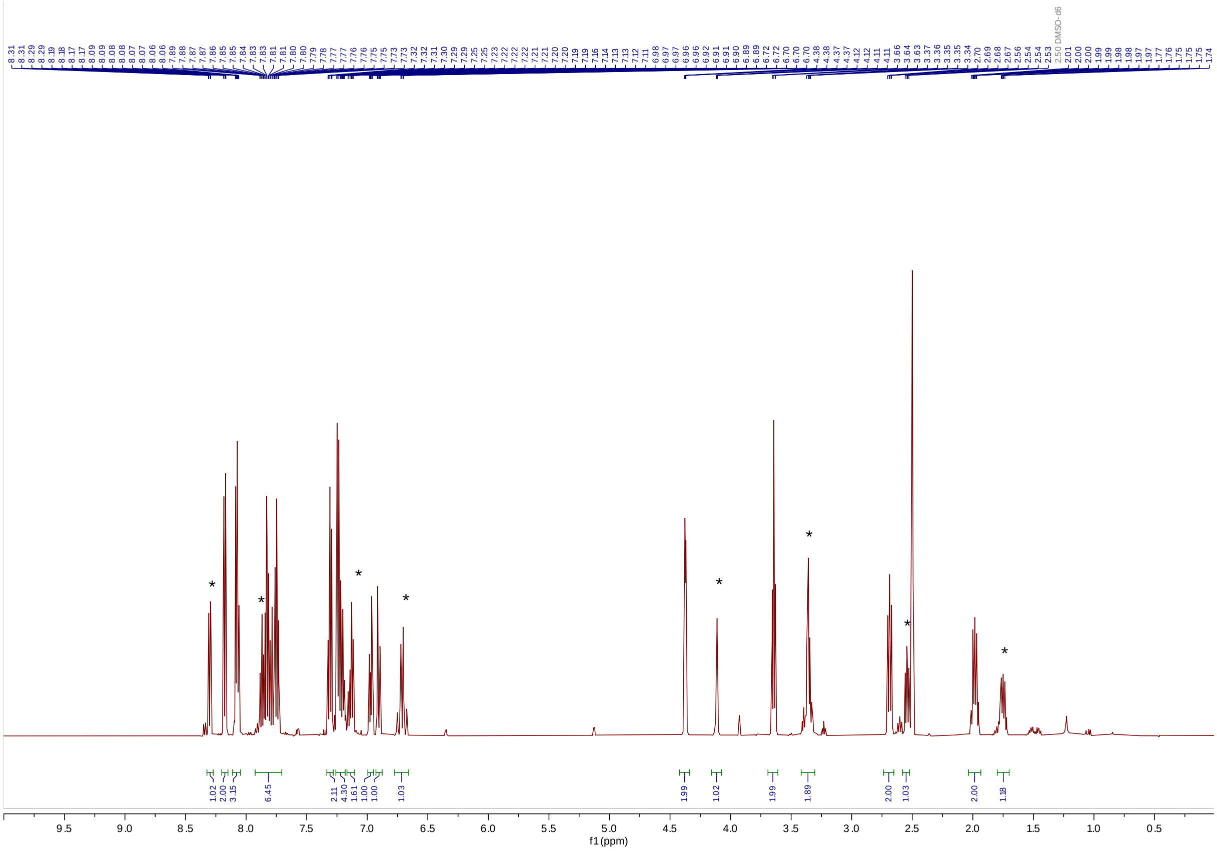

^{13}C-NMR (125.65 MHz, DMSO-d^6) of **2g** (see Procedure)

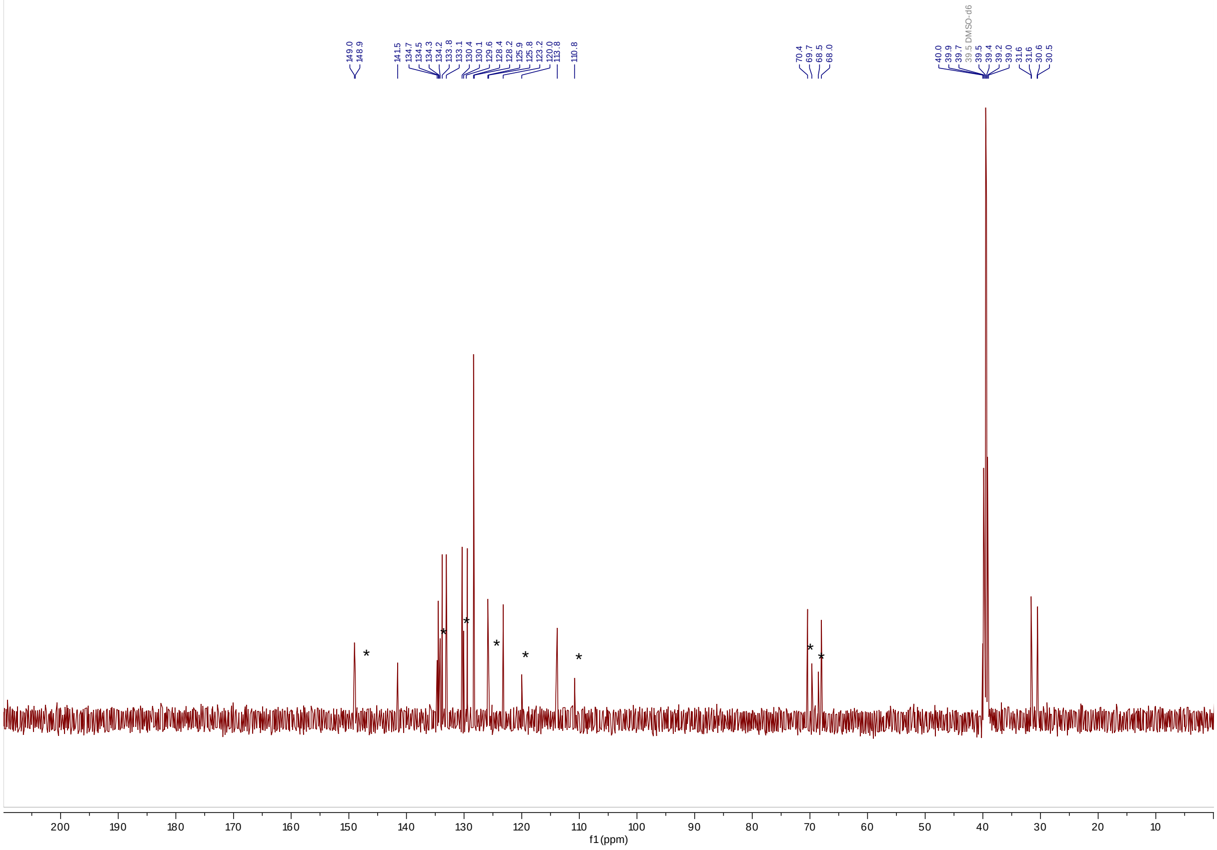

^{19}F-NMR (282.21 MHz, DMSO-*d*[6]) of **2g**

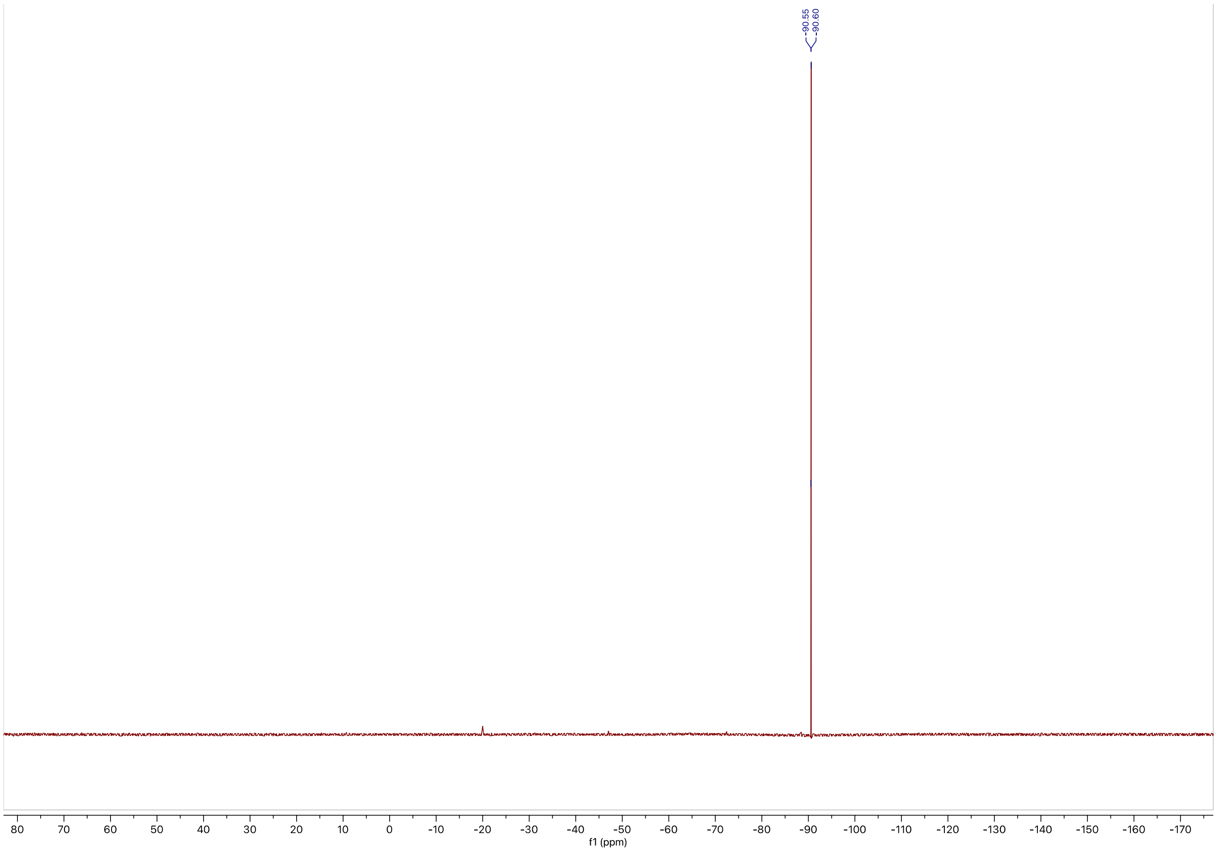

^{1}H-NMR (499.64 MHz, DMSO-d^6) of **2h** (see Procedure)

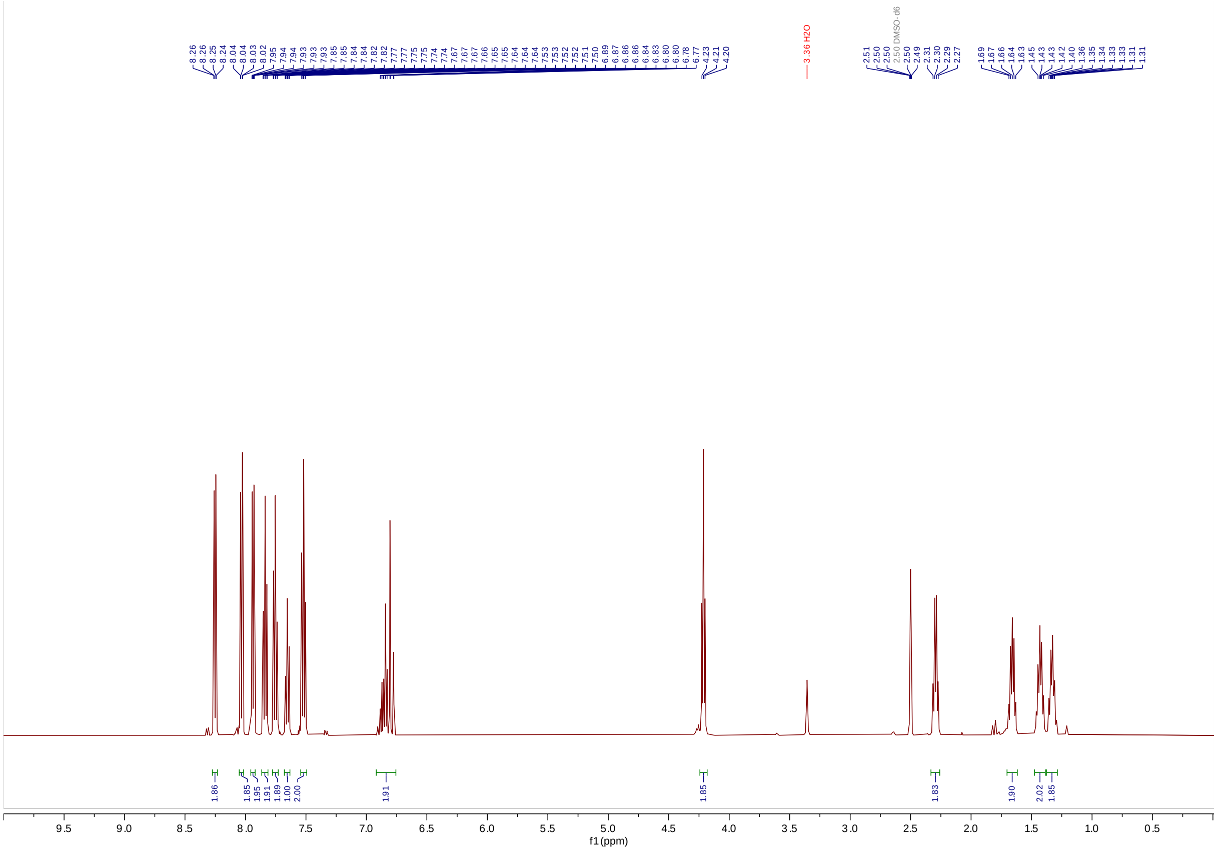

^{13}C-NMR (125.65 MHz, DMSO-d^6) of **2h** (see Procedure)

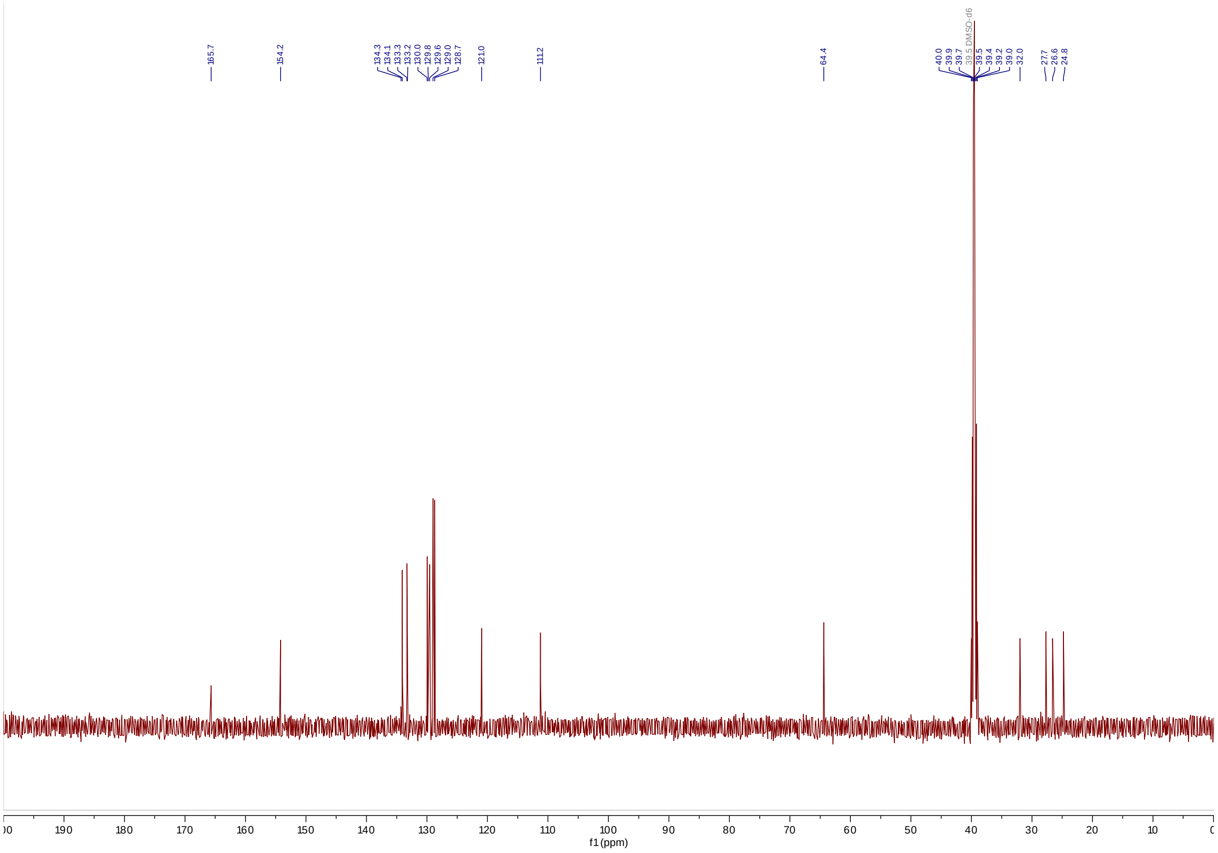

^{19}**F-NMR** (282.21 MHz, DMSO-d^6) of **2h**

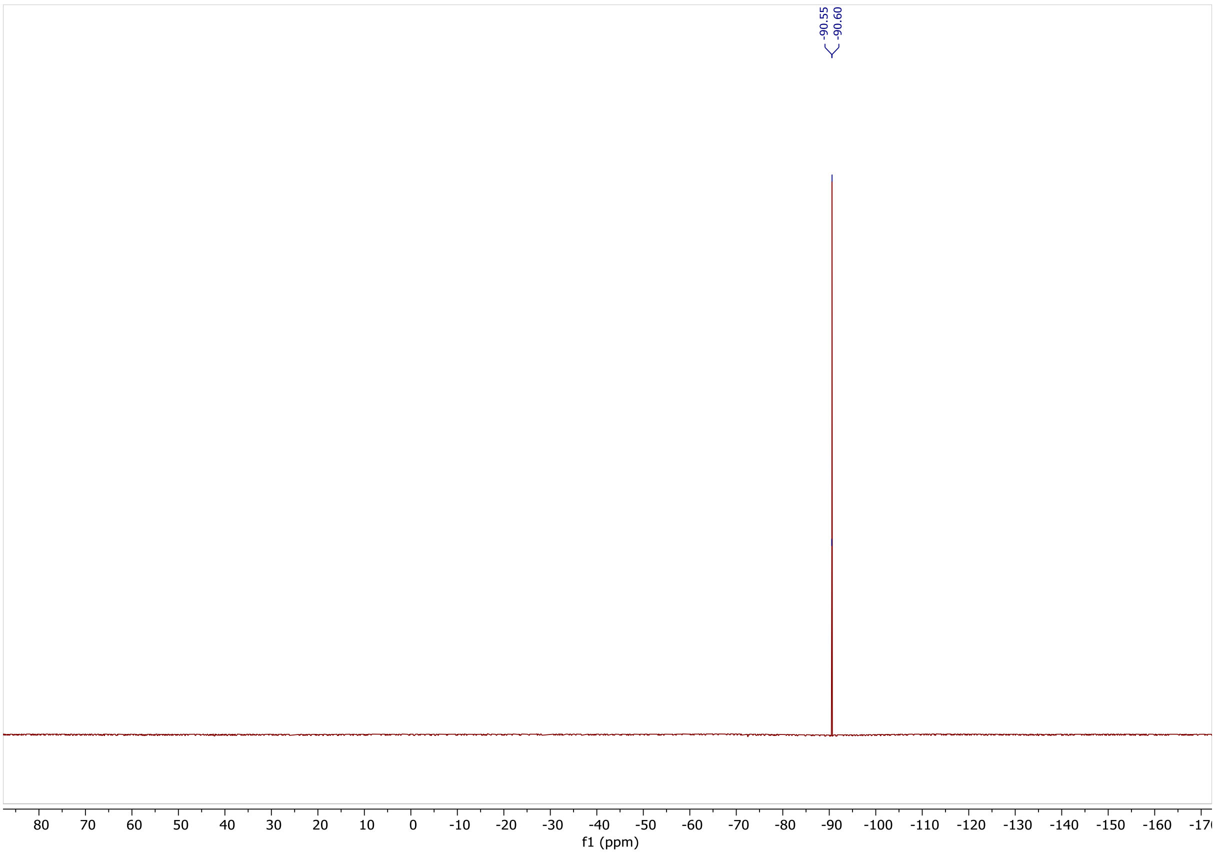

^{1}H-NMR (499.64 MHz, DMSO-d^6) of **2i** (see Procedure)

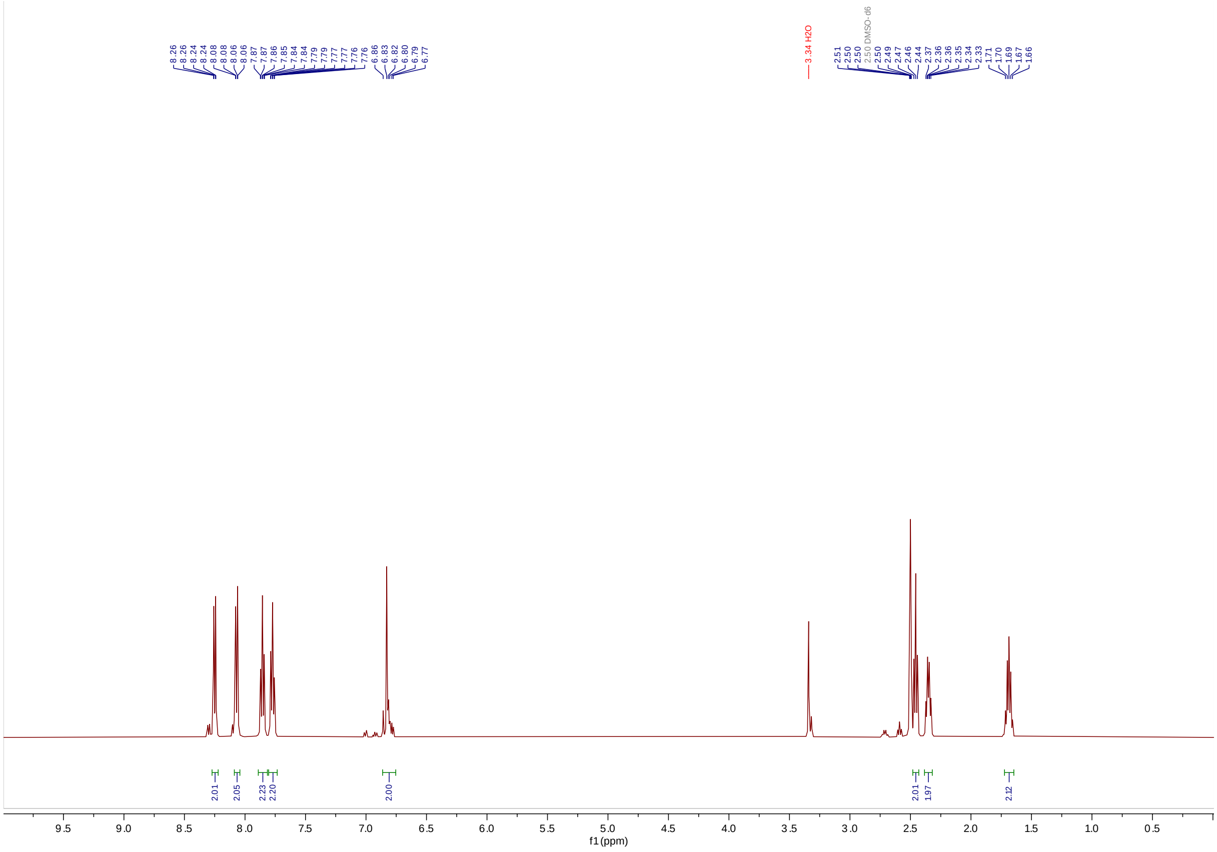

^{13}C-NMR (125.65 MHz, DMSO-d^6) of **2i** (see Procedure)

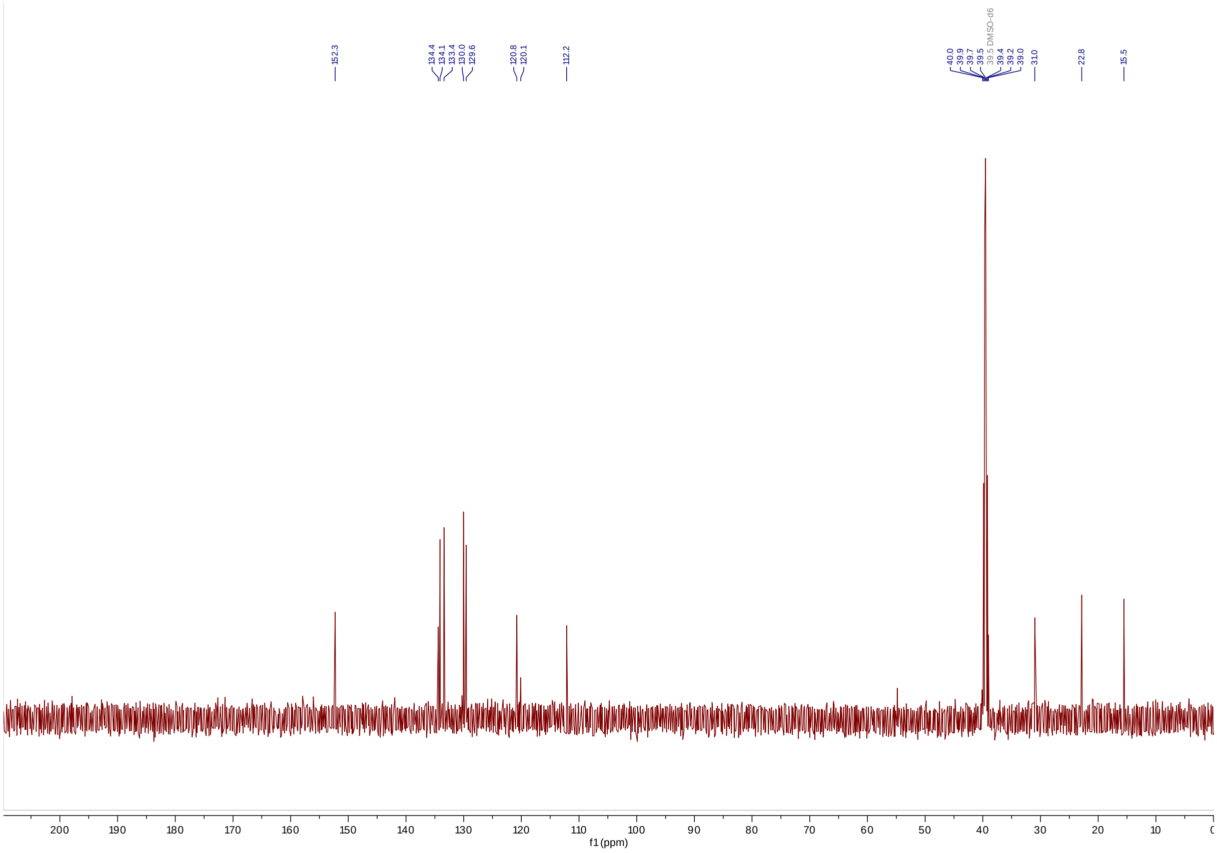

^{19}F-NMR (282.21 MHz, DMSO-*d*[6]) of **2i**

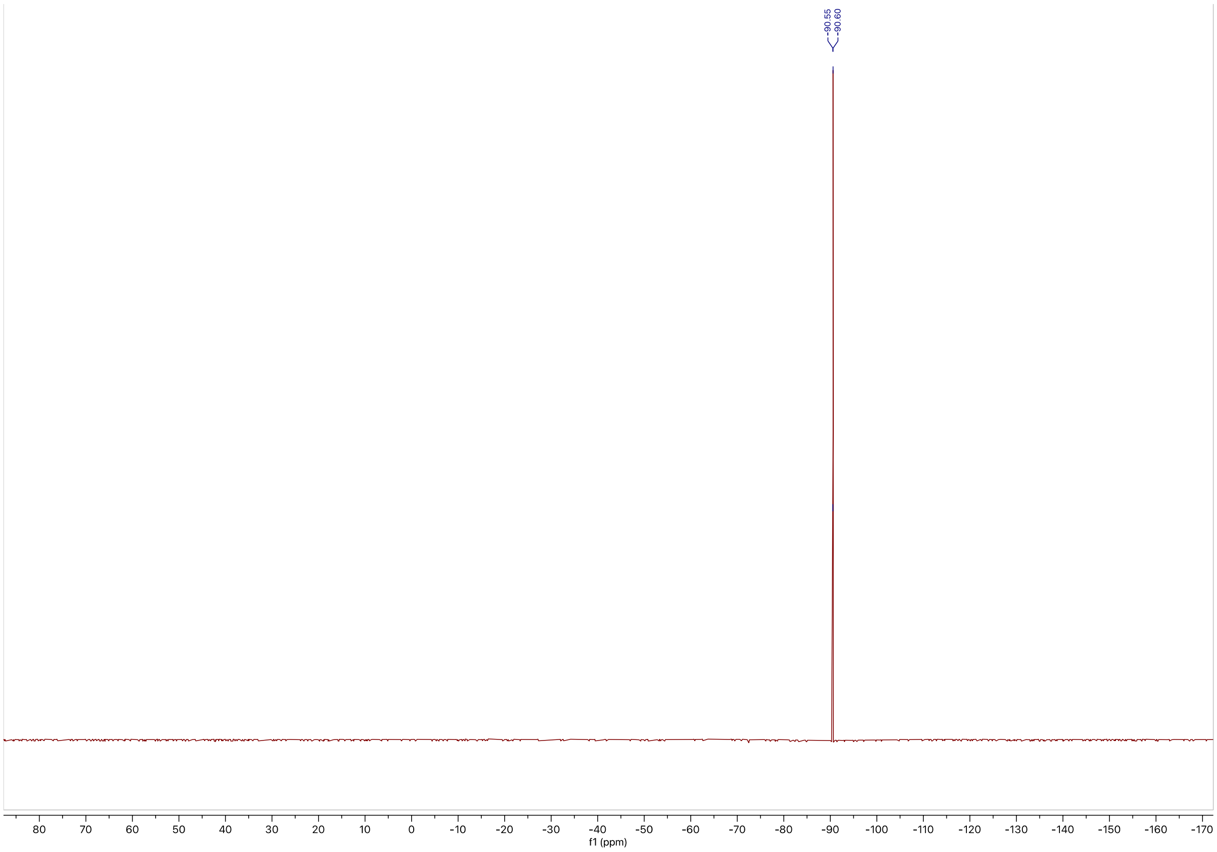

^{1}H-NMR (499.64 MHz, DMSO-d^6) of **2j** (see Procedure)

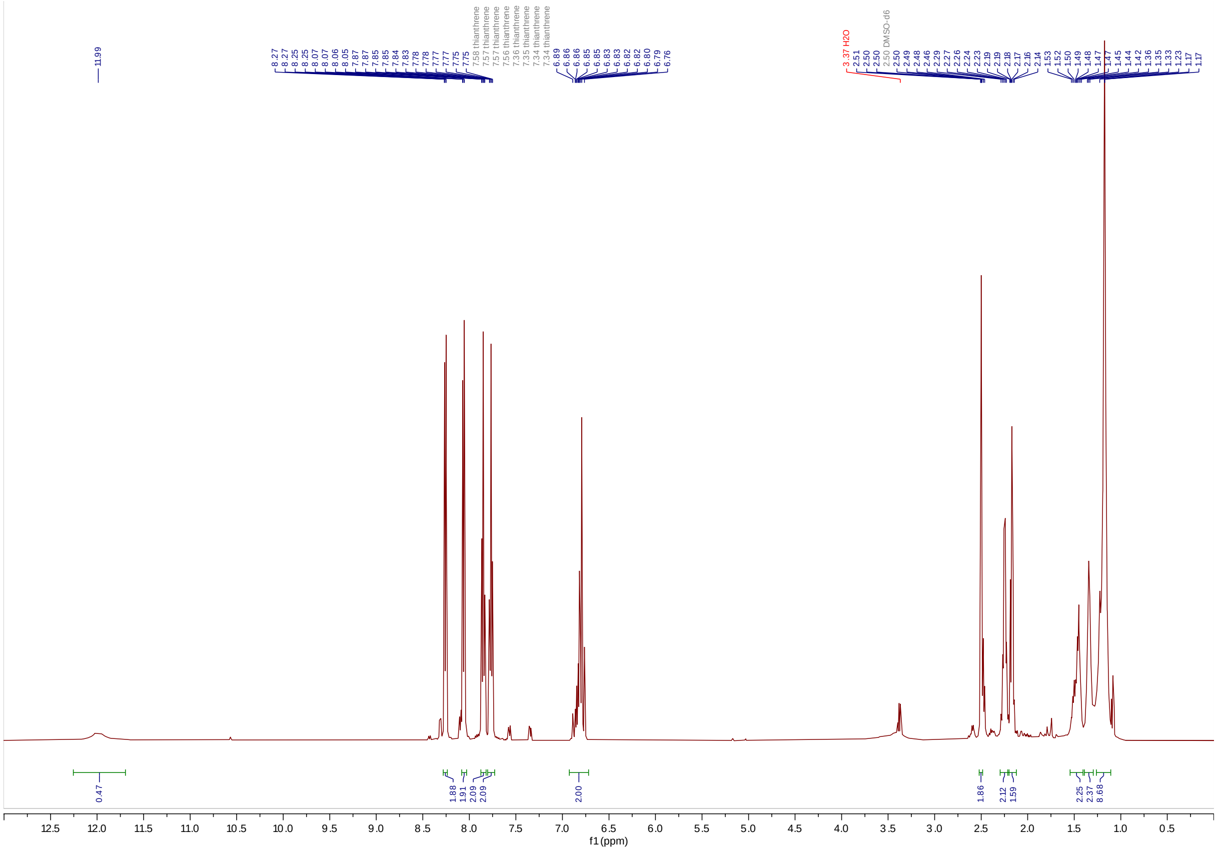

^{13}C-NMR (125.65 MHz, DMSO-d^6) of **2j** (see Procedure)

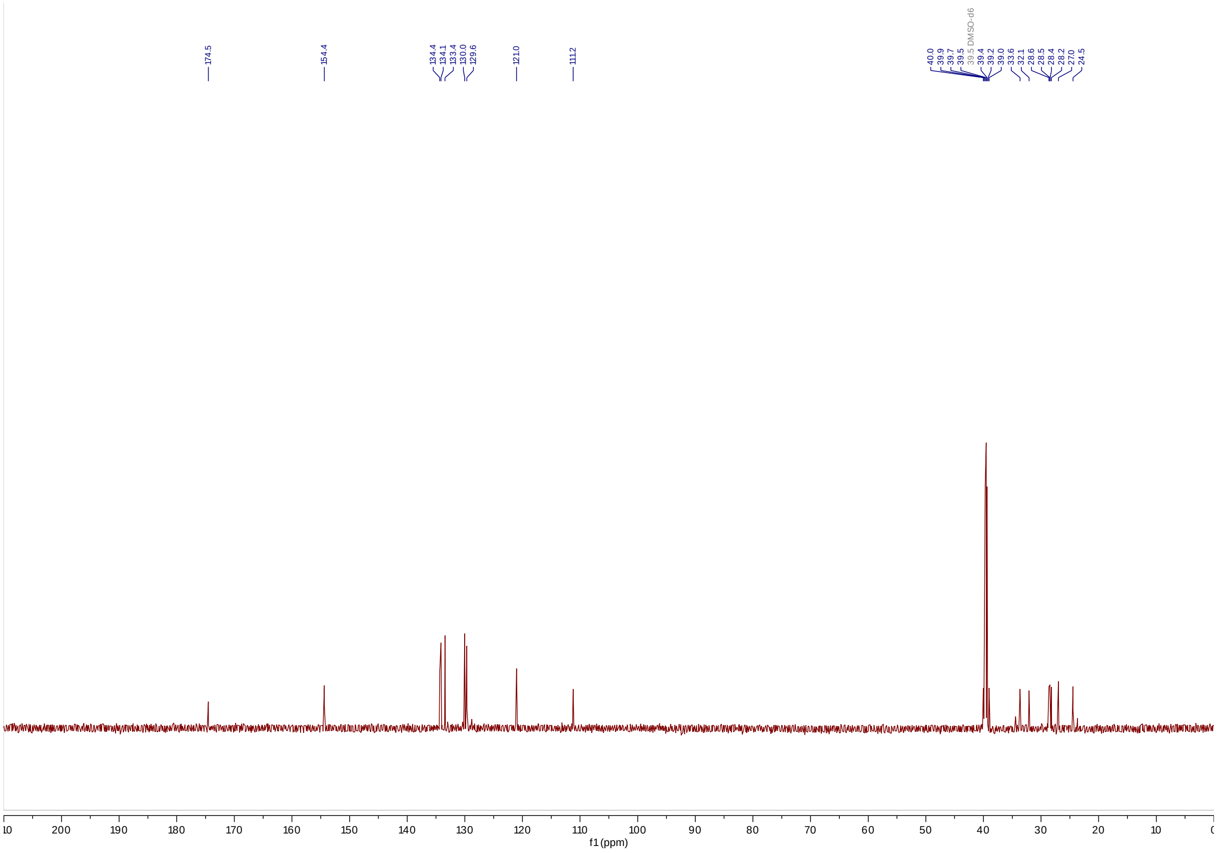

^{19}F-NMR (282.21 MHz, DMSO-*d*^{6}) of **2j**

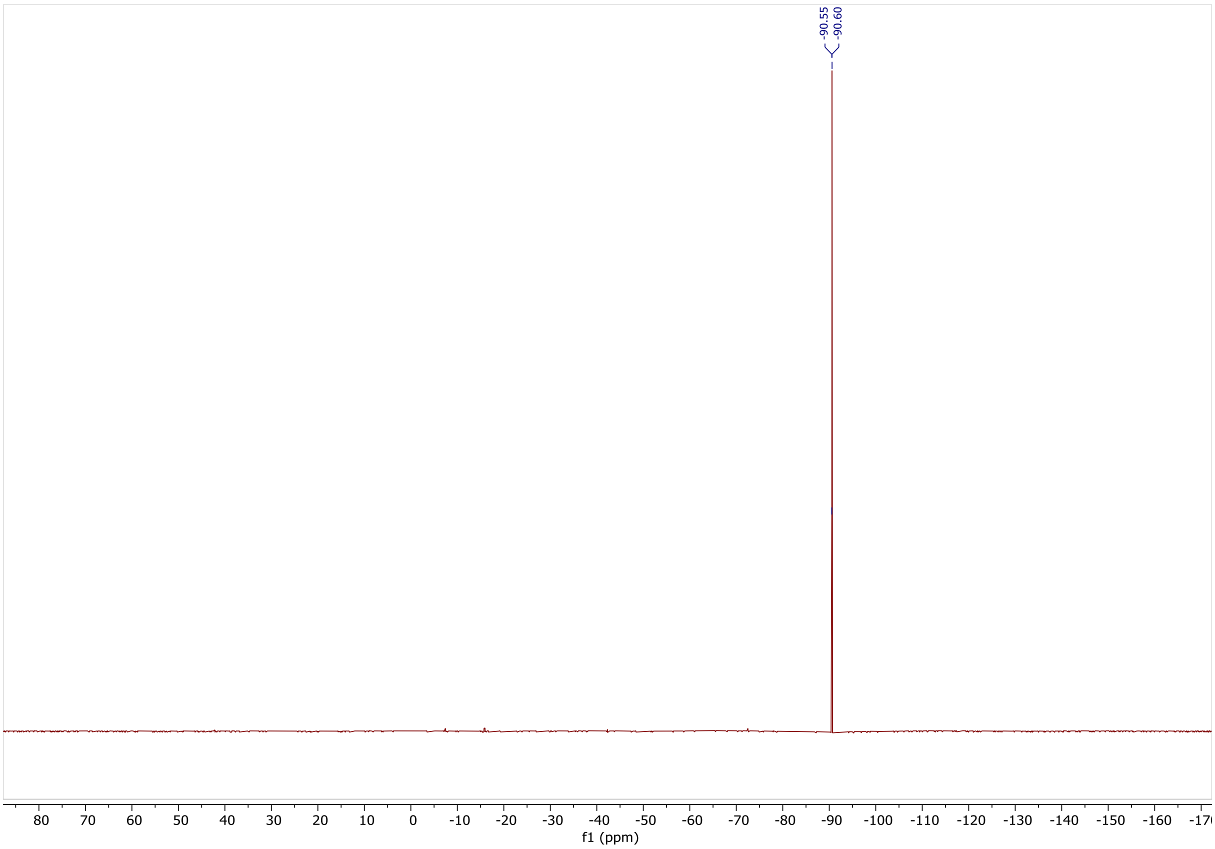

¹H-NMR (499.64 MHz, DMSO-d^6) of **2l** (see Procedure)

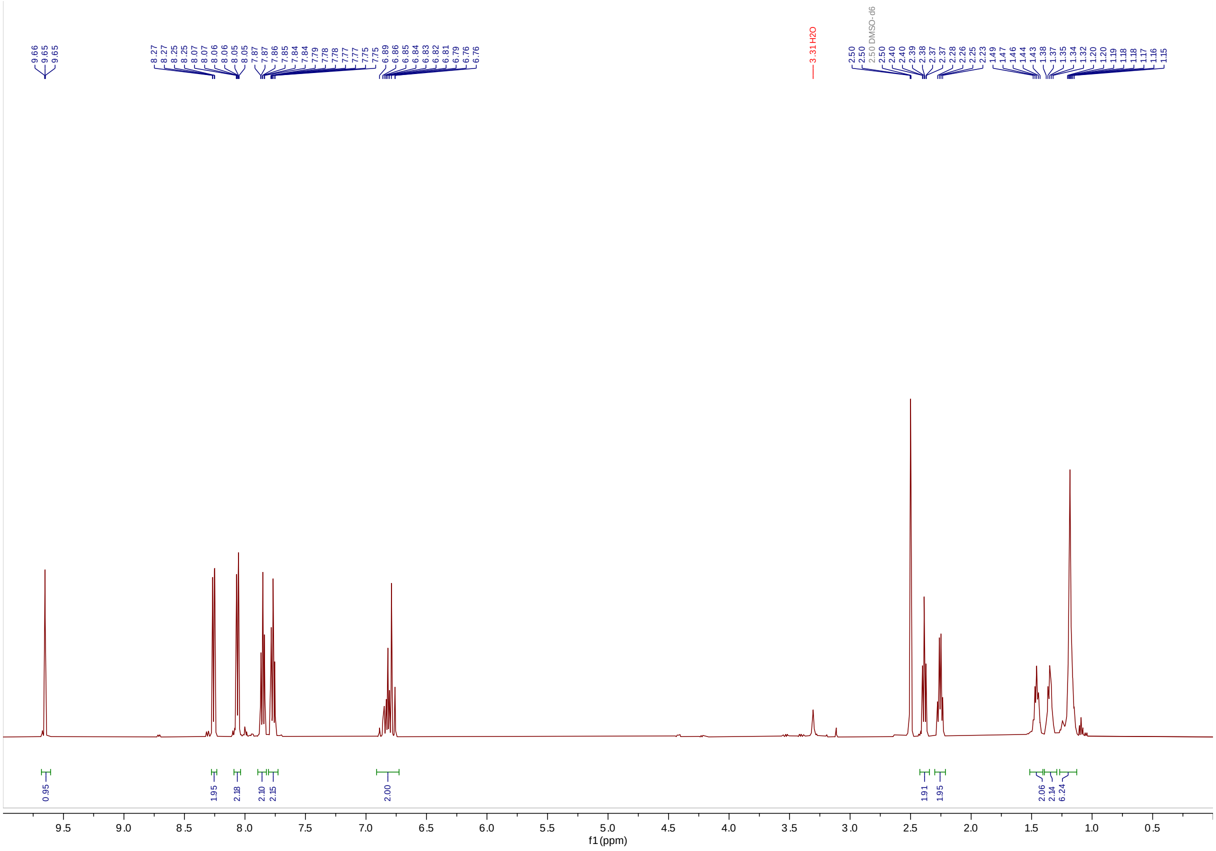

¹³C-NMR (125.65 MHz, DMSO-d^6) of **2l** (see Procedure)

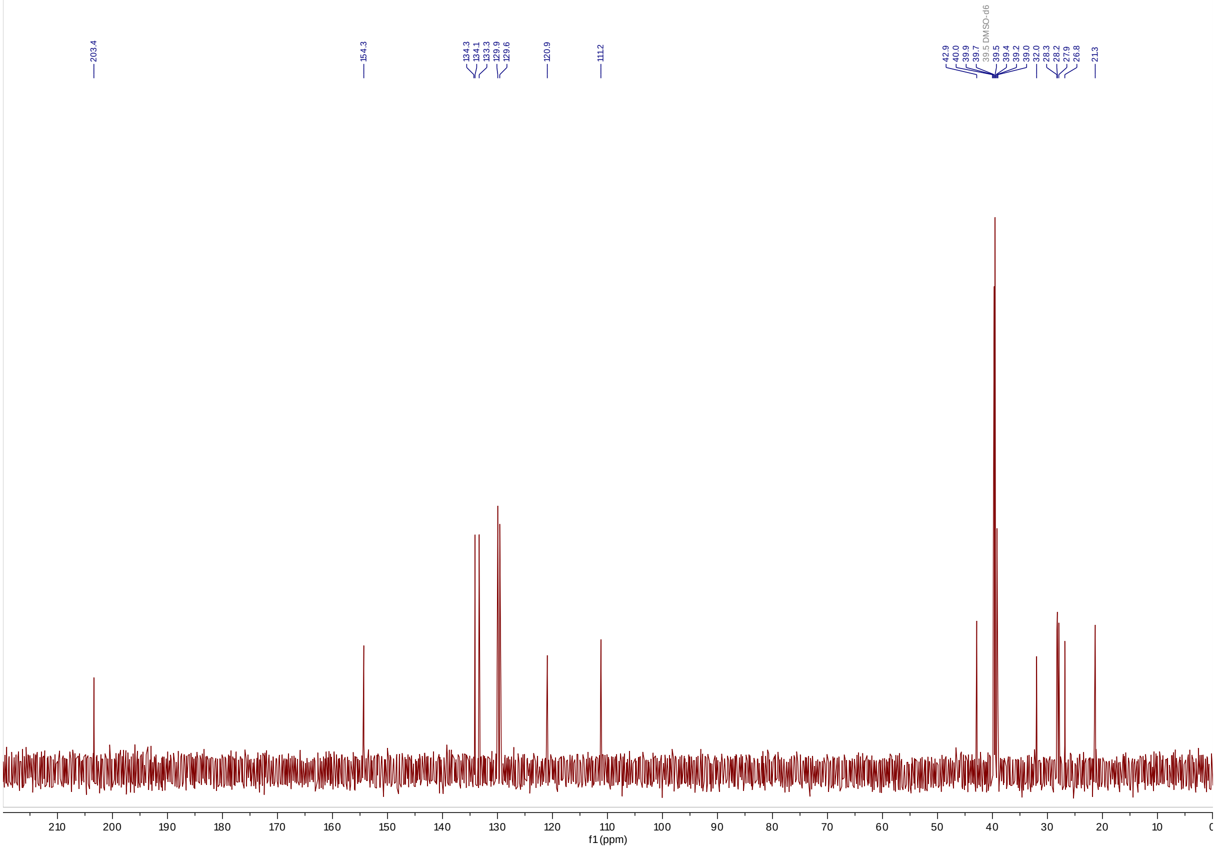

^{19}**F-NMR** (282.21 MHz, DMSO-d^6) of **2l**

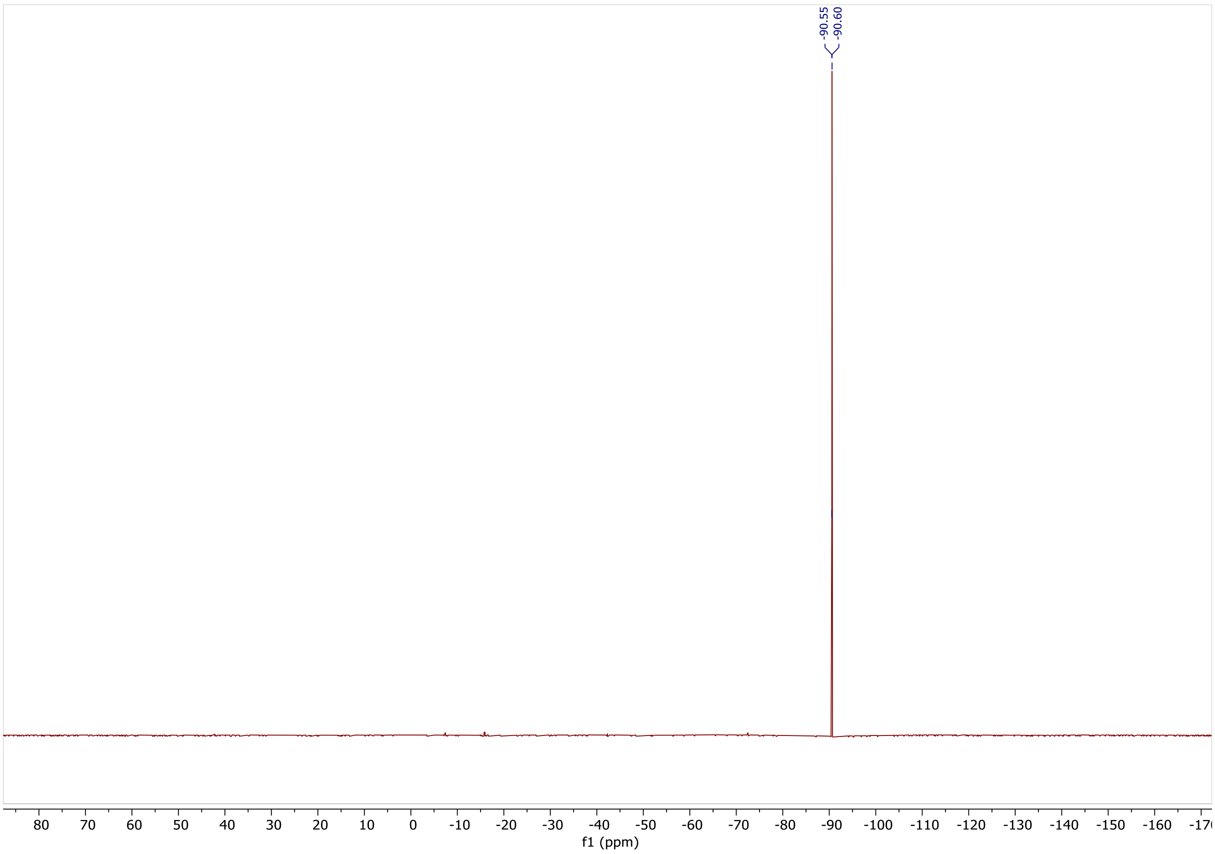

^{1}H-NMR (499.64 MHz, DMSO-d^{6}) of **2m** (see Procedure)

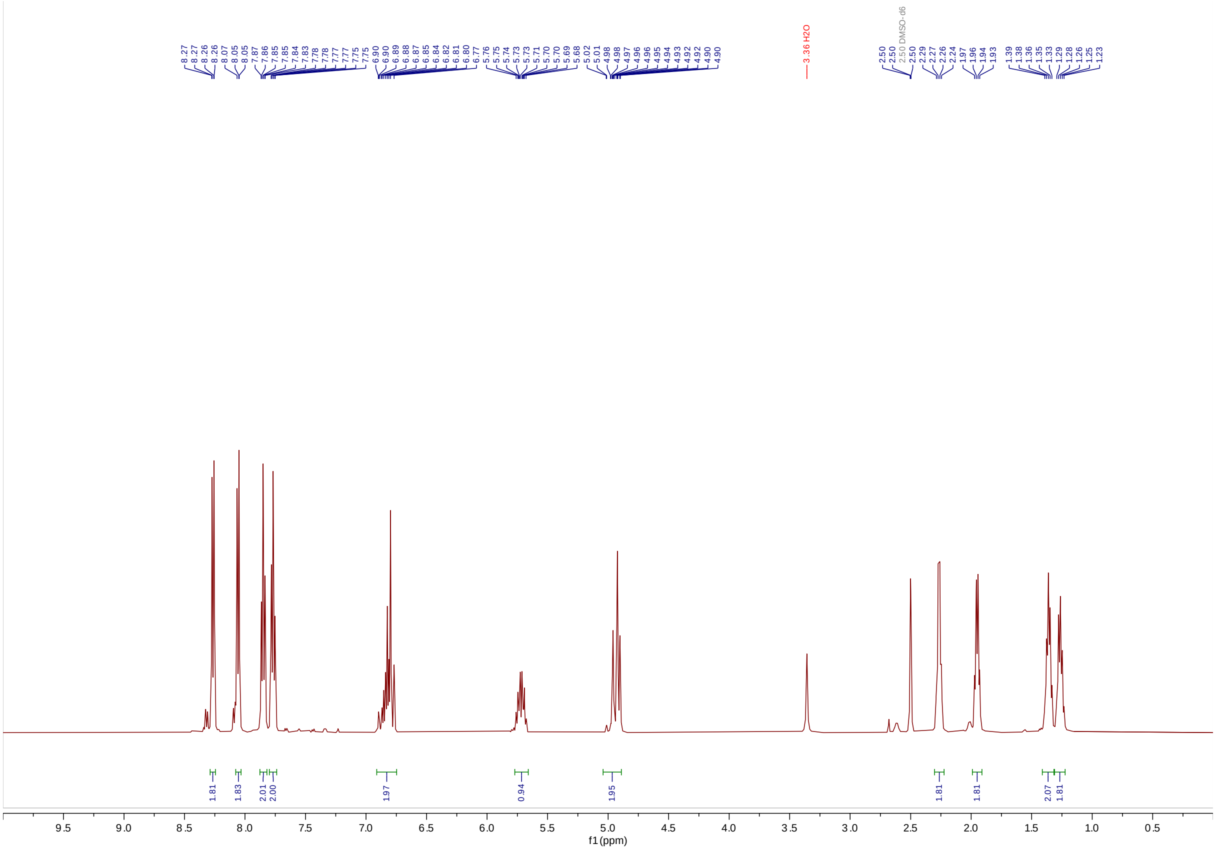

^{13}C-NMR (125.65 MHz, DMSO-d^{6}) of **2m** (see Procedure)

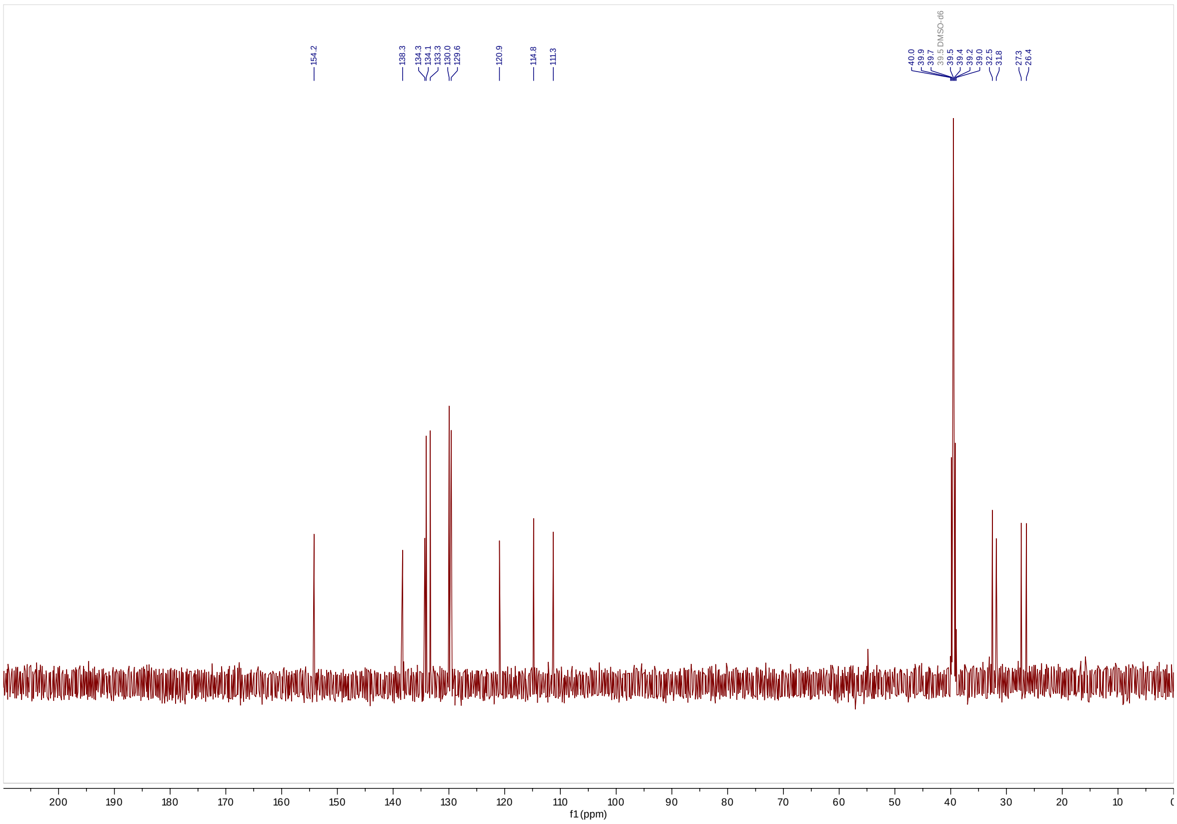

^{19}F-NMR (282.21 MHz, DMSO-d^6) of **2m**

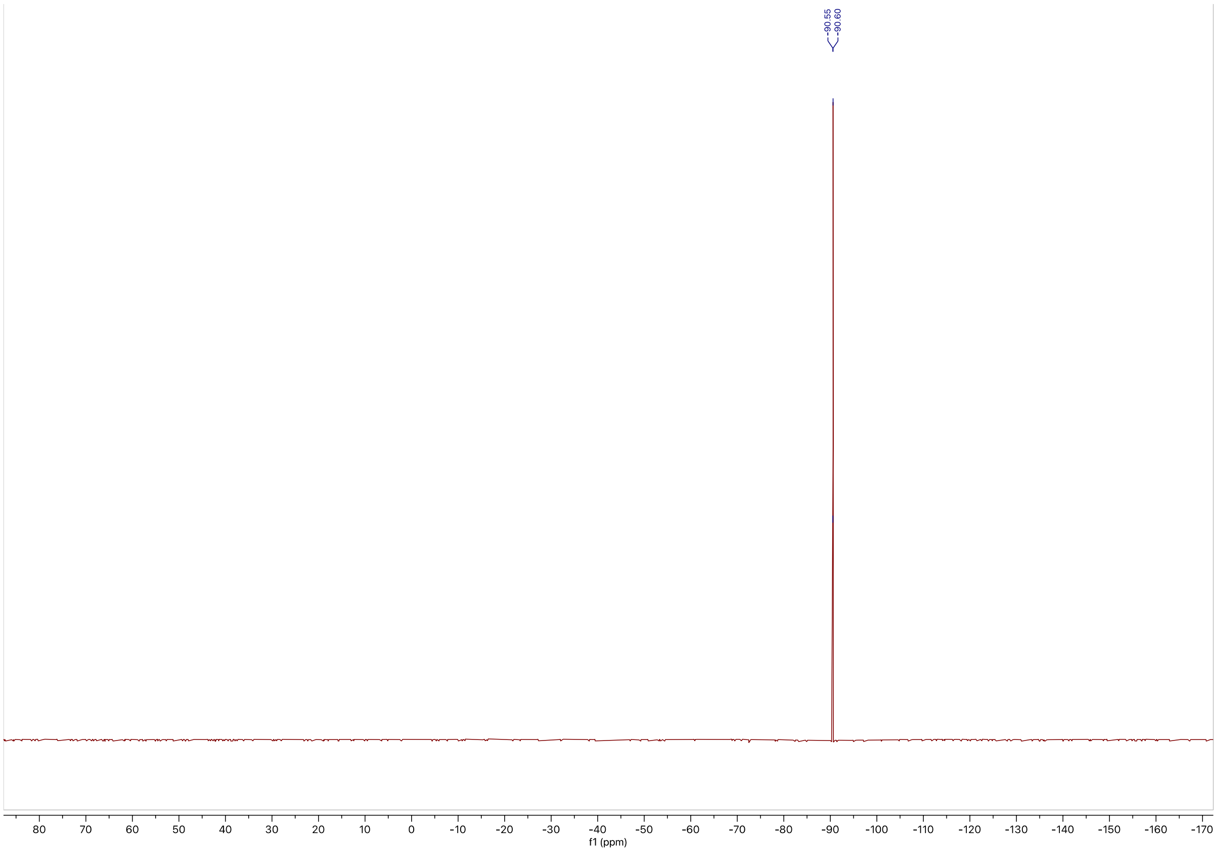

^{1}H-NMR (499.64 MHz, DMSO-d^6) of **2n** (see Procedure)

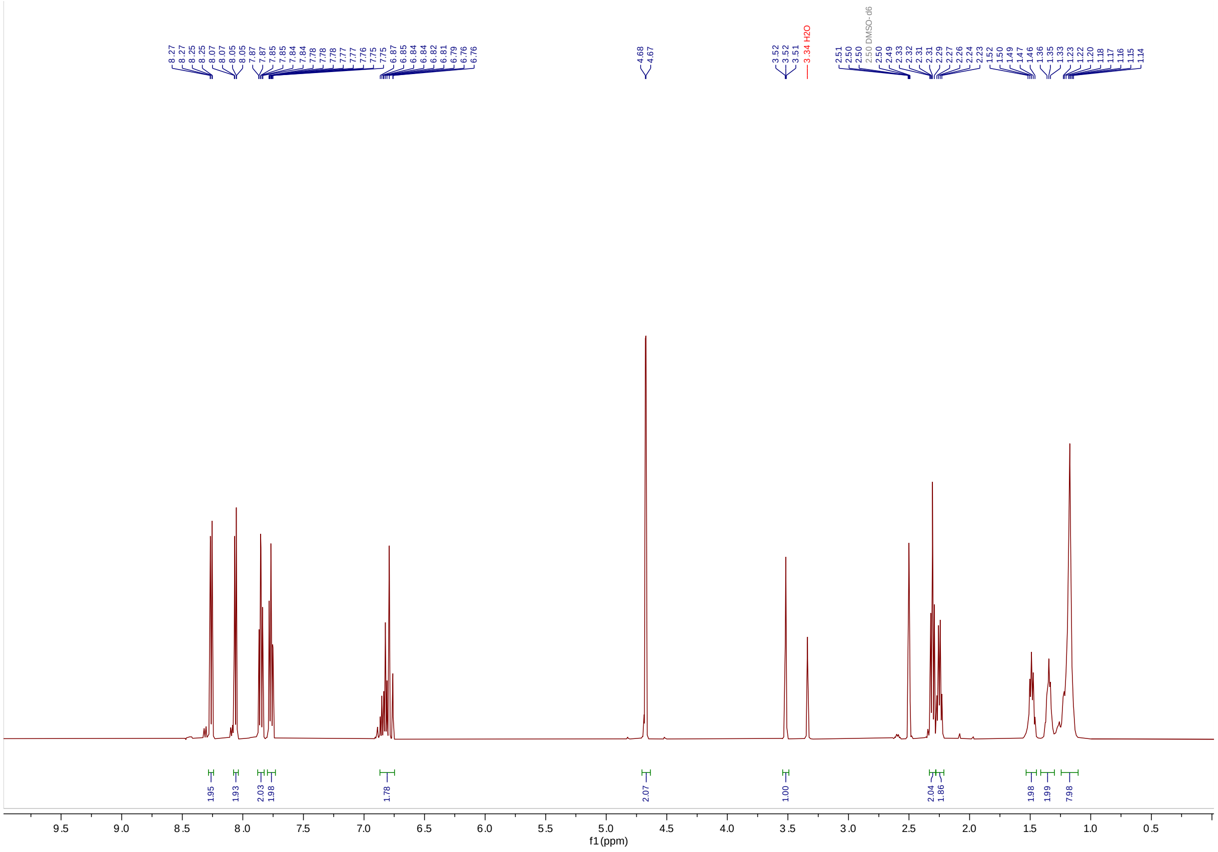

^{13}C-NMR (125.65 MHz, DMSO-d^6) of **2n** (see Procedure)

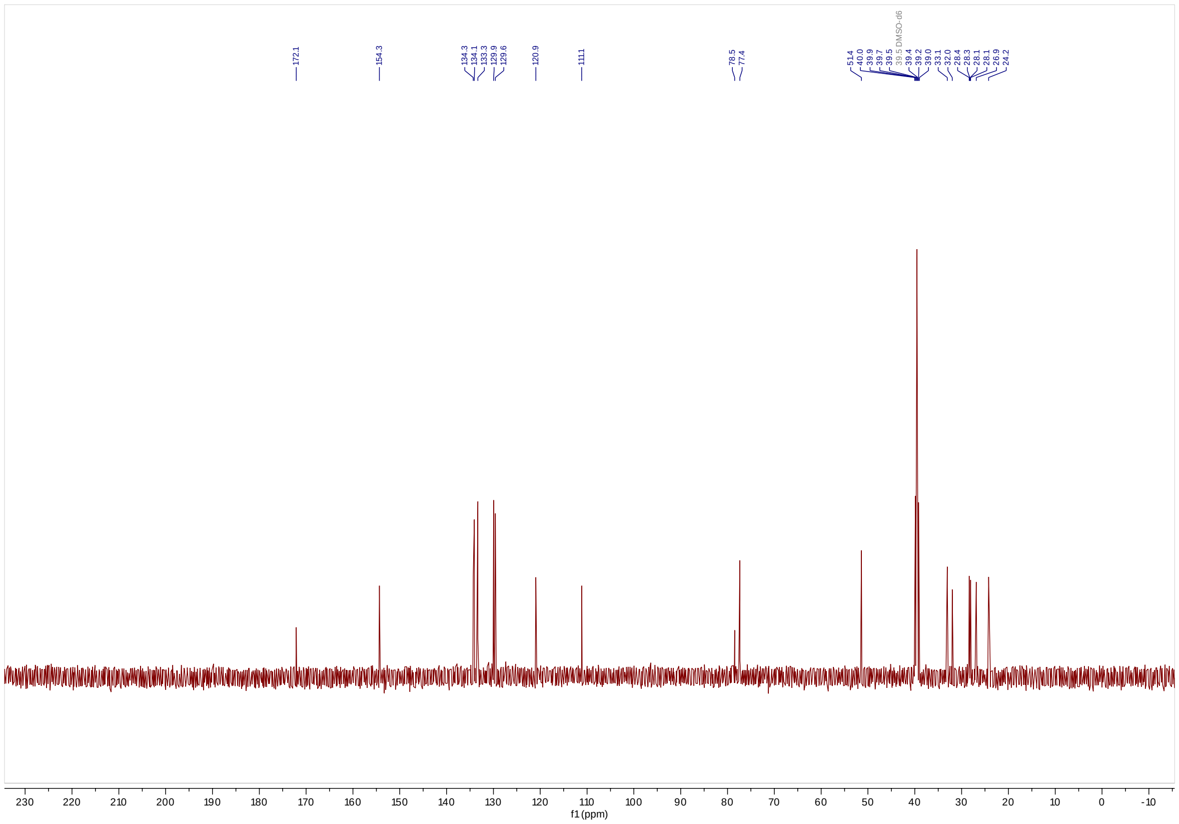

^{19}F-NMR (282.21 MHz, DMSO-d^6) of **2n**

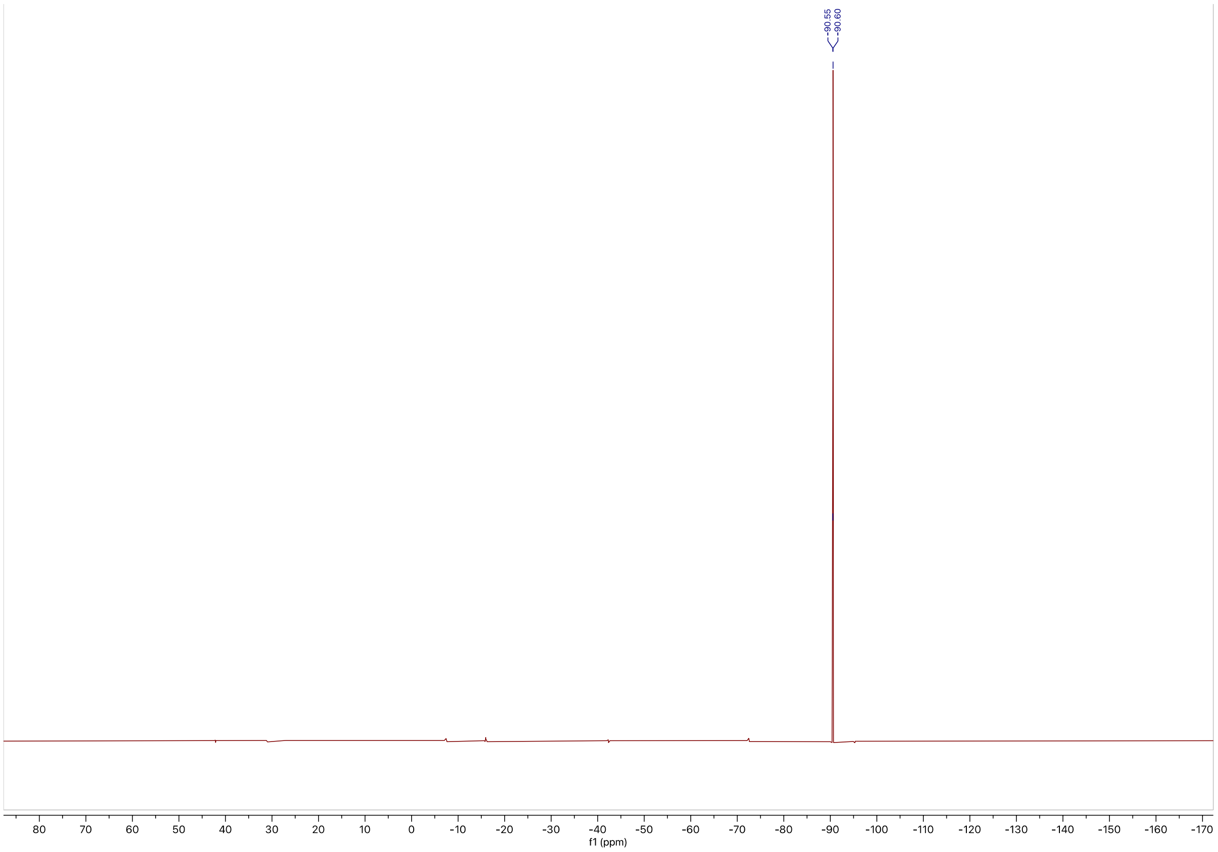

[1]**H-NMR** (499.64 MHz, DMSO-d^6) of **2o** (see Procedure)

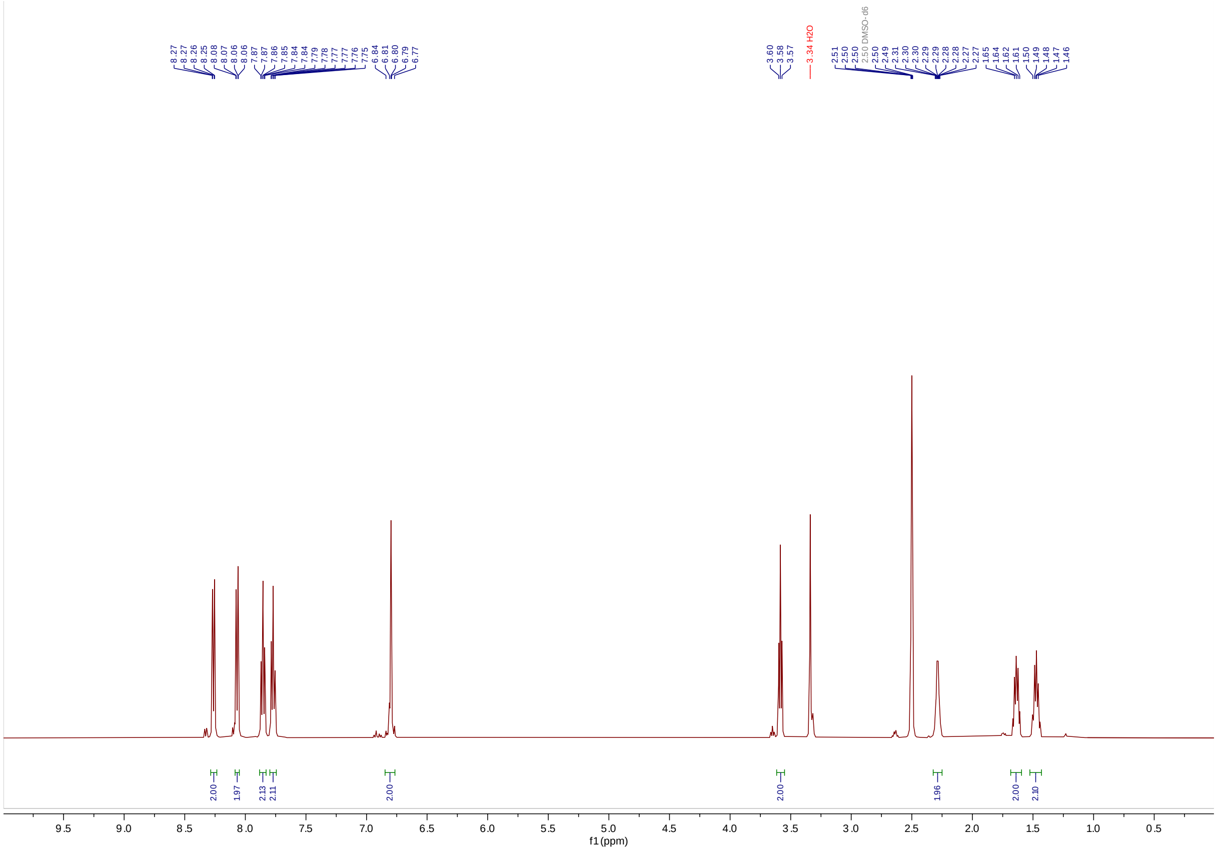

[13]**C-NMR** (125.65 MHz, DMSO-d^6) of **2o** (see Procedure)

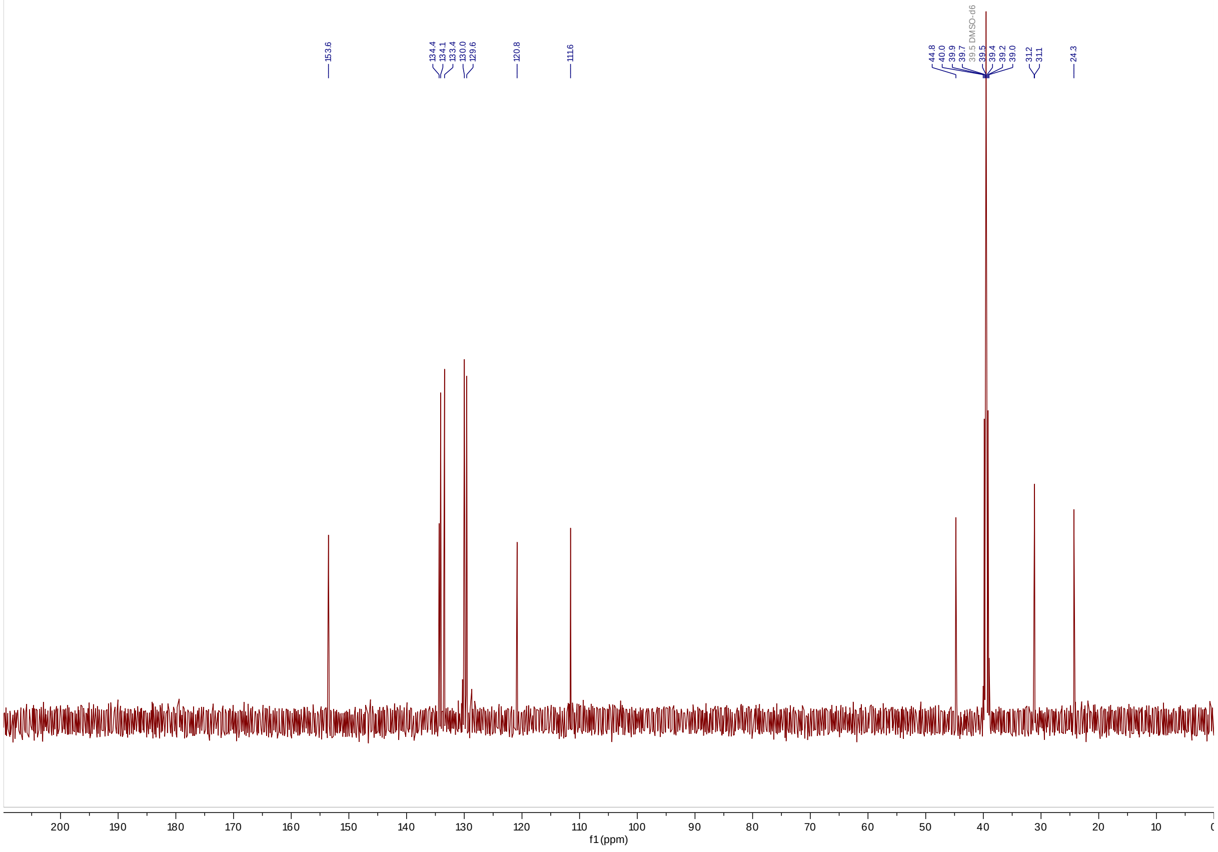

^{19}F-NMR (282.21 MHz, DMSO-d^6) of **2o**

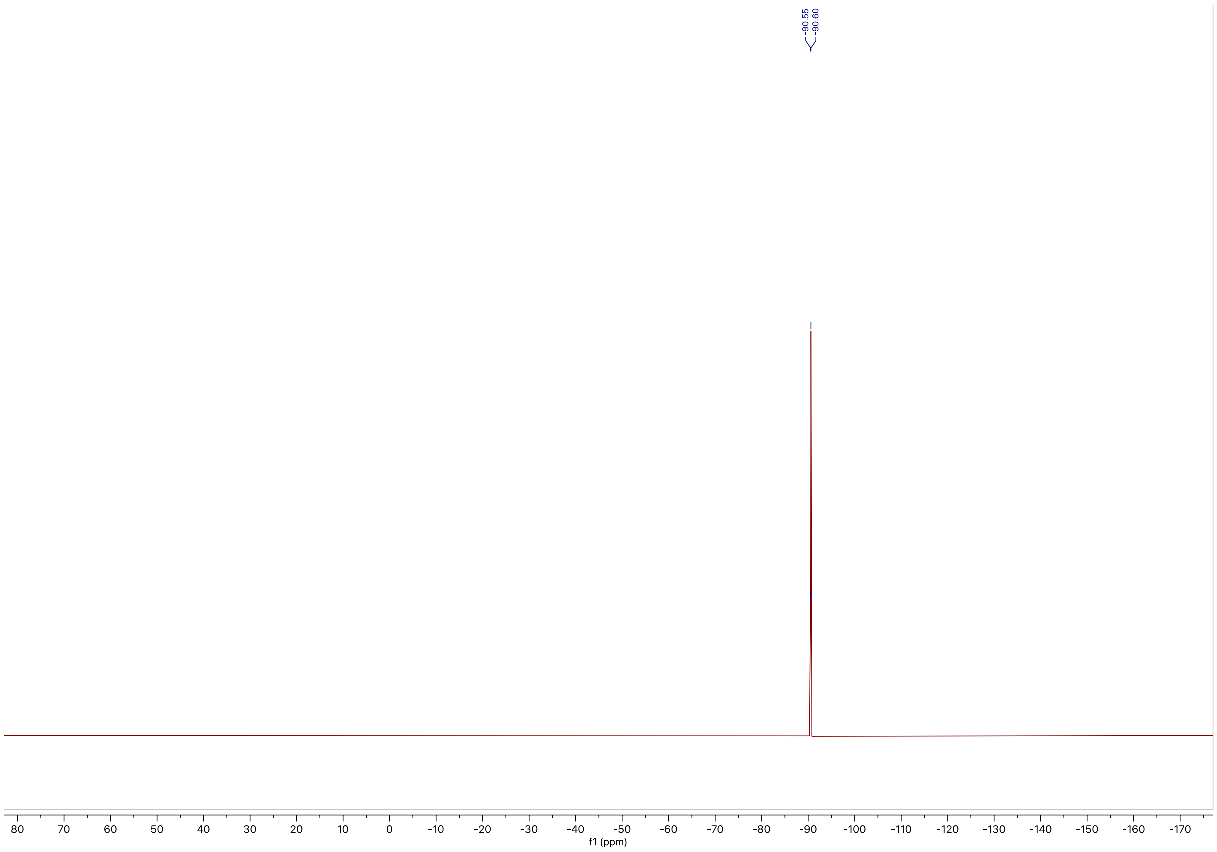

¹H-NMR (499.64 MHz, DMSO-d^6) of **2q** (see Procedure)

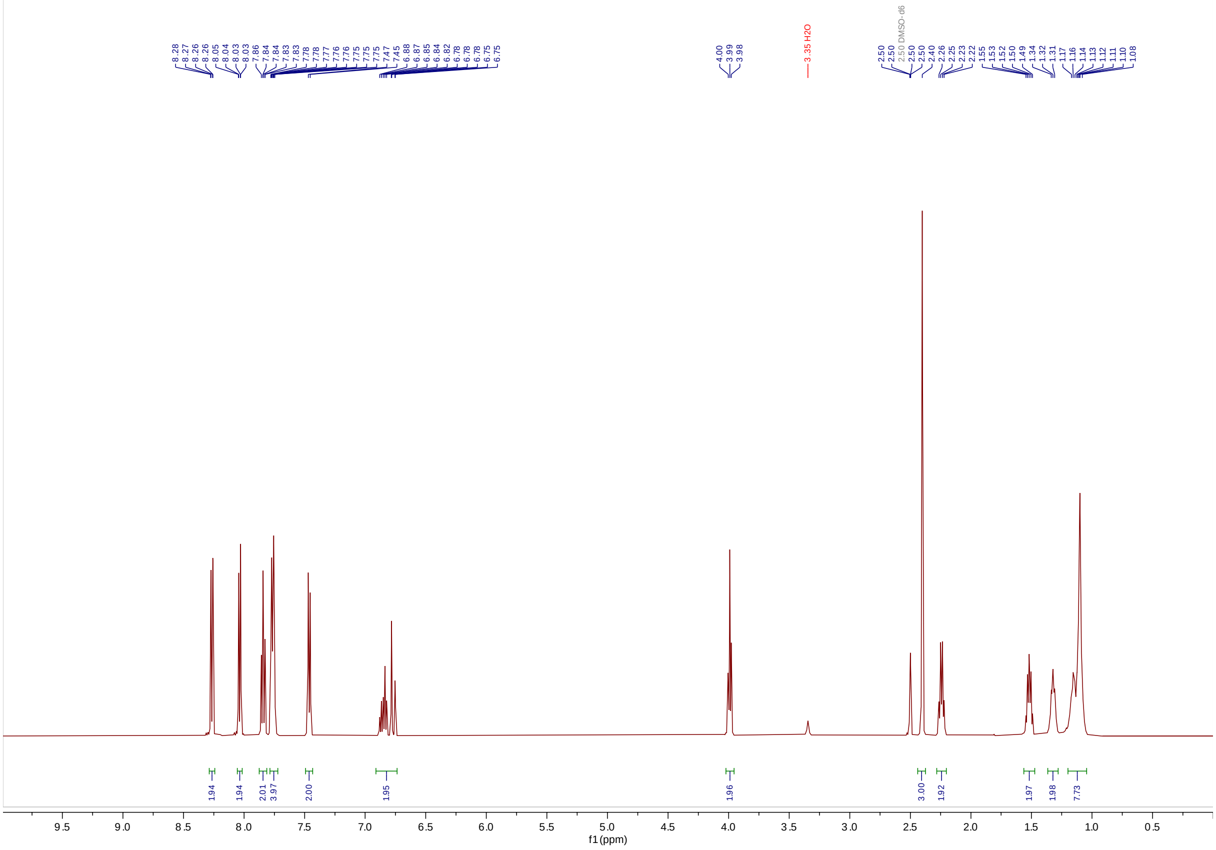

¹³C-NMR (125.65 MHz, DMSO-d^6) of **2q** (see Procedure)

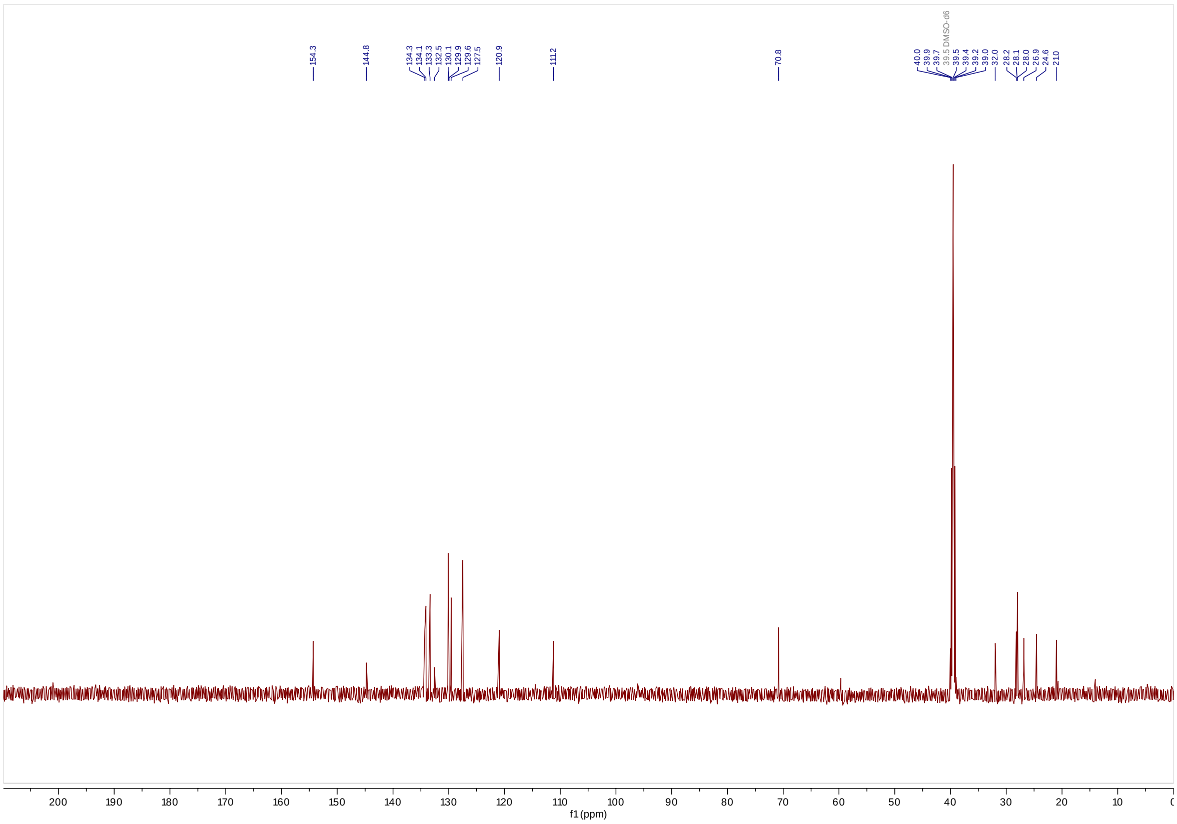

^{19}F-NMR (282.21 MHz, DMSO-d^6) of **2q**

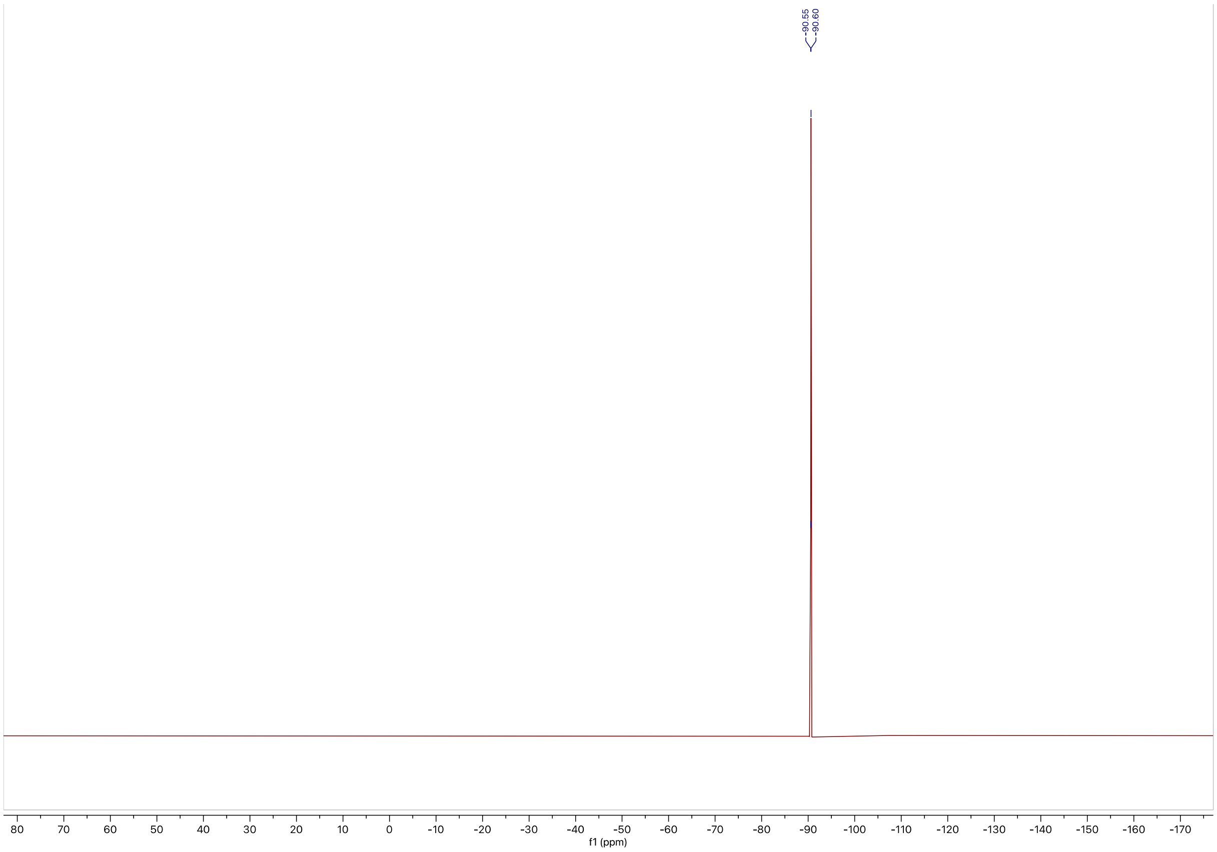

^{1}H-NMR (499.64 MHz, DMSO-*d^6*) of **2v** (see Procedure)

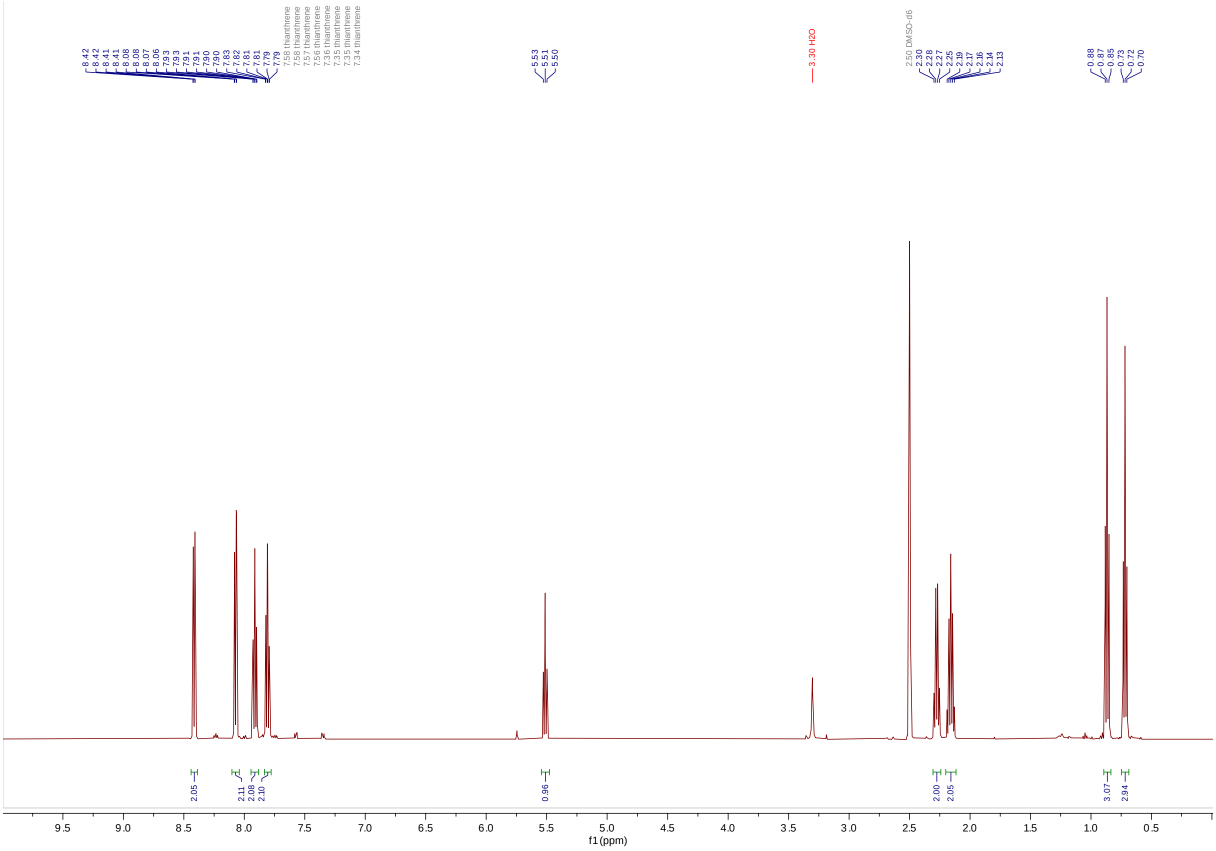

^{13}C-NMR (125.65 MHz, DMSO-*d^6*) of **2v** (see Procedure)

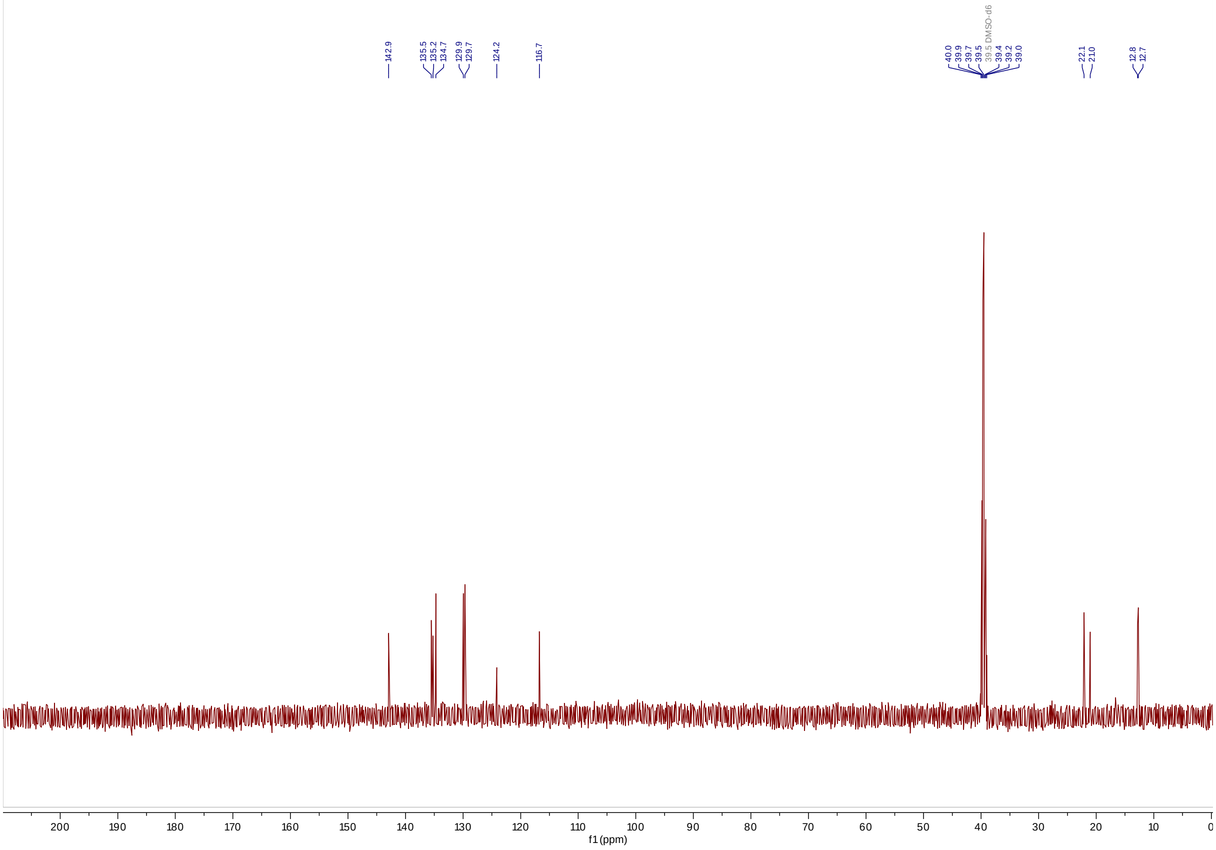

^{19}F-NMR (282.21 MHz, DMSO-d^6) of **2v**

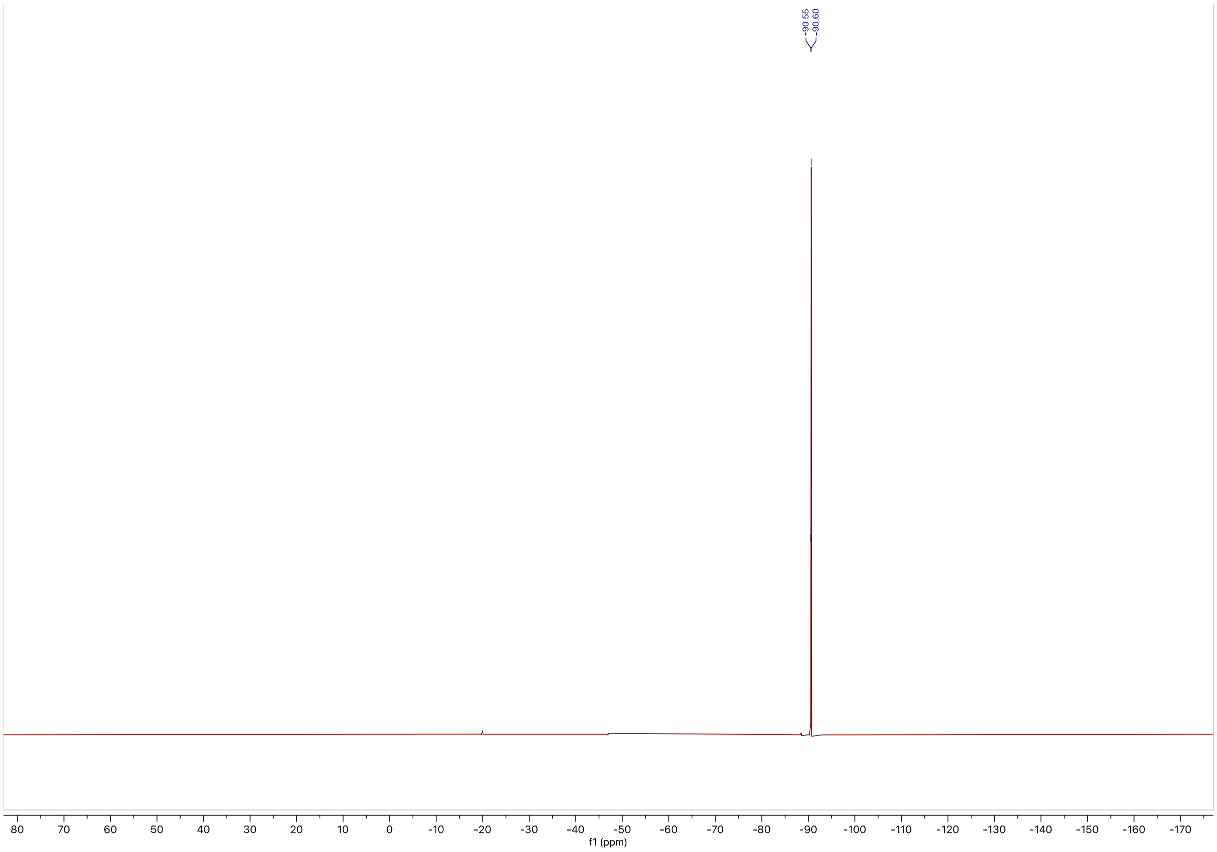

^{1}H-NMR (499.64 MHz, $CDCl_3$) of **3a** (see Procedure)

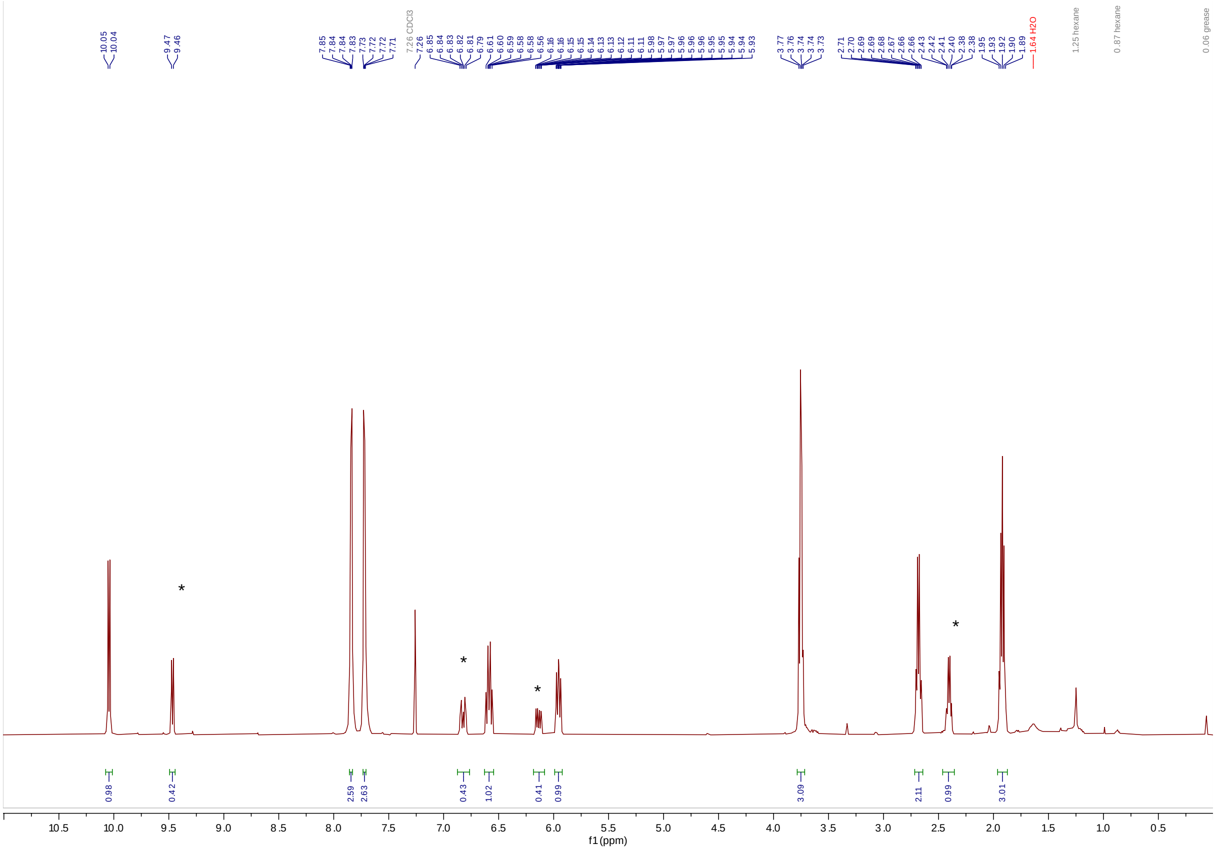

^{13}C-NMR (125.65 MHz, $CDCl_3$) of **3a** (see Procedure)

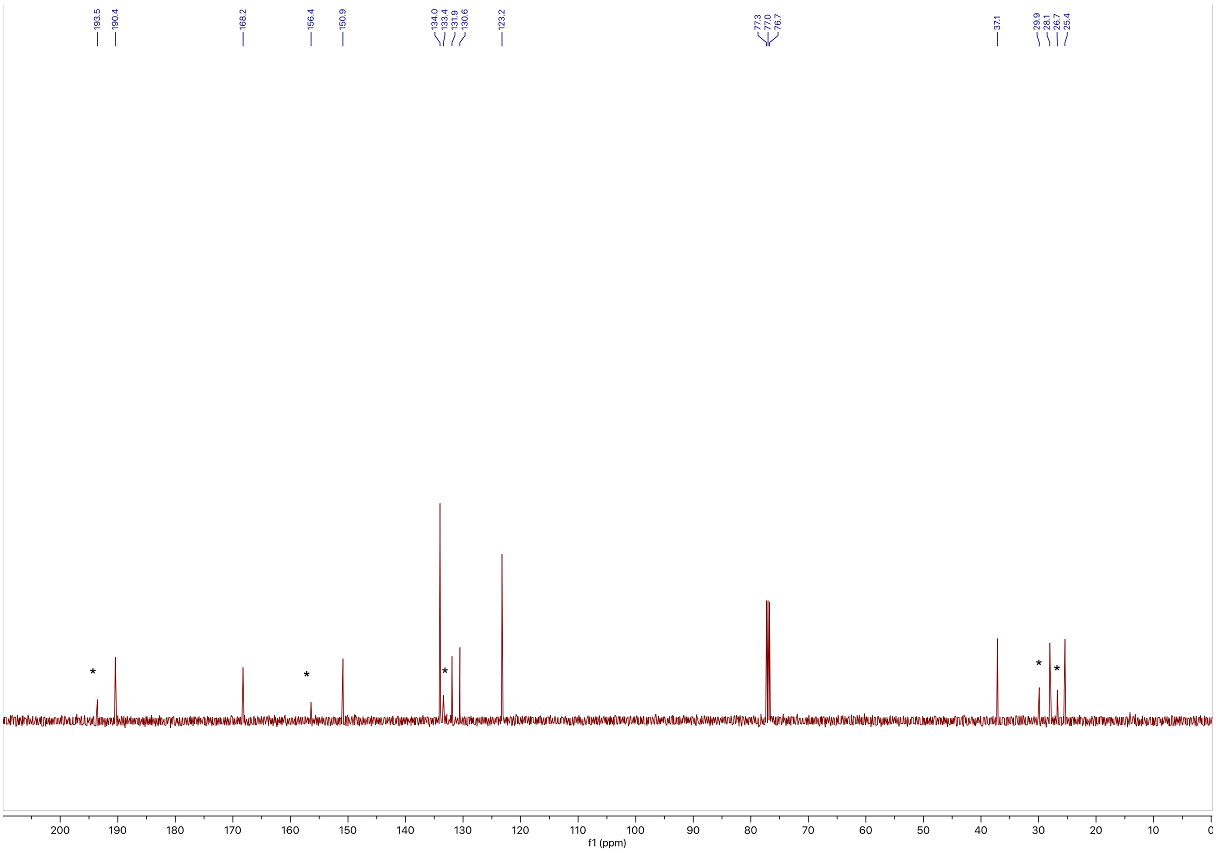

^{1}H-NMR (499.64 MHz, $CDCl_3$) of **3b** (see Procedure)

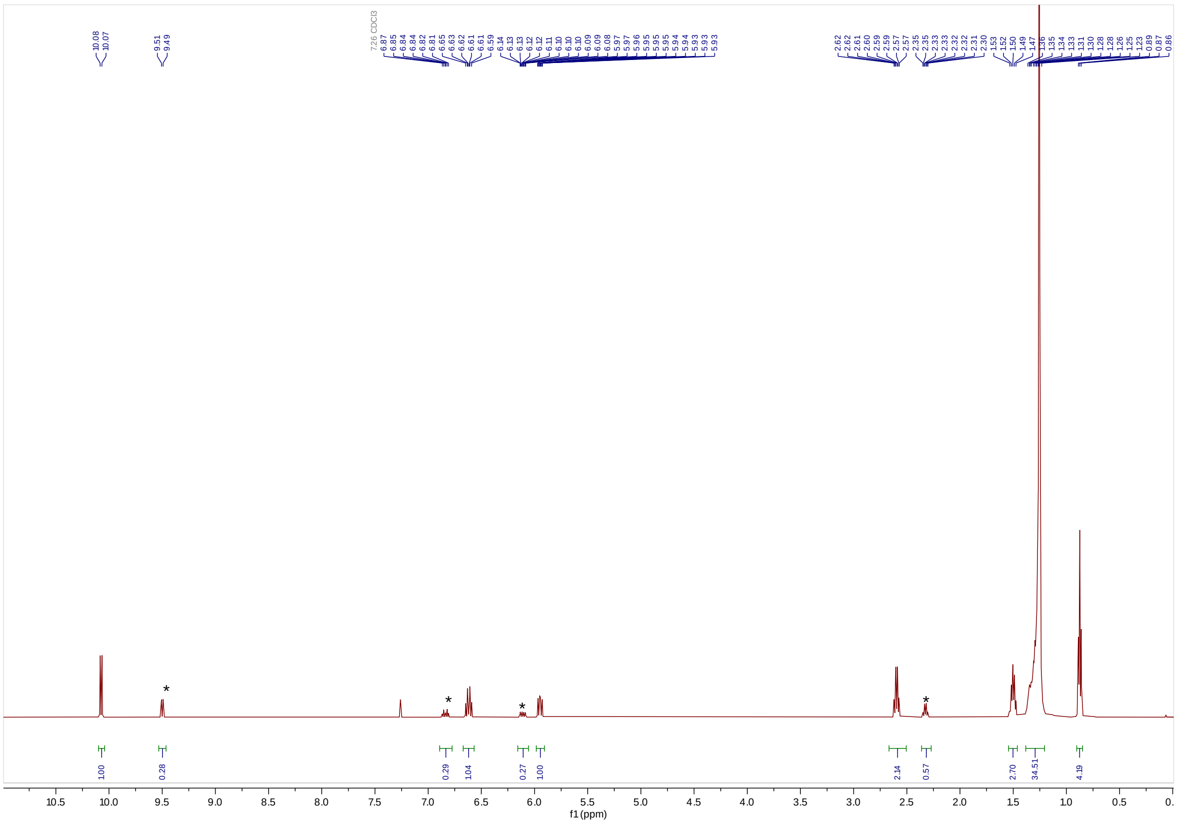

^{13}C-NMR (125.65 MHz, $CDCl_3$) of **3b** (see Procedure)

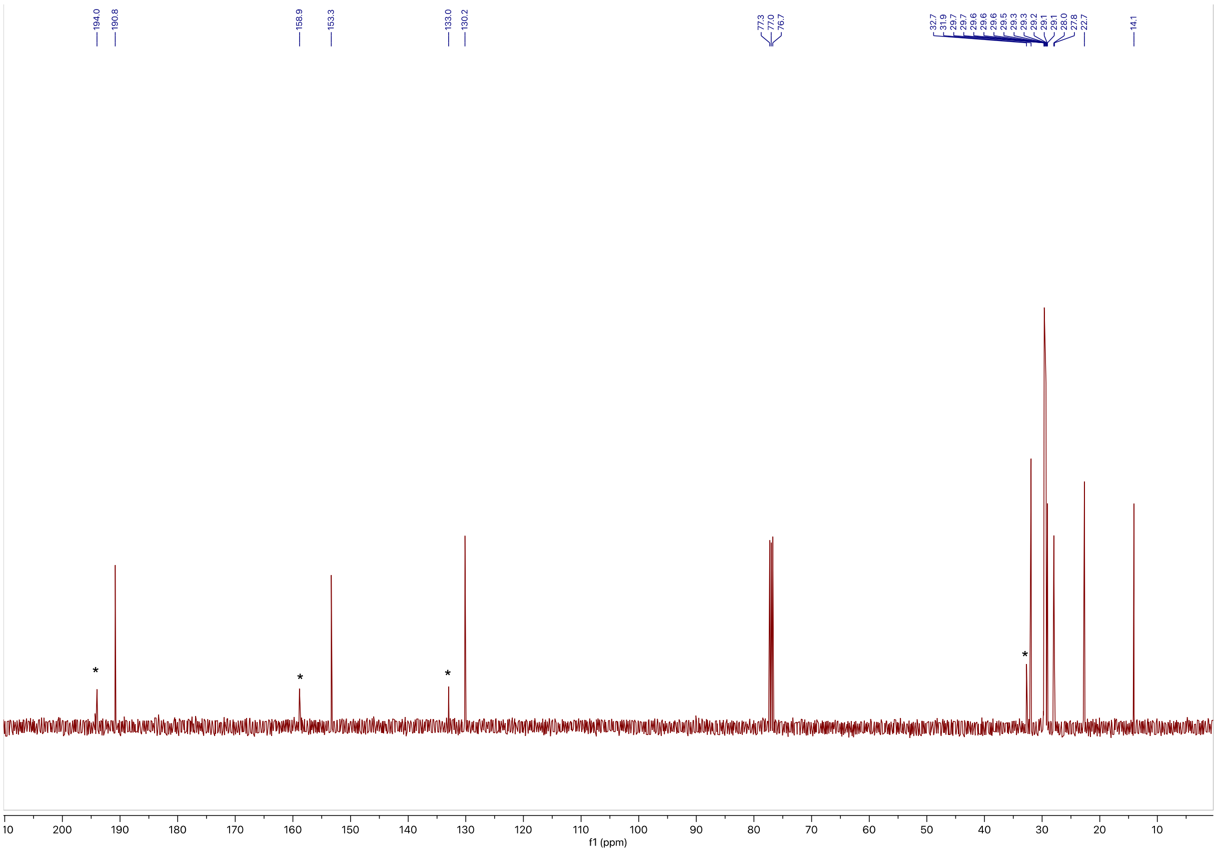

^{1}H-NMR (499.64 MHz, $CDCl_3$) of **3g** [(see Procedure)]

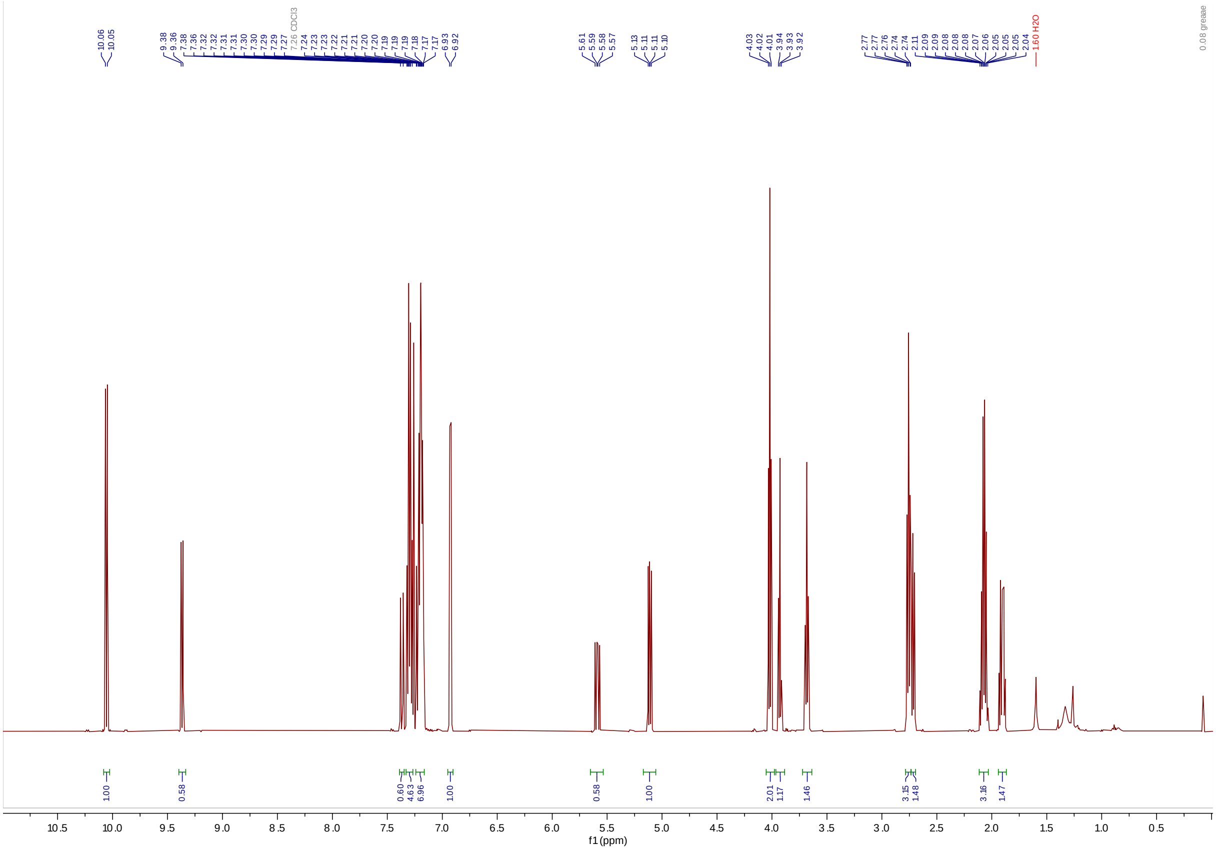

^{13}C-NMR (125.65 MHz, $CDCl_3$) of **3g** [(see Procedure)]

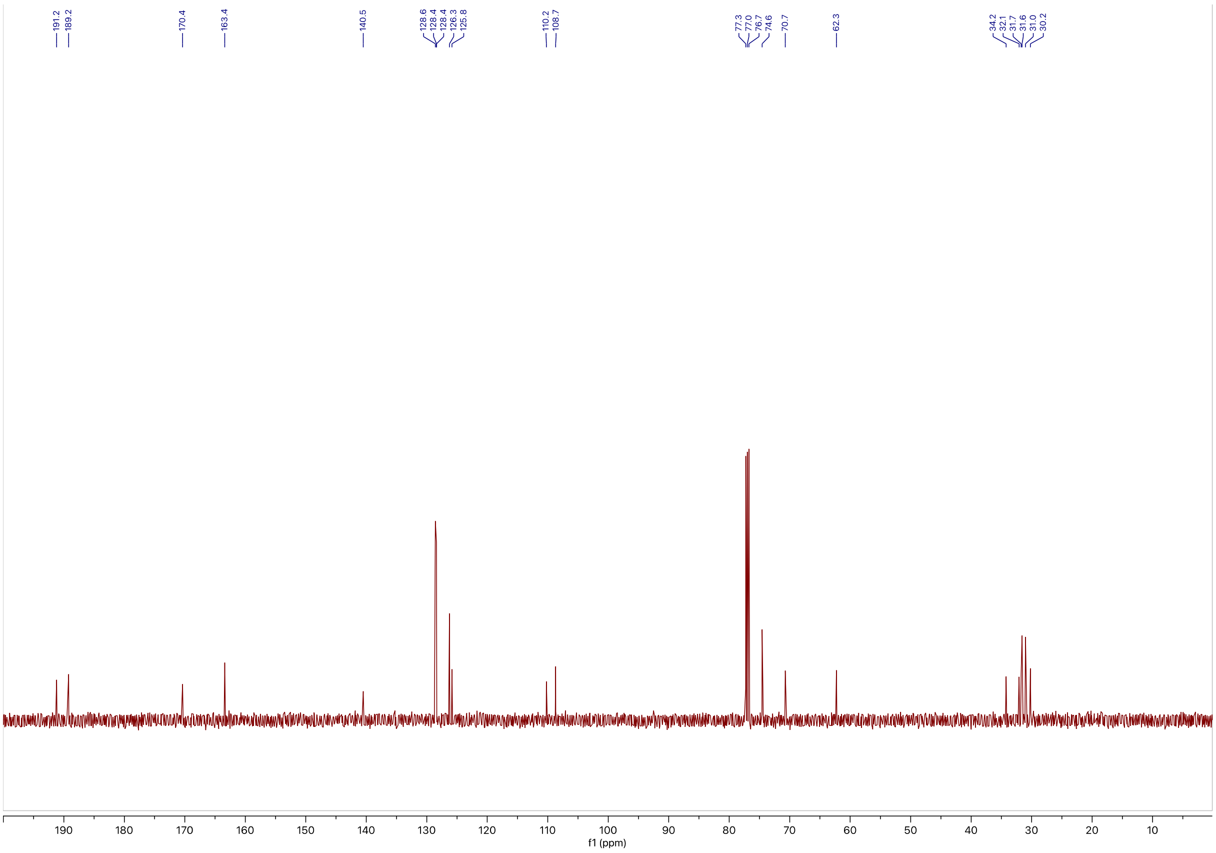

^{1}H-NMR (499.64 MHz, $CDCl_3$) of **3h** [(see Procedure)]

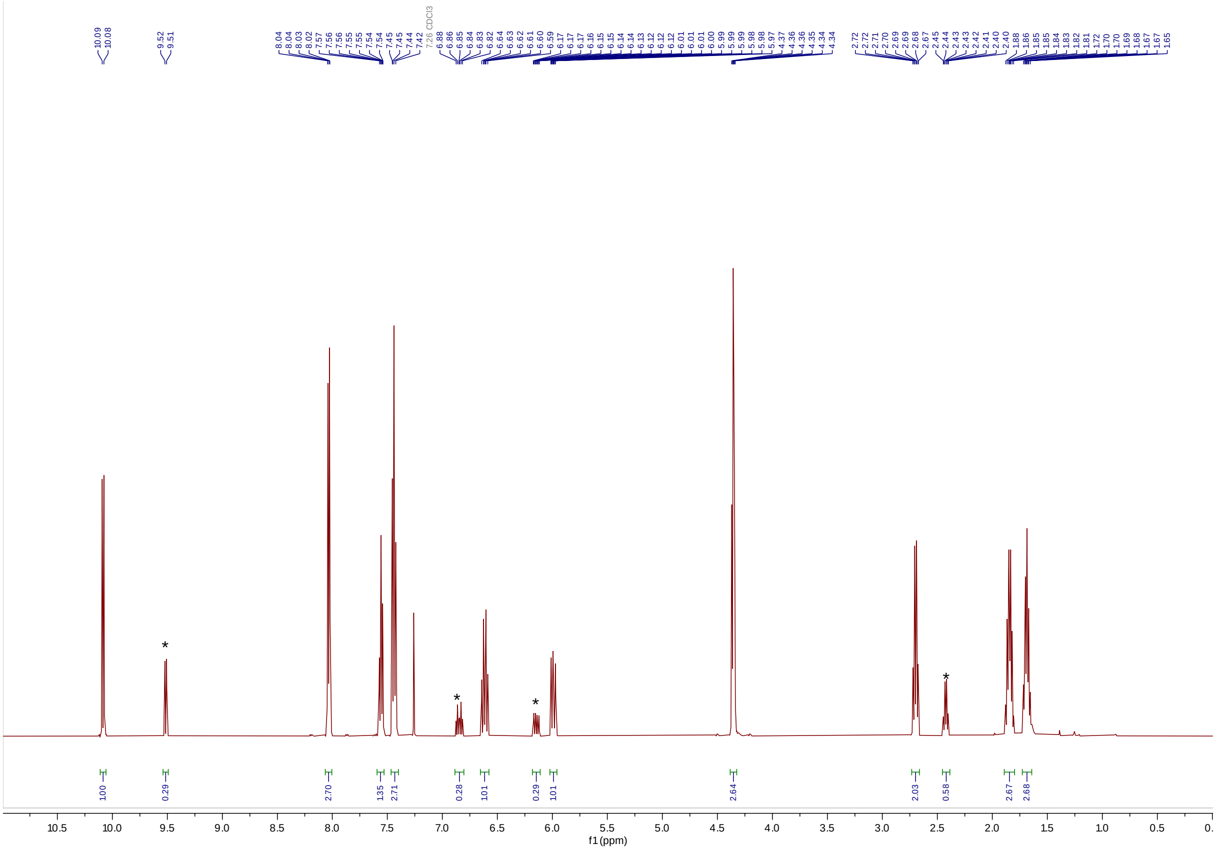

^{13}C-NMR (125.65 MHz, $CDCl_3$) of **3h** [(see Procedure)]

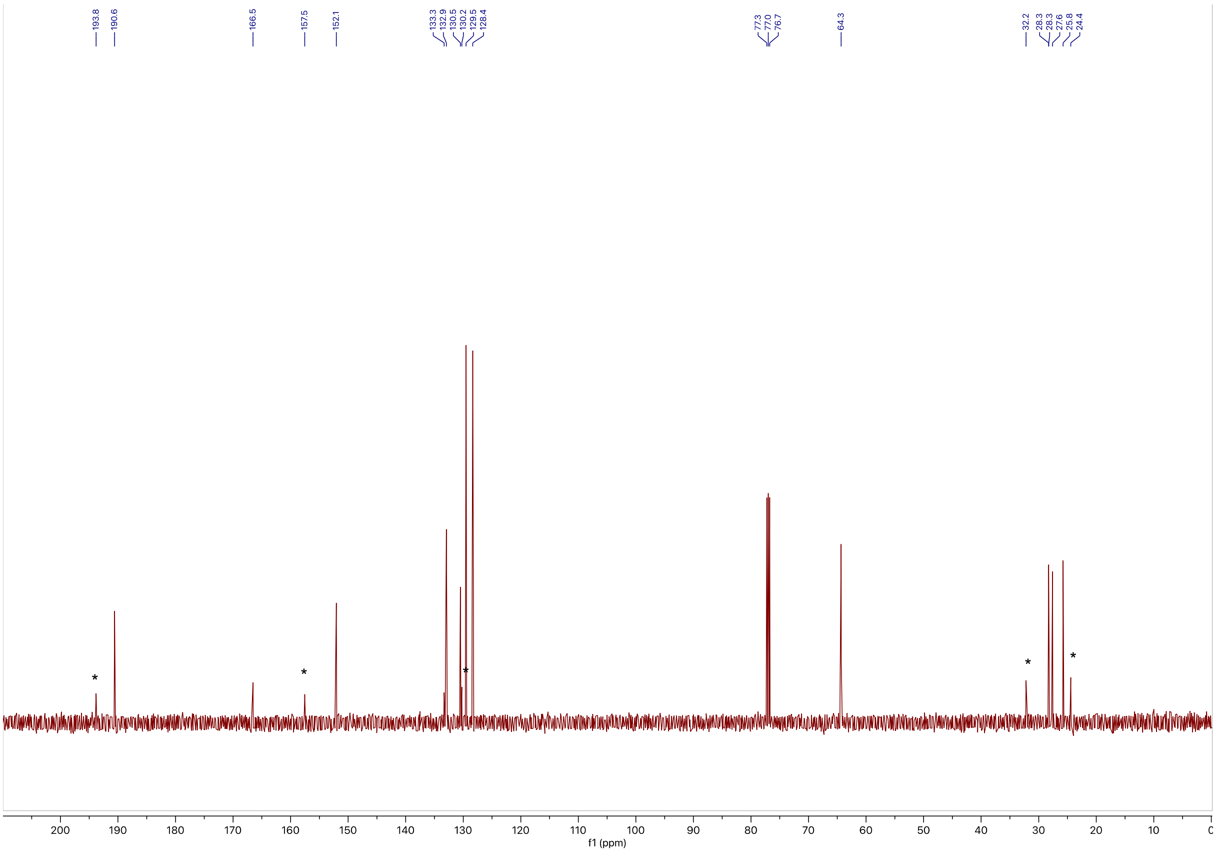

¹H-NMR (499.64 MHz, $CDCl_3$) of **3i** (see Procedure)

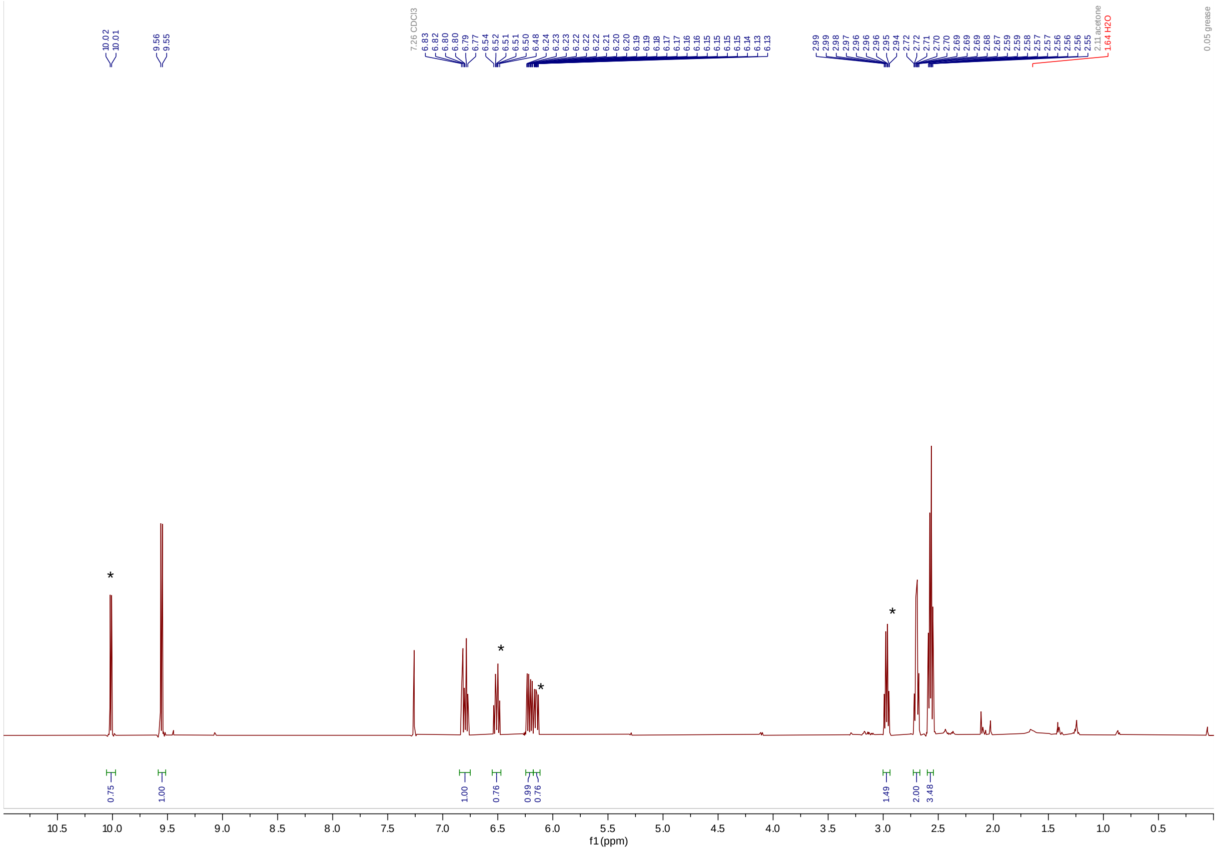

¹³C-NMR (125.65 MHz, $CDCl_3$) of **3i** (see Procedure)

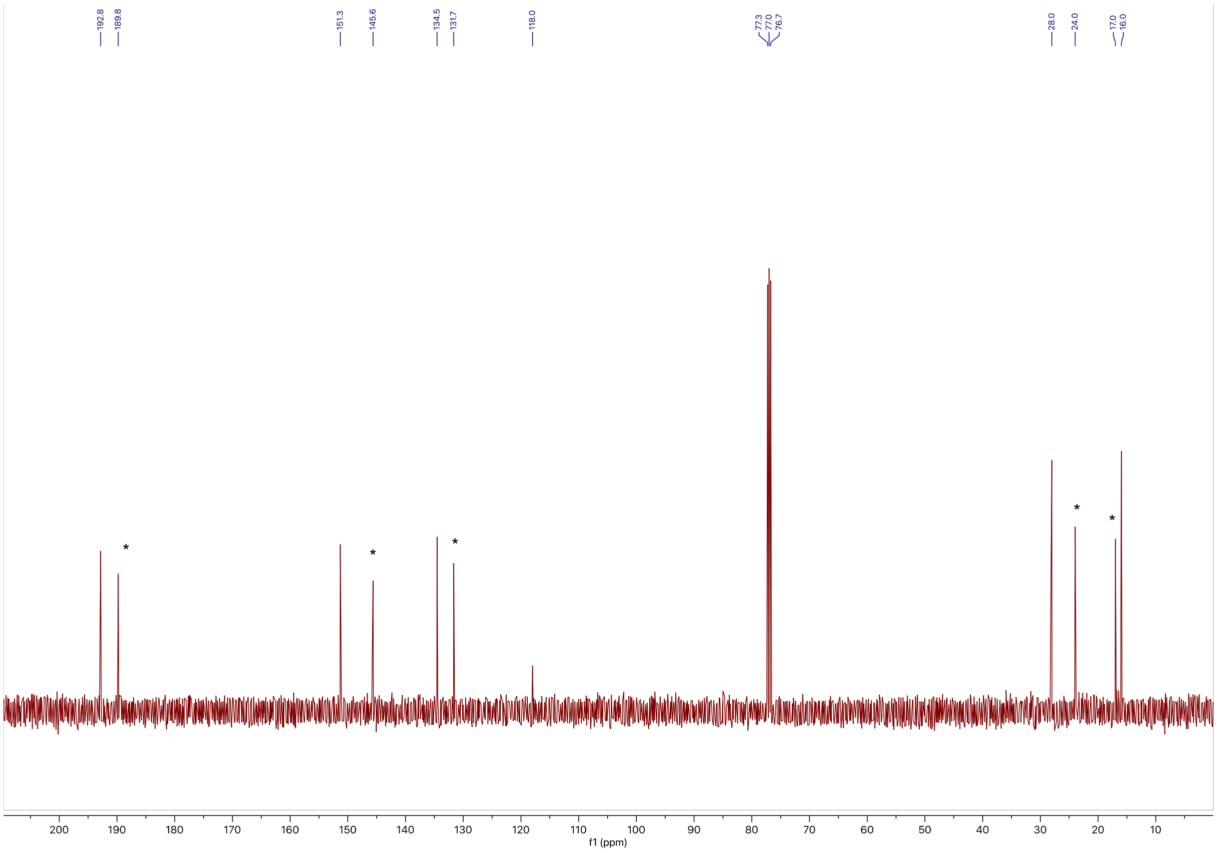

^{1}H-NMR (499.64 MHz, $CDCl_3$) of **3j** (see Procedure)

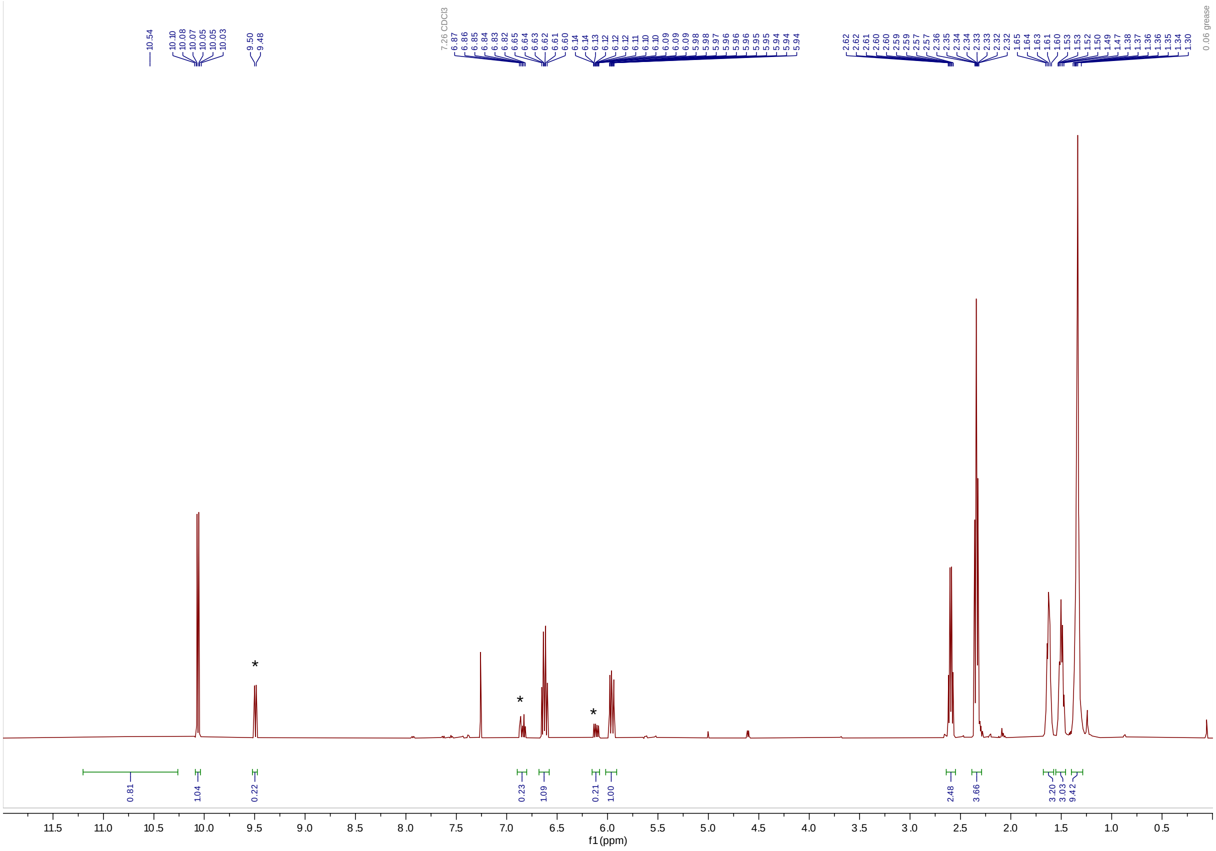

^{13}C-NMR (125.65 MHz, $CDCl_3$) of **3j** (see Procedure)

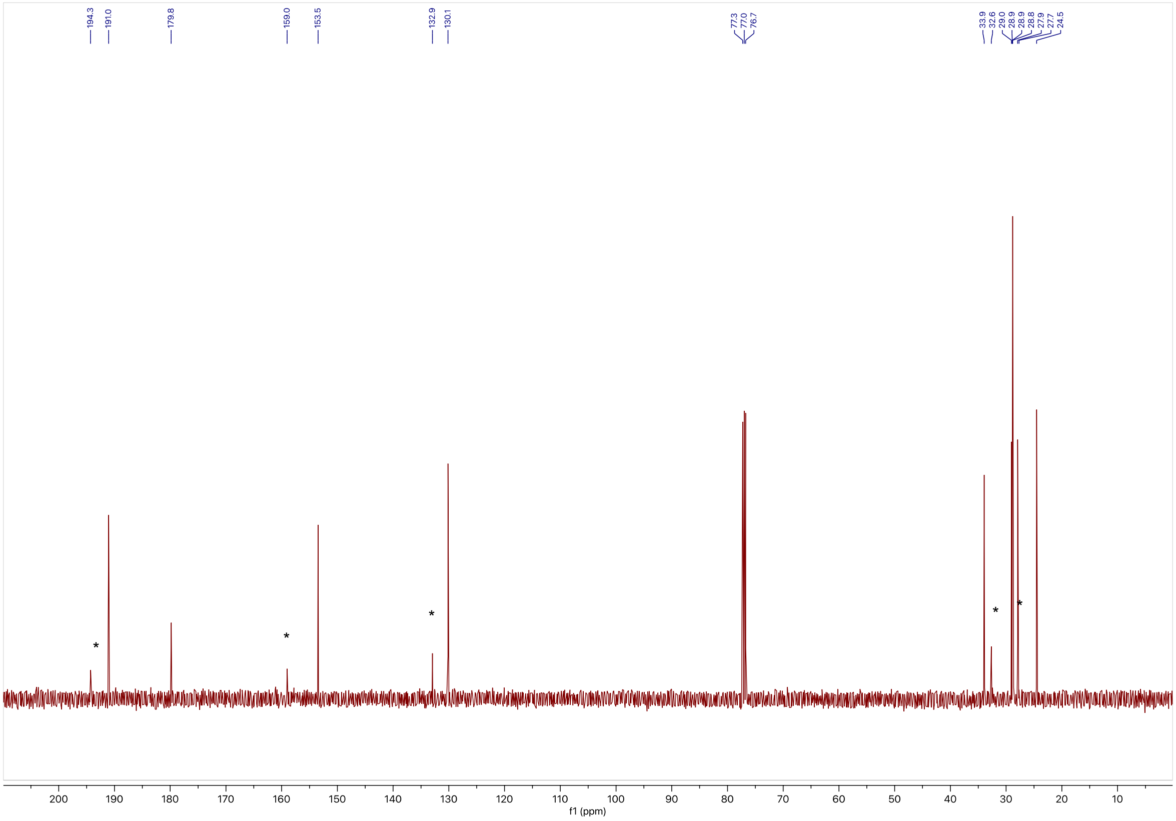

^{1}**H-NMR** (499.64 MHz, $CDCl_3$) of **3k** (see Procedure)

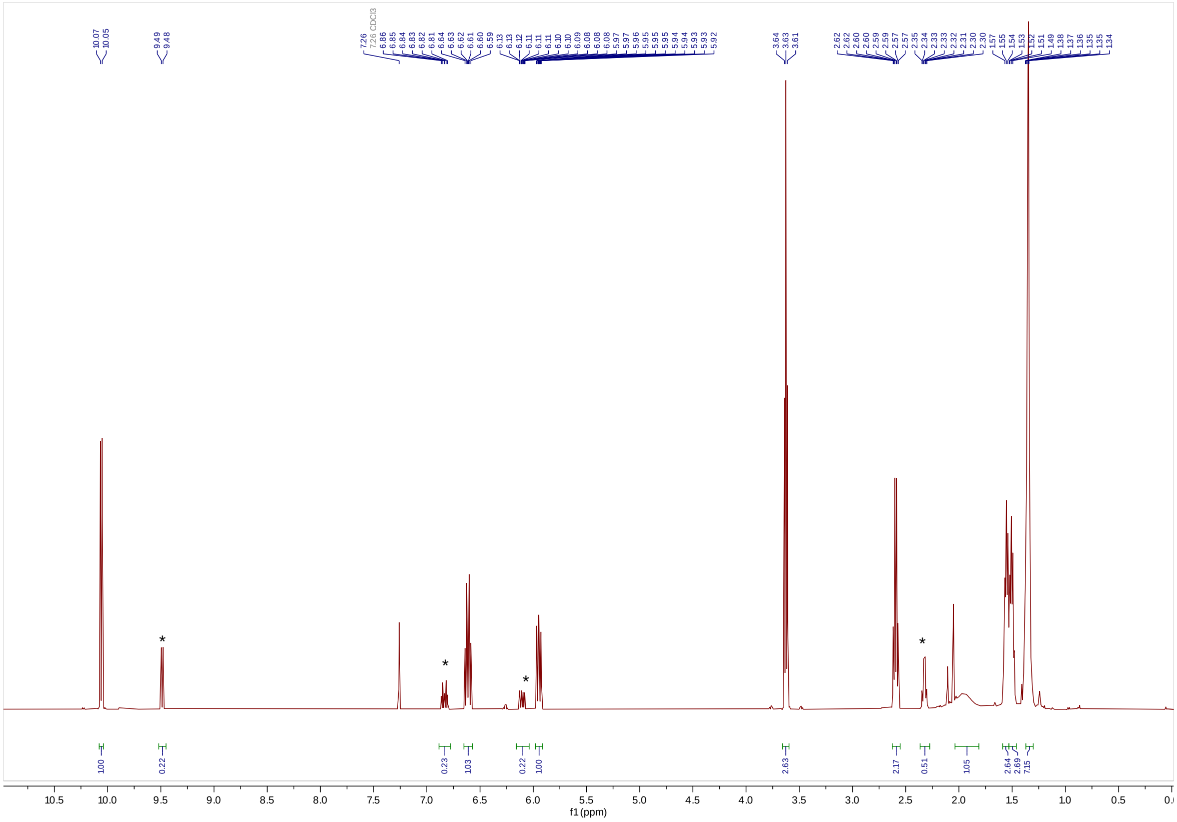

^{13}**C-NMR** (125.65 MHz, $CDCl_3$) of **3k** (see Procedure)

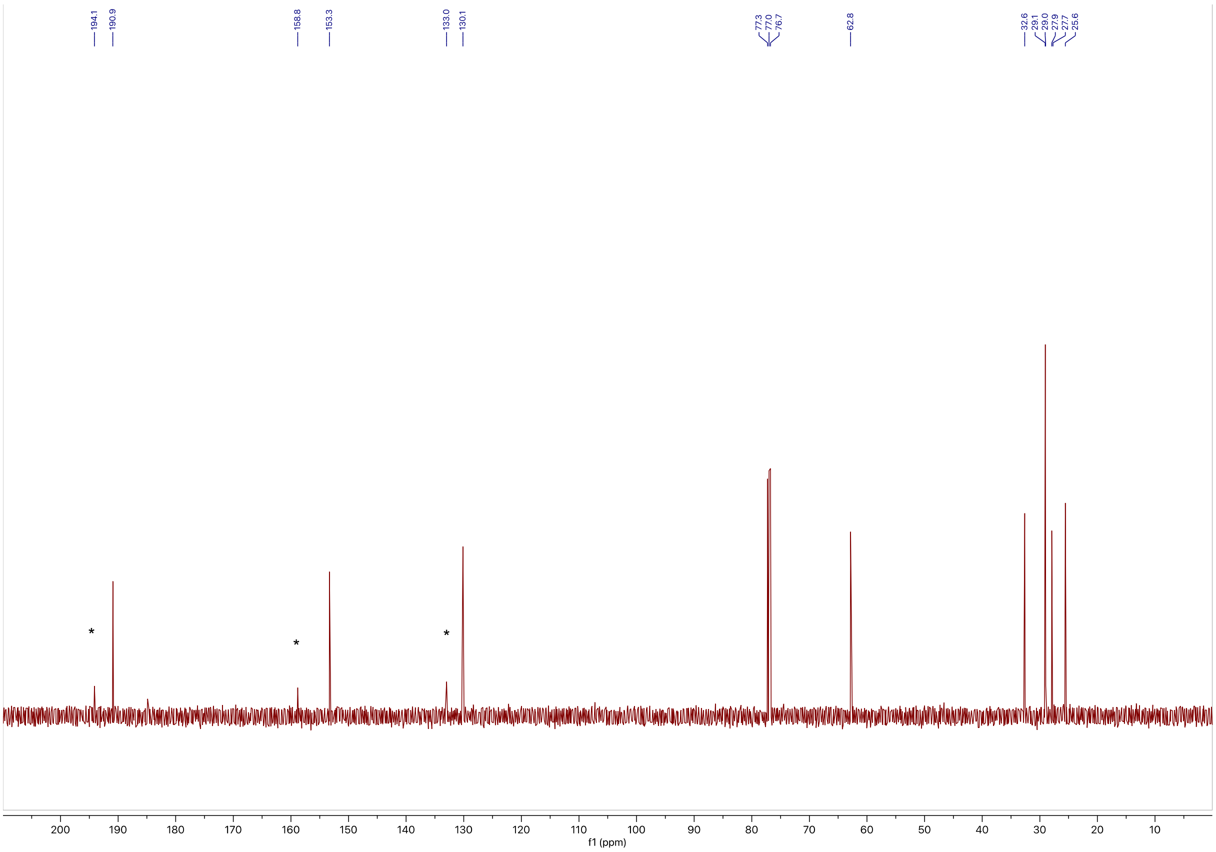

^{1}H-NMR (499.64 MHz, $CDCl_3$) of **3l** [(see Procedure)](#)

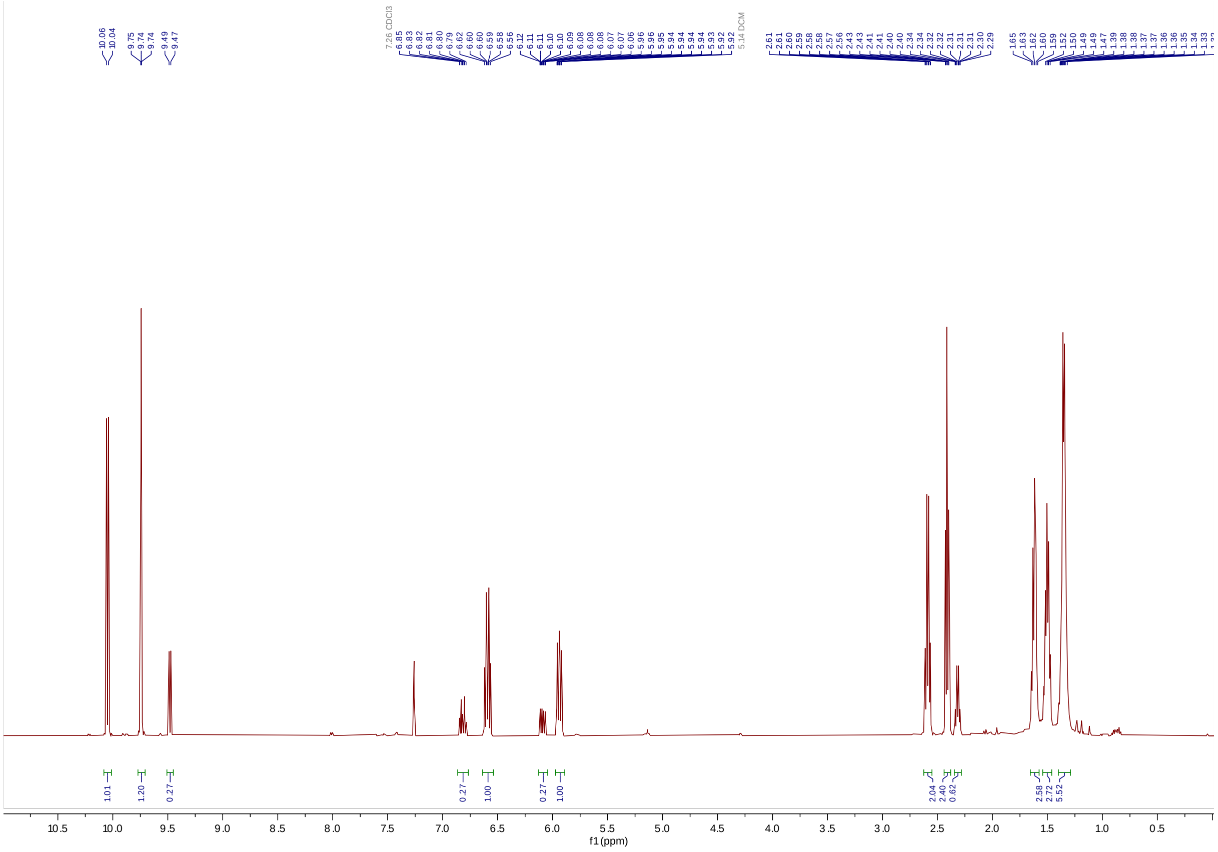

^{13}C-NMR (125.65 MHz, $CDCl_3$) of **3l** [(see Procedure)](#)

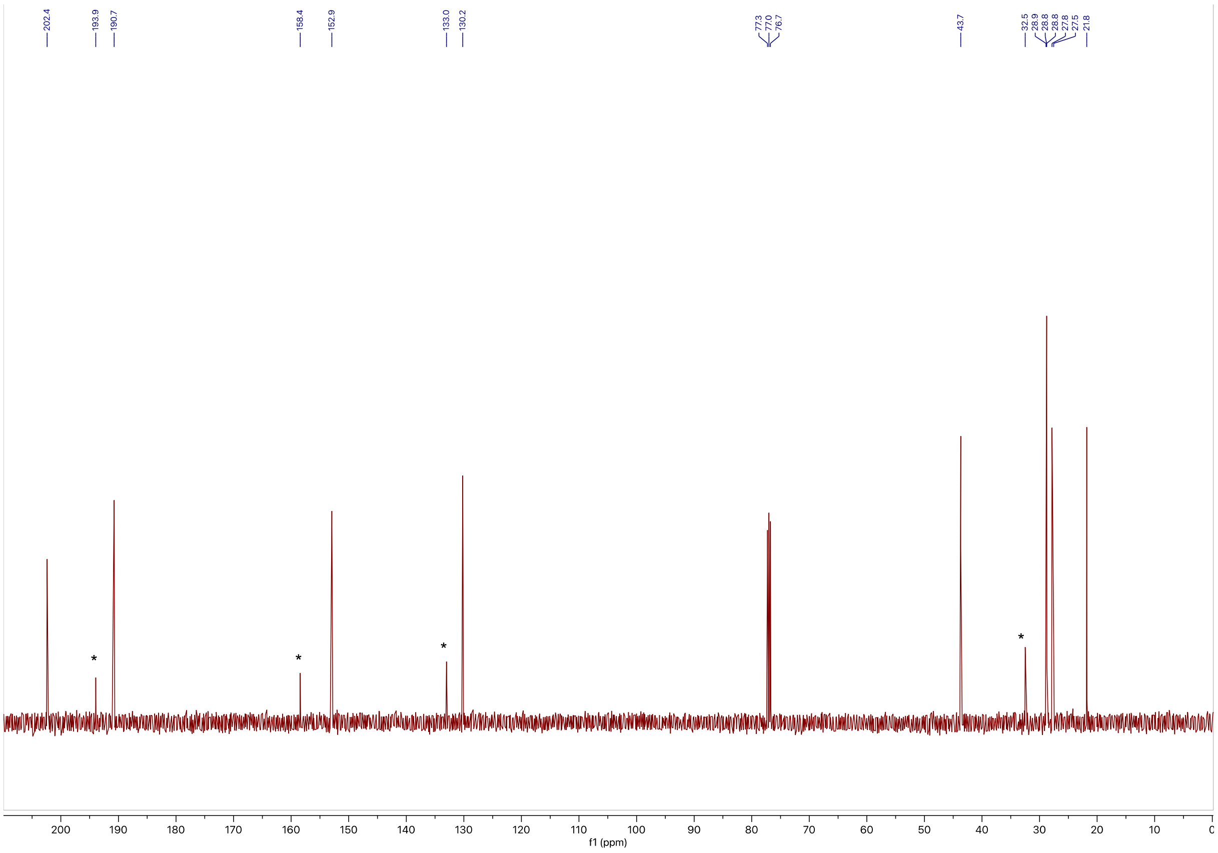

^{1}H-NMR (499.64 MHz, $CDCl_3$) of **3m** (see Procedure)

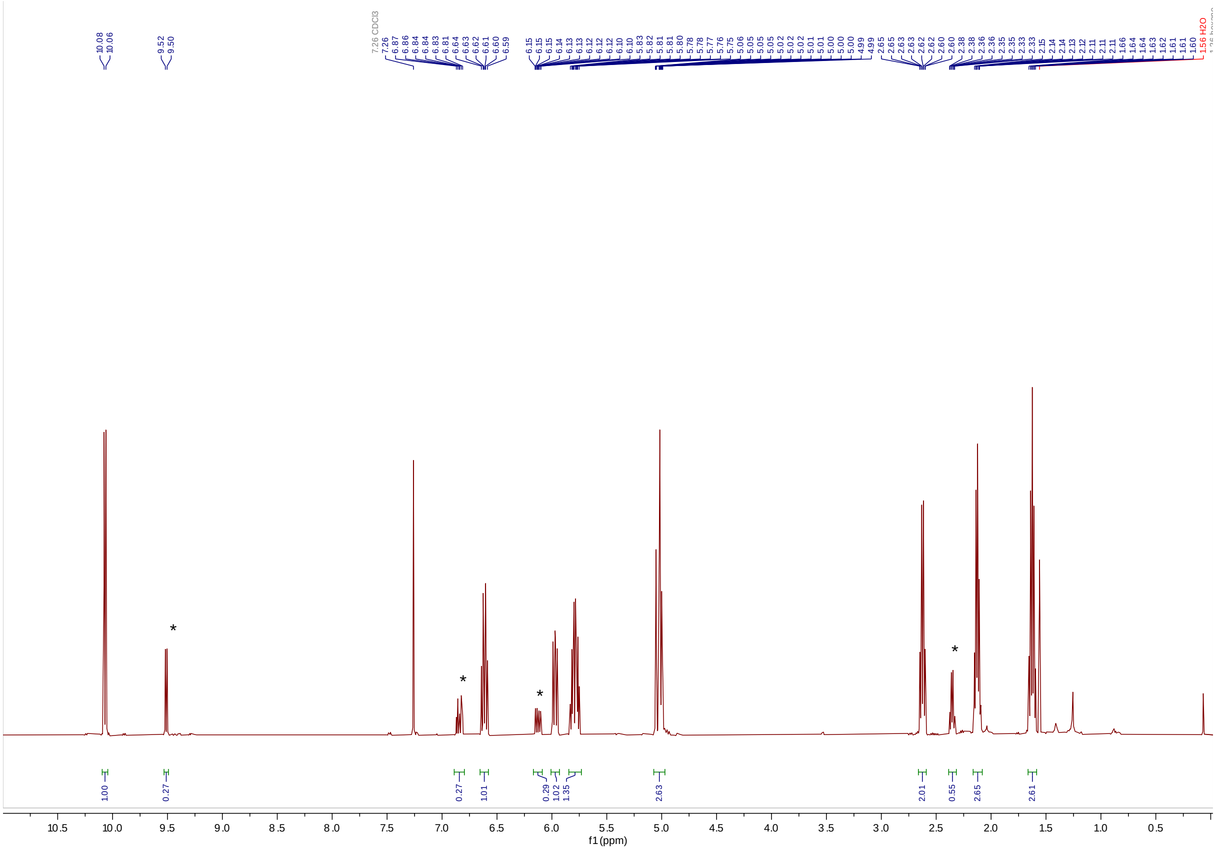

^{13}C-NMR (125.65 MHz, $CDCl_3$) of **3m** (see Procedure)

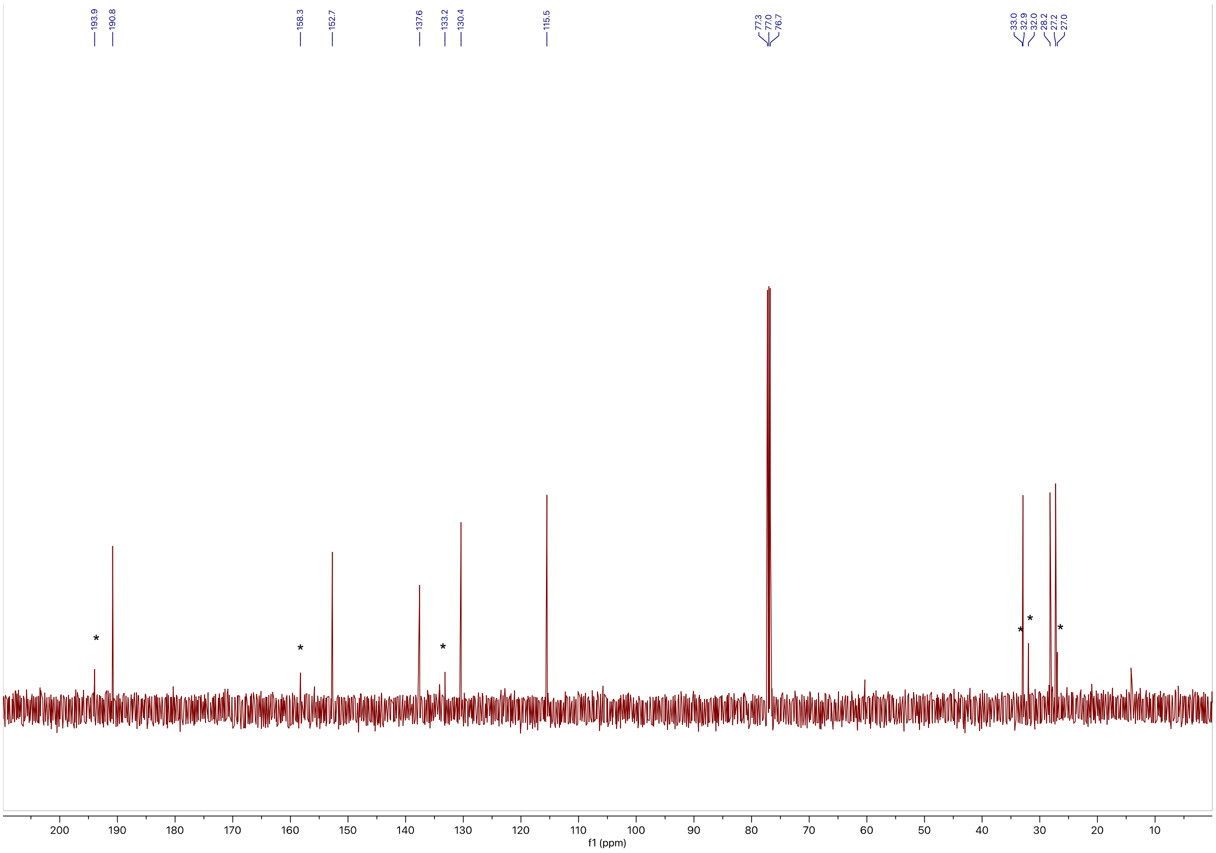

^{1}H-NMR (499.64 MHz, $CDCl_3$) of **3n** (see Procedure)

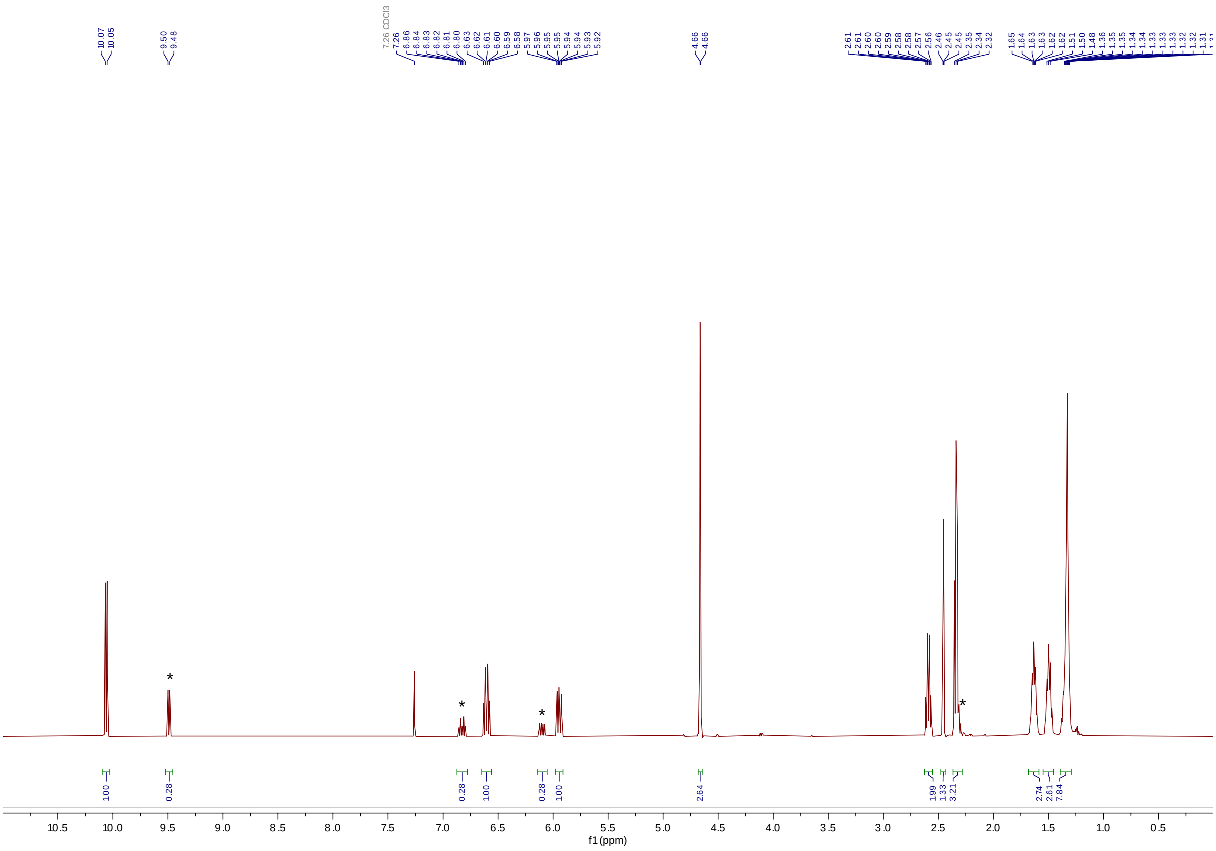

^{13}C-NMR (125.65 MHz, $CDCl_3$) of **3n** (see Procedure)

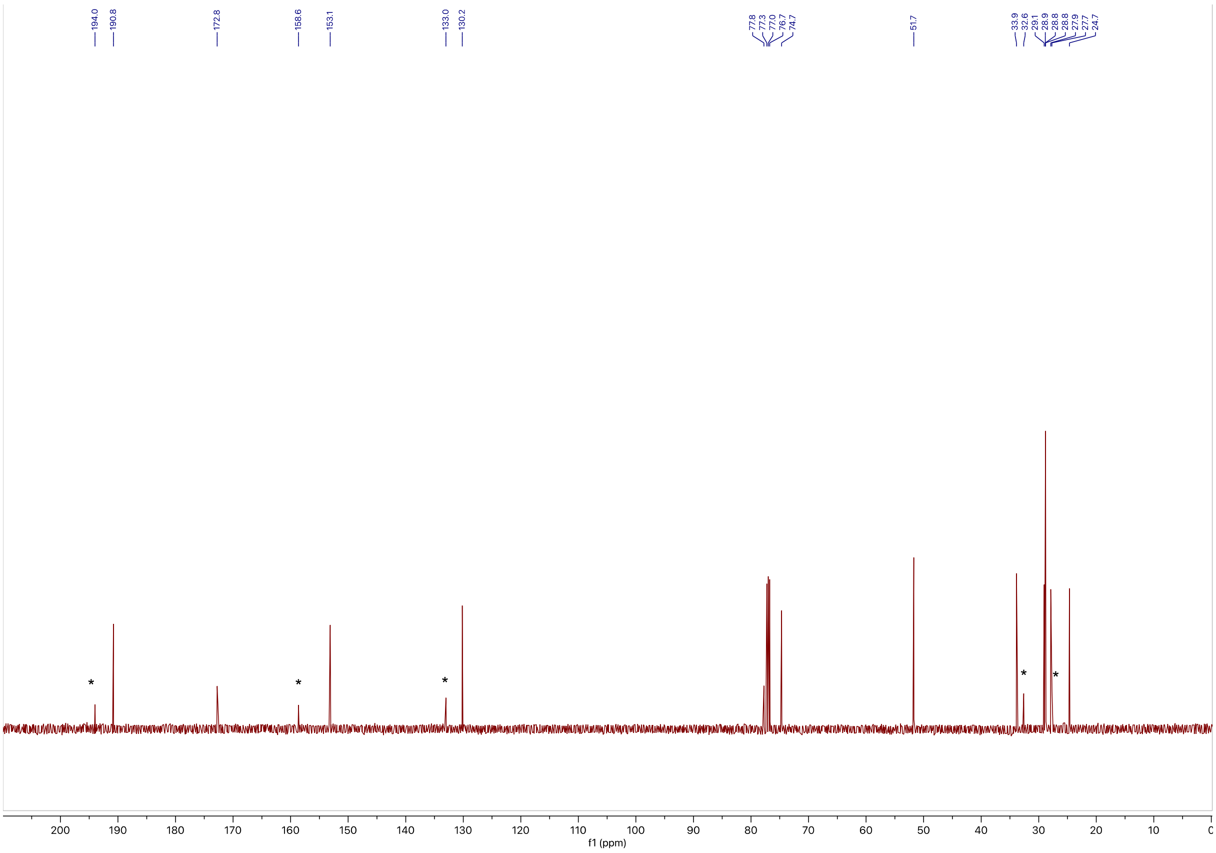

1H-NMR (499.64 MHz, $CDCl_3$) of **3o** (see Procedure)

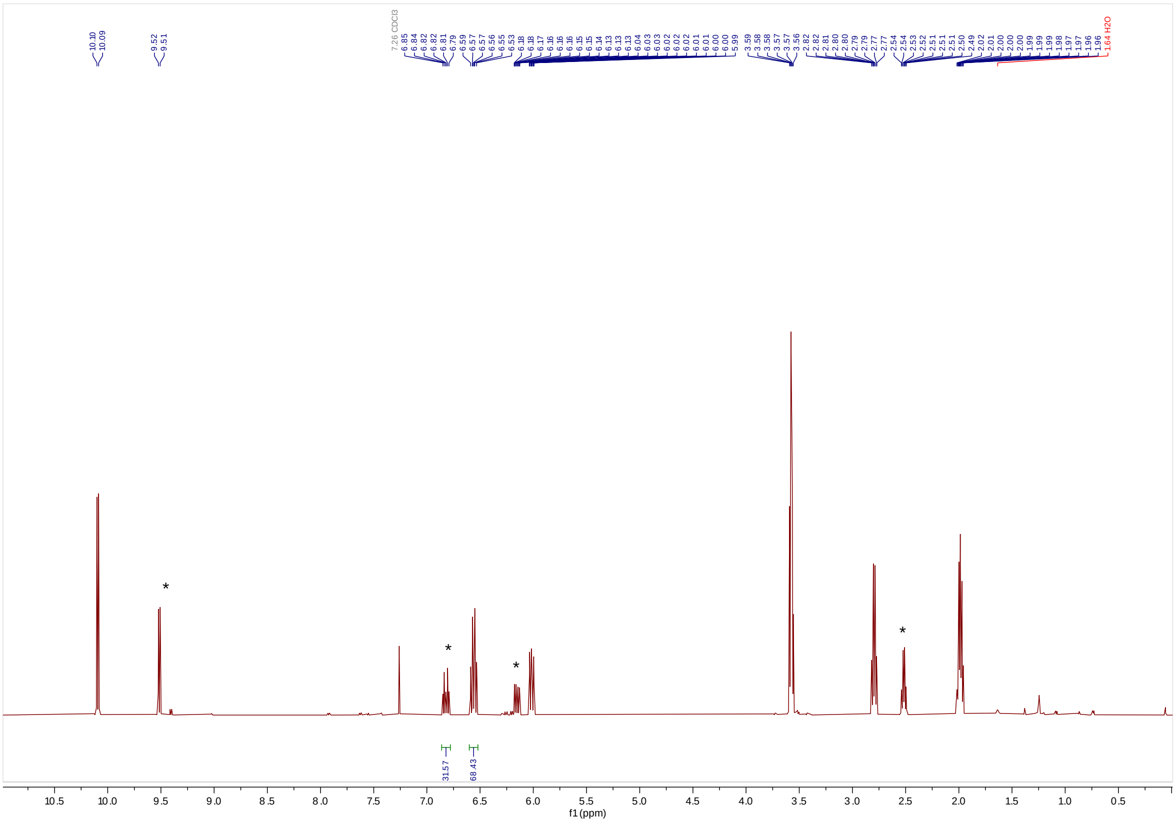

13C-NMR (125.65 MHz, $CDCl_3$) of **3o** (see Procedure)

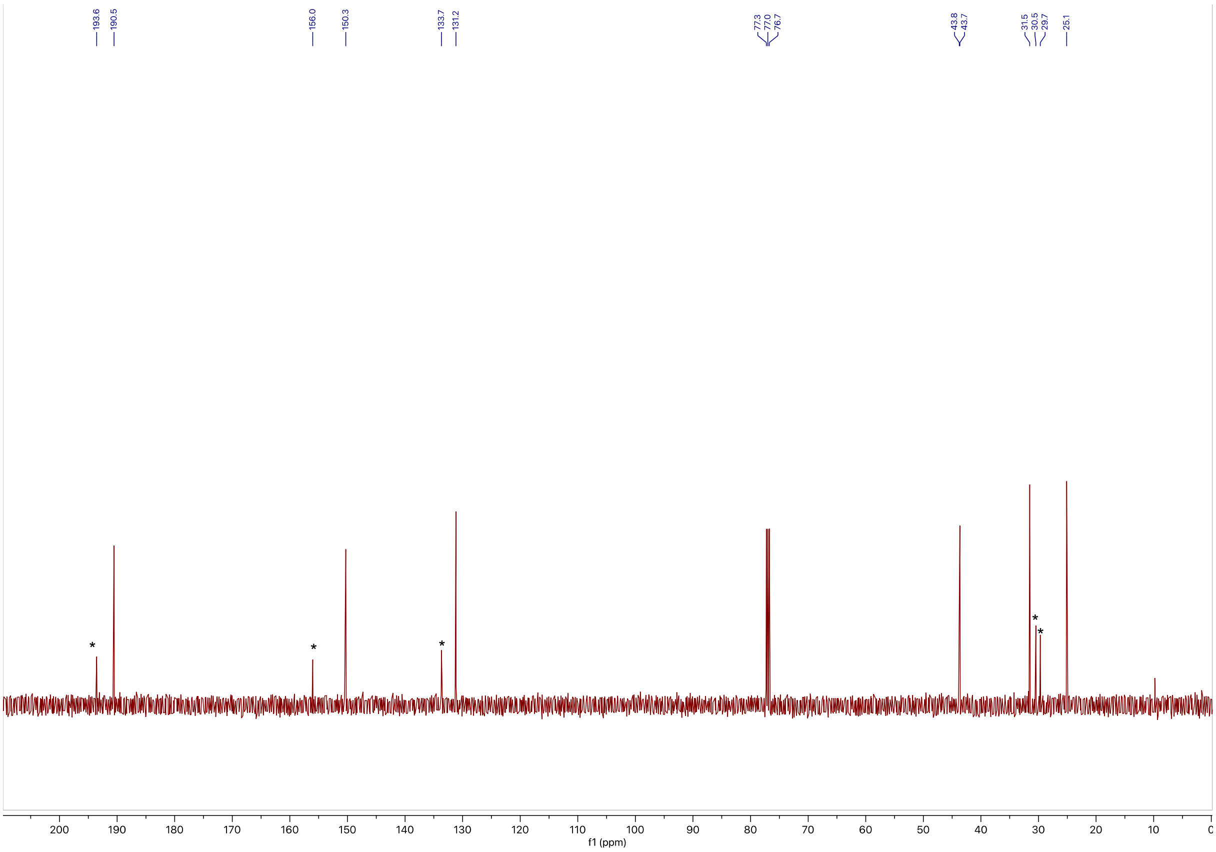

^{1}H-NMR (499.64 MHz, $CDCl_3$) of **3p** [(see Procedure)]

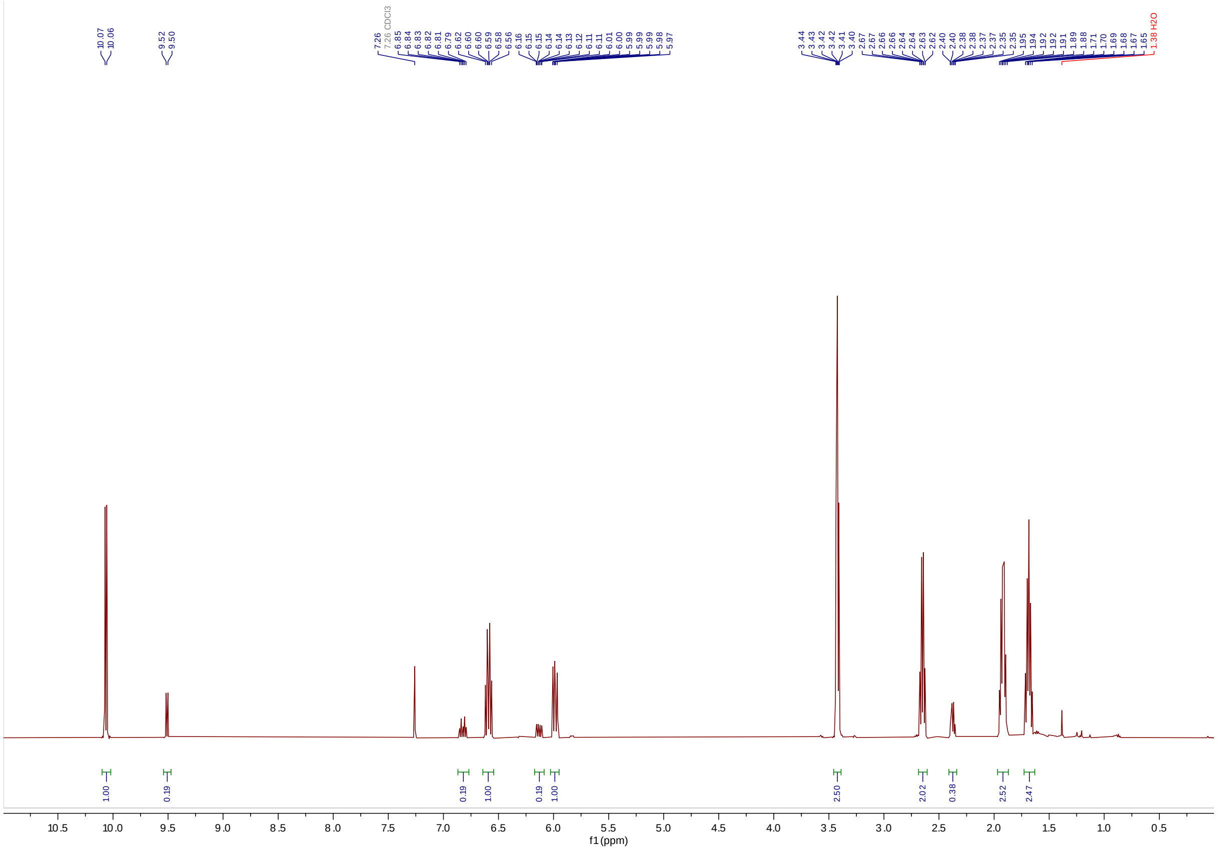

^{13}C-NMR (125.65 MHz, $CDCl_3$) of **3p** [(see Procedure)]

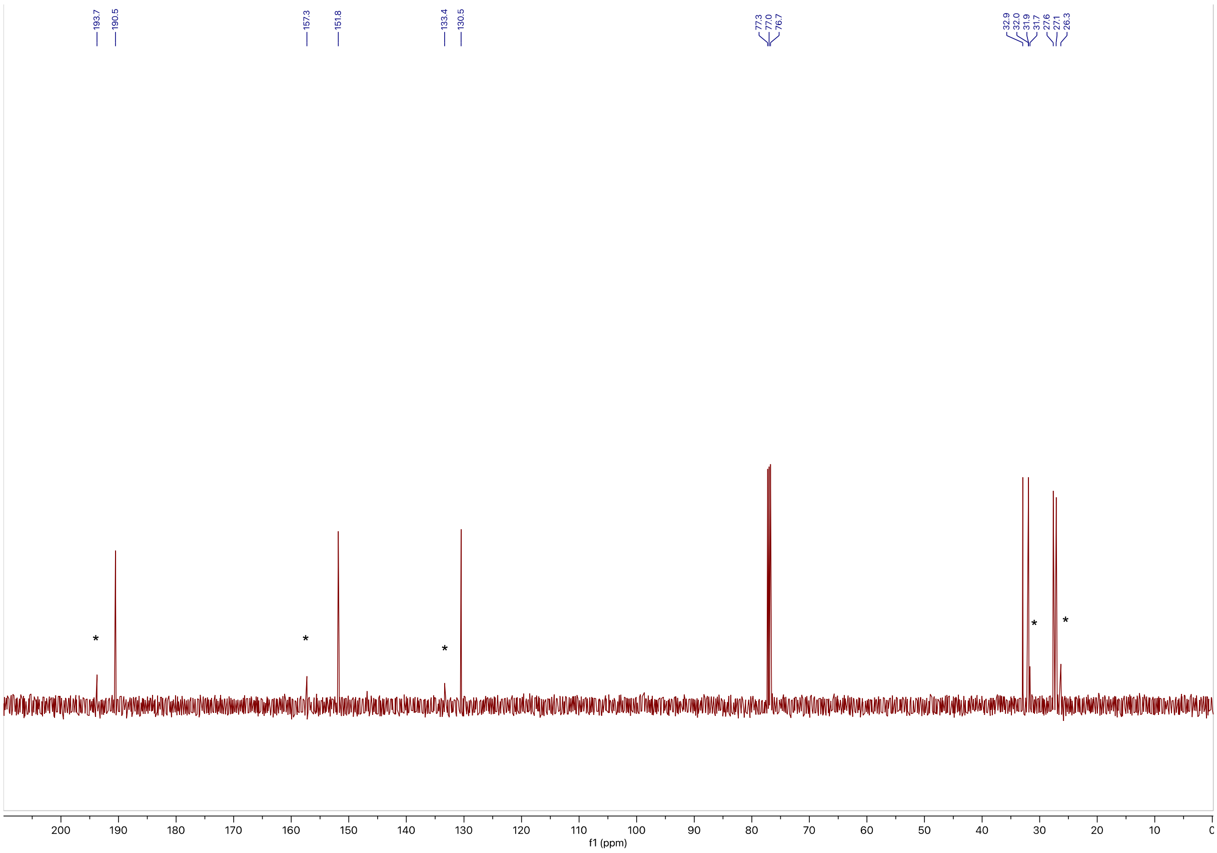

[1]**H-NMR** (499.64 MHz, $CDCl_3$) of **3q** (see Procedure)

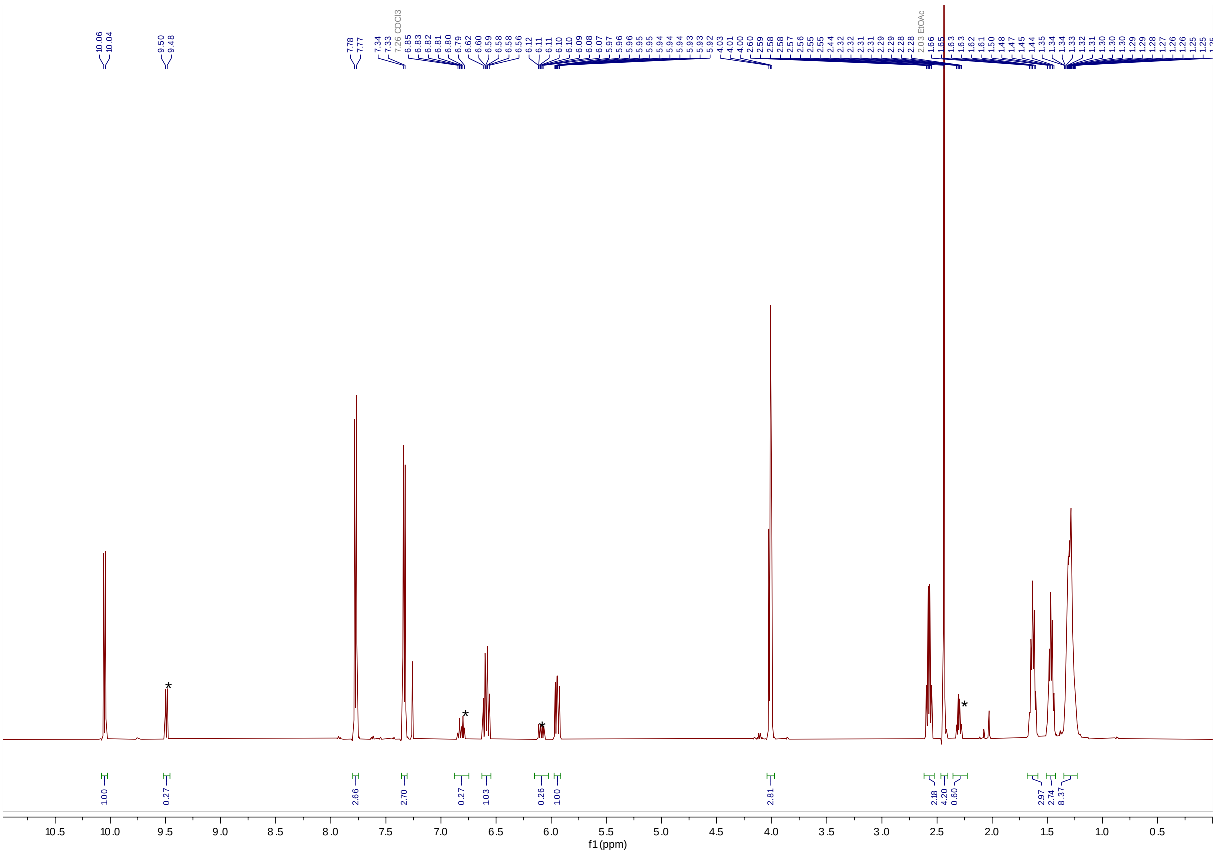

[13]**C-NMR** (125.65 MHz, $CDCl_3$) of **3q** (see Procedure)

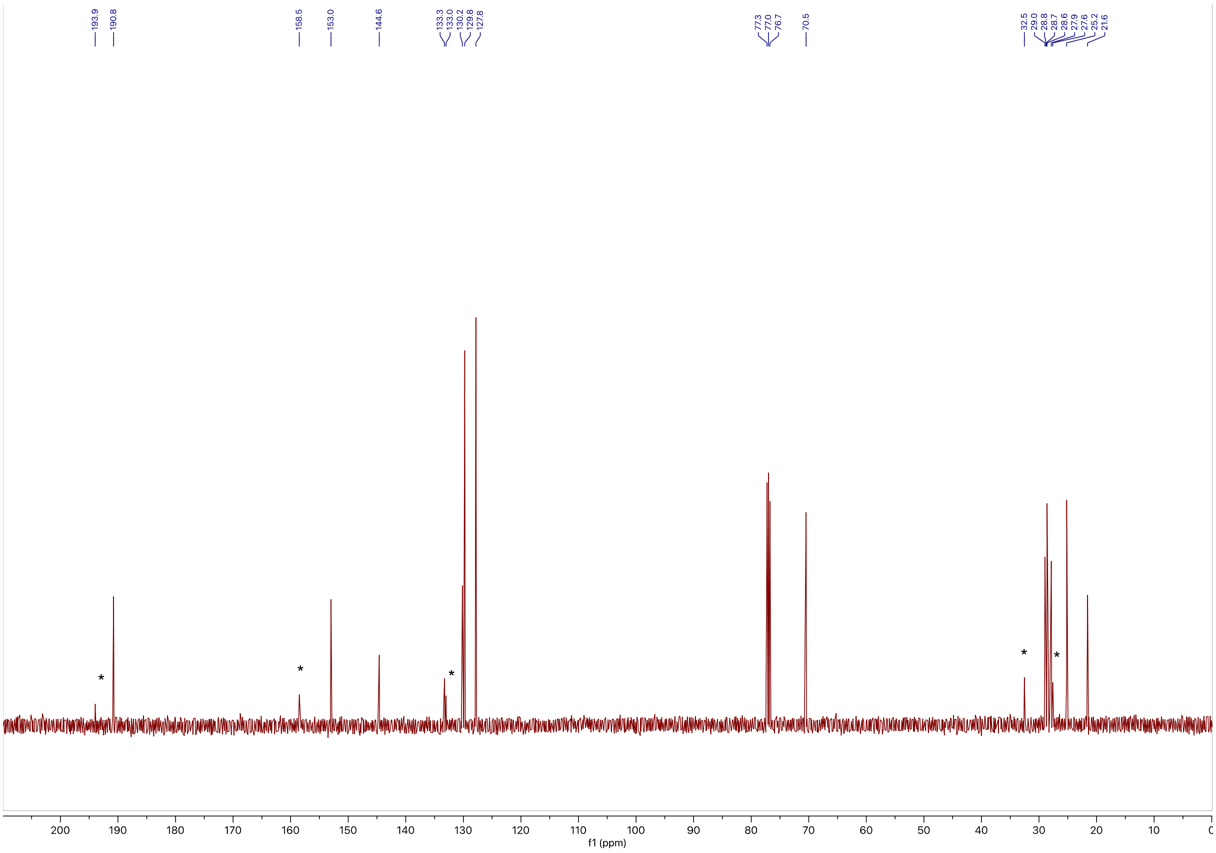

^{1}H-NMR (499.64 MHz, $CDCl_3$) of **5g** (see Procedure)

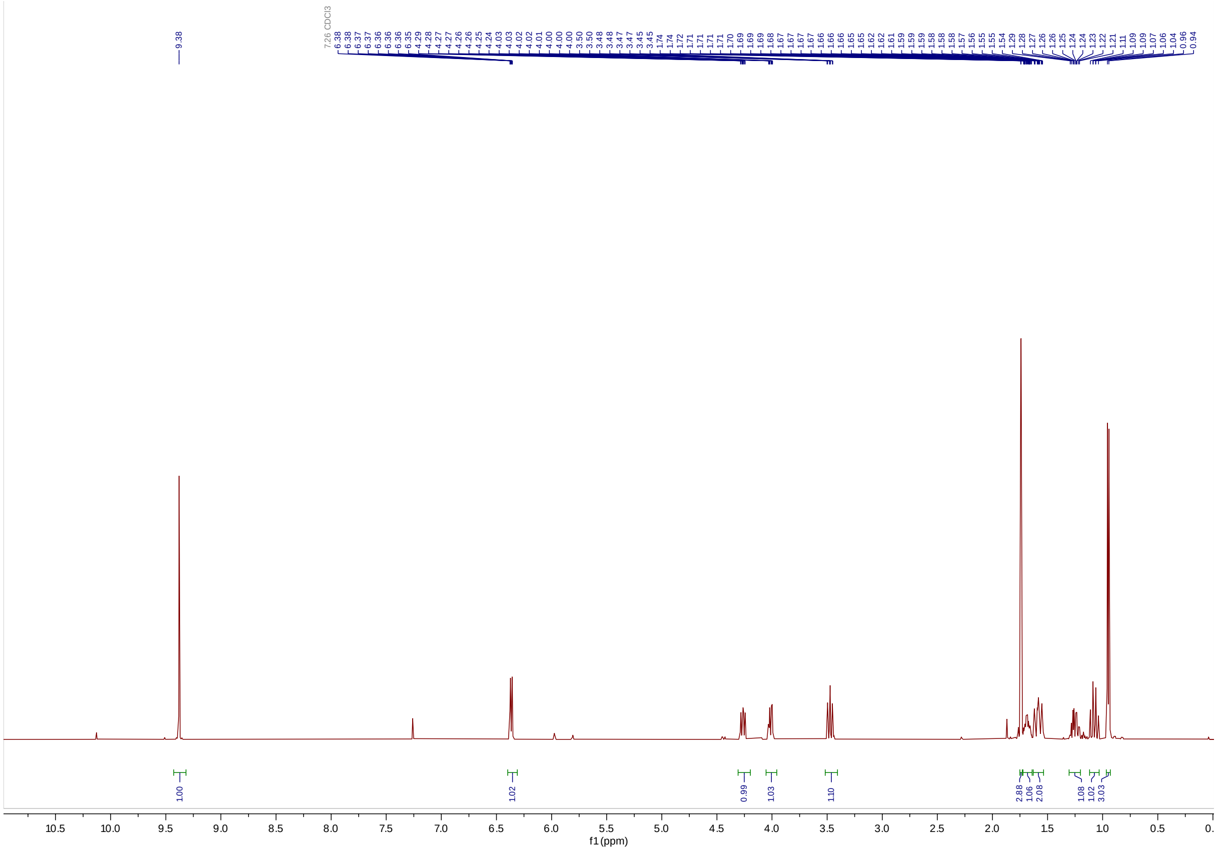

^{13}C-NMR (125.65 MHz, $CDCl_3$) of **5g** (see Procedure)

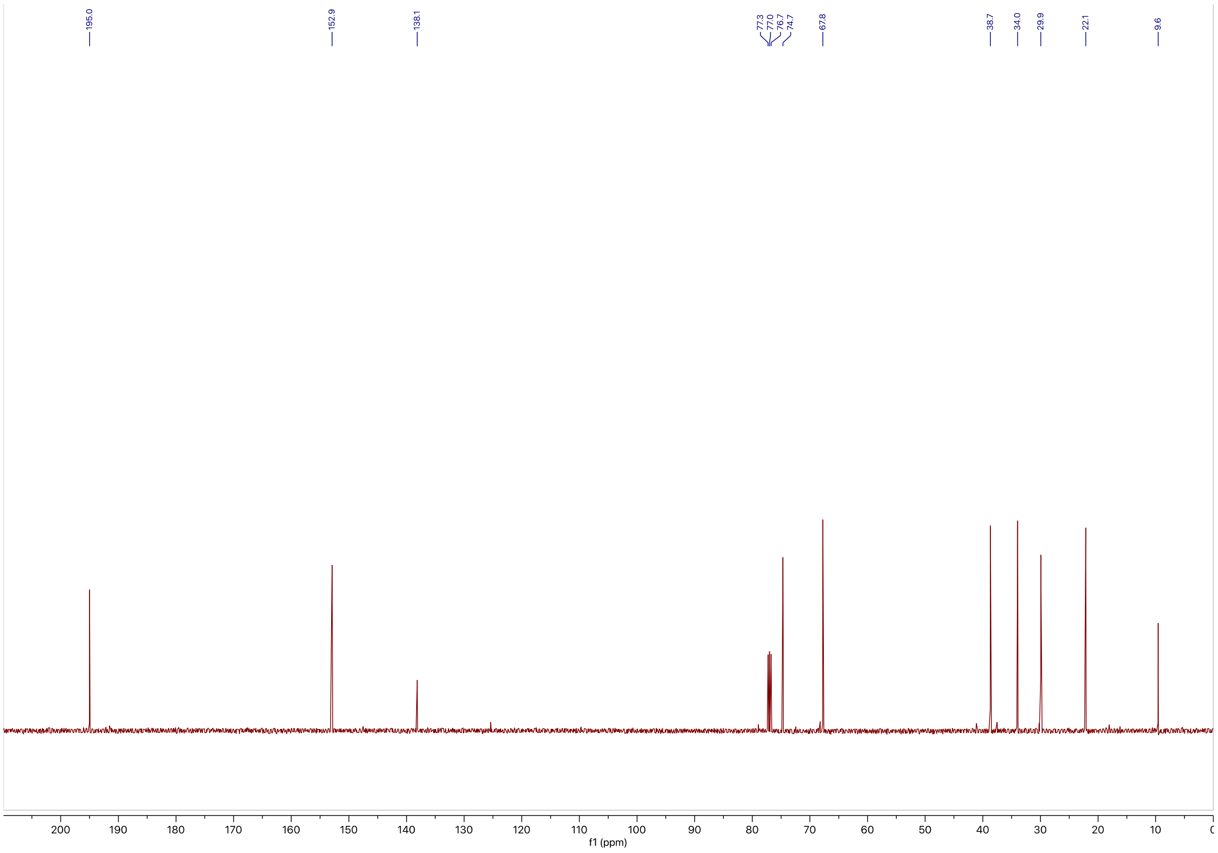

^{1}H-NMR (499.64 MHz, $CDCl_3$) of **5h** (see Procedure)

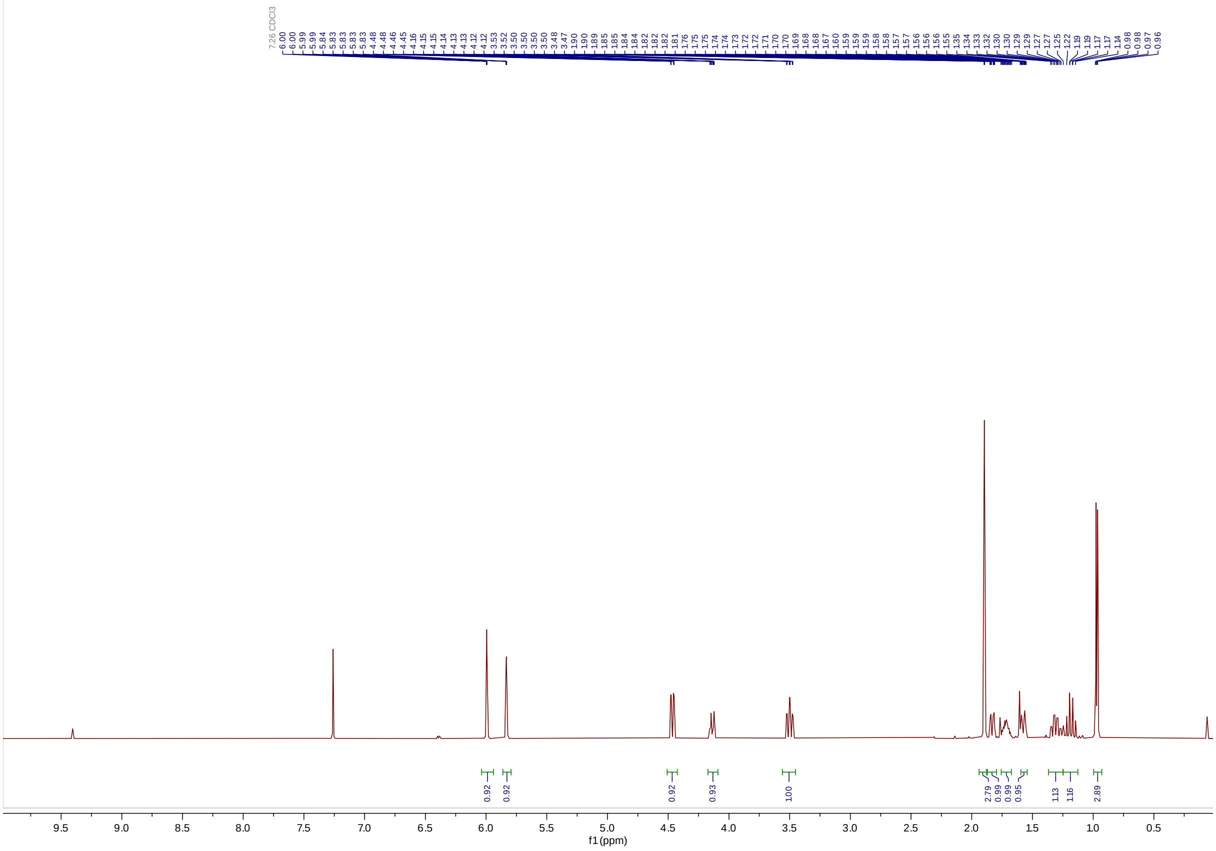

^{13}C-NMR (125.65 MHz, $CDCl_3$) of **5h** (see Procedure)

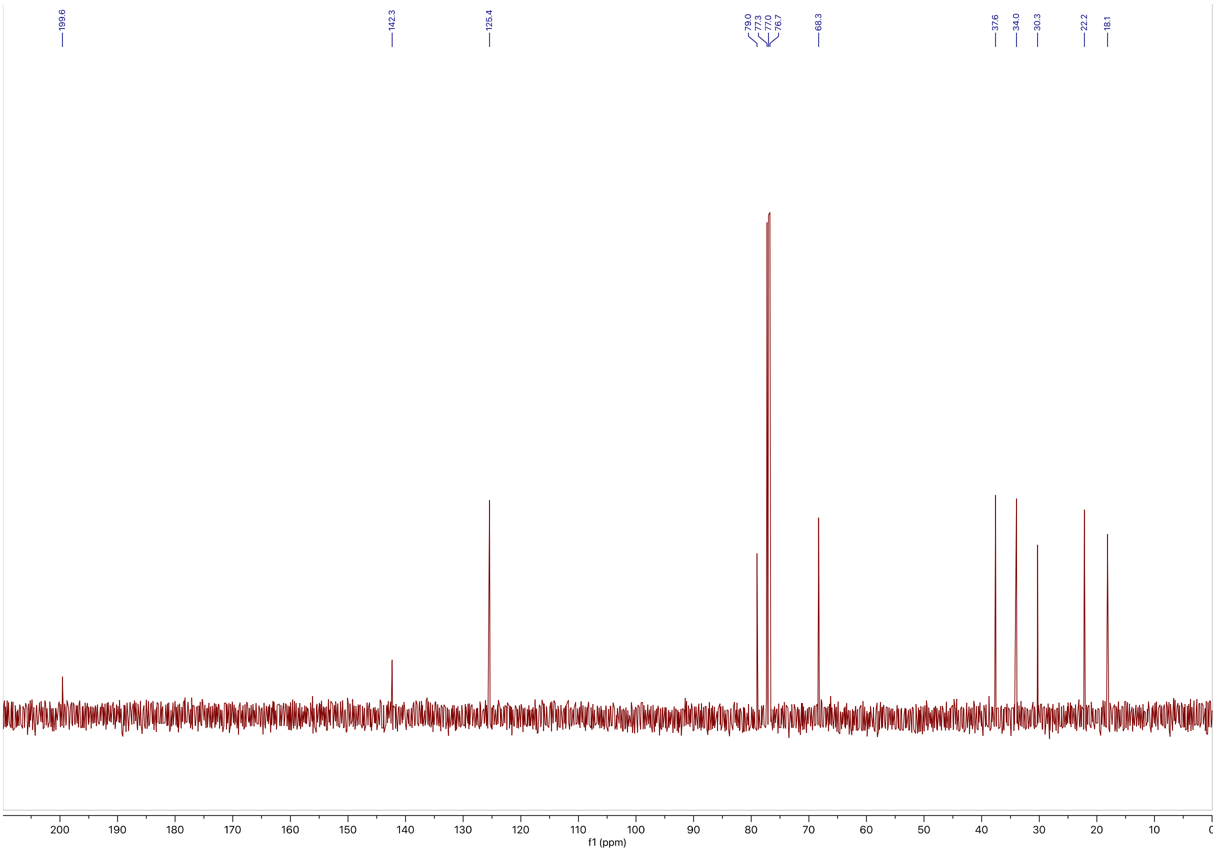

^{1}H-NMR (499.64 MHz, $CDCl_3$) of **6a** (see Procedure)

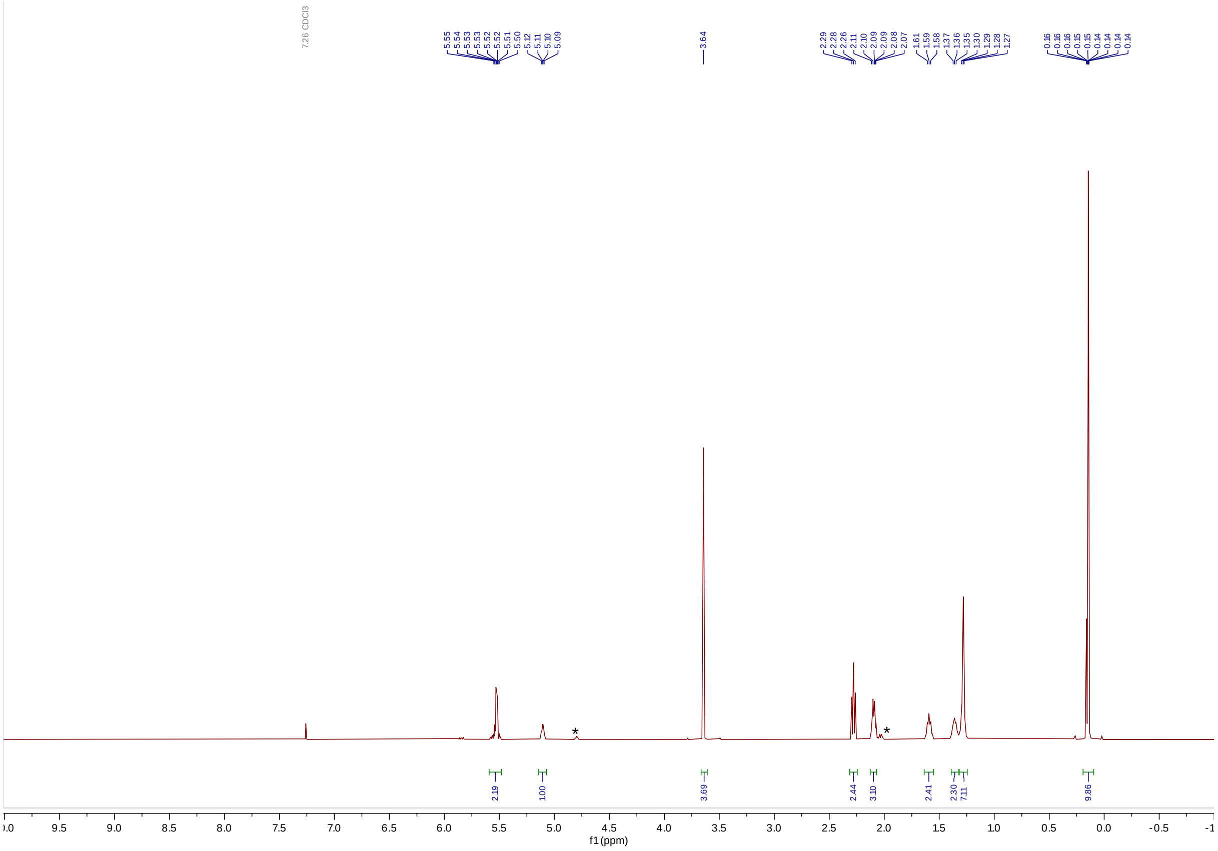

^{13}C-NMR (125.65 MHz, $CDCl_3$) of **6a** (see Procedure)

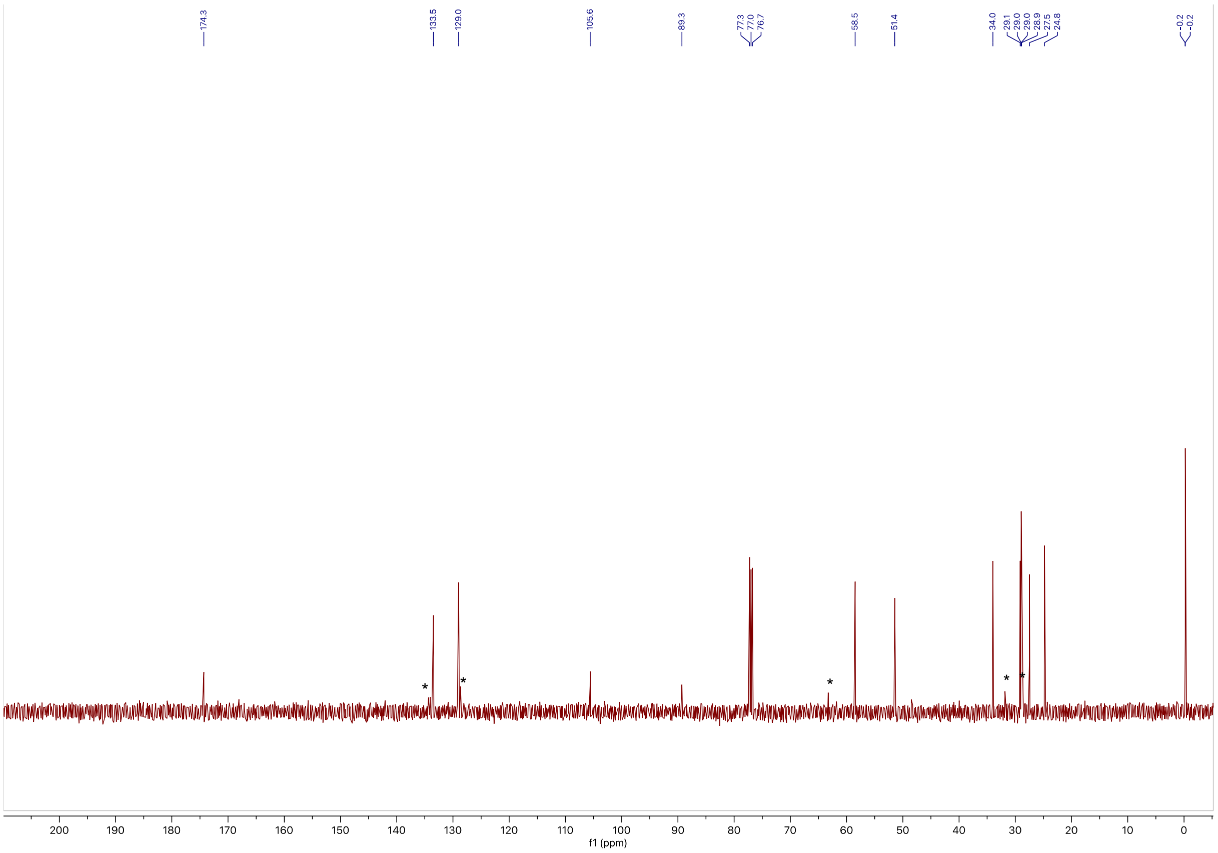

^{1}H-NMR (499.64 MHz, $CDCl_3$) of **SI7** (see Procedure)

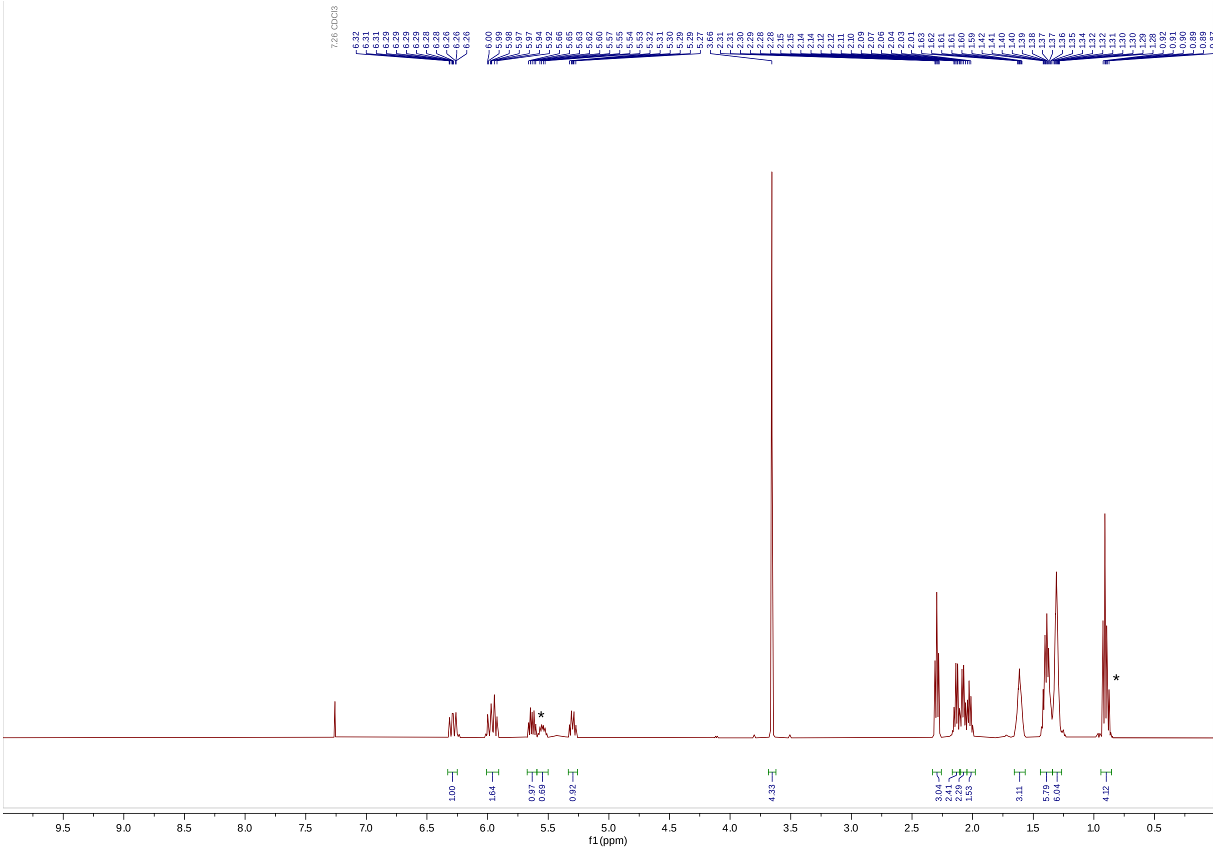

^{13}C-NMR (125.65 MHz, $CDCl_3$) of **SI7** (see Procedure)

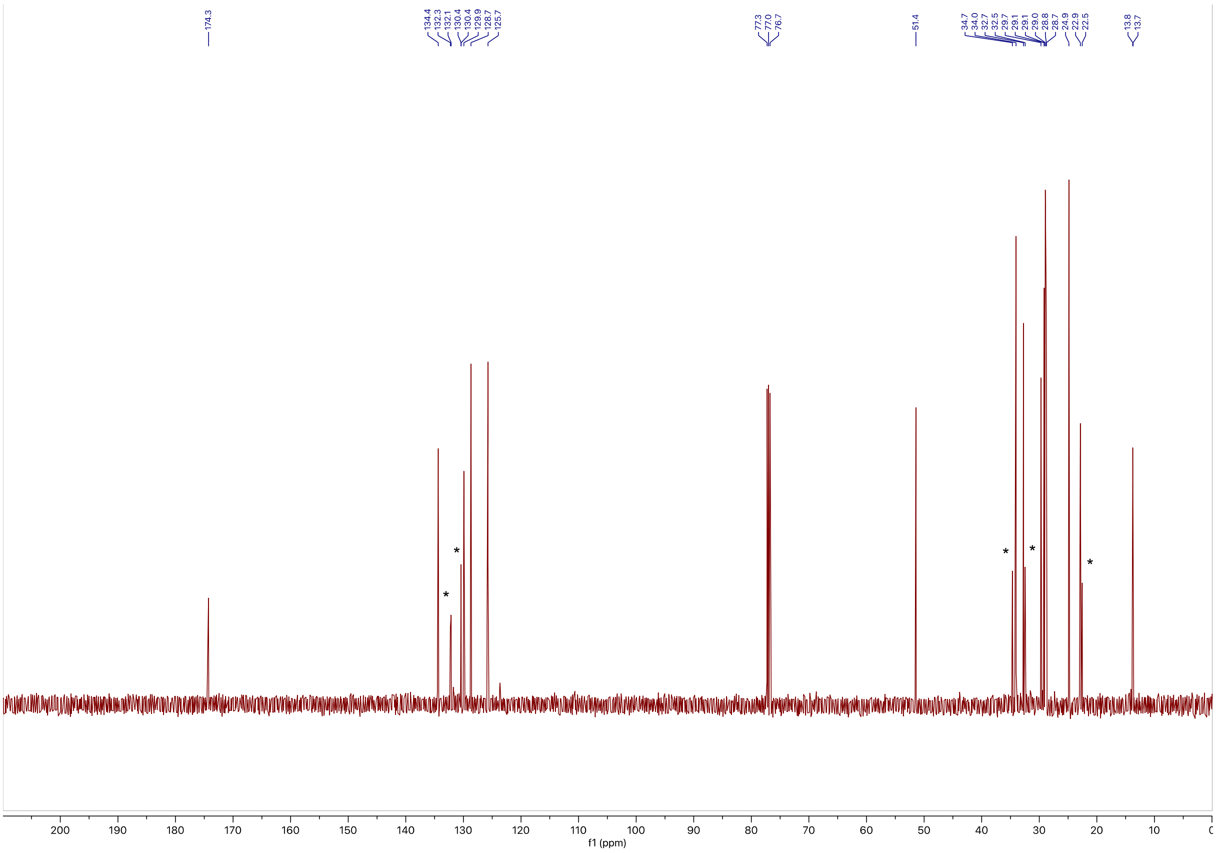

^{1}H-NMR (499.64 MHz, $CDCl_3$) of **7** (see Procedure)

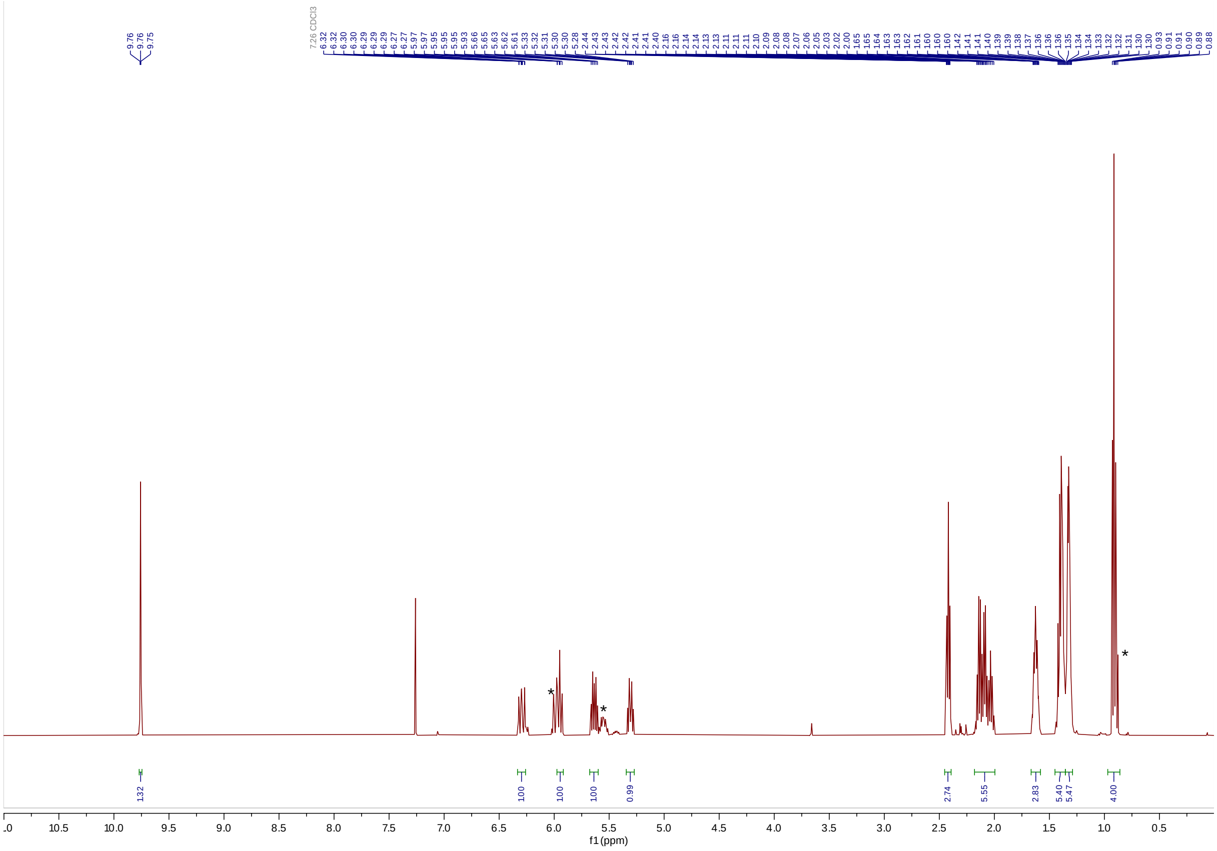

^{13}C-NMR (125.65 MHz, $CDCl_3$) of **7** (see Procedure)

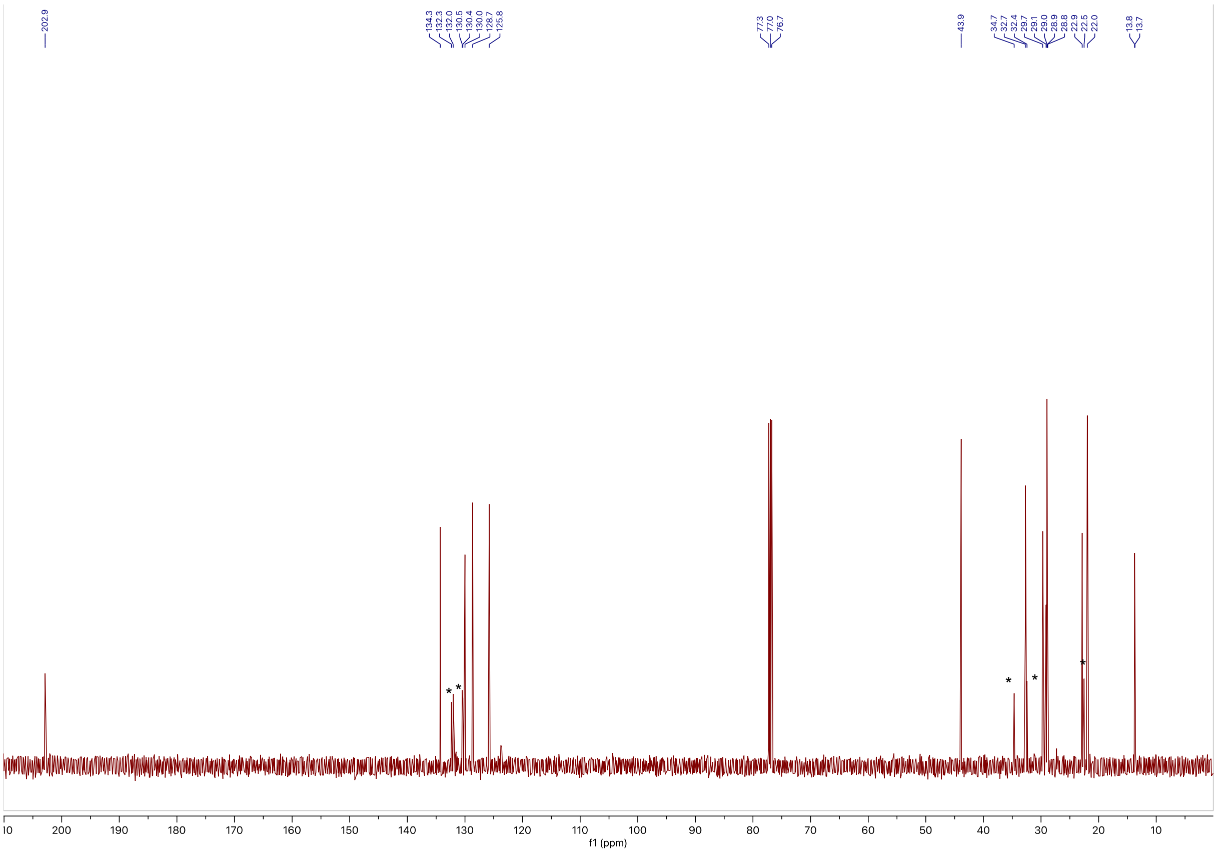

^{1}H-NMR (499.64 MHz, $CDCl_3$) of **8** (see Procedure)

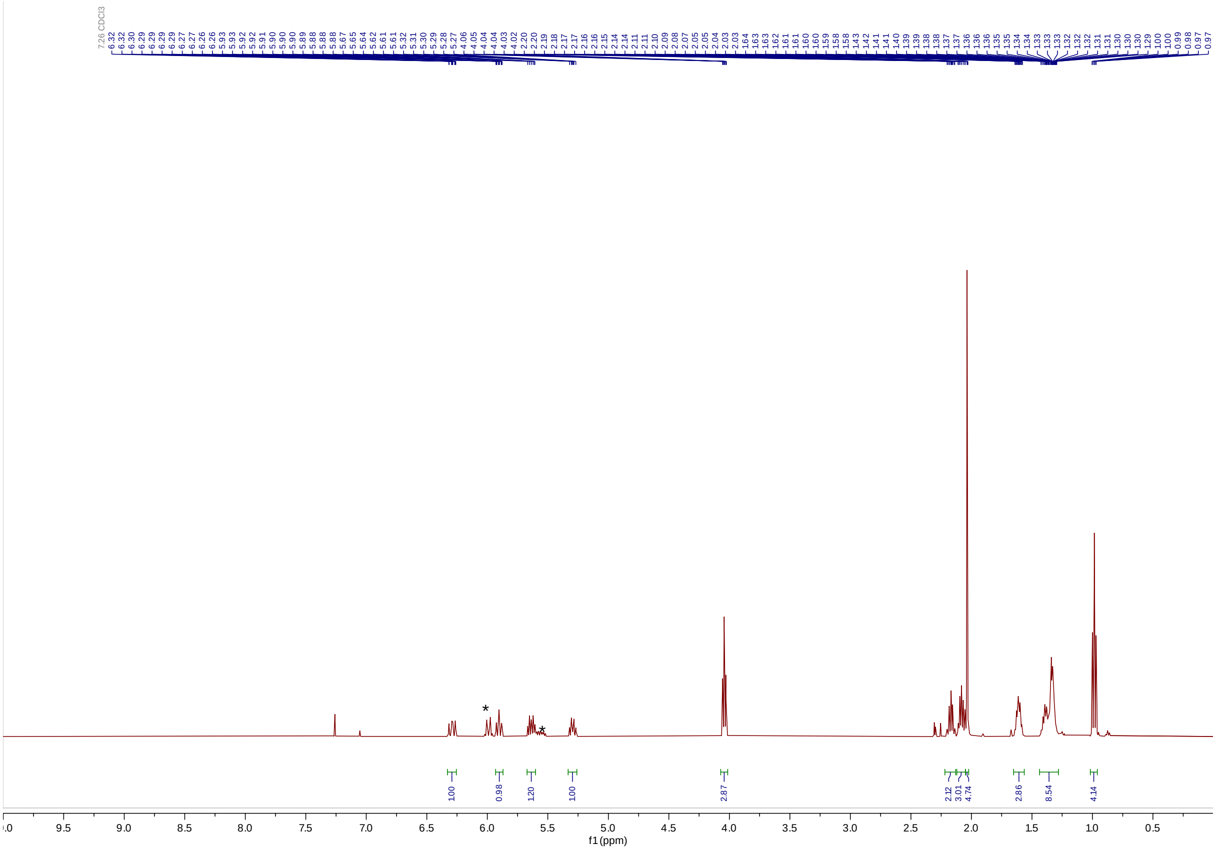

^{13}C-NMR (125.65 MHz, $CDCl_3$) of **8** (see Procedure)

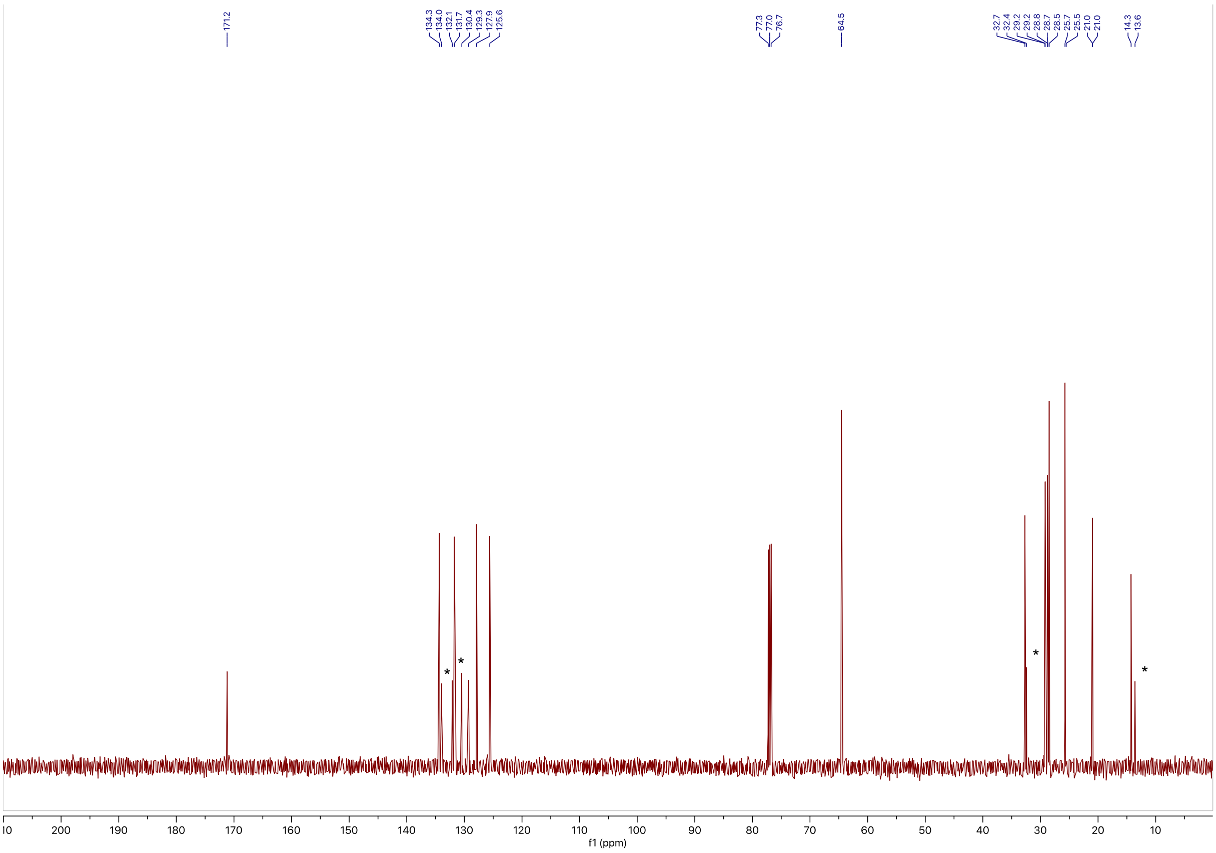

¹H-NMR (499.64 MHz, $CDCl_3$) of **9** (see Procedure)

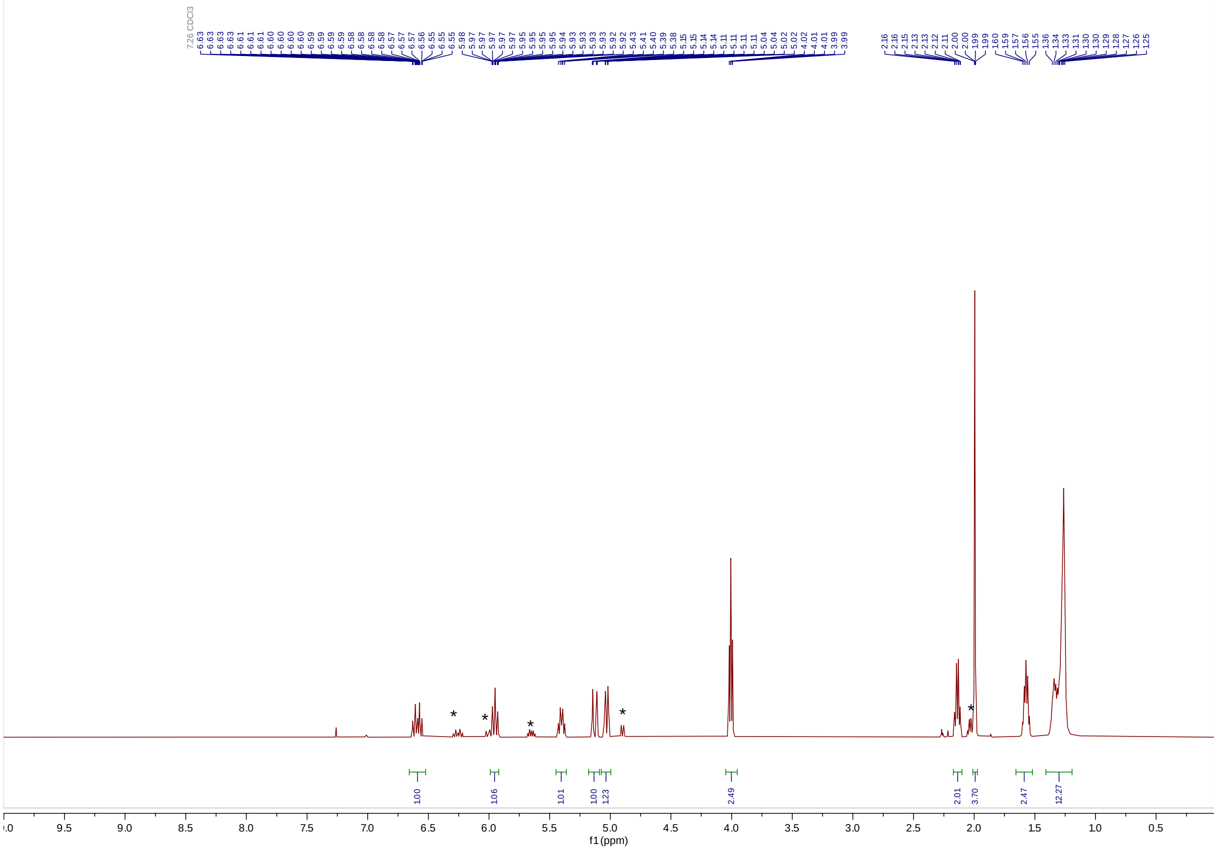

¹³C-NMR (125.65 MHz, $CDCl_3$) of **9** (see Procedure)

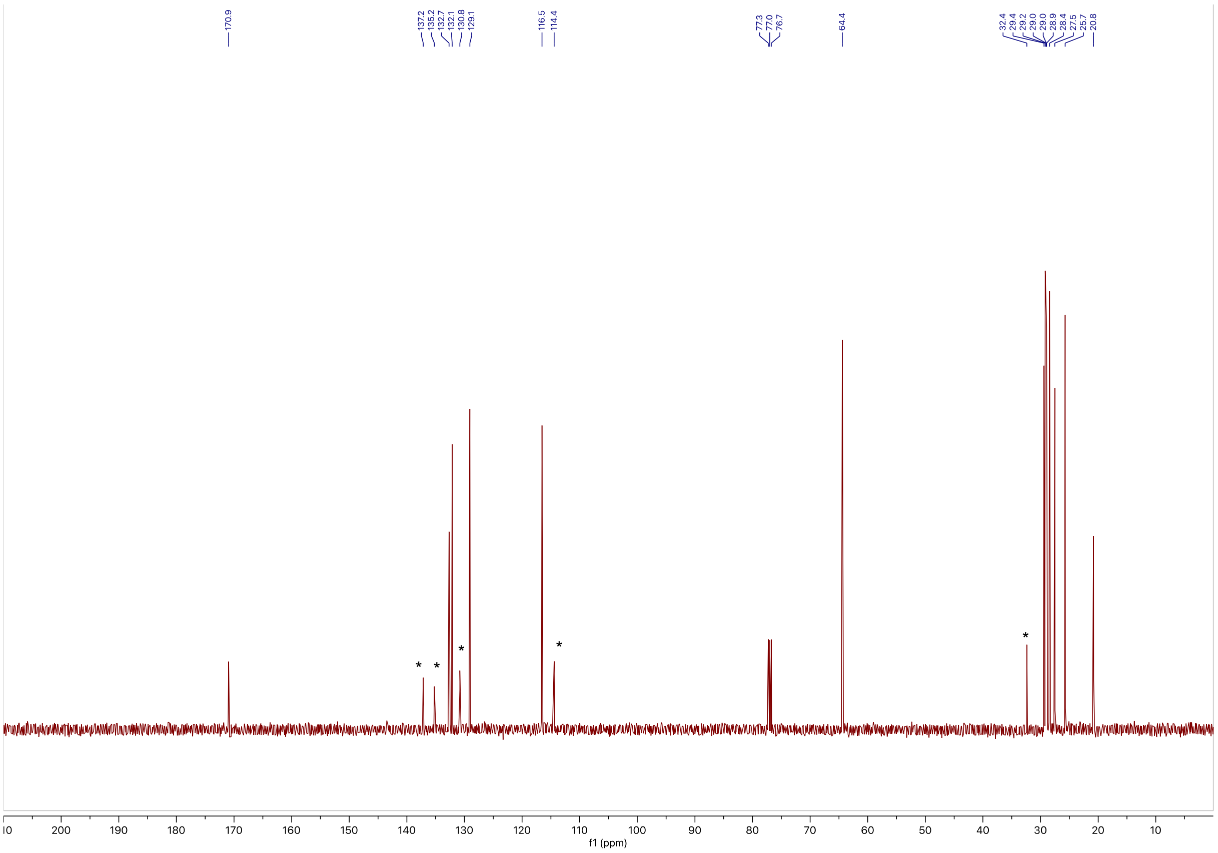

^{1}H-NMR (499.64 MHz, $CDCl_3$) of **10** (see Procedure)

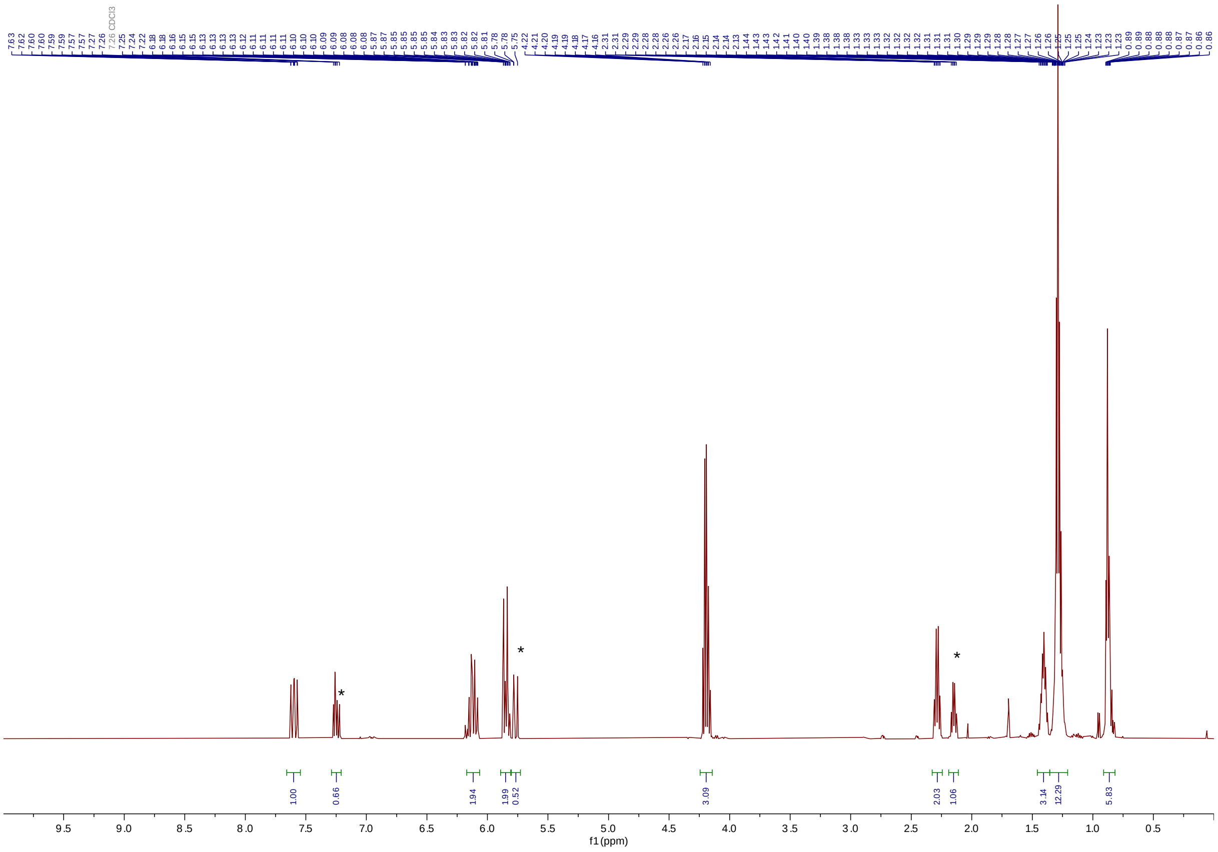

^{13}C-NMR (125.65 MHz, $CDCl_3$) of **10** (see Procedure)

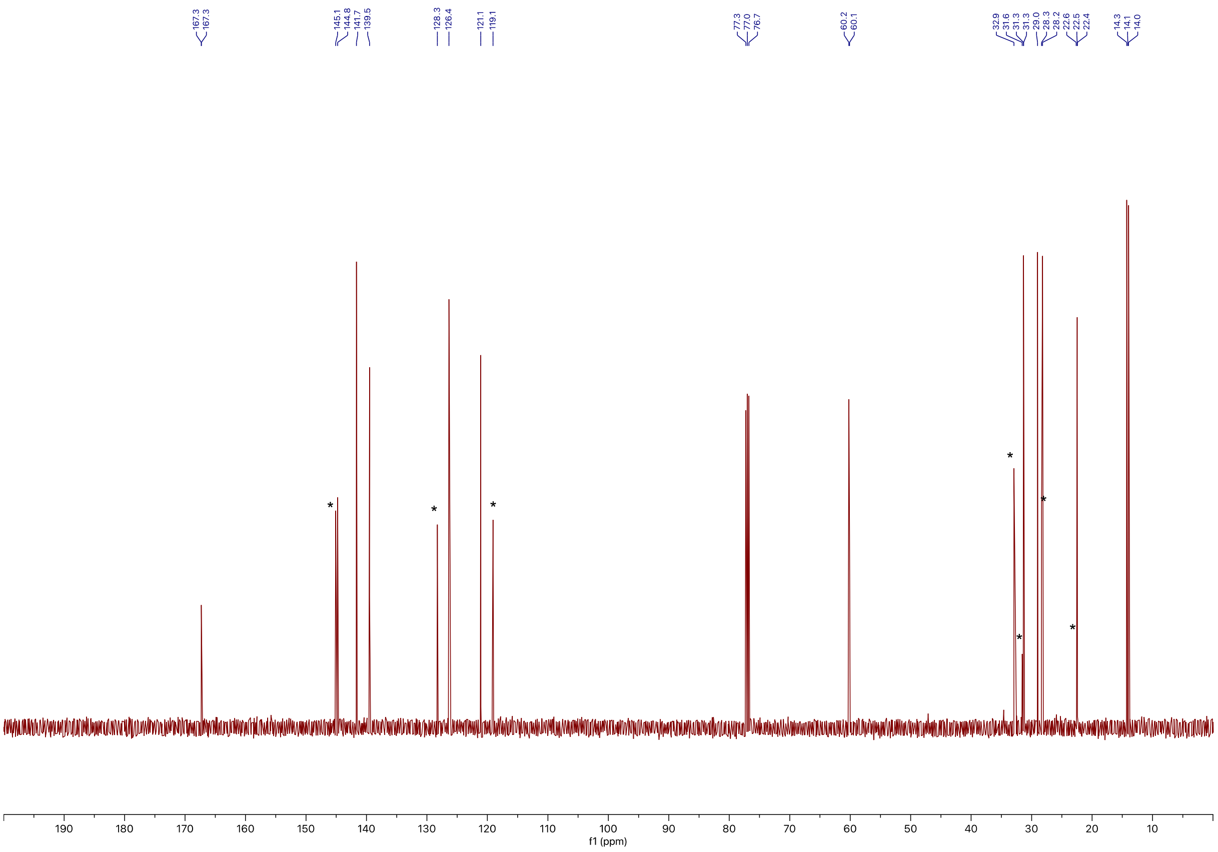

Supplement: Supplementary file 1 — Supporting Information [file ANIE-62-0-s004.pdf]
